# The meaning of alignment: lessons from structural diversity

**DOI:** 10.1186/1471-2105-9-556

**Published:** 2008-12-23

**Authors:** Walter Pirovano, K Anton Feenstra, Jaap Heringa

**Affiliations:** 1 Centre for Integrative Bioinformatics VU (IBIVU), VU University Amsterdam, De Boelelaan 1081A, 1081HV Amsterdam, the Netherlands

## Abstract

**Background:**

Protein structural alignment provides a fundamental basis for deriving principles of functional and evolutionary relationships. It is routinely used for structural classification and functional characterization of proteins and for the construction of sequence alignment benchmarks. However, the available techniques do not fully consider the implications of protein structural diversity and typically generate a single alignment between sequences.

**Results:**

We have taken alternative protein crystal structures and generated simulation snapshots to explicitly investigate the impact of structural changes on the alignments. We show that structural diversity has a significant effect on structural alignment. Moreover, we observe alignment inconsistencies even for modest spatial divergence, implying that the biological interpretation of alignments is less straightforward than commonly assumed. A salient example is the GroES 'mobile loop' where sub-Ångstrom variations give rise to contradictory sequence alignments.

**Conclusion:**

A comprehensive treatment of ambiguous alignment regions is crucial for further development of structural alignment applications and for the representation of alignments in general. For this purpose we have developed an on-line database containing our data and new ways of visualizing alignment inconsistencies, which can be found at .

## Background

Sequence comparison has become a major tool for biological research in the post-genomic era, forming the basis for functional annotation, classification, and analysis of evolutionary relationships. At the residue level, however, the relation between sequence, structure and function can often be obscure, and examples abound of proteins with a clear functional and homologous relationship but sharing negligible similarity at the sequence level.

Structural alignment therefore is the method of choice for reliable homology assessment and derived features like functional classification and phylogeny. This importance is reflected in the number of tools available for structural alignment, such as DALI [1], SSAP [2], STRUCTAL [3], MAMMOTH [4], CE [5] and COMPARER [6] (for recent reviews on the topic, see Kolodny et al. [7] and Mayr et al. [8]). Databases for functional classification such as CATH [9], FSSP [10] and PASS2 [11] each derive directly from the use of one or more of these methods, whereas for SCOP expert input in the structural classification is deemed critical [12]. Structural alignments are also routinely used for benchmarking sequence alignment methods. A number of databases have been developed for this purpose, among which BAliBASE [13], HOMSTRAD [14] and SABmark [15] are widely used. These databases often rely on expert knowledge and include a notion of 'core blocks', *i.e*. where alignment ambiguity does not occur and hence can be trusted. The general problem of uncertainty in sequence alignment has recently been discussed by Wong et al. [16]. Due to the complexity of interpreting non-trivial alignment regions, these are often omitted in large-scale evolutionary analyses, even though there is ample evidence for their fundamental importance [16,17]. An approach to pinpointing alignment ambiguity is the generation of ensembles of suboptimal alignments [18], but computational demands remain prohibitive for genome wide studies.

**Figure 1:**
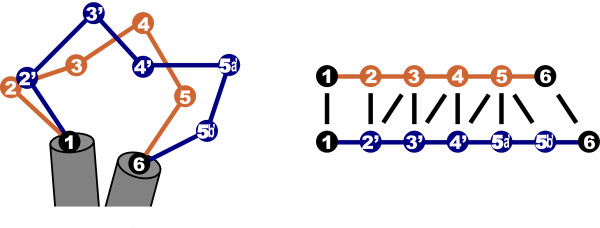
**Dealing with structural flexibility: a single insertion (5', left) can lead to ambiguity in the pairwise residue alignment between the loops (right)**. Therefore, a simple one-to-one functional equivalence between residues from different proteins may not exist.

Recent structural alignment methods have started to place emphasis on dealing with structural flexibility, such as FATCAT [19], MultiProt [20], MATT [21] and RAPIDO [22]. This may increase the consistency of alignments produced by each of these methods, but does not address the intrinsic ambiguity arising from structural divergence. The fundamental issue is whether a one-to-one equivalence exists between residues from different proteins that could be expressed as one definite alignment between sequences [18]. This is illustrated in Figure 1, where we show that a single insertion can lead to ambiguity in the functional correspondence between most residues in the loop.

**Figure 2:**
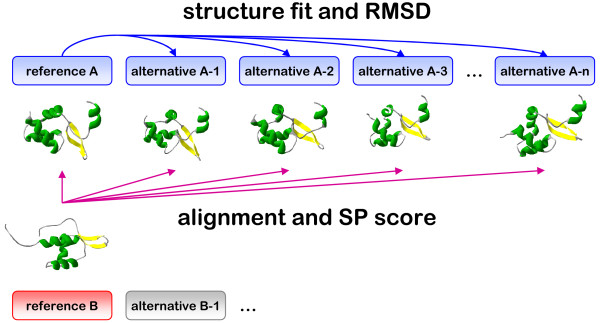
**Overview of the approach**. SP scores are calculated to describe the differences at the sequence level between the reference and alternative structural alignments. In addition each alternative structure (either obtained with molecular simulation or from the PDB) is fit onto the reference structure and root mean square deviations (RMSDs) are calculated.

To further elucidate the effect of structural diversity on structural alignment, we prepared two distinct comprehensive sets of alternative structures for proteins from the HOMSTRAD database of homologous protein families. The first set comprises proteins for which alternative crystal structures are available. The other set is derived from molecular dynamics simulations to explore a more extensive spectrum of possible structures. An overview of our analysis procedure is outlined in Figure 2.

Our main results show that in many cases structural variation strongly affects structural alignments, even for highly similar sequences. Moreover, the derived alignment appears to be highly sensitive to even small conformational changes of the proteins. The uncertainty in pairing up structural equivalent residues makes it difficult to determine which alignment alternative would describe most closely the functional relationship between the proteins. To address this issue, we show how alternative alignment visualizations may be used to exploit the information contained within variable alignment regions.

## Results and discussion

### Structural diversity and alignment stability

**Figure 3:**
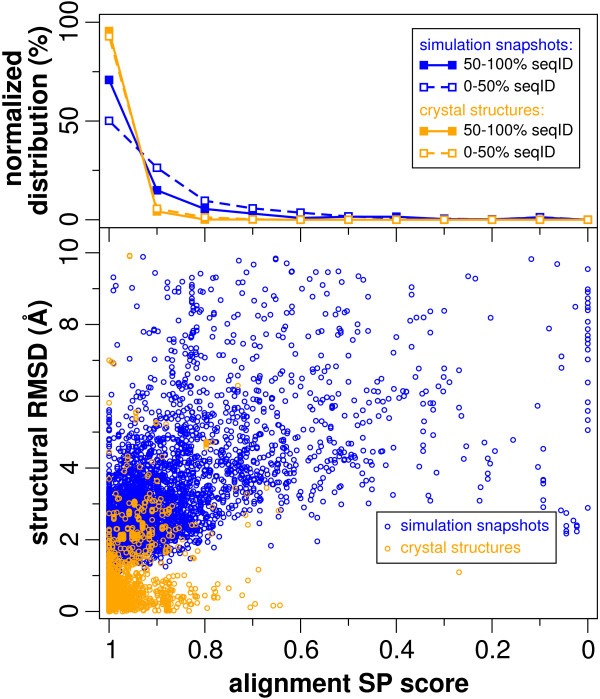
**Effects of structure and sequence variation on the alignment**. The bottom panel shows structural difference (measured by the RMSD) versus alignment similarity, measured by the SP score, which is defined as the fraction of aligned reference residues pairs that are reproduced in the query alignment. The top panel shows distributions of SP scores for alignments sharing less and more than 50% sequence identity. Orange (lighter) refers to alternative crystal structures while blue (darker) refers to alternative structures obtained from molecular simulations.

The relation between the variation of the alternative structures (RMSD) and the corresponding alignment similarity (SP score) is shown in Figure 3 (bottom panel). It is clear that the structural variation between crystal structures (in orange) is much smaller (up to 3–4 Å RMSD) than that of the simulation snapshots (in blue; up to 10 Å RMSD). A crucial aspect is that even for small (<1 Å RMSD) and modest (1–3 Å RMSD) structural differences, alignments can easily vary up to 20% and sometimes as much as 40% or more in their SP score. On the other hand, a considerable number of alignments appear robust to larger (up to 6 Å RMSD) and even extreme (up to 10 Å RMSD) structural variations. Additionally, for the crystal structures, the sequence similarity has no effect on the variation in structural alignments (Figure 3, top panel). For the simulation snapshots, however, there seems to be a slight but distinct tendency for more similar sequences to have less variation in structural alignments, but this can be mainly attributed to the larger variations (>3 Å RMSD) in structure that arise from the simulations (see additional file 1). As an alternative for RMSD measurements we also tested the rho-score [23], a protein size-independent measure, which resulted in the same trend (data not shown).

**Figure 4:**
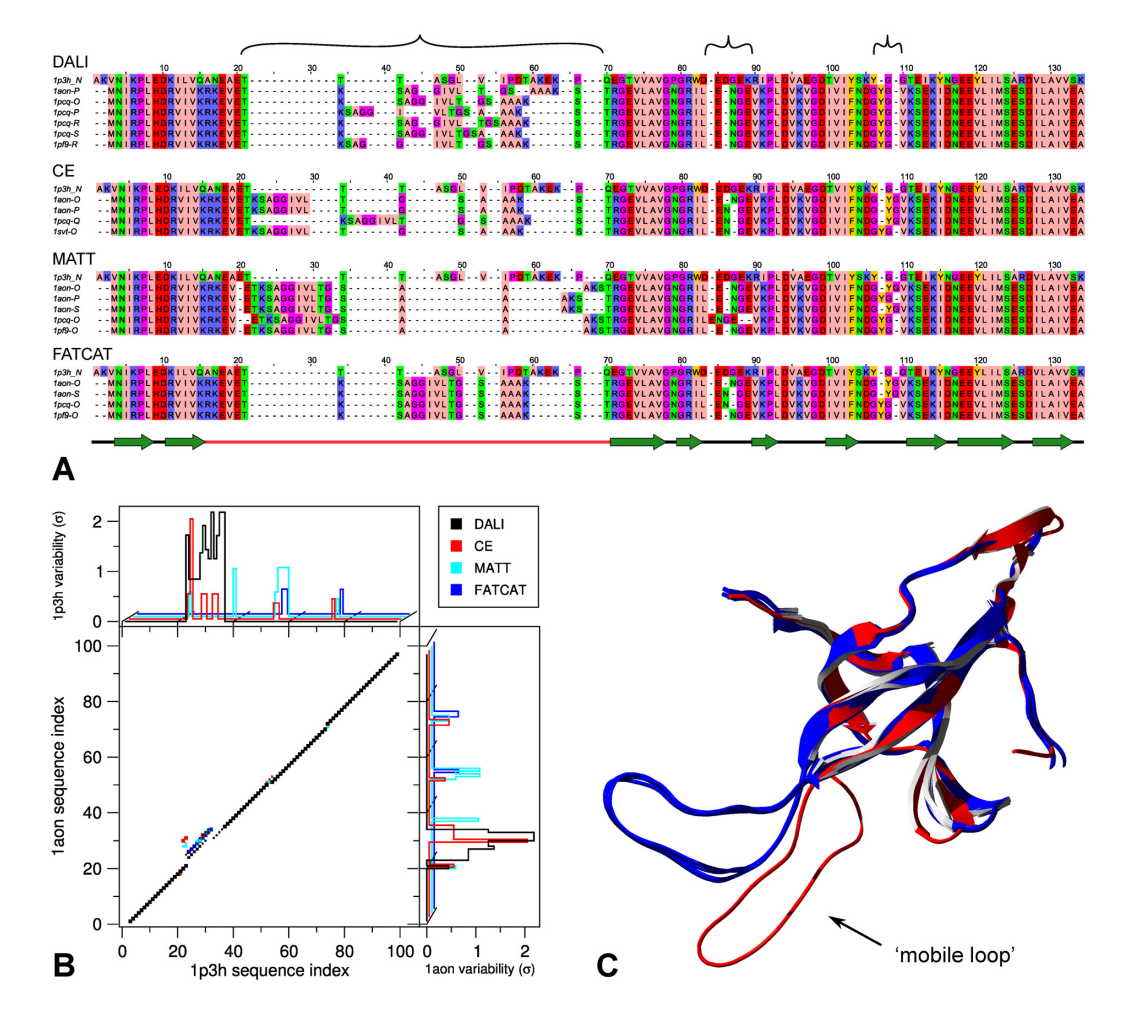
**An example of the impact of tiny structural variations in the GroES 'mobile loop' that lead to quite dramatic variations in the alignment**. A) The 'master-slave' alignments with 1p3h-N as master, variable regions marked at the top, and secondary structure with the mobile loop shown in red at the bottom. B) Alignment matrix with consistency plots along both axes give an overview of variability in each of the alignments from A). C) The different GroEs structures with 1p3h-N in red and 1aon-O and alternatives in blue. Alignment image created using JalView [33] with 'Zappo' colouring; secondary structure assignment according to Xu, et al. [24]. Protein structure rendered using SwissPDBViewer [34] and PovRay [35].

A quite interesting example of the impact that small structural variations can have on the structural alignment is found in the GroES so-called 'mobile loop', which is the main region for interaction with GroEL and therefore is a crucial component of the GroEL/ES chaperonin machinery [24]. The structural variations for this loop in *E. coli *GroES (Figure 4C, shown in blue) are almost negligible (whole protein Cα RMSDs 0.42 ± 0.13 Å). It is therefore surprising that the corresponding DALI sequence alignments with *M. tuberculosis *GroES show remarkable variation in this region (Figure 4A). To pinpoint the source of this variation, we also used three other structural alignment programs: CE, MATT and FATCAT. The latter two explicitly take structural flexibility into account and this leads to more consistent alignments in the variable loop (alignment positions 20–69, Figure 4A). On the other hand, two regions (84–89 and 107–109, Figure 4A) are aligned consistently by DALI but show inconsistencies when aligned by CE and the two flexibility-aware methods. Strikingly, there is no overall consistency between the four methods, which is in line with several other studies where several structural alignment methods are compared [7,8,18]. It should be stressed that the focus of this paper is not on comparing the performance of the various methods but rather on the effects of structural diversity. A comprehensive overview of the GroES variability is given by the alignment matrix and the consistency plots (Figure 4B). The alignment matrix scores the occurrence of aligned residue pairs over all alignments, similar to the dot-plot [25,26]. Consistency plots show for each residue the standard deviation from the alignment position of the consensus pair. The alignment matrix and associated consistency plots allow a detailed visualization of the variability while enabling easy interpretation of the ensemble of alternative alignments.

Although alignment uncertainty has been shown to have a great impact on large scale sequence analysis [16,17], the relation with structural variation has not been widely explored [27]. This is remarkable given that structural alignments are generally employed to benchmark sequence alignment methods. We demonstrate that in many cases structural alignments can vary dramatically even for small structural changes. Trends observed in the set of crystal structures corroborate those observed in the set of simulation snapshots, albeit alignment differences in the latter set are more pronounced due to larger structural variations.

### A depository for alignment variability

It is questionable whether a single reference alignment captures the full width of naturally occurring sequence variability [18]. Yet, current visualization and alignment methods are not designed to take variable regions into account, and they are typically ignored in sequence alignment benchmark protocols. Since variable regions are often important structurally and/or functionally, new approaches for visualization, alignment and benchmarking are desirable.

To this end we have constructed a database of 'flexible' reference alignments. This database is available online  and contains all structures and alignments used in this study. For each alignment in our database, variation is visualized using alignment matrices and consistency plots as shown in Figure 4B. In addition the database contains the ensemble 'master-slave' alignments as shown in Figure 4A. This pinpoints alignment regions that are affected by variability.

## Conclusion

Structural variation, as presented here by alternative crystal structures and molecular dynamics simulations, has a profound effect on structural alignment. The sensitivity to structural variation is a bottleneck for the effective application of structural alignment approaches. This undermines the current basis of all sequence alignment methodologies and is an underestimated problem for the homology assessment used in structural and functional classification. The GroES 'mobile loop' example demonstrates how functionally essential protein regions can coincide with variable structural alignment segments. Our database should therefore be useful for alignment verification and delineation of functionally important protein regions.

## Methods

The HOMSTRAD database of homologous structure alignments [14] was used as a source to select homologous proteins with known structure. HOMSTRAD families containing two homologous proteins (A and B in Figure 2) were selected. The corresponding structures were retrieved from the PDB [28] and taken as reference. For each reference structure, after equilibration, molecular dynamics simulations were performed for up to 10 ns, and snapshot structures were stored every 1 ns. Standard solvated conditions in the Gromos 43a1 forcefield [29] and the Gromacs simulation package [30] were used (details summarized in additional file 2). In addition, for each reference structure, we retrieved all alternative PDB structures with 100% sequence identity. In the subsequent analysis only the residues corresponding to the HOMSTRAD sequences were used.

From each pair of reference HOMSTRAD structures, we constructed reference alignments with the widely used structural alignment tool DALI [1]. We also used DALI to create pairwise alignments between each reference structure and the alternatives of the other reference structure (PDB and snapshots). The sequence differences between the alignments were calculated using Sum-of-Pairs (SP) scoring implemented in the BAliBASE alignment comparison tool [13]. SP scores range from 0 (non-identical) to 1 (identical sequence alignments). Finally we calculated the root mean square deviation (RMSD) between the Cα atoms of the alternative structures and their reference structure using the McLachlan algorithm [31] as implemented in the program ProFit version 2.5.3 [32].

Our final database consists of 496 proteins (divided over 341 families) for which 3309 snapshot structures could be made and 565 proteins (divided over 395 families) for which we found in total 2998 alternative crystal structures with redundant sequences. A full list of all aligned structures and relevant details is provided in additional file 3.

## Authors' contributions

All authors designed the research, analyzed the results and wrote the paper. WP and KAF performed the research. All authors read and approved the final manuscript.

## Supplementary Material

Additional file 1 **Figure S1: Combined effects of structural variation and sequence variation on the alignment.** Click here for file

Additional file 2 **Table S1: Molecular Dynamics simulation set-up.** Click here for file

Additional file 3 **Table S2: Details of aligned Crystal Structures (a) and Simulation Snapshots (b).** Click here for file
