# Supplementary figures and images for: The meaning of alignment: lessons from structural diversity
Source: BMC Bioinformatics. 2008 Dec 23;9:556. doi: 10.1186/1471-2105-9-556 (PMC2630330; doi:10.1186/1471-2105-9-556)

Combined effects of structural variation and sequence variation on the alignment.

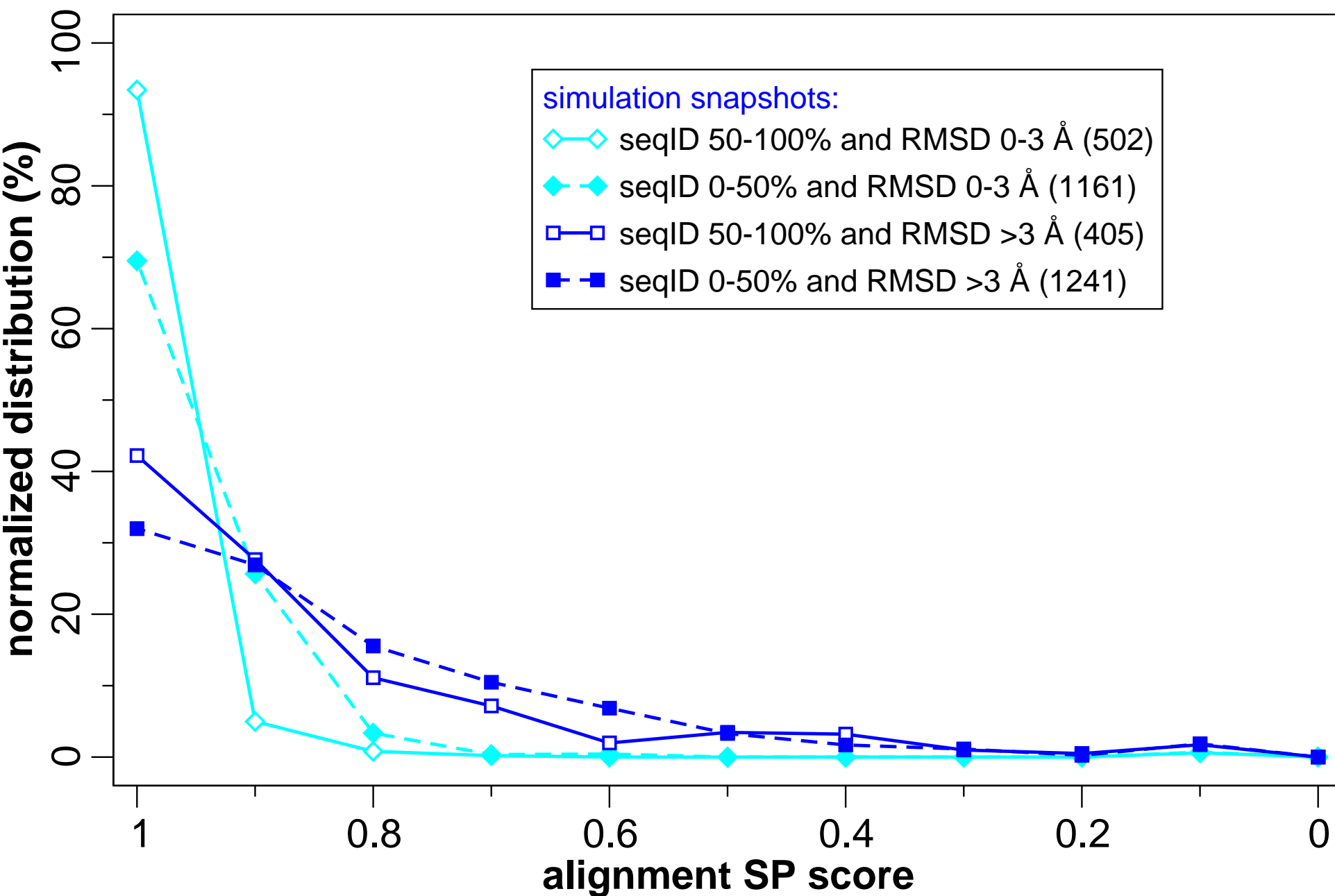

Supplement: Additional file 1 — Figure S1: Combined effects of structural variation and sequence variation on the alignment. [file 1471-2105-9-556-S1.pdf]
