# Supplementary material for: The meaning of alignment: lessons from structural diversity
Source: BMC Bioinformatics. 2008 Dec 23;9:556. doi: 10.1186/1471-2105-9-556 (PMC2630330; doi:10.1186/1471-2105-9-556)
Supplement: Additional file 2 — Table S1: Molecular Dynamics simulation set-up. [file 1471-2105-9-556-S2.pdf]

## Supplementary Material Table S1: Molecular Dynamics simulation set-up



| Parameter/feature | Setting |
|---|---|
| MD package | Gromacs 3.1.4 |
| forcefield | gromos 43a1 |
| cut-off | twin-range 0.8/1.2 nm |
| neighbour list update | 5 steps |
| bond constraints | lincs algorithm |
| time step $\Delta t$ | 2 fs |
| Temperature T | 300 K |
| weak coupling time $\tau_T$ | 1 ps |
| pressure P | 1 bar |
| weak coupling time $\tau_P$ | 0.1 p |
| position restraints $f_c$ | 1000 kJ $mol^{-1}$ $nm^{-1}$ |

**Equilibration:**

- energy minimized of protein in vacuum; non-hydrogen atoms harmonic position restrained
- energy minimized of protein in vacuum; no position restraints
- solvation in periodic cubic box pre-equilibrated simple point-charge (SPC) water with 1.2 nm minimum distance
- energy minimization of solvated system
- random starting velocities from 300 K Maxwell distribution
- water relaxation during 1 ps with protein position restrained
