# Supplementary material for: The meaning of alignment: lessons from structural diversity
Source: BMC Bioinformatics. 2008 Dec 23;9:556. doi: 10.1186/1471-2105-9-556 (PMC2630330; doi:10.1186/1471-2105-9-556)
Supplement: Additional file 3 — Table S2: Details of aligned Crystal Structures (a) and Simulation Snapshots (b). [file 1471-2105-9-556-S3.pdf]

Supplementary Material Table S2a: Details of aligned Crystal Structures −− The Meaning of Alignment (Pirovano, Feenstra & Heringa)

| n | length | %ID | refA−refB | altA | SP | RMSD |
|---|---|---|---|---|---|---|
| 4 | 341 | 90 | 1qme−1k25 | 1qmf_A | 1.000 | 0.775 |
| 8 | 305 | 88 | 1g9m−1g9n | 1rzj_G | 1.000 | 0.460 |
| 8 | 305 | 88 | 1g9n−1g9m | 1rzk_G | 1.000 | 0.123 |
| 9 | 98 | 87 | 1ljp−1lri | 2a8f_B | 1.000 | 0.681 |
| 9 | 98 | 87 | 1ljp−1lri | 2aib_A | 1.000 | 0.696 |
| 9 | 98 | 87 | 1ljp−1lri | 2aib_B | 1.000 | 0.816 |
| 9 | 98 | 87 | 1ljp−1lri | 1ljp_B | 1.000 | 0.342 |
| 9 | 98 | 87 | 1ljp−1lri | 2a8f_A | 1.000 | 0.572 |
| 13 | 62 | 85 | 1fx7−2dtr | 1fx7_D | 1.000 | 0.331 |
| 13 | 62 | 85 | 1fx7−2dtr | 1u8r_A | 1.000 | 0.477 |
| 13 | 62 | 85 | 1fx7−2dtr | 1u8r_D | 1.000 | 0.479 |
| 13 | 62 | 85 | 2dtr−1fx7 | 1bi0_A | 1.000 | 0.387 |
| 13 | 62 | 85 | 2dtr−1fx7 | 1bi1_A | 1.000 | 0.353 |
| 13 | 62 | 85 | 2dtr−1fx7 | 1dpr_A | 1.000 | 1.709 |
| 13 | 62 | 85 | 1fx7−2dtr | 1u8r_B | 1.000 | 0.481 |
| 13 | 62 | 85 | 1fx7−2dtr | 1u8r_C | 1.000 | 0.473 |
| 13 | 62 | 85 | 1fx7−2dtr | 1u8r_I | 1.000 | 0.475 |
| 13 | 62 | 85 | 2dtr−1fx7 | 1bi2_B | 1.000 | 1.498 |
| 13 | 62 | 85 | 2dtr−1fx7 | 1dpr_B | 1.000 | 1.796 |
| 13 | 62 | 85 | 1fx7−2dtr | 1fx7_B | 1.000 | 0.379 |
| 13 | 62 | 85 | 1fx7−2dtr | 1u8r_G | 1.000 | 0.481 |
| 13 | 62 | 85 | 1fx7−2dtr | 1u8r_H | 1.000 | 0.474 |
| 13 | 62 | 85 | 1fx7−2dtr | 1u8r_J | 1.000 | 0.481 |
| 13 | 62 | 85 | 2dtr−1fx7 | 1bi3_A | 1.000 | 0.621 |
| 13 | 62 | 85 | 1fx7−2dtr | 1fx7_C | 1.000 | 0.464 |
| 13 | 62 | 85 | 2dtr−1fx7 | 1bi2_A | 1.000 | 1.400 |
| 13 | 62 | 85 | 2dtr−1fx7 | 1bi3_B | 1.000 | 0.624 |
| 15 | 65 | 84 | 1azp−1c8c | 1wd0_A | 1.000 | 1.309 |
| 15 | 65 | 84 | 1azp−1c8c | 1azq_A | 1.000 | 0.581 |
| 15 | 65 | 84 | 1azp−1c8c | 1ca6_A | 1.000 | 0.483 |
| 15 | 65 | 84 | 1azp−1c8c | 1sap_A | 0.984 | 2.761 |
| 17 | 152 | 84 | 1d09−2atc | 1rag_D | 0.861 | 2.286 |
| 17 | 152 | 84 | 1d09−2atc | 1raa_B | 0.875 | 2.836 |
| 17 | 152 | 84 | 1d09−2atc | 1rab_B | 0.882 | 2.812 |
| 17 | 152 | 84 | 1d09−2atc | 1raf_B | 0.910 | 2.841 |
| 17 | 152 | 84 | 1d09−2atc | 1i5o_B | 0.910 | 1.913 |
| 17 | 152 | 84 | 1d09−2atc | 1rae_D | 0.854 | 2.322 |
| 17 | 152 | 84 | 1d09−2atc | 1tug_D | 0.931 | 2.134 |
| 17 | 152 | 84 | 1d09−2atc | 1r0b_K | 0.882 | 1.627 |
| 17 | 152 | 84 | 1d09−2atc | 1xjw_D | 0.910 | 2.154 |
| 17 | 152 | 84 | 1d09−2atc | 1rad_D | 0.889 | 2.299 |
| 17 | 152 | 84 | 1d09−2atc | 1rah_D | 0.875 | 2.271 |
| 17 | 152 | 84 | 1d09−2atc | 1rad_B | 0.875 | 2.829 |
| 17 | 152 | 84 | 1d09−2atc | 1i5o_D | 0.875 | 2.110 |
| 17 | 152 | 84 | 1d09−2atc | 1raf_D | 0.889 | 2.352 |
| 17 | 152 | 84 | 1d09−2atc | 1tth_B | 0.882 | 0.813 |
| 17 | 152 | 84 | 1d09−2atc | 1r0b_L | 0.819 | 2.419 |
| 17 | 152 | 84 | 1d09−2atc | 1tth_D | 0.868 | 2.102 |
| 17 | 152 | 84 | 1d09−2atc | 1rab_D | 0.889 | 2.304 |
| 17 | 152 | 84 | 1d09−2atc | 1ezz_D | 0.924 | 3.039 |
| 17 | 152 | 84 | 1d09−2atc | 2ipo_D | 0.910 | 2.286 |
| 17 | 152 | 84 | 1d09−2atc | 1ezz_B | 0.889 | 2.798 |
| 17 | 152 | 84 | 1d09−2atc | 1rah_B | 0.910 | 2.786 |
| 17 | 152 | 84 | 1d09−2atc | 2ipo_B | 0.882 | 0.878 |
| 17 | 152 | 84 | 1d09−2atc | 2air_H | 0.889 | 4.319 |
| 17 | 152 | 84 | 1d09−2atc | 1r0c_H | 0.868 | 2.942 |
| 17 | 152 | 84 | 1d09−2atc | 1raa_D | 0.889 | 2.341 |
| 17 | 152 | 84 | 1d09−2atc | 1d09_D | 0.910 | 2.205 |
| 17 | 152 | 84 | 1d09−2atc | 1rac_D | 0.889 | 2.310 |
| 17 | 152 | 84 | 1d09−2atc | 1rai_B | 0.910 | 2.815 |
| 17 | 152 | 84 | 1d09−2atc | 1rag_B | 0.875 | 2.770 |
| 17 | 152 | 84 | 1d09−2atc | 1f1b_D | 0.924 | 2.366 |
| 17 | 152 | 84 | 1d09−2atc | 1r0c_B | 0.896 | 2.936 |
| 17 | 152 | 84 | 1d09−2atc | 1rae_B | 0.903 | 2.851 |
| 17 | 152 | 84 | 1d09−2atc | 1tu0_D | 0.847 | 2.004 |
| 17 | 152 | 84 | 1d09−2atc | 1rac_B | 0.875 | 2.859 |
| 17 | 152 | 84 | 1d09−2atc | 1f1b_B | 0.944 | 0.757 |
| 17 | 152 | 84 | 1d09−2atc | 1q95_G | 0.910 | 2.555 |
| 17 | 152 | 84 | 1d09−2atc | 1q95_J | 0.882 | 1.675 |
| 17 | 152 | 84 | 1d09−2atc | 1q95_K | 0.882 | 2.374 |
| 17 | 152 | 84 | 1d09−2atc | 1q95_I | 0.715 | 2.688 |
| 17 | 152 | 84 | 1d09−2atc | 1q95_H | 0.882 | 1.711 |
| 17 | 152 | 84 | 1d09−2atc | 1q95_L | 0.646 | 2.812 |
| 17 | 152 | 84 | 1d09−2atc | 1r0b_G | 0.910 | 1.831 |
| 17 | 152 | 84 | 1d09−2atc | 1r0b_H | 0.875 | 1.750 |
| 17 | 152 | 84 | 1d09−2atc | 1r0b_I | 0.917 | 2.280 |
| 17 | 152 | 84 | 1d09−2atc | 1r0b_J | 0.896 | 3.049 |
| 17 | 152 | 84 | 1d09−2atc | 1tu0_B | 0.875 | 1.702 |
| 17 | 152 | 84 | 1d09−2atc | 1tug_B | 0.951 | 1.776 |
| 17 | 152 | 84 | 1d09−2atc | 1xjw_B | 0.951 | 0.216 |
| 17 | 152 | 84 | 1d09−2atc | 2air_B | 0.910 | 4.176 |
| 18 | 317 | 84 | 4lip−1cvl | 2lip_A | 0.977 | 0.774 |
| 18 | 317 | 84 | 4lip−1cvl | 4lip_E | 1.000 | 0.188 |
| 18 | 317 | 84 | 4lip−1cvl | 1hqd_A | 0.994 | 0.330 |
| 18 | 317 | 84 | 4lip−1cvl | 3lip_A | 0.997 | 0.371 |
| 18 | 317 | 84 | 4lip−1cvl | 2nw6_A | 1.000 | 0.313 |
| 18 | 317 | 84 | 4lip−1cvl | 1oil_A | 0.981 | 0.368 |
| 18 | 317 | 84 | 1cvl−4lip | 2es4_A | 0.977 | 0.950 |
| 18 | 317 | 84 | 4lip−1cvl | 5lip_A | 1.000 | 0.357 |
| 18 | 317 | 84 | 4lip−1cvl | 1oil_B | 0.990 | 0.384 |
| 20 | 213 | 83 | 1n45−1dvg | 1n45_B | 1.000 | 0.529 |
| 20 | 213 | 83 | 1n45−1dvg | 1ozr_B | 1.000 | 0.552 |
| 20 | 213 | 83 | 1dvg−1n45 | 1dvg_B | 1.000 | 0.250 |
| 20 | 213 | 83 | 1n45−1dvg | 1twn_B | 1.000 | 0.554 |
| 20 | 213 | 83 | 1n45−1dvg | 1s8c_C | 1.000 | 0.639 |
| 20 | 213 | 83 | 1n45−1dvg | 1ozr_A | 1.000 | 0.321 |
| 20 | 213 | 83 | 1n45−1dvg | 1ozw_B | 1.000 | 0.545 |
| 20 | 213 | 83 | 1n45−1dvg | 1twr_B | 1.000 | 0.574 |
| 20 | 213 | 83 | 1n45−1dvg | 1twn_A | 1.000 | 0.324 |
| 20 | 213 | 83 | 1n45−1dvg | 1n3u_B | 1.000 | 0.513 |
| 20 | 213 | 83 | 1n45−1dvg | 1s8c_B | 1.000 | 0.732 |
| 20 | 213 | 83 | 1n45−1dvg | 1ozw_A | 1.000 | 0.158 |
| 20 | 213 | 83 | 1n45−1dvg | 1s13_B | 1.000 | 0.499 |
| 20 | 213 | 83 | 1n45−1dvg | 1n3u_A | 1.000 | 0.516 |
| 20 | 213 | 83 | 1n45−1dvg | 1twr_A | 1.000 | 0.346 |
| 20 | 213 | 83 | 1n45−1dvg | 1s8c_A | 1.000 | 0.591 |
| 20 | 213 | 83 | 1n45−1dvg | 1s13_A | 1.000 | 0.380 |
| 23 | 58 | 83 | 1qme−1k25 | 1qmf_A | 1.000 | 0.346 |
| 25 | 124 | 82 | 1grw−1msp | 1grw_D | 1.000 | 0.576 |
| 25 | 124 | 82 | 1grw−1msp | 1grw_B | 1.000 | 0.321 |
| 25 | 124 | 82 | 1grw−1msp | 1grw_C | 1.000 | 0.447 |
| 26 | 511 | 82 | 1mty−1mhy | 1mmo_D | 0.988 | 0.488 |
| 26 | 511 | 82 | 1mty−1mhy | 1mmo_E | 0.986 | 0.598 |
| 26 | 511 | 82 | 1mhy−1mty | 1mhz_D | 1.000 | 0.257 |
| 26 | 511 | 82 | 1mty−1mhy | 1mty_E | 1.000 | 0.196 |
| 28 | 68 | 82 | 1c9s−1wap | 1gtf_D | 1.000 | 0.163 |
| 28 | 68 | 82 | 1c9s−1wap | 1qaw_E | 1.000 | 0.302 |
| 28 | 68 | 82 | 1wap−1c9s | 1wap_M | 1.000 | 0.235 |
| 28 | 68 | 82 | 1wap−1c9s | 1wap_P | 1.000 | 0.443 |
| 28 | 68 | 82 | 1c9s−1wap | 1gtf_C | 1.000 | 0.145 |
| 28 | 68 | 82 | 1c9s−1wap | 1qaw_C | 1.000 | 0.225 |
| 28 | 68 | 82 | 1c9s−1wap | 1c9s_G | 1.000 | 0.139 |
| 28 | 68 | 82 | 1c9s−1wap | 1utd_H | 1.000 | 0.200 |
| 28 | 68 | 82 | 1c9s−1wap | 1c9s_I | 1.000 | 0.200 |
| 28 | 68 | 82 | 1c9s−1wap | 1gtn_H | 1.000 | 0.209 |
| 28 | 68 | 82 | 1c9s−1wap | 1utd_Q | 1.000 | 0.496 |
| 28 | 68 | 82 | 1c9s−1wap | 1gtf_M | 1.000 | 0.250 |
| 28 | 68 | 82 | 1c9s−1wap | 1gtf_F | 1.000 | 0.136 |
| 28 | 68 | 82 | 1c9s−1wap | 1utd_L | 1.000 | 0.471 |
| 28 | 68 | 82 | 1wap−1c9s | 1wap_S | 1.000 | 0.450 |
| 28 | 68 | 82 | 1c9s−1wap | 1c9s_D | 1.000 | 0.176 |
| 28 | 68 | 82 | 1c9s−1wap | 1c9s_F | 1.000 | 0.144 |
| 28 | 68 | 82 | 1c9s−1wap | 1gtn_K | 1.000 | 0.217 |
| 28 | 68 | 82 | 1c9s−1wap | 1c9s_M | 1.000 | 0.300 |
| 28 | 68 | 82 | 1c9s−1wap | 1utd_R | 1.000 | 0.264 |
| 28 | 68 | 82 | 1c9s−1wap | 1utd_B | 1.000 | 0.245 |
| 28 | 68 | 82 | 1wap−1c9s | 1wap_F | 1.000 | 0.437 |
| 28 | 68 | 82 | 1c9s−1wap | 1gtn_F | 1.000 | 0.195 |
| 28 | 68 | 82 | 1c9s−1wap | 1qaw_B | 1.000 | 0.469 |
| 28 | 68 | 82 | 1c9s−1wap | 1gtf_I | 1.000 | 0.173 |
| 28 | 68 | 82 | 1c9s−1wap | 1utv_D | 1.000 | 0.311 |
| 28 | 68 | 82 | 1c9s−1wap | 1gtf_K | 1.000 | 0.123 |
| 28 | 68 | 82 | 1c9s−1wap | 1c9s_H | 1.000 | 0.127 |
| 28 | 68 | 82 | 1c9s−1wap | 1c9s_K | 1.000 | 0.089 |
| 28 | 68 | 82 | 1c9s−1wap | 1gtn_G | 1.000 | 0.200 |
| 28 | 68 | 82 | 1c9s−1wap | 1gtn_M | 1.000 | 0.279 |
| 28 | 68 | 82 | 1c9s−1wap | 1gtn_O | 1.000 | 0.295 |
| 28 | 68 | 82 | 1wap−1c9s | 1wap_I | 1.000 | 0.440 |
| 28 | 68 | 82 | 1c9s−1wap | 1gtf_G | 1.000 | 0.142 |
| 28 | 68 | 82 | 1c9s−1wap | 1utd_O | 1.000 | 0.357 |
| 28 | 68 | 82 | 1c9s−1wap | 1gtf_A | 1.000 | 0.125 |
| 28 | 68 | 82 | 1c9s−1wap | 1qaw_D | 1.000 | 0.255 |
| 28 | 68 | 82 | 1c9s−1wap | 1c9s_O | 1.000 | 0.236 |
| 28 | 68 | 82 | 1c9s−1wap | 1utd_D | 1.000 | 0.218 |
| 28 | 68 | 82 | 1c9s−1wap | 1gtn_D | 1.000 | 0.195 |
| 28 | 68 | 82 | 1c9s−1wap | 1utd_J | 1.000 | 0.209 |
| 28 | 68 | 82 | 1c9s−1wap | 1gtn_I | 1.000 | 0.218 |
| 28 | 68 | 82 | 1c9s−1wap | 1utv_A | 1.000 | 0.524 |
| 28 | 68 | 82 | 1c9s−1wap | 1utv_B | 1.000 | 0.278 |
| 28 | 68 | 82 | 1c9s−1wap | 1utv_M | 1.000 | 0.347 |
| 28 | 68 | 82 | 1c9s−1wap | 1utf_K | 1.000 | 0.479 |
| 28 | 68 | 82 | 1c9s−1wap | 1utf_B | 1.000 | 0.244 |
| 31 | 440 | 81 | 1kpk−1kpl | 1kpk_C | 1.000 | 0.001 |
| 31 | 440 | 81 | 1kpk−1kpl | 1kpk_D | 1.000 | 0.001 |
| 31 | 440 | 81 | 1kpl−1kpk | 1kpl_C | 1.000 | 0.001 |
| 31 | 440 | 81 | 1kpl−1kpk | 1kpl_B | 0.988 | 0.986 |
| 31 | 440 | 81 | 1kpk−1kpl | 1kpk_E | 1.000 | 0.001 |
| 31 | 440 | 81 | 1kpl−1kpk | 1kpl_D | 0.988 | 0.986 |
| 31 | 440 | 81 | 1kpk−1kpl | 1kpk_B | 1.000 | 0.001 |
| 31 | 440 | 81 | 1kpk−1kpl | 1kpk_F | 1.000 | 0.001 |
| 32 | 133 | 80 | 1jac−1jot | 1ugw_C | 1.000 | 0.278 |
| 32 | 133 | 80 | 1jac−1jot | 1kuj_G | 1.000 | 0.353 |
| 32 | 133 | 80 | 1jac−1jot | 1uh1_G | 1.000 | 0.258 |
| 32 | 133 | 80 | 1jac−1jot | 1jac_C | 1.000 | 0.244 |
| 32 | 133 | 80 | 1jac−1jot | 1ugy_E | 1.000 | 0.300 |
| 32 | 133 | 80 | 1jac−1jot | 1ugy_C | 1.000 | 0.295 |
| 32 | 133 | 80 | 1jac−1jot | 1ws5_C | 1.000 | 0.286 |
| 32 | 133 | 80 | 1jac−1jot | 1kuj_A | 1.000 | 0.325 |
| 32 | 133 | 80 | 1jac−1jot | 1ws4_C | 1.000 | 0.279 |
| 32 | 133 | 80 | 1jac−1jot | 1uh0_G | 1.000 | 0.267 |
| 32 | 133 | 80 | 1jac−1jot | 1pxd_A | 1.000 | 0.302 |
| 32 | 133 | 80 | 1jac−1jot | 1ku8_G | 1.000 | 0.384 |
| 32 | 133 | 80 | 1jac−1jot | 1m26_G | 1.000 | 0.337 |
| 32 | 133 | 80 | 1jac−1jot | 1ugw_E | 1.000 | 0.270 |
| 32 | 133 | 80 | 1jac−1jot | 1jac_G | 1.000 | 0.291 |
| 32 | 133 | 80 | 1jac−1jot | 1ws5_E | 1.000 | 0.299 |
| 32 | 133 | 80 | 1jac−1jot | 1kuj_C | 1.000 | 0.408 |
| 32 | 133 | 80 | 1jac−1jot | 1ws4_E | 1.000 | 0.283 |
| 32 | 133 | 80 | 1jac−1jot | 1uh0_C | 1.000 | 0.267 |
| 32 | 133 | 80 | 1jac−1jot | 1ku8_C | 1.000 | 0.468 |
| 32 | 133 | 80 | 1jac−1jot | 1m26_A | 1.000 | 0.322 |
| 32 | 133 | 80 | 1jac−1jot | 1ku8_A | 1.000 | 0.354 |
| 32 | 133 | 80 | 1jac−1jot | 1m26_E | 1.000 | 0.379 |
| 32 | 133 | 80 | 1jac−1jot | 1uh1_A | 1.000 | 0.259 |
| 32 | 133 | 80 | 1jac−1jot | 1uh0_A | 1.000 | 0.270 |
| 32 | 133 | 80 | 1jac−1jot | 1ku8_E | 1.000 | 0.360 |
| 32 | 133 | 80 | 1jac−1jot | 1kuj_E | 1.000 | 0.376 |
| 32 | 133 | 80 | 1jac−1jot | 1uh1_C | 1.000 | 0.255 |
| 32 | 133 | 80 | 1jac−1jot | 1uh0_E | 1.000 | 0.265 |
| 32 | 133 | 80 | 1jac−1jot | 1m26_C | 1.000 | 0.322 |
| 32 | 133 | 80 | 1jac−1jot | 1uh1_E | 1.000 | 0.253 |
| 32 | 133 | 80 | 1jac−1jot | 1jac_E | 1.000 | 0.213 |
| 33 | 353 | 79 | 1ksa−4bcl | 1ksa_B | 1.000 | 0.001 |
| 34 | 312 | 79 | 1ezr−2mas | 1ezr_D | 1.000 | 0.049 |
| 34 | 312 | 79 | 2mas−1ezr | 2mas_C | 1.000 | 0.038 |
| 34 | 312 | 79 | 1ezr−2mas | 1ezr_B | 1.000 | 0.042 |
| 34 | 312 | 79 | 2mas−1ezr | 2mas_B | 1.000 | 0.041 |
| 34 | 312 | 79 | 2mas−1ezr | 2mas_D | 1.000 | 0.034 |
| 34 | 312 | 79 | 1ezr−2mas | 1ezr_C | 1.000 | 0.041 |
| 40 | 198 | 77 | 1kfu−1kxr | 1kfx_L | 0.968 | 0.718 |
| 40 | 198 | 77 | 1kxr−1kfu | 1kxr_B | 1.000 | 0.185 |
| 42 | 214 | 77 | 2ayh−1gbg | 1byh_A | 1.000 | 0.328 |
| 42 | 214 | 77 | 2ayh−1gbg | 1glh_A | 1.000 | 0.630 |

n: internal numbering; length of alignment; % sequence ID (cf. Homstrad); refA−refB: reference alignment; altA: alternative for struc. A; SP: sum−of−pairs score of alt. alignment w/r reference

| n | length | %ID | refA-refB | altA | SP | RMSD |
|---|---|---|---|---|---|---|
| 44 | 76 | 76 | 1fx7-2dtr | 1fx7_D | 1.000 | 0.435 |
| 44 | 76 | 76 | 1fx7-2dtr | 1u8r_G | 1.000 | 0.384 |
| 44 | 76 | 76 | 1fx7-2dtr | 1u8r_J | 1.000 | 0.385 |
| 44 | 76 | 76 | 1fx7-2dtr | 1u8r_B | 1.000 | 0.391 |
| 44 | 76 | 76 | 1fx7-2dtr | 1u8r_C | 1.000 | 0.390 |
| 44 | 76 | 76 | 1fx7-2dtr | 1u8r_H | 1.000 | 0.386 |
| 44 | 76 | 76 | 1fx7-2dtr | 1fx7_B | 1.000 | 0.400 |
| 44 | 76 | 76 | 1fx7-2dtr | 1u8r_A | 1.000 | 0.396 |
| 44 | 76 | 76 | 1fx7-2dtr | 1u8r_D | 1.000 | 0.392 |
| 44 | 76 | 76 | 2dtr-1fx7 | 1bi2_B | 1.000 | 0.453 |
| 44 | 76 | 76 | 1fx7-2dtr | 1fx7_C | 1.000 | 0.407 |
| 44 | 76 | 76 | 2dtr-1fx7 | 1bi2_A | 1.000 | 0.418 |
| 44 | 76 | 76 | 1fx7-2dtr | 1u8r_I | 1.000 | 0.396 |
| 45 | 160 | 76 | 1rho-1ds6 | 1rho_B | 0.958 | 3.020 |
| 46 | 60 | 76 | 1otf-1bjp | 1otf_E | 1.000 | 0.266 |
| 46 | 60 | 76 | 1otf-1bjp | 1otf_B | 1.000 | 0.223 |
| 46 | 60 | 76 | 1bjp-1otf | 4ota_P | 1.000 | 0.828 |
| 46 | 60 | 76 | 1bjp-1otf | 1bjp_E | 1.000 | 0.141 |
| 46 | 60 | 76 | 1bjp-1otf | 4ota_M | 1.000 | 0.599 |
| 46 | 60 | 76 | 1otf-1bjp | 1otf_C | 1.000 | 0.290 |
| 46 | 60 | 76 | 1bjp-1otf | 4ota_Q | 1.000 | 0.813 |
| 46 | 60 | 76 | 1otf-1bjp | 1otf_F | 1.000 | 0.180 |
| 46 | 60 | 76 | 1bjp-1otf | 1bjp_B | 1.000 | 0.139 |
| 46 | 60 | 76 | 1otf-1bjp | 1otf_D | 1.000 | 0.305 |
| 48 | 65 | 74 | 1g72-1h4i | 2ad6_D | 1.000 | 0.220 |
| 48 | 65 | 74 | 1g72-1h4i | 4aah_B | 1.000 | 0.292 |
| 48 | 65 | 74 | 1g72-1h4i | 2ad7_D | 1.000 | 0.229 |
| 48 | 65 | 74 | 1g72-1h4i | 2ad8_D | 1.000 | 0.212 |
| 48 | 65 | 74 | 1h4i-1g72 | 1h4i_D | 1.000 | 0.020 |
| 48 | 65 | 74 | 1g72-1h4i | 2ad6_B | 1.000 | 0.306 |
| 48 | 65 | 74 | 1g72-1h4i | 2ad7_B | 1.000 | 0.308 |
| 48 | 65 | 74 | 1g72-1h4i | 4aah_D | 1.000 | 0.248 |
| 48 | 65 | 74 | 1g72-1h4i | 2ad8_B | 1.000 | 0.227 |
| 48 | 65 | 74 | 1g72-1h4i | 1g72_D | 1.000 | 0.160 |
| 51 | 100 | 74 | 2kau-1ubp | 1a5m_A | 1.000 | 0.071 |
| 51 | 100 | 74 | 2kau-1ubp | 1krb_A | 1.000 | 0.099 |
| 51 | 100 | 74 | 2kau-1ubp | 1fwj_A | 1.000 | 0.065 |
| 51 | 100 | 74 | 2kau-1ubp | 1fwd_A | 1.000 | 0.068 |
| 51 | 100 | 74 | 2kau-1ubp | 1fwe_A | 1.000 | 0.113 |
| 51 | 100 | 74 | 2kau-1ubp | 1a5k_A | 1.000 | 0.088 |
| 51 | 100 | 74 | 2kau-1ubp | 1a5n_A | 1.000 | 0.097 |
| 51 | 100 | 74 | 2kau-1ubp | 1fwh_A | 1.000 | 0.084 |
| 51 | 100 | 74 | 2kau-1ubp | 1kra_A | 1.000 | 0.119 |
| 51 | 100 | 74 | 2kau-1ubp | 1fwb_A | 1.000 | 0.069 |
| 51 | 100 | 74 | 2kau-1ubp | 1krc_A | 1.000 | 0.092 |
| 51 | 100 | 74 | 2kau-1ubp | 1a5l_A | 1.000 | 0.147 |
| 51 | 100 | 74 | 2kau-1ubp | 1fwf_A | 1.000 | 0.076 |
| 51 | 100 | 74 | 2kau-1ubp | 1fwa_A | 1.000 | 0.067 |
| 51 | 100 | 74 | 2kau-1ubp | 1a5o_A | 1.000 | 0.106 |
| 51 | 100 | 74 | 2kau-1ubp | 1fwi_A | 1.000 | 0.079 |
| 51 | 100 | 74 | 2kau-1ubp | 1fwc_A | 1.000 | 0.065 |
| 51 | 100 | 74 | 2kau-1ubp | 1fwg_A | 1.000 | 0.070 |
| 55 | 282 | 73 | 1f0n-1dqz | 1f0p_A | 0.982 | 0.225 |
| 58 | 140 | 72 | 1aoh-1anu | 1aoh_B | 1.000 | 0.335 |
| 59 | 379 | 72 | 1bcc-1bgy | 2bcc_C | 0.987 | 0.660 |
| 59 | 379 | 72 | 1bgy-1bcc | 1be3_C | 1.000 | 0.241 |
| 59 | 379 | 72 | 1bgy-1bcc | 1bgy_O | 1.000 | 0.082 |
| 59 | 379 | 72 | 1bcc-1bgy | 3bcc_C | 0.987 | 0.761 |
| 60 | 213 | 71 | 1k6m-1bif | 1k6m_B | 1.000 | 0.344 |
| 61 | 250 | 71 | 1cfm-1hcz | 1cfm_C | 1.000 | 0.680 |
| 61 | 250 | 71 | 1cfm-1hcz | 1ewh_A | 1.000 | 0.450 |
| 61 | 250 | 71 | 1cfm-1hcz | 1ewh_B | 1.000 | 0.974 |
| 61 | 250 | 71 | 1cfm-1hcz | 1ewh_C | 1.000 | 0.815 |
| 61 | 250 | 71 | 1cfm-1hcz | 1cfm_B | 1.000 | 0.572 |
| 62 | 553 | 71 | 1eex-1iwp | 1uc4_L | 1.000 | 0.392 |
| 62 | 553 | 71 | 1eex-1iwp | 1egv_A | 1.000 | 0.038 |
| 62 | 553 | 71 | 1iwp-1eex | 1mmf_L | 1.000 | 0.481 |
| 62 | 553 | 71 | 1eex-1iwp | 1uc5_L | 1.000 | 0.310 |
| 62 | 553 | 71 | 1eex-1iwp | 1uc4_A | 1.000 | 0.207 |
| 62 | 553 | 71 | 1iwp-1eex | 1iwp_L | 1.000 | 0.229 |
| 62 | 553 | 71 | 1eex-1iwp | 1iwb_A | 1.000 | 0.275 |
| 62 | 553 | 71 | 1eex-1iwp | 1dio_L | 1.000 | 0.416 |
| 62 | 553 | 71 | 1eex-1iwp | 1egm_A | 1.000 | 0.183 |
| 62 | 553 | 71 | 1eex-1iwp | 1iwb_L | 1.000 | 0.345 |
| 62 | 553 | 71 | 1eex-1iwp | 1egv_L | 1.000 | 0.291 |
| 62 | 553 | 71 | 1eex-1iwp | 1egm_L | 1.000 | 0.379 |
| 62 | 553 | 71 | 1eex-1iwp | 1uc5_A | 1.000 | 0.282 |
| 62 | 553 | 71 | 1eex-1iwp | 1eex_L | 1.000 | 0.285 |
| 62 | 553 | 71 | 1eex-1iwp | 1dio_A | 1.000 | 0.345 |
| 65 | 105 | 71 | 1i50-1qkl | 1y1w_F | 0.952 | 0.579 |
| 65 | 105 | 71 | 1i50-1qkl | 2r92_F | 0.976 | 0.573 |
| 65 | 105 | 71 | 1i50-1qkl | 1nik_F | 0.952 | 0.324 |
| 65 | 105 | 71 | 1i50-1qkl | 2nvq_F | 0.867 | 0.362 |
| 65 | 105 | 71 | 1i50-1qkl | 1wcm_F | 0.952 | 0.548 |
| 65 | 105 | 71 | 1i50-1qkl | 1i3q_F | 0.976 | 0.280 |
| 65 | 105 | 71 | 1i50-1qkl | 1r9s_F | 0.952 | 0.324 |
| 65 | 105 | 71 | 1i50-1qkl | 1y1v_F | 0.952 | 0.642 |
| 65 | 105 | 71 | 1i50-1qkl | 2nvy_F | 0.952 | 0.383 |
| 65 | 105 | 71 | 1i50-1qkl | 2ja6_F | 0.940 | 0.587 |
| 65 | 105 | 71 | 1i50-1qkl | 2ja7_R | 0.976 | 0.577 |
| 65 | 105 | 71 | 1i50-1qkl | 2yu9_F | 0.940 | 0.480 |
| 65 | 105 | 71 | 1i50-1qkl | 1y77_F | 0.952 | 0.548 |
| 65 | 105 | 71 | 1i50-1qkl | 2e2i_F | 0.988 | 0.445 |
| 65 | 105 | 71 | 1i50-1qkl | 1r5u_F | 0.952 | 0.324 |
| 65 | 105 | 71 | 1i50-1qkl | 2ja7_F | 0.952 | 0.585 |
| 65 | 105 | 71 | 1i50-1qkl | 2b8k_F | 0.952 | 0.548 |
| 65 | 105 | 71 | 1i50-1qkl | 2r7z_F | 0.976 | 0.607 |
| 65 | 105 | 71 | 1i50-1qkl | 1i6h_F | 0.952 | 0.324 |
| 65 | 105 | 71 | 1i50-1qkl | 2nvt_F | 0.964 | 0.499 |
| 65 | 105 | 71 | 1i50-1qkl | 2b63_F | 0.952 | 0.541 |
| 65 | 105 | 71 | 1i50-1qkl | 1sfo_F | 0.976 | 0.521 |
| 65 | 105 | 71 | 1i50-1qkl | 2ja8_F | 0.952 | 0.582 |
| 65 | 105 | 71 | 1i50-1qkl | 1k83_F | 1.000 | 0.138 |
| 65 | 105 | 71 | 1i50-1qkl | 2nvx_F | 0.892 | 0.521 |
| 65 | 105 | 71 | 1i50-1qkl | 2r93_F | 0.952 | 0.558 |
| 65 | 105 | 71 | 1i50-1qkl | 1twf_F | 0.964 | 0.245 |
| 65 | 105 | 71 | 1i50-1qkl | 2ja5_F | 0.952 | 0.582 |
| 65 | 105 | 71 | 1i50-1qkl | 1r9t_F | 0.867 | 0.586 |
| 66 | 69 | 71 | 1dd3-1ctf | 1dd4_B | 1.000 | 0.399 |
| 66 | 69 | 71 | 1dd3-1ctf | 1yl3_J | 1.000 | 0.770 |
| 66 | 69 | 71 | 1dd3-1ctf | 1yl3_I | 1.000 | 0.768 |
| 66 | 69 | 71 | 1dd3-1ctf | 1dd3_B | 1.000 | 0.339 |
| 66 | 69 | 71 | 1dd3-1ctf | 1dd4_A | 1.000 | 0.396 |
| 67 | 48 | 71 | 1iva-1oma | 1oav_A | 1 | 7.001 |
| 68 | 210 | 70 | 1yer-1ah6 | 1yet_A | 1.000 | 1.584 |
| 68 | 210 | 70 | 1ah6-1yer | 1ah8_B | 0.995 | 0.999 |
| 68 | 210 | 70 | 1yer-1ah6 | 1byq_A | 0.976 | 1.758 |
| 68 | 210 | 70 | 1ah6-1yer | 1ah8_A | 0.995 | 0.913 |
| 68 | 210 | 70 | 1yer-1ah6 | 1yes_A | 1.000 | 1.577 |
| 70 | 277 | 70 | 2nip-1cp2 | 1m1y_P | 0.996 | 0.912 |
| 70 | 277 | 70 | 2nip-1cp2 | 2nip_B | 0.996 | 0.726 |
| 70 | 277 | 70 | 2nip-1cp2 | 1m1y_F | 0.996 | 0.914 |
| 70 | 277 | 70 | 2nip-1cp2 | 1m1y_G | 0.996 | 0.914 |
| 70 | 277 | 70 | 2nip-1cp2 | 1fp6_B | 1.000 | 0.905 |
| 70 | 277 | 70 | 2nip-1cp2 | 1m1y_E | 0.996 | 0.912 |
| 70 | 277 | 70 | 1cp2-2nip | 1cp2_B | 1.000 | 0.742 |
| 70 | 277 | 70 | 2nip-1cp2 | 1nip_B | 1.000 | 1.322 |
| 70 | 277 | 70 | 2nip-1cp2 | 1fp6_A | 0.996 | 0.962 |
| 70 | 277 | 70 | 2nip-1cp2 | 1g1m_A | 1.000 | 0.923 |
| 70 | 277 | 70 | 2nip-1cp2 | 2afh_F | 0.993 | 0.933 |
| 70 | 277 | 70 | 2nip-1cp2 | 1g5p_A | 1.000 | 0.034 |
| 70 | 277 | 70 | 2nip-1cp2 | 1g5p_B | 0.996 | 0.718 |
| 70 | 277 | 70 | 2nip-1cp2 | 1m1y_N | 0.996 | 0.918 |
| 70 | 277 | 70 | 2nip-1cp2 | 1m1y_M | 0.996 | 0.918 |
| 70 | 277 | 70 | 2nip-1cp2 | 2afh_E | 0.996 | 1.052 |
| 70 | 277 | 70 | 2nip-1cp2 | 1g1m_B | 1.000 | 0.957 |
| 70 | 277 | 70 | 2nip-1cp2 | 1m1y_H | 0.996 | 0.916 |
| 70 | 277 | 70 | 2nip-1cp2 | 1m1y_O | 0.996 | 0.914 |
| 71 | 125 | 69 | 2scu-1eud | 2scu_D | 1.000 | 0.258 |
| 71 | 125 | 69 | 2scu-1eud | 1jkj_A | 1.000 | 0.273 |
| 71 | 125 | 69 | 2scu-1eud | 1jll_D | 1.000 | 0.258 |
| 71 | 125 | 69 | 1eud-2scu | 1euc_A | 1.000 | 0.110 |
| 71 | 125 | 69 | 2scu-1eud | 1jkj_D | 1.000 | 0.278 |
| 71 | 125 | 69 | 2scu-1eud | 1scu_D | 1.000 | 0.337 |
| 71 | 125 | 69 | 2scu-1eud | 1jll_A | 1.000 | 0.252 |
| 71 | 125 | 69 | 2scu-1eud | 1scu_A | 1.000 | 0.289 |
| 76 | 150 | 68 | 1wyk-1vcp | 1wyk_D | 1.000 | 0.074 |
| 76 | 150 | 68 | 1vcp-1wyk | 1vcq_B | 1.000 | 0.445 |
| 76 | 150 | 68 | 1vcp-1wyk | 1dyl_B | 1.000 | 0.437 |
| 76 | 150 | 68 | 1wyk-1vcp | 1wyk_B | 1.000 | 0.579 |
| 76 | 150 | 68 | 1vcp-1wyk | 1vcp_B | 1.000 | 0.178 |
| 76 | 150 | 68 | 1vcp-1wyk | 1dyl_C | 1.000 | 0.437 |
| 76 | 150 | 68 | 1vcp-1wyk | 1dyl_D | 1.000 | 0.437 |
| 76 | 150 | 68 | 1vcp-1wyk | 1vcq_A | 1.000 | 0.437 |
| 76 | 150 | 68 | 1wyk-1vcp | 1wyk_C | 1.000 | 0.611 |
| 76 | 150 | 68 | 1vcp-1wyk | 1dyl_A | 1.000 | 0.437 |
| 76 | 150 | 68 | 1vcp-1wyk | 1vcp_C | 0.993 | 0.312 |
| 78 | 126 | 68 | 2lis-3lyn | 1lis_A | 1.000 | 0.434 |
| 78 | 126 | 68 | 3lyn-2lis | 3lyn_B | 1.000 | 1.001 |
| 80 | 142 | 68 | 1vlt-2asr | 1vls_A | 0.885 | 2.350 |
| 80 | 142 | 68 | 1vlt-2asr | 1was_A | 0.878 | 2.722 |
| 80 | 142 | 68 | 1vlt-2asr | 1wat_A | 0.928 | 2.067 |
| 80 | 142 | 68 | 1vlt-2asr | 1vlt_B | 0.892 | 3.136 |
| 80 | 142 | 68 | 1vlt-2asr | 1wat_B | 0.906 | 3.085 |
| 81 | 118 | 68 | 3pmg-1kfq | 1c4g_B | 1.000 | 0.454 |
| 81 | 118 | 68 | 3pmg-1kfq | 1jdy_A | 1.000 | 0.237 |
| 81 | 118 | 68 | 3pmg-1kfq | 1lxt_B | 1.000 | 0.437 |
| 81 | 118 | 68 | 1kfq-3pmg | 1kfi_A | 1.000 | 0.484 |
| 81 | 118 | 68 | 1kfq-3pmg | 1kfi_B | 1.000 | 0.383 |
| 81 | 118 | 68 | 3pmg-1kfq | 1c47_A | 1.000 | 0.338 |
| 81 | 118 | 68 | 3pmg-1kfq | 1jdy_B | 1.000 | 0.391 |
| 81 | 118 | 68 | 3pmg-1kfq | 1lxt_A | 1.000 | 0.308 |
| 81 | 118 | 68 | 1kfq-3pmg | 1kfq_B | 1.000 | 0.229 |
| 81 | 118 | 68 | 3pmg-1kfq | 1c47_B | 1.000 | 0.388 |
| 81 | 118 | 68 | 3pmg-1kfq | 1vkl_A | 1.000 | 0.218 |
| 81 | 118 | 68 | 3pmg-1kfq | 1vkl_B | 1.000 | 0.381 |
| 81 | 118 | 68 | 3pmg-1kfq | 3pmg_B | 1.000 | 0.408 |
| 81 | 118 | 68 | 3pmg-1kfq | 1c4g_A | 1.000 | 0.489 |
| 84 | 337 | 67 | 1kxr-1kfu | 1kxr_B | 0.965 | 0.599 |
| 87 | 102 | 67 | 1m42-1lyq | 2c9p_A | 0.990 | 2.576 |
| 87 | 102 | 67 | 1m42-1lyq | 2c9p_B | 0.990 | 2.638 |
| 87 | 102 | 67 | 1lyq-1m42 | 1lyq_A | 1.000 | 0.030 |
| 87 | 102 | 67 | 1m42-1lyq | 2c9p_C | 0.990 | 2.598 |
| 87 | 102 | 67 | 1m42-1lyq | 2c9q_A | 0.990 | 2.746 |
| 87 | 102 | 67 | 1m42-1lyq | 1nm4_A | 0.990 | 2.004 |
| 88 | 544 | 67 | 1nir-1aoq | 1n90_B | 1.000 | 0.212 |
| 88 | 544 | 67 | 1aoq-1nir | 1aoq_B | 1.000 | 0.415 |
| 88 | 544 | 67 | 1nir-1aoq | 1gjq_A | 1.000 | 0.572 |
| 88 | 544 | 67 | 1nir-1aoq | 1n15_B | 1.000 | 0.197 |
| 88 | 544 | 67 | 1aoq-1nir | 1aom_B | 1.000 | 0.440 |
| 88 | 544 | 67 | 1nir-1aoq | 1n50_A | 1.000 | 0.171 |
| 88 | 544 | 67 | 1nir-1aoq | 1n50_B | 1.000 | 0.203 |
| 88 | 544 | 67 | 1nir-1aoq | 1n90_A | 1.000 | 0.170 |
| 88 | 544 | 67 | 1nir-1aoq | 1n15_A | 1.000 | 0.153 |
| 88 | 544 | 67 | 1nir-1aoq | 1gjq_B | 1.000 | 0.570 |
| 88 | 544 | 67 | 1aoq-1nir | 1qks_B | 1.000 | 0.414 |
| 88 | 544 | 67 | 1nir-1aoq | 1nir_B | 1.000 | 0.172 |
| 88 | 544 | 67 | 1aoq-1nir | 1qks_A | 1.000 | 0.184 |
| 88 | 544 | 67 | 1nir-1aoq | 1nno_A | 1.000 | 0.576 |
| 88 | 544 | 67 | 1nir-1aoq | 1nno_B | 1.000 | 0.572 |
| 91 | 163 | 67 | 1f1m-1g5z | 1f1m_D | 0.994 | 0.462 |
| 91 | 163 | 67 | 1f1m-1g5z | 1f1m_B | 0.994 | 0.356 |
| 91 | 163 | 67 | 1f1m-1g5z | 1f1m_C | 0.994 | 0.497 |
| 94 | 548 | 67 | 1gnl-1gnt | 1upx_A | 1.000 | 0.130 |
| 94 | 548 | 67 | 1gnt-1gnl | 1oa1_A | 0.998 | 0.305 |
| 94 | 548 | 67 | 1gnl-1gnt | 1gnl_A | 1.000 | 0.001 |
| 94 | 548 | 67 | 1gnl-1gnt | 1gnl_B | 0.998 | 0.415 |
| 94 | 548 | 67 | 1gnl-1gnt | 1oa0_B | 1.000 | 0.434 |
| 94 | 548 | 67 | 1gnl-1gnt | 1upx_B | 1.000 | 0.396 |
| 94 | 548 | 67 | 1gnl-1gnt | 1oa0_A | 1.000 | 0.188 |
| 96 | 132 | 66 | 1ayo-1edy | 1ayo_B | 0.984 | 0.671 |
| 100 | 212 | 66 | 1fxw-1wab | 1vyh_F | 1.000 | 0.390 |
| 100 | 212 | 66 | 1fxw-1wab | 1vyh_J | 1.000 | 0.384 |

n: internal numbering; length of alignment; % sequence ID (cf. Homstrad); refA-refB: reference alignment; altA: alternative for struc. A; SP: sum-of-pairs score of alt. alignment w/r reference

| n | length | %ID | refA-refB | altA | SP | RMSD |
|---|---|---|---|---|---|---|
| 100 | 212 | 66 | 1fxw-1wab | 1vyh_E | 1.000 | 0.383 |
| 100 | 212 | 66 | 1fxw-1wab | 1vyh_Q | 1.000 | 0.382 |
| 100 | 212 | 66 | 1fxw-1wab | 1vyh_N | 1.000 | 0.382 |
| 100 | 212 | 66 | 1fxw-1wab | 1vyh_A | 1.000 | 0.380 |
| 100 | 212 | 66 | 1fxw-1wab | 1vyh_R | 1.000 | 0.381 |
| 100 | 212 | 66 | 1fxw-1wab | 1vyh_I | 1.000 | 0.380 |
| 100 | 212 | 66 | 1fxw-1wab | 1vyh_M | 1.000 | 0.382 |
| 100 | 212 | 66 | 1fxw-1wab | 1vyh_B | 1.000 | 0.382 |
| 102 | 316 | 65 | 4pah-1toh | 5pah_A | 1.000 | 0.167 |
| 102 | 316 | 65 | 4pah-1toh | 6pah_A | 1.000 | 0.186 |
| 102 | 316 | 65 | 4pah-1toh | 1pah_A | 1.000 | 0.225 |
| 102 | 316 | 65 | 4pah-1toh | 3pah_A | 1.000 | 0.072 |
| 108 | 434 | 64 | 1one-1pdz | 1one_B | 1.000 | 0.214 |
| 108 | 434 | 64 | 1one-1pdz | 2al1_A | 1.000 | 0.365 |
| 108 | 434 | 64 | 1one-1pdz | 2one_B | 0.986 | 0.998 |
| 108 | 434 | 64 | 1one-1pdz | 1ebg_A | 1.000 | 0.274 |
| 108 | 434 | 64 | 1one-1pdz | 1ebh_A | 0.988 | 1.633 |
| 108 | 434 | 64 | 1one-1pdz | 2one_A | 1.000 | 0.459 |
| 108 | 434 | 64 | 1pdz-1one | 1pdy_A | 1.000 | 0.658 |
| 108 | 434 | 64 | 1one-1pdz | 1ebg_B | 1.000 | 0.331 |
| 108 | 434 | 64 | 1one-1pdz | 1ebh_B | 0.988 | 1.644 |
| 110 | 567 | 64 | 1ubp-2kau | 3ubp_C | 1.000 | 0.795 |
| 110 | 567 | 64 | 1ubp-2kau | 1s3t_C | 1.000 | 0.152 |
| 110 | 567 | 64 | 2kau-1ubp | 1kra_C | 1.000 | 0.257 |
| 110 | 567 | 64 | 1ubp-2kau | 4ubp_C | 1.000 | 0.193 |
| 110 | 567 | 64 | 1ubp-2kau | 2ubp_C | 0.991 | 0.186 |
| 114 | 103 | 63 | 2pii-2gnk | 1pil_A | 1.000 | 0.319 |
| 116 | 140 | 63 | 1k5d-1rrp | 1k5d_E | 0.948 | 0.117 |
| 116 | 140 | 63 | 1k5d-1rrp | 1k5d_H | 1.000 | 0.089 |
| 116 | 140 | 63 | 1k5d-1rrp | 1k5g_E | 1.000 | 0.393 |
| 116 | 140 | 63 | 1k5d-1rrp | 1k5d_K | 0.948 | 0.101 |
| 116 | 140 | 63 | 1k5d-1rrp | 1k5g_K | 1.000 | 0.387 |
| 116 | 140 | 63 | 1rrp-1k5d | 1rrp_D | 1.000 | 0.598 |
| 116 | 140 | 63 | 1k5d-1rrp | 1k5g_B | 1.000 | 0.405 |
| 116 | 140 | 63 | 1k5d-1rrp | 1k5g_H | 1.000 | 0.391 |
| 118 | 148 | 63 | 1qm4-1mxb | 1o93_B | 1.000 | 0.179 |
| 118 | 148 | 63 | 1qm4-1mxb | 1o9t_A | 1.000 | 0.144 |
| 118 | 148 | 63 | 1qm4-1mxb | 1qm4_B | 1.000 | 0.137 |
| 118 | 148 | 63 | 1mxb-1qm4 | 1p7l_A | 1.000 | 0.737 |
| 118 | 148 | 63 | 1qm4-1mxb | 1o90_A | 1.000 | 0.145 |
| 118 | 148 | 63 | 1mxb-1qm4 | 1xrc_A | 1.000 | 0.311 |
| 118 | 148 | 63 | 1qm4-1mxb | 1o9t_B | 1.000 | 0.182 |
| 118 | 148 | 63 | 1mxb-1qm4 | 1p7l_C | 0.993 | 0.735 |
| 118 | 148 | 63 | 1mxb-1qm4 | 1p7l_D | 1.000 | 0.734 |
| 118 | 148 | 63 | 1mxb-1qm4 | 1rg9_A | 1.000 | 0.735 |
| 118 | 148 | 63 | 1mxb-1qm4 | 1xra_A | 1.000 | 0.330 |
| 118 | 148 | 63 | 1qm4-1mxb | 1o92_A | 1.000 | 0.148 |
| 118 | 148 | 63 | 1mxb-1qm4 | 1mxc_A | 0.993 | 0.162 |
| 118 | 148 | 63 | 1mxb-1qm4 | 1fug_A | 0.993 | 0.893 |
| 118 | 148 | 63 | 1qm4-1mxb | 1o90_B | 1.000 | 0.183 |
| 118 | 148 | 63 | 1qm4-1mxb | 1o92_B | 1.000 | 0.182 |
| 118 | 148 | 63 | 1mxb-1qm4 | 1rg9_B | 1.000 | 0.735 |
| 118 | 148 | 63 | 1mxb-1qm4 | 1fug_B | 0.965 | 0.848 |
| 118 | 148 | 63 | 1qm4-1mxb | 1o93_A | 1.000 | 0.149 |
| 118 | 148 | 63 | 1mxb-1qm4 | 1p7l_B | 0.993 | 0.737 |
| 118 | 148 | 63 | 1mxb-1qm4 | 1mxa_A | 1.000 | 0.285 |
| 118 | 148 | 63 | 1mxb-1qm4 | 1rg9_D | 1.000 | 0.732 |
| 118 | 148 | 63 | 1mxb-1qm4 | 1rg9_C | 0.993 | 0.732 |
| 121 | 173 | 62 | 1cf1-1g4m | 1cf1_C | 1.000 | 0.098 |
| 121 | 173 | 62 | 1cf1-1g4m | 1cf1_D | 0.982 | 3.137 |
| 121 | 173 | 62 | 1g4m-1cf1 | 1g4m_B | 0.959 | 1.243 |
| 122 | 361 | 62 | 1clq-1noz | 1waf_A | 0.982 | 0.984 |
| 122 | 361 | 62 | 1clq-1noz | 1waf_B | 0.997 | 0.992 |
| 122 | 361 | 62 | 1noz-1clq | 1noy_B | 0.997 | 0.903 |
| 122 | 361 | 62 | 1clq-1noz | 1ih7_A | 0.982 | 0.901 |
| 122 | 361 | 62 | 1clq-1noz | 1waj_A | 0.997 | 0.978 |
| 122 | 361 | 62 | 1noz-1clq | 1noz_B | 0.997 | 0.922 |
| 126 | 68 | 62 | 1qa6-1mms | 1c04_C | 1.000 | 0.001 |
| 126 | 68 | 62 | 1mms-1qa6 | 1mms_B | 1.000 | 0.209 |
| 126 | 68 | 62 | 1qa6-1mms | 1qa6_B | 1.000 | 0.001 |
| 127 | 133 | 62 | 1an7-1sei | 1an7_B | 1.000 | 0.390 |
| 127 | 133 | 62 | 1sei-1an7 | 1sei_B | 1.000 | 0.308 |
| 131 | 196 | 61 | 1bgy-1ezv | 1ntz_D | 0.947 | 3.043 |
| 131 | 196 | 61 | 1bgy-1ezv | 1pp9_D | 0.947 | 2.988 |
| 131 | 196 | 61 | 1bgy-1ezv | 1l0l_D | 0.921 | 3.174 |
| 131 | 196 | 61 | 1bgy-1ezv | 2a06_D | 0.947 | 2.971 |
| 131 | 196 | 61 | 1bgy-1ezv | 2a06_Q | 0.947 | 2.985 |
| 131 | 196 | 61 | 1bgy-1ezv | 1pp9_Q | 0.947 | 2.992 |
| 131 | 196 | 61 | 1bgy-1ezv | 1sqv_D | 0.915 | 3.148 |
| 131 | 196 | 61 | 1bgy-1ezv | 1bgy_P | 1.000 | 0.040 |
| 131 | 196 | 61 | 1bgy-1ezv | 1sqb_D | 0.947 | 3.007 |
| 131 | 196 | 61 | 1bgy-1ezv | 1ppj_Q | 0.947 | 3.005 |
| 131 | 196 | 61 | 1bgy-1ezv | 1be3_D | 1.000 | 0.393 |
| 131 | 196 | 61 | 1bgy-1ezv | 1sqp_D | 0.926 | 3.027 |
| 131 | 196 | 61 | 1bgy-1ezv | 1ntm_D | 0.910 | 3.082 |
| 131 | 196 | 61 | 1bgy-1ezv | 1sqx_D | 0.947 | 3.044 |
| 131 | 196 | 61 | 1bgy-1ezv | 1l0n_D | 0.926 | 2.922 |
| 131 | 196 | 61 | 1bgy-1ezv | 1sqq_D | 0.947 | 3.068 |
| 131 | 196 | 61 | 1bgy-1ezv | 1nu1_D | 0.947 | 3.065 |
| 131 | 196 | 61 | 1bgy-1ezv | 2fyu_D | 0.947 | 3.068 |
| 131 | 196 | 61 | 1bgy-1ezv | 1ppj_D | 0.947 | 2.996 |
| 131 | 196 | 61 | 1bgy-1ezv | 1ntk_D | 0.947 | 3.024 |
| 132 | 181 | 61 | 1eex-1iwp | 1eex_E | 1.000 | 0.324 |
| 132 | 181 | 61 | 1eex-1iwp | 1egv_E | 1.000 | 0.334 |
| 132 | 181 | 61 | 1eex-1iwp | 1egm_B | 1.000 | 0.152 |
| 132 | 181 | 61 | 1eex-1iwp | 1uc4_B | 1.000 | 0.162 |
| 132 | 181 | 61 | 1iwp-1eex | 1iwp_E | 1.000 | 0.382 |
| 132 | 181 | 61 | 1eex-1iwp | 1dio_B | 1.000 | 0.284 |
| 132 | 181 | 61 | 1eex-1iwp | 1egv_B | 1.000 | 0.048 |
| 132 | 181 | 61 | 1eex-1iwp | 1uc5_B | 1.000 | 0.240 |
| 132 | 181 | 61 | 1eex-1iwp | 1dio_E | 1.000 | 0.437 |
| 132 | 181 | 61 | 1eex-1iwp | 1iwb_B | 1.000 | 0.236 |
| 134 | 635 | 61 | 1l1y-1fce | 1l1y_F | 0.998 | 0.245 |
| 134 | 635 | 61 | 1l1y-1fce | 1l2a_C | 0.998 | 0.230 |
| 134 | 635 | 61 | 1fce-1l1y | 1g9g_A | 0.997 | 0.111 |
| 134 | 635 | 61 | 1l1y-1fce | 1l1y_B | 0.998 | 0.273 |
| 134 | 635 | 61 | 1l1y-1fce | 1l1y_C | 0.998 | 0.175 |
| 134 | 635 | 61 | 1l1y-1fce | 1l1y_E | 0.998 | 0.271 |
| 134 | 635 | 61 | 1l1y-1fce | 1l2a_B | 0.998 | 0.334 |
| 134 | 635 | 61 | 1l1y-1fce | 1l2a_D | 1.000 | 0.258 |
| 134 | 635 | 61 | 1fce-1l1y | 1fae_A | 1.000 | 0.098 |
| 134 | 635 | 61 | 1fce-1l1y | 1fbo_A | 1.000 | 0.117 |
| 134 | 635 | 61 | 1l1y-1fce | 1l2a_E | 0.998 | 0.304 |
| 134 | 635 | 61 | 1l1y-1fce | 1l2a_A | 1.000 | 0.210 |
| 134 | 635 | 61 | 1l1y-1fce | 1l2a_F | 1.000 | 0.268 |
| 134 | 635 | 61 | 1l1y-1fce | 1l1y_D | 0.998 | 0.245 |
| 134 | 635 | 61 | 1fce-1l1y | 1f9o_A | 1.000 | 0.147 |
| 135 | 158 | 61 | 1kko-1kd0 | 1kkr_B | 1.000 | 0.777 |
| 135 | 158 | 61 | 1kd0-1kko | 1kd0_A | 1.000 | 0.006 |
| 135 | 158 | 61 | 1kko-1kd0 | 1kkr_A | 1.000 | 0.471 |
| 135 | 158 | 61 | 1kd0-1kko | 1kd0_B | 1.000 | 0.379 |
| 135 | 158 | 61 | 1kko-1kd0 | 1kko_B | 1.000 | 0.490 |
| 138 | 100 | 61 | 1qm4-1mxb | 1o92_B | 1.000 | 0.133 |
| 138 | 100 | 61 | 1mxb-1qm4 | 1p7l_B | 0.980 | 1.997 |
| 138 | 100 | 61 | 1mxb-1qm4 | 1p7l_C | 0.980 | 1.997 |
| 138 | 100 | 61 | 1qm4-1mxb | 1o93_B | 1.000 | 0.130 |
| 138 | 100 | 61 | 1mxb-1qm4 | 1fug_A | 1.000 | 0.697 |
| 138 | 100 | 61 | 1mxb-1qm4 | 1xrc_A | 1.000 | 0.276 |
| 138 | 100 | 61 | 1mxb-1qm4 | 1fug_B | 1.000 | 0.698 |
| 138 | 100 | 61 | 1mxb-1qm4 | 1mxa_A | 1.000 | 0.219 |
| 138 | 100 | 61 | 1qm4-1mxb | 1o90_A | 1.000 | 0.134 |
| 138 | 100 | 61 | 1mxb-1qm4 | 1p7l_D | 0.980 | 1.998 |
| 138 | 100 | 61 | 1mxb-1qm4 | 1p7l_A | 0.980 | 1.997 |
| 138 | 100 | 61 | 1qm4-1mxb | 1o90_B | 1.000 | 0.133 |
| 138 | 100 | 61 | 1mxb-1qm4 | 1rg9_C | 0.980 | 1.998 |
| 138 | 100 | 61 | 1qm4-1mxb | 1qm4_B | 1.000 | 0.034 |
| 138 | 100 | 61 | 1mxb-1qm4 | 1xra_A | 1.000 | 0.253 |
| 138 | 100 | 61 | 1qm4-1mxb | 1o92_A | 1.000 | 0.134 |
| 138 | 100 | 61 | 1mxb-1qm4 | 1rg9_B | 0.980 | 1.997 |
| 138 | 100 | 61 | 1qm4-1mxb | 1o9t_A | 1.000 | 0.133 |
| 138 | 100 | 61 | 1mxb-1qm4 | 1rg9_A | 0.980 | 1.997 |
| 138 | 100 | 61 | 1mxb-1qm4 | 1mxc_A | 1.000 | 0.121 |
| 138 | 100 | 61 | 1qm4-1mxb | 1o93_A | 1.000 | 0.132 |
| 138 | 100 | 61 | 1mxb-1qm4 | 1rg9_D | 0.980 | 1.997 |
| 138 | 100 | 61 | 1qm4-1mxb | 1o9t_B | 1.000 | 0.134 |
| 142 | 123 | 60 | 1d9c-1fyh | 1d9g_A | 0.967 | 1.141 |
| 142 | 123 | 60 | 1fyh-1d9c | 1fyh_D | 1.000 | 0.552 |
| 143 | 271 | 60 | 1qr6-1llq | 1efk_D | 1.000 | 0.935 |
| 143 | 271 | 60 | 1llq-1qr6 | 1o0s_A | 0.980 | 0.178 |
| 143 | 271 | 60 | 1qr6-1llq | 1efk_A | 1.000 | 0.945 |
| 143 | 271 | 60 | 1qr6-1llq | 1efk_B | 0.980 | 0.927 |
| 143 | 271 | 60 | 1qr6-1llq | 1efl_A | 1.000 | 0.844 |
| 143 | 271 | 60 | 1qr6-1llq | 1efl_B | 0.980 | 0.849 |
| 143 | 271 | 60 | 1qr6-1llq | 1efl_D | 1.000 | 0.844 |
| 143 | 271 | 60 | 1qr6-1llq | 1efk_C | 0.980 | 0.924 |
| 143 | 271 | 60 | 1qr6-1llq | 1pjl_A | 0.980 | 0.754 |
| 143 | 271 | 60 | 1llq-1qr6 | 1llq_B | 0.980 | 0.181 |
| 143 | 271 | 60 | 1qr6-1llq | 1efl_C | 1.000 | 0.839 |
| 143 | 271 | 60 | 1llq-1qr6 | 1o0s_B | 0.980 | 0.316 |
| 144 | 383 | 60 | 1mhy-1mty | 1mhz_B | 1.000 | 0.230 |
| 144 | 383 | 60 | 1mty-1mhy | 1mty_C | 1.000 | 0.280 |
| 153 | 249 | 59 | 1efp-1efv | 1efp_D | 1.000 | 0.447 |
| 154 | 151 | 59 | 1flk-1f3v | 1fll_B | 0.973 | 0.856 |
| 154 | 151 | 59 | 1flk-1f3v | 1flk_B | 0.973 | 0.714 |
| 154 | 151 | 59 | 1flk-1f3v | 1fll_A | 1.000 | 1.133 |
| 156 | 159 | 59 | 1bv1-1e09 | 1fsk_G | 1.000 | 0.652 |
| 156 | 159 | 59 | 1bv1-1e09 | 1fsk_J | 1.000 | 0.652 |
| 156 | 159 | 59 | 1bv1-1e09 | 1bv1_A | 1.000 | 0.020 |
| 156 | 159 | 59 | 1bv1-1e09 | 1fsk_A | 1.000 | 0.651 |
| 156 | 159 | 59 | 1bv1-1e09 | 1fsk_D | 1.000 | 0.651 |
| 157 | 298 | 59 | 1nfk-1a3q | 1nfk_B | 0.979 | 1.465 |
| 157 | 298 | 59 | 1a3q-1nfk | 1a3q_B | 0.982 | 1.397 |
| 159 | 319 | 59 | 1onr-1f05 | 1onr_B | 1.000 | 0.011 |
| 159 | 319 | 59 | 1f05-1onr | 1f05_B | 0.975 | 0.517 |
| 162 | 243 | 58 | 1bgy-1ezv | 1sqb_D | 0.958 | 2.735 |
| 162 | 243 | 58 | 1bgy-1ezv | 2a06_Q | 0.958 | 2.715 |
| 162 | 243 | 58 | 1bgy-1ezv | 1ppj_Q | 0.958 | 2.737 |
| 162 | 243 | 58 | 1bgy-1ezv | 1sqq_D | 0.958 | 2.789 |
| 162 | 243 | 58 | 1bgy-1ezv | 1ppj_D | 0.958 | 2.737 |
| 162 | 243 | 58 | 1bgy-1ezv | 1sqv_D | 0.932 | 2.860 |
| 162 | 243 | 58 | 1bgy-1ezv | 1pp9_Q | 0.958 | 2.722 |
| 162 | 243 | 58 | 1bgy-1ezv | 2fyu_D | 0.958 | 2.791 |
| 162 | 243 | 58 | 1bgy-1ezv | 1pp9_D | 0.958 | 2.716 |
| 162 | 243 | 58 | 1bgy-1ezv | 1ntk_D | 0.958 | 2.753 |
| 162 | 243 | 58 | 1bgy-1ezv | 1ntm_D | 0.928 | 2.801 |
| 162 | 243 | 58 | 1bgy-1ezv | 1nu1_D | 0.958 | 2.793 |
| 162 | 243 | 58 | 1bgy-1ezv | 1be3_D | 1.000 | 0.363 |
| 162 | 243 | 58 | 1bgy-1ezv | 1sqp_D | 0.941 | 2.757 |
| 162 | 243 | 58 | 1bgy-1ezv | 1bgy_P | 1.000 | 0.045 |
| 162 | 243 | 58 | 1bgy-1ezv | 1ntz_D | 0.958 | 2.772 |
| 162 | 243 | 58 | 1bgy-1ezv | 1sqx_D | 0.958 | 2.774 |
| 162 | 243 | 58 | 1bgy-1ezv | 1l0n_D | 0.941 | 2.653 |
| 162 | 243 | 58 | 1bgy-1ezv | 2a06_D | 0.958 | 2.708 |
| 163 | 273 | 58 | 1dea-1ne7 | 1dea_B | 1.000 | 0.260 |
| 163 | 273 | 58 | 1dea-1ne7 | 1fqo_A | 0.996 | 0.224 |
| 163 | 273 | 58 | 1dea-1ne7 | 1frz_B | 1.000 | 0.256 |
| 163 | 273 | 58 | 1ne7-1dea | 1ne7_A | 1.000 | 0.017 |
| 163 | 273 | 58 | 1dea-1ne7 | 1fqo_B | 1.000 | 0.250 |
| 163 | 273 | 58 | 1dea-1ne7 | 1hor_B | 1.000 | 0.262 |
| 163 | 273 | 58 | 1dea-1ne7 | 1fs5_B | 0.996 | 0.567 |
| 163 | 273 | 58 | 1dea-1ne7 | 1hor_A | 1.000 | 0.137 |
| 163 | 273 | 58 | 1dea-1ne7 | 1cd5_A | 0.996 | 1.065 |
| 163 | 273 | 58 | 1dea-1ne7 | 1fsf_A | 0.996 | 1.111 |
| 163 | 273 | 58 | 1dea-1ne7 | 1hot_B | 1.000 | 0.275 |
| 163 | 273 | 58 | 1dea-1ne7 | 1frz_A | 0.996 | 0.220 |
| 163 | 273 | 58 | 1dea-1ne7 | 1fs6_A | 0.996 | 1.057 |
| 163 | 273 | 58 | 1dea-1ne7 | 1hot_A | 1.000 | 0.122 |
| 163 | 273 | 58 | 1dea-1ne7 | 1fs5_A | 0.996 | 0.297 |
| 164 | 399 | 58 | 1kko-1kd0 | 1kkr_A | 1.000 | 0.423 |
| 164 | 399 | 58 | 1kko-1kd0 | 1kkr_B | 1.000 | 0.643 |
| 164 | 399 | 58 | 1kd0-1kko | 1kd0_A | 1.000 | 0.004 |
| 164 | 399 | 58 | 1kko-1kd0 | 1kko_B | 1.000 | 0.381 |
| 164 | 399 | 58 | 1kd0-1kko | 1kd0_B | 1.000 | 0.371 |

| n | length | %ID | refA−refB | altA | SP | RMSD |
|---|---|---|---|---|---|---|
| 165 | 116 | 58 | 1eo6−1gnu | 1eo6_B | 0.966 | 2.585 |
| 166 | 76 | 58 | 1fjg−1pkp | 2hgi_H | 1.000 | 0.962 |
| 166 | 76 | 58 | 1fjg−1pkp | 2j00_E | 1.000 | 0.510 |
| 166 | 76 | 58 | 1fjg−1pkp | 1hr0_E | 1.000 | 0.232 |
| 166 | 76 | 58 | 1fjg−1pkp | 2uu9_E | 1.000 | 0.353 |
| 166 | 76 | 58 | 1fjg−1pkp | 1xnq_E | 1.000 | 0.303 |
| 166 | 76 | 58 | 1fjg−1pkp | 1fka_E | 1.000 | 2.141 |
| 166 | 76 | 58 | 1fjg−1pkp | 2f4v_E | 1.000 | 0.431 |
| 166 | 76 | 58 | 1fjg−1pkp | 2uxb_E | 1.000 | 0.439 |
| 166 | 76 | 58 | 1fjg−1pkp | 1ibk_E | 1.000 | 0.325 |
| 166 | 76 | 58 | 1fjg−1pkp | 1ibm_E | 1.000 | 0.350 |
| 166 | 76 | 58 | 1fjg−1pkp | 2b64_E | 1.000 | 0.785 |
| 166 | 76 | 58 | 1fjg−1pkp | 2hgr_H | 1.000 | 0.891 |
| 166 | 76 | 58 | 1fjg−1pkp | 1xnr_E | 1.000 | 0.340 |
| 166 | 76 | 58 | 1fjg−1pkp | 2b9o_E | 1.000 | 0.765 |
| 166 | 76 | 58 | 1fjg−1pkp | 2hhh_E | 1.000 | 0.357 |
| 166 | 76 | 58 | 1fjg−1pkp | 1hnx_E | 1.000 | 0.239 |
| 166 | 76 | 58 | 1fjg−1pkp | 1hnz_E | 1.000 | 0.305 |
| 166 | 76 | 58 | 1fjg−1pkp | 2v48_E | 1.000 | 0.656 |
| 166 | 76 | 58 | 1fjg−1pkp | 1ibl_E | 1.000 | 0.327 |
| 166 | 76 | 58 | 1fjg−1pkp | 2hgp_H | 1.000 | 1.051 |
| 166 | 76 | 58 | 1fjg−1pkp | 2uxc_E | 1.000 | 0.354 |
| 166 | 76 | 58 | 1fjg−1pkp | 1yl4_H | 1.000 | 0.758 |
| 166 | 76 | 58 | 1fjg−1pkp | 2uxd_E | 1.000 | 0.417 |
| 166 | 76 | 58 | 1fjg−1pkp | 1hnw_E | 1.000 | 0.271 |
| 166 | 76 | 58 | 1fjg−1pkp | 1xmq_E | 1.000 | 0.284 |
| 166 | 76 | 58 | 1fjg−1pkp | 2ow8_f | 1.000 | 1.782 |
| 166 | 76 | 58 | 1fjg−1pkp | 1xmo_E | 1.000 | 0.410 |
| 166 | 76 | 58 | 1fjg−1pkp | 2b9m_E | 1.000 | 0.755 |
| 166 | 76 | 58 | 1fjg−1pkp | 2j02_E | 1.000 | 0.510 |
| 166 | 76 | 58 | 1fjg−1pkp | 2v46_E | 1.000 | 0.657 |
| 166 | 76 | 58 | 1fjg−1pkp | 2qnh_f | 0.887 | 1.981 |
| 166 | 76 | 58 | 1fjg−1pkp | 2uua_E | 1.000 | 0.336 |
| 166 | 76 | 58 | 1fjg−1pkp | 2uub_E | 1.000 | 0.355 |
| 166 | 76 | 58 | 1fjg−1pkp | 2uuc_E | 1.000 | 0.337 |
| 170 | 287 | 57 | 1kph−1kpi | 1kph_D | 0.979 | 0.635 |
| 170 | 287 | 57 | 1kph−1kpi | 1kph_B | 1.000 | 0.382 |
| 170 | 287 | 57 | 1kph−1kpi | 1kph_C | 1.000 | 0.451 |
| 173 | 407 | 57 | 2nmt−1nmt | 1iic_A | 0.997 | 1.268 |
| 173 | 407 | 57 | 1nmt−2nmt | 1iyk_B | 0.990 | 6.901 |
| 173 | 407 | 57 | 1nmt−2nmt | 1nmt_B | 0.997 | 0.298 |
| 173 | 407 | 57 | 2nmt−1nmt | 1iic_B | 0.997 | 1.039 |
| 173 | 407 | 57 | 1nmt−2nmt | 1iyk_A | 0.995 | 6.958 |
| 173 | 407 | 57 | 2nmt−1nmt | 1iid_A | 0.997 | 1.149 |
| 176 | 621 | 57 | 1jqk−1jjy | 1jqk_D | 1.000 | 0.187 |
| 176 | 621 | 57 | 1jqk−1jjy | 1jqk_E | 1.000 | 0.191 |
| 176 | 621 | 57 | 1jqk−1jjy | 1jqk_B | 1.000 | 0.318 |
| 176 | 621 | 57 | 1jqk−1jjy | 1jqk_F | 1.000 | 0.188 |
| 176 | 621 | 57 | 1jqk−1jjy | 1jqk_C | 1.000 | 0.197 |
| 177 | 885 | 57 | 1kbl−1h6z | 1kc7_A | 0.832 | 0.287 |
| 178 | 367 | 57 | 1kbl−1h6z | 1kc7_A | 1.000 | 0.245 |
| 180 | 313 | 57 | 1azw−1qtr | 1azw_B | 1.000 | 0.112 |
| 183 | 159 | 56 | 1q0n−1cbk | 1f9y_A | 1.000 | 0.116 |
| 183 | 159 | 56 | 1q0n−1cbk | 1hka_A | 0.904 | 1.867 |
| 183 | 159 | 56 | 1q0n−1cbk | 1rb0_A | 0.936 | 1.358 |
| 183 | 159 | 56 | 1cbk−1q0n | 1cbk_B | 1.000 | 0.385 |
| 183 | 159 | 56 | 1q0n−1cbk | 1dy3_A | 0.994 | 0.753 |
| 183 | 159 | 56 | 1q0n−1cbk | 1rao_A | 0.968 | 3.906 |
| 183 | 159 | 56 | 1q0n−1cbk | 1eqm_A | 0.936 | 4.030 |
| 183 | 159 | 56 | 1q0n−1cbk | 1ex8_A | 0.968 | 0.611 |
| 184 | 541 | 56 | 1i2d−1g8f | 1i2d_C | 1.000 | 0.436 |
| 184 | 541 | 56 | 1g8f−1i2d | 1g8g_A | 0.978 | 0.951 |
| 184 | 541 | 56 | 1g8f−1i2d | 1g8h_B | 0.998 | 1.012 |
| 184 | 541 | 56 | 1i2d−1g8f | 1i2d_B | 1.000 | 0.411 |
| 184 | 541 | 56 | 1g8f−1i2d | 1g8g_B | 0.984 | 1.072 |
| 184 | 541 | 56 | 1g8f−1i2d | 1g8h_A | 0.998 | 0.991 |
| 188 | 138 | 56 | 1eex−1iwp | 1dio_M | 1.000 | 0.755 |
| 188 | 138 | 56 | 1eex−1iwp | 1eex_M | 1.000 | 0.606 |
| 188 | 138 | 56 | 1eex−1iwp | 1egv_M | 1.000 | 0.610 |
| 188 | 138 | 56 | 1eex−1iwp | 1iwb_M | 1.000 | 0.759 |
| 188 | 138 | 56 | 1eex−1iwp | 1egm_G | 1.000 | 0.187 |

| n | length | %ID | refA−refB | altA | SP | RMSD |
|---|---|---|---|---|---|---|
| 188 | 138 | 56 | 1eex−1iwp | 1dio_G | 1.000 | 0.365 |
| 188 | 138 | 56 | 1eex−1iwp | 1uc5_G | 1.000 | 0.328 |
| 188 | 138 | 56 | 1eex−1iwp | 1uc4_M | 1.000 | 0.485 |
| 188 | 138 | 56 | 1iwp−1eex | 1iwp_M | 1.000 | 0.248 |
| 188 | 138 | 56 | 1eex−1iwp | 1uc4_G | 1.000 | 0.251 |
| 188 | 138 | 56 | 1eex−1iwp | 1egv_G | 1.000 | 0.046 |
| 188 | 138 | 56 | 1eex−1iwp | 1iwb_G | 1.000 | 0.320 |
| 188 | 138 | 56 | 1eex−1iwp | 1egm_M | 1.000 | 0.475 |
| 188 | 138 | 56 | 1eex−1iwp | 1uc5_M | 1.000 | 0.706 |
| 190 | 241 | 56 | 1kko−1kd0 | 1kko_B | 1.000 | 0.203 |
| 190 | 241 | 56 | 1kko−1kd0 | 1kkr_B | 1.000 | 0.237 |
| 190 | 241 | 56 | 1kd0−1kko | 1kd0_B | 1.000 | 0.237 |
| 190 | 241 | 56 | 1kko−1kd0 | 1kkr_A | 1.000 | 0.175 |
| 194 | 73 | 56 | 1utg−1ccd | 2utg_A | 1.000 | 0.544 |
| 194 | 73 | 56 | 1utg−1ccd | 2utg_B | 1.000 | 0.486 |
| 195 | 213 | 56 | 1b43−1a77 | 1b43_B | 0.941 | 0.984 |
| 195 | 213 | 56 | 1a77−1b43 | 1a76_A | 0.915 | 0.603 |
| 197 | 309 | 55 | 1efp−1efv | 1efp_C | 0.984 | 0.535 |
| 199 | 156 | 55 | 1ko7−1jb1 | 1ko7_A | 1.000 | 0.006 |
| 199 | 156 | 55 | 1ko7−1jb1 | 1ko7_B | 1.000 | 0.487 |
| 202 | 319 | 55 | 4pfk−1pfk | 3pfk_A | 1.000 | 0.278 |
| 202 | 319 | 55 | 1pfk−4pfk | 1pfk_B | 1.000 | 0.674 |
| 203 | 115 | 55 | 1kfq−3pmg | 1kfq_B | 1.000 | 0.260 |
| 203 | 115 | 55 | 3pmg−1kfq | 1c47_A | 1.000 | 0.302 |
| 203 | 115 | 55 | 3pmg−1kfq | 1c4g_A | 1.000 | 0.525 |
| 203 | 115 | 55 | 3pmg−1kfq | 1vkl_A | 1.000 | 0.258 |
| 203 | 115 | 55 | 1kfq−3pmg | 1kfi_A | 1.000 | 0.459 |
| 203 | 115 | 55 | 3pmg−1kfq | 3pmg_B | 1.000 | 0.317 |
| 203 | 115 | 55 | 3pmg−1kfq | 1jdy_A | 1.000 | 0.261 |
| 203 | 115 | 55 | 3pmg−1kfq | 1jdy_B | 1.000 | 0.343 |
| 203 | 115 | 55 | 1kfq−3pmg | 1kfi_B | 1.000 | 0.467 |
| 203 | 115 | 55 | 3pmg−1kfq | 1lxt_B | 1.000 | 0.374 |
| 203 | 115 | 55 | 3pmg−1kfq | 1c47_B | 1.000 | 0.358 |
| 203 | 115 | 55 | 3pmg−1kfq | 1c4g_B | 1.000 | 0.502 |
| 203 | 115 | 55 | 3pmg−1kfq | 1lxt_A | 1.000 | 0.305 |
| 203 | 115 | 55 | 3pmg−1kfq | 1vkl_B | 1.000 | 0.323 |
| 205 | 147 | 55 | 1fjg−1pkp | 1yl4_H | 1.000 | 0.753 |
| 205 | 147 | 55 | 1fjg−1pkp | 2j02_E | 1.000 | 0.652 |
| 205 | 147 | 55 | 1fjg−1pkp | 1hnx_E | 1.000 | 0.318 |
| 205 | 147 | 55 | 1fjg−1pkp | 1xmq_E | 1.000 | 0.363 |
| 205 | 147 | 55 | 1fjg−1pkp | 1hr0_E | 1.000 | 0.308 |
| 205 | 147 | 55 | 1fjg−1pkp | 2hgp_H | 1.000 | 1.057 |
| 205 | 147 | 55 | 1fjg−1pkp | 2qnh_f | 1.000 | 1.916 |
| 205 | 147 | 55 | 1fjg−1pkp | 1fka_E | 1.000 | 4.446 |
| 205 | 147 | 55 | 1fjg−1pkp | 2v48_E | 1.000 | 0.688 |
| 205 | 147 | 55 | 1fjg−1pkp | 2hgr_H | 1.000 | 0.858 |
| 205 | 147 | 55 | 1fjg−1pkp | 2uu9_E | 1.000 | 0.431 |
| 205 | 147 | 55 | 1fjg−1pkp | 1ibm_E | 1.000 | 0.383 |
| 205 | 147 | 55 | 1fjg−1pkp | 2b9o_E | 1.000 | 0.767 |
| 205 | 147 | 55 | 1fjg−1pkp | 2uxc_E | 1.000 | 0.400 |
| 205 | 147 | 55 | 1fjg−1pkp | 1ibk_E | 1.000 | 0.368 |
| 205 | 147 | 55 | 1fjg−1pkp | 2hgi_H | 1.000 | 0.922 |
| 205 | 147 | 55 | 1fjg−1pkp | 1xnq_E | 1.000 | 0.383 |
| 205 | 147 | 55 | 1fjg−1pkp | 1hnz_E | 1.000 | 0.378 |
| 205 | 147 | 55 | 1fjg−1pkp | 2uua_E | 1.000 | 0.406 |
| 205 | 147 | 55 | 1fjg−1pkp | 1ibl_E | 1.000 | 0.412 |
| 205 | 147 | 55 | 1fjg−1pkp | 2b9m_E | 1.000 | 0.759 |
| 205 | 147 | 55 | 1fjg−1pkp | 2ow8_f | 1.000 | 1.777 |
| 205 | 147 | 55 | 1fjg−1pkp | 1hnw_E | 1.000 | 0.326 |
| 205 | 147 | 55 | 1fjg−1pkp | 2f4v_E | 1.000 | 0.478 |
| 205 | 147 | 55 | 1fjg−1pkp | 2j00_E | 1.000 | 0.653 |
| 205 | 147 | 55 | 1fjg−1pkp | 2b64_E | 1.000 | 0.779 |
| 205 | 147 | 55 | 1fjg−1pkp | 2hhh_E | 1.000 | 0.425 |
| 205 | 147 | 55 | 1fjg−1pkp | 1xnr_E | 1.000 | 0.406 |
| 205 | 147 | 55 | 1fjg−1pkp | 1xmo_E | 1.000 | 0.498 |
| 205 | 147 | 55 | 1fjg−1pkp | 2uuc_E | 1.000 | 0.405 |
| 205 | 147 | 55 | 1fjg−1pkp | 2uub_E | 1.000 | 0.410 |
| 205 | 147 | 55 | 1fjg−1pkp | 2uxd_E | 1.000 | 0.519 |
| 205 | 147 | 55 | 1fjg−1pkp | 2uxb_E | 1.000 | 0.495 |
| 205 | 147 | 55 | 1fjg−1pkp | 2v46_E | 1.000 | 0.689 |
| 207 | 521 | 54 | 2occ−1ar1 | 2occ_N | 1.000 | 0.033 |

| n | length | %ID | refA−refB | altA | SP | RMSD |
|---|---|---|---|---|---|---|
| 207 | 521 | 54 | 2occ−1ar1 | 1ocr_N | 0.990 | 0.216 |
| 207 | 521 | 54 | 2occ−1ar1 | 1oco_A | 0.990 | 0.247 |
| 207 | 521 | 54 | 2occ−1ar1 | 1occ_A | 1.000 | 0.151 |
| 207 | 521 | 54 | 2occ−1ar1 | 1oco_N | 0.990 | 0.250 |
| 207 | 521 | 54 | 2occ−1ar1 | 1ocz_N | 0.998 | 0.185 |
| 207 | 521 | 54 | 2occ−1ar1 | 1ocz_A | 0.998 | 0.182 |
| 207 | 521 | 54 | 2occ−1ar1 | 1occ_N | 1.000 | 0.151 |
| 207 | 521 | 54 | 2occ−1ar1 | 1ocr_A | 0.990 | 0.214 |
| 214 | 216 | 54 | 1gs0−1efy | 1gs0_B | 1.000 | 0.212 |
| 216 | 71 | 54 | 1fjg−1pkp | 2b64_E | 1.000 | 0.694 |
| 216 | 71 | 54 | 1fjg−1pkp | 2qnh_f | 1.000 | 1.339 |
| 216 | 71 | 54 | 1fjg−1pkp | 2uxc_E | 1.000 | 0.365 |
| 216 | 71 | 54 | 1fjg−1pkp | 1xnr_E | 1.000 | 0.413 |
| 216 | 71 | 54 | 1fjg−1pkp | 1ibl_E | 1.000 | 0.421 |
| 216 | 71 | 54 | 1fjg−1pkp | 2uua_E | 1.000 | 0.388 |
| 216 | 71 | 54 | 1fjg−1pkp | 2uuc_E | 1.000 | 0.391 |
| 216 | 71 | 54 | 1fjg−1pkp | 1fka_E | 1.000 | 5.816 |
| 216 | 71 | 54 | 1fjg−1pkp | 1hnx_E | 1.000 | 0.345 |
| 216 | 71 | 54 | 1fjg−1pkp | 2b9m_E | 1.000 | 0.694 |
| 216 | 71 | 54 | 1fjg−1pkp | 2b9o_E | 1.000 | 0.694 |
| 216 | 71 | 54 | 1fjg−1pkp | 2hgi_H | 1.000 | 0.817 |
| 216 | 71 | 54 | 1fjg−1pkp | 1ibm_E | 1.000 | 0.381 |
| 216 | 71 | 54 | 1fjg−1pkp | 2uxb_E | 1.000 | 0.492 |
| 216 | 71 | 54 | 1fjg−1pkp | 1xmo_E | 1.000 | 0.472 |
| 216 | 71 | 54 | 1fjg−1pkp | 1hr0_E | 1.000 | 0.330 |
| 216 | 71 | 54 | 1fjg−1pkp | 1xnq_E | 1.000 | 0.381 |
| 216 | 71 | 54 | 1fjg−1pkp | 2hgr_H | 1.000 | 0.774 |
| 216 | 71 | 54 | 1fjg−1pkp | 2f4v_E | 1.000 | 0.504 |
| 216 | 71 | 54 | 1fjg−1pkp | 1yl4_H | 1.000 | 0.674 |
| 216 | 71 | 54 | 1fjg−1pkp | 1hnw_E | 1.000 | 0.320 |
| 216 | 71 | 54 | 1fjg−1pkp | 2ow8_f | 1.000 | 1.371 |
| 216 | 71 | 54 | 1fjg−1pkp | 1hnz_E | 1.000 | 0.358 |
| 216 | 71 | 54 | 1fjg−1pkp | 2hgp_H | 1.000 | 0.993 |
| 216 | 71 | 54 | 1fjg−1pkp | 1ibk_E | 1.000 | 0.362 |
| 216 | 71 | 54 | 1fjg−1pkp | 1xmq_E | 1.000 | 0.340 |
| 216 | 71 | 54 | 1fjg−1pkp | 2uub_E | 1.000 | 0.386 |
| 216 | 71 | 54 | 1fjg−1pkp | 2hhh_E | 1.000 | 0.440 |
| 216 | 71 | 54 | 1fjg−1pkp | 2j00_E | 1.000 | 0.742 |
| 216 | 71 | 54 | 1fjg−1pkp | 2j02_E | 1.000 | 0.740 |
| 216 | 71 | 54 | 1fjg−1pkp | 2uu9_E | 1.000 | 0.412 |
| 216 | 71 | 54 | 1fjg−1pkp | 2uxd_E | 1.000 | 0.514 |
| 216 | 71 | 54 | 1fjg−1pkp | 2v46_E | 1.000 | 0.696 |
| 216 | 71 | 54 | 1fjg−1pkp | 2v48_E | 1.000 | 0.695 |
| 218 | 111 | 54 | 2kau−1ubp | 1fwe_B | 1.000 | 0.107 |
| 218 | 111 | 54 | 2kau−1ubp | 1fwa_B | 1.000 | 0.093 |
| 218 | 111 | 54 | 2kau−1ubp | 1fwg_B | 1.000 | 0.101 |
| 218 | 111 | 54 | 1ubp−2kau | 2ubp_B | 1.000 | 0.158 |
| 218 | 111 | 54 | 2kau−1ubp | 1fwh_B | 1.000 | 0.112 |
| 218 | 111 | 54 | 2kau−1ubp | 1fwi_B | 1.000 | 0.098 |
| 218 | 111 | 54 | 2kau−1ubp | 1fwd_B | 1.000 | 0.093 |
| 218 | 111 | 54 | 2kau−1ubp | 1fwc_B | 1.000 | 0.086 |
| 218 | 111 | 54 | 2kau−1ubp | 1fwb_B | 1.000 | 0.093 |
| 218 | 111 | 54 | 2kau−1ubp | 1fwf_B | 1.000 | 0.107 |
| 218 | 111 | 54 | 2kau−1ubp | 1fwj_B | 1.000 | 0.086 |
| 218 | 111 | 54 | 2kau−1ubp | 1kra_B | 1.000 | 0.149 |
| 218 | 111 | 54 | 2kau−1ubp | 1krb_B | 1.000 | 0.129 |
| 218 | 111 | 54 | 2kau−1ubp | 1krc_B | 1.000 | 0.092 |
| 220 | 218 | 53 | 1a35−1ois | 1a36_A | 1.000 | 0.600 |
| 221 | 114 | 53 | 1ed1−1hiw | 1ecw_A | 1.000 | 0.355 |
| 224 | 568 | 53 | 1qr6−1llq | 1efk_A | 0.992 | 1.515 |
| 224 | 568 | 53 | 1qr6−1llq | 1efk_B | 0.998 | 1.494 |
| 224 | 568 | 53 | 1qr6−1llq | 1efk_D | 0.998 | 1.511 |
| 224 | 568 | 53 | 1qr6−1llq | 1efk_C | 0.998 | 1.493 |
| 224 | 568 | 53 | 1qr6−1llq | 1efl_A | 1.000 | 1.522 |
| 224 | 568 | 53 | 1qr6−1llq | 1efl_D | 0.992 | 1.516 |
| 224 | 568 | 53 | 1llq−1qr6 | 1o0s_A | 0.996 | 0.262 |
| 224 | 568 | 53 | 1qr6−1llq | 1efl_C | 0.994 | 1.496 |
| 224 | 568 | 53 | 1qr6−1llq | 1pjl_A | 0.992 | 1.055 |
| 224 | 568 | 53 | 1qr6−1llq | 1efl_B | 0.825 | 1.500 |
| 225 | 554 | 53 | 1mvm−4dpv | 1z1c_A | 0.998 | 0.615 |
| 227 | 327 | 53 | 1b43−1a77 | 1b43_B | 0.936 | 0.964 |

n: internal numbering; length of alignment; % sequence ID (cf. Homstrad); refA−refB: reference alignment; altA: alternative for struc. A; SP: sum−of−pairs score of alt. alignment w/r reference

| n | length | %ID | refA−refB | altA | SP | RMSD |
|---|---|---|---|---|---|---|
| 227 | 327 | 53 | 1a77−1b43 | 1a76_A | 0.953 | 0.510 |
| 230 | 138 | 52 | 1kxr−1kfu | 1kxr_B | 1.000 | 0.785 |
| 231 | 157 | 52 | 1iqc−1eb7 | 1iqc_B | 0.973 | 0.510 |
| 231 | 157 | 52 | 1iqc−1eb7 | 1iqc_D | 0.925 | 1.565 |
| 231 | 157 | 52 | 1iqc−1eb7 | 1iqc_C | 0.973 | 0.563 |
| 233 | 59 | 52 | 1prb−1gjt | 1tf0_B | 0.827 | 2.409 |
| 234 | 773 | 52 | 1ea0−1llw | 1ea0_B | 0.993 | 0.290 |
| 234 | 773 | 52 | 1llw−1ea0 | 1llz_A | 1.000 | 0.315 |
| 234 | 773 | 52 | 1llw−1ea0 | 1lm1_A | 0.992 | 0.233 |
| 237 | 306 | 52 | 1aq0−1ghs | 1aq0_B | 0.993 | 0.401 |
| 237 | 306 | 52 | 1aq0−1ghs | 1ghr_A | 1.000 | 0.284 |
| 237 | 306 | 52 | 1ghs−1aq0 | 1ghs_B | 0.997 | 0.297 |
| 243 | 205 | 52 | 1sac−1b09 | 1gyk_B | 1.000 | 0.309 |
| 243 | 205 | 52 | 1sac−1b09 | 2a3w_O | 1.000 | 0.249 |
| 243 | 205 | 52 | 1sac−1b09 | 2a3w_P | 1.000 | 0.229 |
| 243 | 205 | 52 | 1sac−1b09 | 2a3w_S | 1.000 | 0.275 |
| 243 | 205 | 52 | 1sac−1b09 | 1lgn_D | 0.995 | 0.222 |
| 243 | 205 | 52 | 1sac−1b09 | 2a3x_D | 0.995 | 0.313 |
| 243 | 205 | 52 | 1sac−1b09 | 1lgn_A | 1.000 | 0.403 |
| 243 | 205 | 52 | 1sac−1b09 | 2a3x_I | 1.000 | 0.334 |
| 243 | 205 | 52 | 1sac−1b09 | 1sac_E | 1.000 | 0.208 |
| 243 | 205 | 52 | 1sac−1b09 | 1gyk_A | 0.995 | 0.297 |
| 243 | 205 | 52 | 1sac−1b09 | 2a3x_H | 1.000 | 0.289 |
| 243 | 205 | 52 | 1sac−1b09 | 1lgn_C | 0.995 | 0.217 |
| 243 | 205 | 52 | 1sac−1b09 | 2a3w_Q | 1.000 | 0.271 |
| 243 | 205 | 52 | 1sac−1b09 | 2a3w_R | 1.000 | 0.243 |
| 243 | 205 | 52 | 1sac−1b09 | 2a3y_B | 1.000 | 0.285 |
| 243 | 205 | 52 | 1sac−1b09 | 2a3x_B | 1.000 | 0.383 |
| 243 | 205 | 52 | 1sac−1b09 | 2a3y_C | 1.000 | 0.244 |
| 243 | 205 | 52 | 1sac−1b09 | 2a3x_J | 1.000 | 0.371 |
| 243 | 205 | 52 | 1sac−1b09 | 2a3w_M | 1.000 | 0.272 |
| 243 | 205 | 52 | 1sac−1b09 | 1lgn_B | 0.995 | 0.224 |
| 243 | 205 | 52 | 1sac−1b09 | 2a3x_E | 0.995 | 0.334 |
| 243 | 205 | 52 | 1sac−1b09 | 2a3w_C | 1.000 | 0.351 |
| 243 | 205 | 52 | 1sac−1b09 | 2a3y_D | 1.000 | 0.259 |
| 243 | 205 | 52 | 1sac−1b09 | 2a3w_T | 1.000 | 0.273 |
| 243 | 205 | 52 | 1sac−1b09 | 2a3x_A | 0.995 | 0.379 |
| 243 | 205 | 52 | 1sac−1b09 | 2a3x_C | 0.995 | 0.323 |
| 243 | 205 | 52 | 1sac−1b09 | 2a3w_D | 1.000 | 0.270 |
| 243 | 205 | 52 | 1sac−1b09 | 2a3x_G | 1.000 | 0.421 |
| 243 | 205 | 52 | 1sac−1b09 | 2a3y_A | 1.000 | 0.375 |
| 243 | 205 | 52 | 1sac−1b09 | 2a3w_N | 1.000 | 0.281 |
| 243 | 205 | 52 | 1sac−1b09 | 1gyk_E | 0.995 | 0.304 |
| 243 | 205 | 52 | 1sac−1b09 | 2a3y_E | 1.000 | 0.277 |
| 243 | 205 | 52 | 1sac−1b09 | 1gyk_D | 1.000 | 0.296 |
| 243 | 205 | 52 | 1sac−1b09 | 1sac_D | 1.000 | 0.223 |
| 243 | 205 | 52 | 1sac−1b09 | 2a3x_F | 1.000 | 0.424 |
| 243 | 205 | 52 | 1sac−1b09 | 2a3w_F | 1.000 | 0.247 |
| 243 | 205 | 52 | 1sac−1b09 | 1lgn_E | 0.995 | 0.221 |
| 243 | 205 | 52 | 1sac−1b09 | 2a3w_G | 1.000 | 0.244 |
| 243 | 205 | 52 | 1sac−1b09 | 1sac_B | 1.000 | 0.203 |
| 243 | 205 | 52 | 1sac−1b09 | 1gyk_C | 1.000 | 0.298 |
| 243 | 205 | 52 | 1sac−1b09 | 2a3w_E | 1.000 | 0.246 |
| 243 | 205 | 52 | 1sac−1b09 | 2a3w_B | 1.000 | 0.261 |
| 243 | 205 | 52 | 1sac−1b09 | 1sac_C | 1.000 | 0.213 |
| 243 | 205 | 52 | 1sac−1b09 | 2a3w_A | 1.000 | 0.353 |
| 243 | 205 | 52 | 1sac−1b09 | 2a3w_H | 1.000 | 0.303 |
| 243 | 205 | 52 | 1sac−1b09 | 2a3w_I | 1.000 | 0.257 |
| 243 | 205 | 52 | 1sac−1b09 | 2a3w_J | 1.000 | 0.245 |
| 243 | 205 | 52 | 1sac−1b09 | 2a3w_K | 1.000 | 0.270 |
| 243 | 205 | 52 | 1sac−1b09 | 2a3w_L | 1.000 | 0.251 |
| 243 | 205 | 52 | 1b09−1sac | 1b09_B | 1.000 | 0.274 |
| 243 | 205 | 52 | 1b09−1sac | 1b09_C | 1.000 | 0.284 |
| 243 | 205 | 52 | 1b09−1sac | 1b09_D | 1.000 | 0.250 |
| 243 | 205 | 52 | 1b09−1sac | 1b09_E | 1.000 | 0.288 |
| 243 | 205 | 52 | 1b09−1sac | 1gnh_A | 0.995 | 0.355 |
| 243 | 205 | 52 | 1b09−1sac | 1gnh_B | 0.995 | 0.356 |
| 243 | 205 | 52 | 1b09−1sac | 1gnh_C | 0.995 | 0.354 |
| 243 | 205 | 52 | 1b09−1sac | 1gnh_D | 0.956 | 1.721 |
| 243 | 205 | 52 | 1b09−1sac | 1gnh_E | 0.995 | 0.355 |
| 243 | 205 | 52 | 1b09−1sac | 1gnh_F | 0.995 | 0.354 |
| 243 | 205 | 52 | 1b09−1sac | 1gnh_G | 0.995 | 0.356 |
| 243 | 205 | 52 | 1b09−1sac | 1gnh_H | 0.995 | 0.356 |
| 243 | 205 | 52 | 1b09−1sac | 1gnh_I | 0.995 | 0.354 |
| 243 | 205 | 52 | 1b09−1sac | 1gnh_J | 0.956 | 1.725 |
| 243 | 205 | 52 | 1b09−1sac | 1lj7_B | 0.995 | 0.677 |
| 243 | 205 | 52 | 1b09−1sac | 1lj7_F | 0.995 | 0.517 |
| 243 | 205 | 52 | 1b09−1sac | 1lj7_G | 0.995 | 0.865 |
| 243 | 205 | 52 | 1b09−1sac | 1lj7_J | 0.995 | 0.674 |
| 244 | 135 | 52 | 1kfq−3pmg | 1kfq_B | 1.000 | 0.310 |
| 244 | 135 | 52 | 3pmg−1kfq | 1c47_A | 1.000 | 0.466 |
| 244 | 135 | 52 | 3pmg−1kfq | 1c4g_A | 0.953 | 2.287 |
| 244 | 135 | 52 | 3pmg−1kfq | 1vkl_A | 1.000 | 0.546 |
| 244 | 135 | 52 | 1kfq−3pmg | 1kfi_A | 1.000 | 1.171 |
| 244 | 135 | 52 | 3pmg−1kfq | 3pmg_B | 1.000 | 0.901 |
| 244 | 135 | 52 | 3pmg−1kfq | 1jdy_A | 1.000 | 0.626 |
| 244 | 135 | 52 | 3pmg−1kfq | 1jdy_B | 1.000 | 0.858 |
| 244 | 135 | 52 | 1kfq−3pmg | 1kfi_B | 1.000 | 1.037 |
| 244 | 135 | 52 | 3pmg−1kfq | 1lxt_B | 0.961 | 1.038 |
| 244 | 135 | 52 | 3pmg−1kfq | 1c47_B | 0.961 | 0.963 |
| 244 | 135 | 52 | 3pmg−1kfq | 1c4g_B | 1.000 | 1.030 |
| 244 | 135 | 52 | 3pmg−1kfq | 1lxt_A | 1.000 | 0.595 |
| 244 | 135 | 52 | 3pmg−1kfq | 1vkl_B | 1.000 | 0.916 |
| 247 | 184 | 52 | 1h6f−1xbr | 1h6f_B | 1.000 | 0.633 |
| 250 | 164 | 51 | 1mty−1mhy | 1mmo_G | 1.000 | 0.223 |
| 250 | 164 | 51 | 1mty−1mhy | 1mty_H | 1.000 | 0.200 |
| 250 | 164 | 51 | 1mhy−1mty | 1mhz_G | 1.000 | 0.261 |
| 250 | 164 | 51 | 1mty−1mhy | 1mmo_H | 1.000 | 0.280 |
| 252 | 353 | 50 | 2phl−2cav | 2phl_C | 0.997 | 0.431 |
| 252 | 353 | 50 | 2phl−2cav | 2phl_B | 0.997 | 0.381 |
| 253 | 166 | 50 | 1f15−1laj | 1f15_B | 0.961 | 2.324 |
| 253 | 166 | 50 | 1f15−1laj | 1f15_C | 0.961 | 2.297 |
| 253 | 166 | 50 | 1laj−1f15 | 1laj_B | 0.961 | 4.243 |
| 253 | 166 | 50 | 1laj−1f15 | 1laj_C | 0.961 | 4.326 |
| 261 | 60 | 50 | 1g19−2reb | 1mo5_A | 1.000 | 0.309 |
| 261 | 60 | 50 | 1g19−2reb | 1mo6_A | 1.000 | 0.344 |
| 261 | 60 | 50 | 1g19−2reb | 1g18_A | 1.000 | 0.271 |
| 261 | 60 | 50 | 1g19−2reb | 1mo4_A | 1.000 | 0.310 |
| 261 | 60 | 50 | 1g19−2reb | 1mo3_A | 1.000 | 0.356 |
| 262 | 240 | 50 | 2erc−1yub | 1qaq_A | 0.996 | 1.504 |
| 262 | 240 | 50 | 2erc−1yub | 1qam_A | 0.996 | 1.439 |
| 262 | 240 | 50 | 2erc−1yub | 2erc_B | 1.000 | 0.512 |
| 262 | 240 | 50 | 2erc−1yub | 1qan_A | 0.996 | 1.510 |
| 262 | 240 | 50 | 2erc−1yub | 1qao_A | 0.996 | 1.476 |
| 266 | 445 | 49 | 1ax4−1tpl | 1ax4_B | 1.000 | 0.552 |
| 266 | 445 | 49 | 1ax4−1tpl | 1ax4_D | 1.000 | 0.265 |
| 266 | 445 | 49 | 1ax4−1tpl | 1ax4_C | 1.000 | 0.311 |
| 268 | 183 | 49 | 1dzr−1ep0 | 1dzt_B | 0.972 | 1.319 |
| 268 | 183 | 49 | 1dzr−1ep0 | 1dzr_B | 1.000 | 1.263 |
| 268 | 183 | 49 | 1ep0−1dzr | 1epz_A | 1.000 | 0.187 |
| 268 | 183 | 49 | 1dzr−1ep0 | 1dzt_A | 1.000 | 0.226 |
| 277 | 123 | 49 | 1mxb−1qm4 | 1xrc_A | 1.000 | 0.294 |
| 277 | 123 | 49 | 1mxb−1qm4 | 1p7l_B | 1.000 | 1.150 |
| 277 | 123 | 49 | 1qm4−1mxb | 1o93_B | 1.000 | 0.116 |
| 277 | 123 | 49 | 1qm4−1mxb | 1o9t_A | 1.000 | 0.116 |
| 277 | 123 | 49 | 1mxb−1qm4 | 1p7l_C | 1.000 | 1.138 |
| 277 | 123 | 49 | 1mxb−1qm4 | 1mxa_A | 1.000 | 0.288 |
| 277 | 123 | 49 | 1qm4−1mxb | 1o90_B | 1.000 | 0.121 |
| 277 | 123 | 49 | 1qm4−1mxb | 1o90_A | 1.000 | 0.117 |
| 277 | 123 | 49 | 1mxb−1qm4 | 1p7l_D | 1.000 | 1.139 |
| 277 | 123 | 49 | 1mxb−1qm4 | 1fug_A | 0.950 | 1.604 |
| 277 | 123 | 49 | 1qm4−1mxb | 1qm4_B | 1.000 | 0.040 |
| 277 | 123 | 49 | 1qm4−1mxb | 1o92_B | 1.000 | 0.117 |
| 277 | 123 | 49 | 1mxb−1qm4 | 1mxc_A | 1.000 | 0.127 |
| 277 | 123 | 49 | 1qm4−1mxb | 1o92_A | 1.000 | 0.114 |
| 277 | 123 | 49 | 1mxb−1qm4 | 1rg9_B | 1.000 | 1.149 |
| 277 | 123 | 49 | 1mxb−1qm4 | 1xra_A | 1.000 | 0.306 |
| 277 | 123 | 49 | 1qm4−1mxb | 1o9t_B | 1.000 | 0.119 |
| 277 | 123 | 49 | 1mxb−1qm4 | 1fug_B | 1.000 | 1.449 |
| 277 | 123 | 49 | 1mxb−1qm4 | 1rg9_A | 1.000 | 1.146 |
| 277 | 123 | 49 | 1qm4−1mxb | 1o93_A | 1.000 | 0.114 |
| 277 | 123 | 49 | 1mxb−1qm4 | 1p7l_A | 1.000 | 1.146 |
| 277 | 123 | 49 | 1mxb−1qm4 | 1rg9_D | 1.000 | 1.138 |
| 277 | 123 | 49 | 1mxb−1qm4 | 1rg9_C | 1.000 | 1.137 |
| 279 | 387 | 48 | 1kij−1ei1 | 1kij_B | 0.984 | 0.765 |
| 280 | 312 | 48 | 1iqc−1eb7 | 1iqc_D | 1.000 | 1.165 |
| 280 | 312 | 48 | 1iqc−1eb7 | 1iqc_B | 1.000 | 0.428 |
| 280 | 312 | 48 | 1iqc−1eb7 | 1iqc_C | 0.942 | 0.434 |
| 282 | 365 | 48 | 1jq5−1kq3 | 1jq5_A | 1.000 | 0.003 |
| 283 | 297 | 48 | 1qr6−1llq | 1efk_B | 0.996 | 1.174 |
| 283 | 297 | 48 | 1qr6−1llq | 1efk_C | 0.996 | 1.182 |
| 283 | 297 | 48 | 1qr6−1llq | 1efl_A | 1.000 | 1.184 |
| 283 | 297 | 48 | 1qr6−1llq | 1efl_C | 0.996 | 1.159 |
| 283 | 297 | 48 | 1qr6−1llq | 1pjl_A | 1.000 | 1.004 |
| 283 | 297 | 48 | 1qr6−1llq | 1efk_D | 0.996 | 1.192 |
| 283 | 297 | 48 | 1llq−1qr6 | 1o0s_A | 1.000 | 0.305 |
| 283 | 297 | 48 | 1qr6−1llq | 1efl_D | 1.000 | 1.179 |
| 283 | 297 | 48 | 1qr6−1llq | 1efl_B | 0.996 | 1.158 |
| 283 | 297 | 48 | 1qr6−1llq | 1efk_A | 1.000 | 1.182 |
| 284 | 345 | 48 | 1fa0−1f5a | 1fa0_B | 1.000 | 0.711 |
| 288 | 68 | 48 | 1jj2−1rl2 | 1k9m_C | 1.000 | 0.074 |
| 288 | 68 | 48 | 1jj2−1rl2 | 1kc8_C | 1.000 | 0.071 |
| 288 | 68 | 48 | 1jj2−1rl2 | 1m1k_C | 1.000 | 0.071 |
| 288 | 68 | 48 | 1jj2−1rl2 | 1q81_C | 1.000 | 0.142 |
| 288 | 68 | 48 | 1jj2−1rl2 | 1w2b_A | 1.000 | 0.224 |
| 288 | 68 | 48 | 1jj2−1rl2 | 1qvg_A | 1.000 | 0.114 |
| 288 | 68 | 48 | 1jj2−1rl2 | 1kd1_C | 1.000 | 0.076 |
| 288 | 68 | 48 | 1jj2−1rl2 | 1kqs_A | 1.000 | 0.088 |
| 288 | 68 | 48 | 1jj2−1rl2 | 1nji_C | 1.000 | 0.055 |
| 288 | 68 | 48 | 1jj2−1rl2 | 1k73_C | 1.000 | 0.036 |
| 288 | 68 | 48 | 1jj2−1rl2 | 1q82_C | 1.000 | 0.092 |
| 288 | 68 | 48 | 1jj2−1rl2 | 1m90_C | 1.000 | 0.067 |
| 288 | 68 | 48 | 1jj2−1rl2 | 1n8r_C | 1.000 | 0.086 |
| 288 | 68 | 48 | 1jj2−1rl2 | 1q7y_C | 1.000 | 0.126 |
| 288 | 68 | 48 | 1jj2−1rl2 | 1qvf_A | 1.000 | 0.046 |
| 288 | 68 | 48 | 1jj2−1rl2 | 1k8a_C | 1.000 | 0.071 |
| 288 | 68 | 48 | 1jj2−1rl2 | 1q86_C | 1.000 | 0.131 |
| 288 | 68 | 48 | 1rl2−1jj2 | 1rl2_B | 1.000 | 0.663 |
| 291 | 319 | 48 | 1evu−1g0d | 1evu_B | 1.000 | 0.235 |
| 294 | 310 | 47 | 1b7b−1e19 | 1b7b_B | 1.000 | 0.214 |
| 294 | 310 | 47 | 1b7b−1e19 | 1b7b_C | 1.000 | 0.190 |
| 294 | 310 | 47 | 1b7b−1e19 | 1b7b_D | 1.000 | 0.253 |
| 294 | 310 | 47 | 1e19−1b7b | 1e19_B | 1.000 | 0.455 |
| 295 | 225 | 47 | 1i6v−1bdf | 1i6v_B | 1.000 | 1.964 |
| 296 | 158 | 47 | 1bcf−1jgc | 1bfr_C | 1.000 | 0.252 |
| 296 | 158 | 47 | 1jgc−1bcf | 1jgc_B | 1.000 | 0.014 |
| 296 | 158 | 47 | 1jgc−1bcf | 1jgc_C | 1.000 | 0.013 |
| 296 | 158 | 47 | 1bcf−1jgc | 1bfr_E | 1.000 | 0.252 |
| 296 | 158 | 47 | 1bcf−1jgc | 1bfr_R | 1.000 | 0.252 |
| 296 | 158 | 47 | 1bcf−1jgc | 1bfr_S | 1.000 | 0.252 |
| 296 | 158 | 47 | 1bcf−1jgc | 1bfr_I | 1.000 | 0.252 |
| 296 | 158 | 47 | 1bcf−1jgc | 1bfr_P | 1.000 | 0.252 |
| 296 | 158 | 47 | 1bcf−1jgc | 1bcf_J | 1.000 | 0.012 |
| 296 | 158 | 47 | 1bcf−1jgc | 1bfr_D | 1.000 | 0.252 |
| 296 | 158 | 47 | 1jgc−1bcf | 1jgc_A | 1.000 | 0.003 |
| 296 | 158 | 47 | 1bcf−1jgc | 1bfr_K | 1.000 | 0.252 |
| 296 | 158 | 47 | 1bcf−1jgc | 1bfr_V | 1.000 | 0.252 |
| 296 | 158 | 47 | 1bcf−1jgc | 1bcf_H | 1.000 | 0.012 |
| 296 | 158 | 47 | 1bcf−1jgc | 1bcf_L | 1.000 | 0.012 |
| 296 | 158 | 47 | 1bcf−1jgc | 1bfr_B | 1.000 | 0.252 |
| 296 | 158 | 47 | 1bcf−1jgc | 1bfr_H | 1.000 | 0.252 |
| 296 | 158 | 47 | 1bcf−1jgc | 1bcf_F | 1.000 | 0.012 |
| 296 | 158 | 47 | 1bcf−1jgc | 1bfr_M | 1.000 | 0.252 |
| 296 | 158 | 47 | 1bcf−1jgc | 1bcf_I | 1.000 | 0.001 |
| 296 | 158 | 47 | 1bcf−1jgc | 1bfr_A | 1.000 | 0.252 |
| 296 | 158 | 47 | 1bcf−1jgc | 1bfr_J | 1.000 | 0.252 |
| 296 | 158 | 47 | 1bcf−1jgc | 1bfr_G | 1.000 | 0.252 |
| 296 | 158 | 47 | 1bcf−1jgc | 1bfr_O | 1.000 | 0.252 |
| 296 | 158 | 47 | 1bcf−1jgc | 1bfr_Q | 1.000 | 0.252 |
| 296 | 158 | 47 | 1bcf−1jgc | 1bfr_F | 1.000 | 0.252 |
| 296 | 158 | 47 | 1bcf−1jgc | 1bfr_L | 1.000 | 0.252 |
| 296 | 158 | 47 | 1bcf−1jgc | 1bfr_N | 1.000 | 0.252 |
| 296 | 158 | 47 | 1bcf−1jgc | 1bfr_T | 1.000 | 0.252 |

n: internal numbering; length of alignment; % sequence ID (cf. Homstrad); refA−refB: reference alignment; altA: alternative for struc. A; SP: sum−of−pairs score of alt. alignment w/r reference

| n | length | %ID | refA−refB | altA | SP | RMSD |
|---|---|---|---|---|---|---|
| 296 | 158 | 47 | 1bcf−1jgc | 1bfr_W | 1.000 | 0.252 |
| 296 | 158 | 47 | 1bcf−1jgc | 1bfr_X | 1.000 | 0.252 |
| 296 | 158 | 47 | 1bcf−1jgc | 1bfr_U | 1.000 | 0.252 |
| 296 | 158 | 47 | 1bcf−1jgc | 2htn_A | 1.000 | 0.294 |
| 296 | 158 | 47 | 1bcf−1jgc | 2htn_B | 1.000 | 0.295 |
| 296 | 158 | 47 | 1bcf−1jgc | 2htn_C | 1.000 | 0.294 |
| 296 | 158 | 47 | 1bcf−1jgc | 2htn_D | 1.000 | 0.297 |
| 296 | 158 | 47 | 1bcf−1jgc | 2htn_E | 1.000 | 0.295 |
| 296 | 158 | 47 | 1bcf−1jgc | 2htn_F | 1.000 | 0.295 |
| 296 | 158 | 47 | 1bcf−1jgc | 2htn_G | 1.000 | 0.295 |
| 296 | 158 | 47 | 1bcf−1jgc | 2htn_H | 1.000 | 0.297 |
| 296 | 158 | 47 | 1bcf−1jgc | 1bcf_B | 1.000 | 0.012 |
| 296 | 158 | 47 | 1bcf−1jgc | 1bcf_D | 1.000 | 0.012 |
| 300 | 105 | 47 | 1m0w−2hgs | 1m0t_A | 1.000 | 0.289 |
| 300 | 105 | 47 | 1m0w−2hgs | 1m0w_A | 1.000 | 0.010 |
| 305 | 443 | 47 | 1dfo−1bj4 | 1dfo_B | 1.000 | 0.106 |
| 305 | 443 | 47 | 1dfo−1bj4 | 1dfo_C | 1.000 | 0.096 |
| 305 | 443 | 47 | 1dfo−1bj4 | 1dfo_D | 1.000 | 0.059 |
| 309 | 210 | 46 | 1k4i−1g57 | 1k4o_A | 1.000 | 0.510 |
| 309 | 210 | 46 | 1k4i−1g57 | 1k4p_A | 1.000 | 0.166 |
| 309 | 210 | 46 | 1k4i−1g57 | 1k4l_A | 1.000 | 0.112 |
| 310 | 360 | 46 | 1bt4−1bjo | 2c0r_B | 1.000 | 0.484 |
| 310 | 360 | 46 | 1bt4−1bjo | 2c0r_A | 1.000 | 0.549 |
| 311 | 117 | 46 | 1d5y−1bl0 | 1d5y_C | 1.000 | 0.316 |
| 311 | 117 | 46 | 1d5y−1bl0 | 1d5y_D | 1.000 | 0.370 |
| 311 | 117 | 46 | 1d5y−1bl0 | 1d5y_B | 1.000 | 0.434 |
| 313 | 46 | 46 | 1bgy−1ezv | 2fyu_D | 1.000 | 0.543 |
| 313 | 46 | 46 | 1bgy−1ezv | 1ntk_D | 1.000 | 0.612 |
| 313 | 46 | 46 | 1bgy−1ezv | 1ntz_D | 1.000 | 0.570 |
| 313 | 46 | 46 | 1bgy−1ezv | 1sqx_D | 1.000 | 0.558 |
| 313 | 46 | 46 | 1bgy−1ezv | 1sqv_D | 1.000 | 0.522 |
| 313 | 46 | 46 | 1bgy−1ezv | 1pp9_Q | 1.000 | 0.412 |
| 313 | 46 | 46 | 1bgy−1ezv | 1sqp_D | 1.000 | 0.578 |
| 313 | 46 | 46 | 1bgy−1ezv | 1sqb_D | 1.000 | 0.616 |
| 313 | 46 | 46 | 1bgy−1ezv | 2a06_D | 1.000 | 0.490 |
| 313 | 46 | 46 | 1bgy−1ezv | 1be3_D | 1.000 | 0.131 |
| 313 | 46 | 46 | 1bgy−1ezv | 1nu1_D | 1.000 | 0.588 |
| 313 | 46 | 46 | 1bgy−1ezv | 1pp9_D | 1.000 | 0.439 |
| 313 | 46 | 46 | 1bgy−1ezv | 1l0n_D | 1.000 | 0.539 |
| 313 | 46 | 46 | 1bgy−1ezv | 1ntm_D | 1.000 | 0.547 |
| 313 | 46 | 46 | 1bgy−1ezv | 1ppj_Q | 1.000 | 0.546 |
| 313 | 46 | 46 | 1bgy−1ezv | 2a06_Q | 1.000 | 0.443 |
| 313 | 46 | 46 | 1bgy−1ezv | 1bgy_P | 1.000 | 0.060 |
| 313 | 46 | 46 | 1bgy−1ezv | 1sqq_D | 1.000 | 0.553 |
| 313 | 46 | 46 | 1bgy−1ezv | 1ppj_D | 1.000 | 0.535 |
| 314 | 269 | 46 | 1llw−1ea0 | 1ofd_A | 0.970 | 0.808 |
| 314 | 269 | 46 | 1llw−1ea0 | 1lm1_A | 1.000 | 0.273 |
| 314 | 269 | 46 | 1llw−1ea0 | 1ofe_B | 0.970 | 0.814 |
| 314 | 269 | 46 | 1llw−1ea0 | 1llz_A | 0.996 | 0.265 |
| 314 | 269 | 46 | 1ea0−1llw | 1ea0_B | 1.000 | 0.178 |
| 314 | 269 | 46 | 1llw−1ea0 | 1ofe_A | 0.970 | 0.819 |
| 314 | 269 | 46 | 1llw−1ea0 | 1ofd_B | 0.970 | 0.797 |
| 316 | 123 | 46 | 1ijx−1ijy | 1ijx_C | 0.992 | 0.711 |
| 316 | 123 | 46 | 1ijx−1ijy | 1ijx_F | 0.992 | 1.089 |
| 316 | 123 | 46 | 1ijy−1ijx | 1ijy_B | 1.000 | 1.138 |
| 321 | 586 | 46 | 1bvz−1sma | 1bvz_B | 0.997 | 0.406 |
| 321 | 586 | 46 | 1bvz−1sma | 1vfk_B | 0.984 | 0.997 |
| 321 | 586 | 46 | 1bvz−1sma | 1ji2_B | 0.997 | 0.676 |
| 321 | 586 | 46 | 1bvz−1sma | 1vfk_A | 0.981 | 0.993 |
| 321 | 586 | 46 | 1sma−1bvz | 1sma_B | 0.998 | 0.003 |
| 321 | 586 | 46 | 1bvz−1sma | 1ji2_A | 0.991 | 0.650 |
| 323 | 182 | 46 | 1erx−1euo | 1d3s_A | 0.989 | 0.903 |
| 323 | 182 | 46 | 1erx−1euo | 1ikj_A | 1.000 | 0.251 |
| 323 | 182 | 46 | 1erx−1euo | 2at6_X | 1.000 | 0.560 |
| 323 | 182 | 46 | 1euo−1erx | 2a3f_X | 1.000 | 0.303 |
| 323 | 182 | 46 | 1erx−1euo | 1koi_A | 1.000 | 0.181 |
| 323 | 182 | 46 | 1erx−1euo | 1ywa_A | 1.000 | 0.280 |
| 323 | 182 | 46 | 1erx−1euo | 1x8o_A | 1.000 | 0.195 |
| 323 | 182 | 46 | 1erx−1euo | 1x8p_A | 1.000 | 0.575 |
| 323 | 182 | 46 | 1erx−1euo | 1ywc_A | 1.000 | 0.557 |
| 323 | 182 | 46 | 1erx−1euo | 1d2u_A | 0.989 | 0.873 |

| n | length | %ID | refA−refB | altA | SP | RMSD |
|---|---|---|---|---|---|---|
| 323 | 182 | 46 | 1euo−1erx | 2hys_A | 0.983 | 0.900 |
| 323 | 182 | 46 | 1erx−1euo | 2at5_X | 1.000 | 0.227 |
| 323 | 182 | 46 | 1erx−1euo | 1x8q_A | 1.000 | 0.552 |
| 323 | 182 | 46 | 1euo−1erx | 2gtf_X | 0.994 | 0.872 |
| 323 | 182 | 46 | 1erx−1euo | 2at8_X | 1.000 | 0.233 |
| 323 | 182 | 46 | 1erx−1euo | 1ml7_A | 0.994 | 0.591 |
| 323 | 182 | 46 | 1euo−1erx | 1pee_A | 0.994 | 0.885 |
| 323 | 182 | 46 | 1erx−1euo | 1ike_A | 0.989 | 0.902 |
| 323 | 182 | 46 | 1erx−1euo | 1x8n_A | 1.000 | 0.491 |
| 323 | 182 | 46 | 1euo−1erx | 2acp_X | 0.989 | 0.368 |
| 323 | 182 | 46 | 1erx−1euo | 1u0x_A | 1.000 | 0.543 |
| 323 | 182 | 46 | 1euo−1erx | 2al0_X | 0.989 | 1.005 |
| 323 | 182 | 46 | 1erx−1euo | 1ywd_A | 0.989 | 0.937 |
| 323 | 182 | 46 | 1erx−1euo | 1np4_A | 0.983 | 1.069 |
| 323 | 182 | 46 | 1euo−1erx | 1t68_X | 1.000 | 0.362 |
| 323 | 182 | 46 | 1erx−1euo | 1ywb_A | 1.000 | 0.239 |
| 323 | 182 | 46 | 1erx−1euo | 1eqd_A | 0.989 | 0.875 |
| 323 | 182 | 46 | 1erx−1euo | 2ofm_X | 1.000 | 0.404 |
| 323 | 182 | 46 | 1euo−1erx | 2ah7_X | 0.983 | 0.950 |
| 323 | 182 | 46 | 1erx−1euo | 2at4_X | 1.000 | 0.592 |
| 323 | 182 | 46 | 1erx−1euo | 2at7_X | 1.000 | 0.569 |
| 324 | 321 | 46 | 6prc−1eys | 6prc_C | 1.000 | 0.006 |
| 324 | 321 | 46 | 6prc−1eys | 2prc_C | 1.000 | 0.148 |
| 324 | 321 | 46 | 6prc−1eys | 1prc_C | 1.000 | 0.252 |
| 324 | 321 | 46 | 6prc−1eys | 3prc_C | 1.000 | 0.128 |
| 324 | 321 | 46 | 6prc−1eys | 7prc_C | 1.000 | 0.151 |
| 324 | 321 | 46 | 6prc−1eys | 1dxr_C | 0.997 | 0.147 |
| 324 | 321 | 46 | 6prc−1eys | 1r2c_C | 1.000 | 0.273 |
| 324 | 321 | 46 | 6prc−1eys | 5prc_C | 1.000 | 0.136 |
| 324 | 321 | 46 | 6prc−1eys | 2i5n_C | 1.000 | 0.236 |
| 325 | 60 | 46 | 1i50−1ef4 | 2e2j_J | 1.000 | 0.393 |
| 325 | 60 | 46 | 1i50−1ef4 | 1i3q_J | 0.981 | 0.317 |
| 325 | 60 | 46 | 1i50−1ef4 | 1nik_J | 0.981 | 0.328 |
| 325 | 60 | 46 | 1i50−1ef4 | 1sfo_J | 0.981 | 0.508 |
| 325 | 60 | 46 | 1i50−1ef4 | 2nvq_J | 0.981 | 0.348 |
| 325 | 60 | 46 | 1i50−1ef4 | 1r9t_J | 0.981 | 0.514 |
| 325 | 60 | 46 | 1i50−1ef4 | 2ja7_J | 0.981 | 0.500 |
| 325 | 60 | 46 | 1i50−1ef4 | 2e2h_J | 0.981 | 0.474 |
| 325 | 60 | 46 | 1i50−1ef4 | 2ja7_V | 0.981 | 0.506 |
| 325 | 60 | 46 | 1i50−1ef4 | 2ja8_J | 0.981 | 0.504 |
| 325 | 60 | 46 | 1i50−1ef4 | 2ja5_J | 0.981 | 0.498 |
| 325 | 60 | 46 | 1i50−1ef4 | 2r7z_J | 0.981 | 0.501 |
| 325 | 60 | 46 | 1i50−1ef4 | 1y77_J | 0.981 | 0.485 |
| 325 | 60 | 46 | 1i50−1ef4 | 2ja6_J | 0.981 | 0.518 |
| 325 | 60 | 46 | 1i50−1ef4 | 2nvx_J | 0.981 | 0.417 |
| 325 | 60 | 46 | 1i50−1ef4 | 1i6h_J | 0.981 | 0.328 |
| 325 | 60 | 46 | 1i50−1ef4 | 2b63_J | 0.981 | 0.550 |
| 325 | 60 | 46 | 1i50−1ef4 | 1y1v_J | 0.981 | 0.480 |
| 325 | 60 | 46 | 1i50−1ef4 | 2nvt_J | 0.981 | 0.460 |
| 325 | 60 | 46 | 1i50−1ef4 | 1r9s_J | 0.981 | 0.329 |
| 325 | 60 | 46 | 1i50−1ef4 | 2r92_J | 0.981 | 0.491 |
| 325 | 60 | 46 | 1i50−1ef4 | 2b8k_J | 0.981 | 0.497 |
| 325 | 60 | 46 | 1i50−1ef4 | 1k83_J | 1.000 | 0.137 |
| 325 | 60 | 46 | 1i50−1ef4 | 1r5u_J | 0.981 | 0.329 |
| 325 | 60 | 46 | 1i50−1ef4 | 2nvy_J | 0.981 | 0.388 |
| 325 | 60 | 46 | 1i50−1ef4 | 1wcm_J | 0.981 | 0.498 |
| 325 | 60 | 46 | 1i50−1ef4 | 2e2i_J | 1.000 | 0.433 |
| 325 | 60 | 46 | 1i50−1ef4 | 1y1w_J | 0.981 | 0.483 |
| 325 | 60 | 46 | 1i50−1ef4 | 2nvz_J | 0.981 | 0.512 |
| 325 | 60 | 46 | 1i50−1ef4 | 1twf_J | 1.000 | 0.239 |
| 325 | 60 | 46 | 1i50−1ef4 | 2r93_J | 0.981 | 0.490 |
| 325 | 60 | 46 | 1i50−1ef4 | 2yu9_J | 0.981 | 0.510 |
| 326 | 88 | 46 | 1lfd−1rlf | 1lfd_C | 0.778 | 0.547 |
| 334 | 389 | 45 | 2scu−1eud | 2nu8_E | 1.000 | 0.799 |
| 334 | 389 | 45 | 2scu−1eud | 2nu9_B | 1.000 | 0.459 |
| 334 | 389 | 45 | 2scu−1eud | 1scu_B | 1.000 | 0.333 |
| 334 | 389 | 45 | 2scu−1eud | 2nu8_B | 1.000 | 0.700 |
| 334 | 389 | 45 | 2scu−1eud | 2scu_E | 1.000 | 0.348 |
| 334 | 389 | 45 | 2scu−1eud | 2nu6_B | 1.000 | 0.624 |
| 334 | 389 | 45 | 2scu−1eud | 2nu7_B | 1.000 | 0.680 |
| 334 | 389 | 45 | 2scu−1eud | 1jkj_B | 1.000 | 0.625 |

| n | length | %ID | refA−refB | altA | SP | RMSD |
|---|---|---|---|---|---|---|
| 334 | 389 | 45 | 2scu−1eud | 2nu6_E | 1.000 | 0.720 |
| 334 | 389 | 45 | 2scu−1eud | 2nu9_G | 1.000 | 0.464 |
| 334 | 389 | 45 | 2scu−1eud | 2nua_E | 1.000 | 0.582 |
| 334 | 389 | 45 | 2scu−1eud | 1scu_E | 1.000 | 0.412 |
| 334 | 389 | 45 | 2scu−1eud | 2nu7_E | 1.000 | 0.774 |
| 334 | 389 | 45 | 2scu−1eud | 2nu9_I | 1.000 | 0.464 |
| 334 | 389 | 45 | 2scu−1eud | 2nu9_E | 1.000 | 0.466 |
| 334 | 389 | 45 | 2scu−1eud | 2nua_B | 1.000 | 0.576 |
| 334 | 389 | 45 | 2scu−1eud | 1jkj_E | 1.000 | 0.719 |
| 341 | 232 | 45 | 1aq6−1zrn | 1aq6_B | 1.000 | 0.300 |
| 341 | 232 | 45 | 1zrn−1aq6 | 1zrm_A | 1.000 | 0.526 |
| 349 | 114 | 45 | 1b43−1a77 | 1b43_B | 0.907 | 0.868 |
| 349 | 114 | 45 | 1a77−1b43 | 1a76_A | 1.000 | 0.245 |
| 355 | 172 | 44 | 1kij−1ei1 | 1kij_B | 1.000 | 1.010 |
| 356 | 155 | 44 | 1iqc−1eb7 | 1iqc_D | 1.000 | 0.303 |
| 356 | 155 | 44 | 1iqc−1eb7 | 1iqc_B | 1.000 | 0.327 |
| 356 | 155 | 44 | 1iqc−1eb7 | 1iqc_C | 1.000 | 0.211 |
| 359 | 275 | 44 | 1k4v−1lzj | 1gww_A | 1.000 | 0.148 |
| 359 | 275 | 44 | 1k4v−1lzj | 1gww_B | 1.000 | 0.179 |
| 359 | 275 | 44 | 1k4v−1lzj | 1gx4_A | 0.996 | 0.249 |
| 359 | 275 | 44 | 1lzj−1k4v | 1lzj_A | 1.000 | 0.016 |
| 359 | 275 | 44 | 1k4v−1lzj | 1gx0_A | 1.000 | 0.123 |
| 359 | 275 | 44 | 1k4v−1lzj | 1gwv_A | 1.000 | 0.179 |
| 359 | 275 | 44 | 1k4v−1lzj | 1o7q_B | 1.000 | 0.182 |
| 359 | 275 | 44 | 1k4v−1lzj | 1gx0_B | 1.000 | 0.152 |
| 359 | 275 | 44 | 1k4v−1lzj | 1o7q_A | 1.000 | 0.143 |
| 359 | 275 | 44 | 1k4v−1lzj | 1gwv_B | 0.996 | 0.209 |
| 359 | 275 | 44 | 1k4v−1lzj | 1gx4_B | 1.000 | 0.278 |
| 360 | 392 | 44 | 1feh−1hfe | 1c4c_A | 0.989 | 0.348 |
| 360 | 392 | 44 | 1hfe−1feh | 1hfe_L | 1.000 | 0.019 |
| 360 | 392 | 44 | 1hfe−1feh | 1hfe_M | 0.995 | 0.149 |
| 360 | 392 | 44 | 1feh−1hfe | 1c4a_A | 0.984 | 0.407 |
| 361 | 312 | 44 | 1b04−1dgt | 1b04_B | 0.912 | 1.430 |
| 367 | 74 | 44 | 1dzf−1eik | 1y1v_E | 0.930 | 2.449 |
| 367 | 74 | 44 | 1dzf−1eik | 2nvz_E | 0.930 | 2.358 |
| 367 | 74 | 44 | 1dzf−1eik | 1i6h_E | 0.930 | 2.371 |
| 367 | 74 | 44 | 1dzf−1eik | 1nik_E | 0.930 | 2.371 |
| 367 | 74 | 44 | 1dzf−1eik | 2ja7_E | 0.930 | 2.439 |
| 367 | 74 | 44 | 1dzf−1eik | 2nvt_E | 0.930 | 2.465 |
| 367 | 74 | 44 | 1dzf−1eik | 1r9t_E | 0.930 | 2.418 |
| 367 | 74 | 44 | 1dzf−1eik | 2ja6_E | 0.930 | 2.430 |
| 367 | 74 | 44 | 1dzf−1eik | 2r92_E | 0.930 | 2.445 |
| 367 | 74 | 44 | 1dzf−1eik | 1r9s_E | 0.930 | 2.371 |
| 367 | 74 | 44 | 1dzf−1eik | 1y77_E | 0.930 | 2.430 |
| 367 | 74 | 44 | 1dzf−1eik | 1k83_E | 0.930 | 2.352 |
| 367 | 74 | 44 | 1dzf−1eik | 2r7z_E | 0.930 | 2.429 |
| 367 | 74 | 44 | 1dzf−1eik | 2ja7_Q | 0.930 | 2.434 |
| 367 | 74 | 44 | 1dzf−1eik | 2e2j_E | 0.930 | 2.349 |
| 367 | 74 | 44 | 1dzf−1eik | 2ja5_E | 0.930 | 2.434 |
| 367 | 74 | 44 | 1dzf−1eik | 1i3q_E | 0.930 | 2.398 |
| 367 | 74 | 44 | 1dzf−1eik | 1twa_E | 0.930 | 2.382 |
| 367 | 74 | 44 | 1dzf−1eik | 2e2h_E | 0.930 | 2.365 |
| 367 | 74 | 44 | 1dzf−1eik | 2r93_E | 0.930 | 2.422 |
| 367 | 74 | 44 | 1dzf−1eik | 1twc_E | 0.930 | 2.380 |
| 367 | 74 | 44 | 1dzf−1eik | 1y1w_E | 0.930 | 2.421 |
| 367 | 74 | 44 | 1dzf−1eik | 2nvy_E | 0.930 | 2.316 |
| 367 | 74 | 44 | 1dzf−1eik | 1wcm_E | 0.930 | 2.430 |
| 367 | 74 | 44 | 1dzf−1eik | 2nvx_E | 0.930 | 2.408 |
| 367 | 74 | 44 | 1dzf−1eik | 1sfo_E | 0.930 | 2.413 |
| 367 | 74 | 44 | 1dzf−1eik | 2b8k_E | 0.930 | 2.430 |
| 367 | 74 | 44 | 1dzf−1eik | 1i50_E | 0.930 | 2.369 |
| 367 | 74 | 44 | 1dzf−1eik | 1twf_E | 0.930 | 2.390 |
| 367 | 74 | 44 | 1dzf−1eik | 2e2i_E | 0.930 | 2.405 |
| 367 | 74 | 44 | 1dzf−1eik | 2b63_E | 0.930 | 2.429 |
| 367 | 74 | 44 | 1dzf−1eik | 1r5u_E | 0.930 | 2.371 |
| 367 | 74 | 44 | 1dzf−1eik | 1twh_E | 0.930 | 2.383 |
| 367 | 74 | 44 | 1dzf−1eik | 1twg_E | 0.930 | 2.396 |
| 367 | 74 | 44 | 1dzf−1eik | 2yu9_E | 0.930 | 2.401 |
| 367 | 74 | 44 | 1dzf−1eik | 2ja8_E | 0.930 | 2.443 |
| 367 | 74 | 44 | 1dzf−1eik | 2nvq_E | 0.930 | 2.414 |
| 368 | 137 | 44 | 1jj2−1rl2 | 1k9m_C | 1.000 | 0.078 |

n: internal numbering; length of alignment; % sequence ID (cf. Homstrad); refA−refB: reference alignment; altA: alternative for struc. A; SP: sum−of−pairs score of alt. alignment w/r reference

| n | length | %ID | refA−refB | altA | SP | RMSD |
|---|---|---|---|---|---|---|
| 368 | 137 | 44 | 1jj2−1rl2 | 1kc8_C | 1.000 | 0.082 |
| 368 | 137 | 44 | 1jj2−1rl2 | 1m1k_C | 1.000 | 0.075 |
| 368 | 137 | 44 | 1jj2−1rl2 | 1q81_C | 1.000 | 0.149 |
| 368 | 137 | 44 | 1jj2−1rl2 | 1w2b_A | 1.000 | 0.276 |
| 368 | 137 | 44 | 1jj2−1rl2 | 1qvg_A | 1.000 | 0.116 |
| 368 | 137 | 44 | 1jj2−1rl2 | 1kd1_C | 1.000 | 0.080 |
| 368 | 137 | 44 | 1jj2−1rl2 | 1kqs_A | 1.000 | 0.106 |
| 368 | 137 | 44 | 1jj2−1rl2 | 1nji_C | 1.000 | 0.056 |
| 368 | 137 | 44 | 1jj2−1rl2 | 1k73_C | 1.000 | 0.037 |
| 368 | 137 | 44 | 1jj2−1rl2 | 1q82_C | 1.000 | 0.104 |
| 368 | 137 | 44 | 1jj2−1rl2 | 1m90_C | 1.000 | 0.068 |
| 368 | 137 | 44 | 1jj2−1rl2 | 1n8r_C | 1.000 | 0.101 |
| 368 | 137 | 44 | 1jj2−1rl2 | 1q7y_C | 1.000 | 0.130 |
| 368 | 137 | 44 | 1jj2−1rl2 | 1qvf_A | 1.000 | 0.050 |
| 368 | 137 | 44 | 1jj2−1rl2 | 1k8a_C | 1.000 | 0.076 |
| 368 | 137 | 44 | 1jj2−1rl2 | 1q86_C | 1.000 | 0.130 |
| 368 | 137 | 44 | 1rl2−1jj2 | 1rl2_B | 1.000 | 1.019 |
| 377 | 303 | 43 | 2cev−1d3v | 5cev_B | 1.000 | 0.445 |
| 377 | 303 | 43 | 1d3v−2cev | 1hqf_A | 1.000 | 0.486 |
| 377 | 303 | 43 | 2cev−1d3v | 1cev_E | 0.983 | 0.953 |
| 377 | 303 | 43 | 1d3v−2cev | 1hqf_C | 1.000 | 0.486 |
| 377 | 303 | 43 | 2cev−1d3v | 5cev_A | 1.000 | 0.189 |
| 377 | 303 | 43 | 2cev−1d3v | 5cev_C | 1.000 | 0.360 |
| 377 | 303 | 43 | 1d3v−2cev | 1hq5_B | 1.000 | 0.319 |
| 377 | 303 | 43 | 2cev−1d3v | 4cev_E | 1.000 | 0.353 |
| 377 | 303 | 43 | 1d3v−2cev | 1hqh_A | 1.000 | 0.452 |
| 377 | 303 | 43 | 1d3v−2cev | 1hq5_A | 1.000 | 0.318 |
| 377 | 303 | 43 | 2cev−1d3v | 1cev_B | 0.980 | 0.953 |
| 377 | 303 | 43 | 2cev−1d3v | 3cev_D | 1.000 | 0.399 |
| 377 | 303 | 43 | 2cev−1d3v | 4cev_C | 1.000 | 0.376 |
| 377 | 303 | 43 | 2cev−1d3v | 3cev_A | 1.000 | 0.366 |
| 377 | 303 | 43 | 2cev−1d3v | 5cev_D | 1.000 | 0.377 |
| 377 | 303 | 43 | 2cev−1d3v | 5cev_F | 1.000 | 0.339 |
| 377 | 303 | 43 | 2cev−1d3v | 2cev_F | 1.000 | 0.232 |
| 377 | 303 | 43 | 1d3v−2cev | 1hqh_C | 1.000 | 0.452 |
| 377 | 303 | 43 | 2cev−1d3v | 3cev_C | 1.000 | 0.353 |
| 377 | 303 | 43 | 2cev−1d3v | 4cev_B | 0.983 | 0.457 |
| 377 | 303 | 43 | 2cev−1d3v | 1cev_C | 0.962 | 0.953 |
| 377 | 303 | 43 | 1d3v−2cev | 1rla_A | 1.000 | 0.322 |
| 377 | 303 | 43 | 2cev−1d3v | 3cev_F | 1.000 | 0.417 |
| 377 | 303 | 43 | 1d3v−2cev | 1d3v_B | 1.000 | 0.099 |
| 377 | 303 | 43 | 2cev−1d3v | 1cev_F | 0.983 | 0.931 |
| 377 | 303 | 43 | 1d3v−2cev | 1hqh_B | 1.000 | 0.452 |
| 377 | 303 | 43 | 2cev−1d3v | 2cev_C | 1.000 | 0.345 |
| 377 | 303 | 43 | 2cev−1d3v | 5cev_E | 1.000 | 0.303 |
| 377 | 303 | 43 | 2cev−1d3v | 3cev_B | 1.000 | 0.478 |
| 377 | 303 | 43 | 2cev−1d3v | 4cev_A | 1.000 | 0.417 |
| 377 | 303 | 43 | 1d3v−2cev | 1r1o_A | 1.000 | 0.367 |
| 377 | 303 | 43 | 2cev−1d3v | 1cev_D | 0.973 | 0.936 |
| 377 | 303 | 43 | 2cev−1d3v | 2cev_D | 1.000 | 0.348 |
| 377 | 303 | 43 | 2cev−1d3v | 4cev_D | 1.000 | 0.393 |
| 377 | 303 | 43 | 1d3v−2cev | 1r1o_B | 1.000 | 0.365 |
| 377 | 303 | 43 | 2cev−1d3v | 3cev_E | 1.000 | 0.353 |
| 377 | 303 | 43 | 2cev−1d3v | 1cev_A | 0.962 | 0.950 |
| 377 | 303 | 43 | 2cev−1d3v | 2cev_B | 1.000 | 0.427 |
| 377 | 303 | 43 | 1d3v−2cev | 1hqf_B | 1.000 | 0.486 |
| 377 | 303 | 43 | 2cev−1d3v | 2cev_E | 1.000 | 0.279 |
| 377 | 303 | 43 | 2cev−1d3v | 4cev_F | 1.000 | 0.419 |
| 377 | 303 | 43 | 1d3v−2cev | 1rla_C | 1.000 | 0.319 |
| 377 | 303 | 43 | 1d3v−2cev | 1rla_B | 1.000 | 0.323 |
| 377 | 303 | 43 | 1d3v−2cev | 1r1o_C | 1.000 | 0.369 |
| 380 | 98 | 43 | 1aon−1p3h | 1aon_Q | 1.000 | 0.162 |
| 380 | 98 | 43 | 1aon−1p3h | 1aon_R | 1.000 | 0.164 |
| 380 | 98 | 43 | 1aon−1p3h | 1pf9_U | 0.872 | 0.359 |
| 380 | 98 | 43 | 1aon−1p3h | 1gru_U | 1.000 | 0.166 |
| 380 | 98 | 43 | 1aon−1p3h | 1pcq_R | 0.936 | 0.521 |
| 380 | 98 | 43 | 1aon−1p3h | 1sx4_O | 0.936 | 0.463 |
| 380 | 98 | 43 | 1aon−1p3h | 1aon_T | 1.000 | 0.176 |
| 380 | 98 | 43 | 1aon−1p3h | 1gru_T | 1.000 | 0.176 |
| 380 | 98 | 43 | 1aon−1p3h | 1svt_Q | 0.883 | 0.601 |
| 380 | 98 | 43 | 1aon−1p3h | 1svt_S | 0.872 | 0.600 |

| n | length | %ID | refA−refB | altA | SP | RMSD |
|---|---|---|---|---|---|---|
| 380 | 98 | 43 | 1aon−1p3h | 1svt_T | 0.872 | 0.601 |
| 380 | 98 | 43 | 1aon−1p3h | 1pf9_S | 0.936 | 0.359 |
| 380 | 98 | 43 | 1aon−1p3h | 1pcq_P | 0.872 | 0.521 |
| 380 | 98 | 43 | 1aon−1p3h | 1pf9_R | 0.872 | 0.359 |
| 380 | 98 | 43 | 1aon−1p3h | 1aon_P | 1.000 | 0.178 |
| 380 | 98 | 43 | 1aon−1p3h | 1sx4_P | 0.936 | 0.465 |
| 380 | 98 | 43 | 1aon−1p3h | 1gru_R | 1.000 | 0.164 |
| 380 | 98 | 43 | 1aon−1p3h | 1sx4_U | 0.936 | 0.467 |
| 380 | 98 | 43 | 1aon−1p3h | 1gru_Q | 1.000 | 0.162 |
| 380 | 98 | 43 | 1aon−1p3h | 1pf9_Q | 0.936 | 0.359 |
| 380 | 98 | 43 | 1aon−1p3h | 1pcq_Q | 0.872 | 0.522 |
| 380 | 98 | 43 | 1aon−1p3h | 1svt_R | 0.872 | 0.600 |
| 380 | 98 | 43 | 1aon−1p3h | 1aon_U | 1.000 | 0.166 |
| 380 | 98 | 43 | 1aon−1p3h | 1pcq_O | 0.883 | 0.521 |
| 380 | 98 | 43 | 1aon−1p3h | 1pf9_T | 1.000 | 0.359 |
| 380 | 98 | 43 | 1aon−1p3h | 1gru_P | 1.000 | 0.178 |
| 380 | 98 | 43 | 1aon−1p3h | 1pf9_O | 0.936 | 0.359 |
| 380 | 98 | 43 | 1aon−1p3h | 1svt_P | 0.872 | 0.599 |
| 380 | 98 | 43 | 1aon−1p3h | 1aon_S | 1.000 | 0.160 |
| 380 | 98 | 43 | 1aon−1p3h | 1pcq_U | 0.872 | 0.521 |
| 380 | 98 | 43 | 1aon−1p3h | 1pf9_P | 0.936 | 0.361 |
| 380 | 98 | 43 | 1aon−1p3h | 1svt_U | 0.872 | 0.598 |
| 380 | 98 | 43 | 1aon−1p3h | 1sx4_R | 0.936 | 0.462 |
| 380 | 98 | 43 | 1aon−1p3h | 1pcq_T | 0.883 | 0.521 |
| 380 | 98 | 43 | 1aon−1p3h | 1gru_S | 1.000 | 0.160 |
| 380 | 98 | 43 | 1aon−1p3h | 1svt_O | 0.872 | 0.599 |
| 380 | 98 | 43 | 1aon−1p3h | 1pcq_S | 0.883 | 0.522 |
| 380 | 98 | 43 | 1aon−1p3h | 1sx4_Q | 0.936 | 0.460 |
| 380 | 98 | 43 | 1aon−1p3h | 1sx4_S | 0.936 | 0.459 |
| 380 | 98 | 43 | 1aon−1p3h | 1sx4_T | 0.936 | 0.460 |
| 381 | 143 | 43 | 1d0i−2dhq | 1d0i_K | 1.000 | 0.125 |
| 381 | 143 | 43 | 1d0i−2dhq | 1d0i_B | 1.000 | 0.215 |
| 381 | 143 | 43 | 1d0i−2dhq | 1d0i_D | 1.000 | 0.227 |
| 381 | 143 | 43 | 1d0i−2dhq | 1d0i_H | 1.000 | 0.216 |
| 381 | 143 | 43 | 1d0i−2dhq | 1d0i_E | 1.000 | 0.264 |
| 381 | 143 | 43 | 1d0i−2dhq | 1d0i_G | 1.000 | 0.251 |
| 381 | 143 | 43 | 1d0i−2dhq | 1d0i_I | 1.000 | 0.375 |
| 381 | 143 | 43 | 1d0i−2dhq | 1d0i_L | 1.000 | 0.217 |
| 381 | 143 | 43 | 1d0i−2dhq | 1d0i_F | 1.000 | 0.134 |
| 381 | 143 | 43 | 1d0i−2dhq | 1d0i_J | 1.000 | 0.210 |
| 381 | 143 | 43 | 1d0i−2dhq | 1d0i_C | 1.000 | 0.189 |
| 382 | 422 | 43 | 1llw−1ea0 | 1llz_A | 1.000 | 0.255 |
| 382 | 422 | 43 | 1llw−1ea0 | 1lm1_A | 1.000 | 0.243 |
| 382 | 422 | 43 | 1llw−1ea0 | 1ofe_A | 0.959 | 0.978 |
| 382 | 422 | 43 | 1llw−1ea0 | 1ofd_A | 0.959 | 0.904 |
| 382 | 422 | 43 | 1ea0−1llw | 1ea0_B | 0.995 | 0.169 |
| 382 | 422 | 43 | 1llw−1ea0 | 1ofd_B | 0.959 | 0.903 |
| 382 | 422 | 43 | 1llw−1ea0 | 1ofe_B | 0.959 | 0.990 |
| 384 | 94 | 43 | 1hkt−3hts | 1hks_A | 0.951 | 2.330 |
| 393 | 73 | 43 | 1b4f−1b0x | 1b4f_D | 0.944 | 5.532 |
| 393 | 73 | 43 | 1b4f−1b0x | 1b4f_E | 1.000 | 0.383 |
| 393 | 73 | 43 | 1b4f−1b0x | 1b4f_A | 1.000 | 0.015 |
| 393 | 73 | 43 | 1b4f−1b0x | 1b4f_F | 0.944 | 5.562 |
| 393 | 73 | 43 | 1b4f−1b0x | 1b4f_H | 0.944 | 5.397 |
| 393 | 73 | 43 | 1b4f−1b0x | 1b4f_B | 0.944 | 5.336 |
| 393 | 73 | 43 | 1b4f−1b0x | 1b4f_C | 1.000 | 0.346 |
| 394 | 436 | 43 | 2tpt−1brw | 1azy_A | 0.998 | 1.076 |
| 394 | 436 | 43 | 2tpt−1brw | 1otp_A | 1.000 | 1.099 |
| 394 | 436 | 43 | 2tpt−1brw | 1azy_B | 0.998 | 1.081 |
| 395 | 181 | 43 | 1br9−1uea | 1gxd_C | 0.948 | 1.962 |
| 395 | 181 | 43 | 1br9−1uea | 1gxd_D | 0.977 | 1.342 |
| 395 | 181 | 43 | 1uea−1br9 | 1uea_D | 0.965 | 1.101 |
| 400 | 324 | 42 | 1gyt−1lam | 1gyt_D | 0.997 | 0.284 |
| 400 | 324 | 42 | 1gyt−1lam | 1gyt_E | 0.997 | 0.307 |
| 400 | 324 | 42 | 1gyt−1lam | 1gyt_H | 0.997 | 0.341 |
| 400 | 324 | 42 | 1lam−1gyt | 1lan_A | 1.000 | 0.132 |
| 400 | 324 | 42 | 1lam−1gyt | 1lcp_B | 1.000 | 0.178 |
| 400 | 324 | 42 | 1gyt−1lam | 1gyt_F | 1.000 | 0.253 |
| 400 | 324 | 42 | 1gyt−1lam | 1gyt_G | 1.000 | 0.301 |
| 400 | 324 | 42 | 1gyt−1lam | 1gyt_K | 1.000 | 0.233 |
| 400 | 324 | 42 | 1gyt−1lam | 1gyt_B | 1.000 | 0.285 |

| n | length | %ID | refA−refB | altA | SP | RMSD |
|---|---|---|---|---|---|---|
| 400 | 324 | 42 | 1gyt−1lam | 1gyt_I | 0.997 | 0.322 |
| 400 | 324 | 42 | 1gyt−1lam | 1gyt_J | 1.000 | 0.291 |
| 400 | 324 | 42 | 1gyt−1lam | 1gyt_L | 1.000 | 0.350 |
| 400 | 324 | 42 | 1gyt−1lam | 1gyt_C | 1.000 | 0.263 |
| 400 | 324 | 42 | 1lam−1gyt | 1lcp_A | 0.997 | 0.154 |
| 402 | 159 | 42 | 1czt−1d7p | 1czs_A | 1.000 | 0.491 |
| 405 | 482 | 42 | 1fo3−1dl2 | 1fo2_A | 1.000 | 0.193 |
| 406 | 689 | 42 | 1k9d−1gqi | 1k9d_A | 1.000 | 0.013 |
| 406 | 689 | 42 | 1gqi−1k9d | 1gqj_B | 0.695 | 0.212 |
| 406 | 689 | 42 | 1gqi−1k9d | 1gqk_B | 0.977 | 0.228 |
| 406 | 689 | 42 | 1gqi−1k9d | 1gqj_A | 0.705 | 0.115 |
| 406 | 689 | 42 | 1gqi−1k9d | 1gqi_B | 0.994 | 0.206 |
| 406 | 689 | 42 | 1gqi−1k9d | 1gqk_A | 0.995 | 0.133 |
| 408 | 74 | 42 | 1hst−1ghc | 1hst_B | 0.973 | 2.694 |
| 412 | 286 | 42 | 1qpo−1qap | 1qpr_B | 1.000 | 0.520 |
| 412 | 286 | 42 | 1qpo−1qap | 1qpr_E | 1.000 | 0.717 |
| 412 | 286 | 42 | 1qpo−1qap | 1qpr_A | 1.000 | 0.546 |
| 412 | 286 | 42 | 1qpo−1qap | 1qpr_D | 1.000 | 0.578 |
| 412 | 286 | 42 | 1qpo−1qap | 1qpn_A | 1.000 | 0.176 |
| 412 | 286 | 42 | 1qpo−1qap | 1qpq_A | 1.000 | 0.540 |
| 412 | 286 | 42 | 1qpo−1qap | 1qpr_C | 1.000 | 0.640 |
| 412 | 286 | 42 | 1qap−1qpo | 1qap_B | 1.000 | 0.729 |
| 412 | 286 | 42 | 1qpo−1qap | 1qpr_F | 1.000 | 0.627 |
| 413 | 127 | 42 | 1jj2−1whi | 1k9m_L | 1.000 | 0.078 |
| 413 | 127 | 42 | 1jj2−1whi | 1kc8_L | 1.000 | 0.065 |
| 413 | 127 | 42 | 1jj2−1whi | 1m1k_L | 1.000 | 0.090 |
| 413 | 127 | 42 | 1jj2−1whi | 1q81_L | 1.000 | 0.152 |
| 413 | 127 | 42 | 1jj2−1whi | 1w2b_J | 1.000 | 0.220 |
| 413 | 127 | 42 | 1jj2−1whi | 1qvg_J | 1.000 | 0.131 |
| 413 | 127 | 42 | 1jj2−1whi | 1kd1_L | 1.000 | 0.079 |
| 413 | 127 | 42 | 1jj2−1whi | 1kqs_J | 1.000 | 0.087 |
| 413 | 127 | 42 | 1jj2−1whi | 1nji_L | 1.000 | 0.053 |
| 413 | 127 | 42 | 1jj2−1whi | 1k73_L | 1.000 | 0.038 |
| 413 | 127 | 42 | 1jj2−1whi | 1q82_L | 1.000 | 0.094 |
| 413 | 127 | 42 | 1whi−1jj2 | 1c04_D | 0.992 | 1.068 |
| 413 | 127 | 42 | 1jj2−1whi | 1m90_L | 1.000 | 0.063 |
| 413 | 127 | 42 | 1jj2−1whi | 1n8r_L | 1.000 | 0.097 |
| 413 | 127 | 42 | 1jj2−1whi | 1q7y_L | 1.000 | 0.155 |
| 413 | 127 | 42 | 1jj2−1whi | 1qvf_J | 1.000 | 0.057 |
| 413 | 127 | 42 | 1jj2−1whi | 1k8a_L | 1.000 | 0.081 |
| 413 | 127 | 42 | 1jj2−1whi | 1q86_L | 1.000 | 0.111 |
| 413 | 127 | 42 | 1whi−1jj2 | 1yl3_N | 0.992 | 0.928 |
| 413 | 127 | 42 | 1whi−1jj2 | 2b66_O | 0.992 | 0.921 |
| 413 | 127 | 42 | 1whi−1jj2 | 2b9n_O | 0.992 | 0.921 |
| 413 | 127 | 42 | 1whi−1jj2 | 2b9p_O | 0.992 | 0.921 |
| 413 | 127 | 42 | 1whi−1jj2 | 487d_M | 0.992 | 1.068 |
| 414 | 260 | 42 | 1mvh−1ml9 | 1mvx_A | 1.000 | 0.113 |
| 416 | 55 | 41 | 1l1c−1auu | 1l1c_B | 0.963 | 0.668 |
| 419 | 176 | 41 | 1cd3−1gff | 1al0_G | 1.000 | 0.265 |
| 419 | 176 | 41 | 1cd3−1gff | 2bpa_2 | 1.000 | 0.379 |
| 420 | 282 | 41 | 1e3m−1ewq | 1e3m_B | 0.996 | 1.382 |
| 423 | 69 | 41 | 1jj2−1rl2 | 1k9m_C | 1.000 | 0.078 |
| 423 | 69 | 41 | 1jj2−1rl2 | 1kc8_C | 1.000 | 0.085 |
| 423 | 69 | 41 | 1jj2−1rl2 | 1m1k_C | 1.000 | 0.076 |
| 423 | 69 | 41 | 1rl2−1jj2 | 1c04_A | 0.921 | 1.143 |
| 423 | 69 | 41 | 1jj2−1rl2 | 1q81_C | 1.000 | 0.146 |
| 423 | 69 | 41 | 1jj2−1rl2 | 1w2b_A | 1.000 | 0.290 |
| 423 | 69 | 41 | 1jj2−1rl2 | 1qvg_A | 1.000 | 0.114 |
| 423 | 69 | 41 | 1jj2−1rl2 | 1kd1_C | 1.000 | 0.081 |
| 423 | 69 | 41 | 1jj2−1rl2 | 1kqs_A | 1.000 | 0.114 |
| 423 | 69 | 41 | 1jj2−1rl2 | 1nji_C | 1.000 | 0.055 |
| 423 | 69 | 41 | 1jj2−1rl2 | 1k73_C | 1.000 | 0.037 |
| 423 | 69 | 41 | 1jj2−1rl2 | 1q82_C | 1.000 | 0.111 |
| 423 | 69 | 41 | 1jj2−1rl2 | 1m90_C | 1.000 | 0.065 |
| 423 | 69 | 41 | 1jj2−1rl2 | 1n8r_C | 1.000 | 0.105 |
| 423 | 69 | 41 | 1jj2−1rl2 | 1q7y_C | 1.000 | 0.128 |
| 423 | 69 | 41 | 1jj2−1rl2 | 1qvf_A | 1.000 | 0.053 |
| 423 | 69 | 41 | 1jj2−1rl2 | 1k8a_C | 1.000 | 0.080 |
| 423 | 69 | 41 | 1rl2−1jj2 | 1rl2_B | 0.921 | 1.175 |
| 423 | 69 | 41 | 1jj2−1rl2 | 1q86_C | 1.000 | 0.123 |
| 427 | 608 | 40 | 1b25−1aor | 1b25_B | 0.988 | 0.128 |

n: internal numbering; length of alignment; % sequence ID (cf. Homstrad); refA−refB: reference alignment; altA: alternative for struc. A; SP: sum−of−pairs score of alt. alignment w/r reference

| n | length | %ID | refA−refB | altA | SP | RMSD |
|---|---|---|---|---|---|---|
| 427 | 608 | 40 | 1b25−1aor | 1b25_D | 1.000 | 0.136 |
| 427 | 608 | 40 | 1b25−1aor | 1b4n_D | 1.000 | 0.191 |
| 427 | 608 | 40 | 1b25−1aor | 1b25_C | 1.000 | 0.128 |
| 427 | 608 | 40 | 1b25−1aor | 1b4n_A | 0.998 | 0.175 |
| 427 | 608 | 40 | 1b25−1aor | 1b4n_C | 1.000 | 0.181 |
| 427 | 608 | 40 | 1aor−1b25 | 1aor_B | 1.000 | 0.171 |
| 427 | 608 | 40 | 1b25−1aor | 1b4n_B | 1.000 | 0.184 |
| 430 | 184 | 40 | 1fvk−1bed | 1a2m_B | 1.000 | 0.466 |
| 430 | 184 | 40 | 1fvk−1bed | 1dsb_A | 1.000 | 0.154 |
| 430 | 184 | 40 | 1fvk−1bed | 1fvk_B | 1.000 | 0.912 |
| 430 | 184 | 40 | 1fvk−1bed | 1a23_A | 0.983 | 2.302 |
| 430 | 184 | 40 | 1fvk−1bed | 1dsb_B | 1.000 | 0.929 |
| 430 | 184 | 40 | 1fvk−1bed | 1a2j_A | 1.000 | 0.932 |
| 430 | 184 | 40 | 1fvk−1bed | 1a2m_A | 1.000 | 0.652 |
| 435 | 470 | 40 | 1f8m−1dqu | 1f8m_B | 0.997 | 0.195 |
| 435 | 470 | 40 | 1f8m−1dqu | 1f8m_D | 1.000 | 0.180 |
| 435 | 470 | 40 | 1f8m−1dqu | 1f8m_C | 1.000 | 0.207 |
| 438 | 452 | 40 | 1cb5−1gcb | 1cb5_B | 1.000 | 0.001 |
| 448 | 354 | 39 | 1bwd−1jdw | 1bwd_B | 0.994 | 0.199 |
| 448 | 354 | 39 | 1jdw−1bwd | 2jdw_A | 0.994 | 0.093 |
| 448 | 354 | 39 | 1jdw−1bwd | 3jdw_A | 0.980 | 0.809 |
| 453 | 148 | 39 | 1m6b−1igr | 1m6b_B | 0.833 | 0.752 |
| 458 | 72 | 39 | 1tnt−1qpm | 1tns_A | 0.986 | 3.285 |
| 462 | 118 | 39 | 1dlw−1dly | 1uvy_A | 1.000 | 0.278 |
| 465 | 201 | 39 | 1n1b−5eau | 1n1b_B | 1.000 | 0.293 |
| 470 | 472 | 38 | 1dnp−1qnf | 1dnp_B | 1.000 | 0.110 |
| 473 | 273 | 38 | 1ajz−1ad1 | 1aj0_A | 1.000 | 0.245 |
| 473 | 273 | 38 | 1ajz−1ad1 | 1aj2_A | 1.000 | 0.370 |
| 474 | 126 | 38 | 1g51−1c0a | 1l0w_A | 0.992 | 0.953 |
| 474 | 126 | 38 | 1g51−1c0a | 1efw_A | 0.992 | 0.929 |
| 474 | 126 | 38 | 1g51−1c0a | 1efw_B | 0.992 | 0.812 |
| 481 | 159 | 38 | 1jj2−1iq4 | 1kd1_F | 1.000 | 0.062 |
| 481 | 159 | 38 | 1jj2−1iq4 | 1m1k_F | 1.000 | 0.065 |
| 481 | 159 | 38 | 1jj2−1iq4 | 1nji_F | 1.000 | 0.039 |
| 481 | 159 | 38 | 1jj2−1iq4 | 1w2b_D | 1.000 | 0.223 |
| 481 | 159 | 38 | 1jj2−1iq4 | 1q86_F | 1.000 | 0.103 |
| 481 | 159 | 38 | 1jj2−1iq4 | 1kc8_F | 1.000 | 0.045 |
| 481 | 159 | 38 | 1jj2−1iq4 | 1m90_F | 1.000 | 0.042 |
| 481 | 159 | 38 | 1jj2−1iq4 | 1n8r_F | 1.000 | 0.064 |
| 481 | 159 | 38 | 1jj2−1iq4 | 1q82_F | 1.000 | 0.065 |
| 481 | 159 | 38 | 1jj2−1iq4 | 1k8a_F | 1.000 | 0.055 |
| 481 | 159 | 38 | 1jj2−1iq4 | 1k9m_F | 1.000 | 0.059 |
| 481 | 159 | 38 | 1jj2−1iq4 | 1kqs_D | 1.000 | 0.065 |
| 481 | 159 | 38 | 1jj2−1iq4 | 1q7y_F | 1.000 | 0.101 |
| 481 | 159 | 38 | 1jj2−1iq4 | 1qvf_D | 1.000 | 0.043 |
| 481 | 159 | 38 | 1jj2−1iq4 | 1k73_F | 1.000 | 0.029 |
| 481 | 159 | 38 | 1jj2−1iq4 | 1qvg_D | 1.000 | 0.111 |
| 481 | 159 | 38 | 1jj2−1iq4 | 1q81_F | 1.000 | 0.136 |
| 481 | 159 | 38 | 1iq4−1jj2 | 1iq4_B | 1.000 | 2.736 |
| 492 | 90 | 37 | 1qce−1df4 | 2ezo_A | 1.000 | 1.025 |
| 492 | 90 | 37 | 1qce−1df4 | 2ezo_B | 1.000 | 1.025 |
| 492 | 90 | 37 | 1qce−1df4 | 2ezo_C | 1.000 | 1.025 |
| 493 | 477 | 37 | 1m0w−2hgs | 1m0w_A | 1.000 | 0.015 |
| 502 | 171 | 37 | 1qjz−1auy | 1e57_B | 0.957 | 9.904 |
| 502 | 171 | 37 | 1auy−1qjz | 1w39_B | 0.963 | 1.340 |
| 502 | 171 | 37 | 1auy−1qjz | 1auy_B | 0.963 | 1.259 |
| 502 | 171 | 37 | 1auy−1qjz | 1auy_C | 0.950 | 1.304 |
| 502 | 171 | 37 | 1qjz−1auy | 1qjz_B | 0.957 | 9.922 |
| 502 | 171 | 37 | 1auy−1qjz | 1w39_A | 0.963 | 1.096 |
| 502 | 171 | 37 | 1auy−1qjz | 1w39_C | 0.944 | 1.340 |
| 505 | 108 | 36 | 1hcn−1fl7 | 1hrp_B | 0.941 | 0.787 |
| 505 | 108 | 36 | 1hcn−1fl7 | 1qfw_B | 0.941 | 2.208 |
| 507 | 106 | 36 | 1dqi−1dfx | 1dqi_D | 1.000 | 1.454 |
| 507 | 106 | 36 | 1dqi−1dfx | 1dqk_C | 1.000 | 1.446 |
| 507 | 106 | 36 | 1dqi−1dfx | 1do6_A | 1.000 | 0.198 |
| 507 | 106 | 36 | 1dqi−1dfx | 1dqi_B | 1.000 | 1.488 |
| 507 | 106 | 36 | 1dqi−1dfx | 1do6_B | 1.000 | 1.430 |
| 507 | 106 | 36 | 1dqi−1dfx | 1dqk_D | 1.000 | 1.452 |
| 507 | 106 | 36 | 1dqi−1dfx | 1dqk_A | 1.000 | 1.427 |
| 507 | 106 | 36 | 1dqi−1dfx | 1dqi_C | 1.000 | 0.103 |
| 507 | 106 | 36 | 1dqi−1dfx | 1dqk_B | 1.000 | 1.424 |
| 511 | 208 | 36 | 1fb1−1fbx | 1fb1_E | 0.979 | 0.452 |
| 511 | 208 | 36 | 1fbx−1fb1 | 1fbx_K | 1.000 | 0.191 |
| 511 | 208 | 36 | 1fbx−1fb1 | 1gtp_O | 1.000 | 0.455 |
| 511 | 208 | 36 | 1fbx−1fb1 | 1fbx_C | 1.000 | 0.234 |
| 511 | 208 | 36 | 1fbx−1fb1 | 1fbx_D | 1.000 | 0.206 |
| 511 | 208 | 36 | 1fbx−1fb1 | 1gtp_M | 1.000 | 0.454 |
| 511 | 208 | 36 | 1fbx−1fb1 | 1fbx_I | 1.000 | 0.213 |
| 511 | 208 | 36 | 1fbx−1fb1 | 1fbx_M | 1.000 | 0.219 |
| 511 | 208 | 36 | 1fbx−1fb1 | 1gtp_R | 1.000 | 0.457 |
| 511 | 208 | 36 | 1fb1−1fbx | 1fb1_C | 0.979 | 0.416 |
| 511 | 208 | 36 | 1fbx−1fb1 | 1gtp_K | 1.000 | 0.456 |
| 511 | 208 | 36 | 1fbx−1fb1 | 1gtp_L | 1.000 | 0.458 |
| 511 | 208 | 36 | 1fbx−1fb1 | 1gtp_C | 1.000 | 0.459 |
| 511 | 208 | 36 | 1fbx−1fb1 | 1gtp_E | 1.000 | 0.456 |
| 511 | 208 | 36 | 1fbx−1fb1 | 1fbx_N | 1.000 | 0.210 |
| 511 | 208 | 36 | 1fbx−1fb1 | 1fbx_B | 1.000 | 0.200 |
| 511 | 208 | 36 | 1fbx−1fb1 | 1fbx_J | 1.000 | 0.210 |
| 511 | 208 | 36 | 1fbx−1fb1 | 1gtp_G | 1.000 | 0.454 |
| 511 | 208 | 36 | 1fbx−1fb1 | 1gtp_I | 1.000 | 0.454 |
| 511 | 208 | 36 | 1fb1−1fbx | 1fb1_D | 0.959 | 0.410 |
| 511 | 208 | 36 | 1fbx−1fb1 | 1gtp_D | 1.000 | 0.454 |
| 511 | 208 | 36 | 1fbx−1fb1 | 1fbx_H | 1.000 | 0.215 |
| 511 | 208 | 36 | 1fbx−1fb1 | 1fbx_L | 1.000 | 0.216 |
| 511 | 208 | 36 | 1fbx−1fb1 | 1gtp_A | 1.000 | 0.456 |
| 511 | 208 | 36 | 1fbx−1fb1 | 1fbx_F | 1.000 | 0.208 |
| 511 | 208 | 36 | 1fbx−1fb1 | 1fbx_G | 1.000 | 0.222 |
| 511 | 208 | 36 | 1fbx−1fb1 | 1gtp_H | 1.000 | 0.455 |
| 511 | 208 | 36 | 1fbx−1fb1 | 1gtp_B | 1.000 | 0.456 |
| 511 | 208 | 36 | 1fb1−1fbx | 1fb1_B | 0.953 | 0.447 |
| 511 | 208 | 36 | 1fbx−1fb1 | 1gtp_F | 1.000 | 0.455 |
| 511 | 208 | 36 | 1fbx−1fb1 | 1gtp_N | 1.000 | 0.458 |
| 511 | 208 | 36 | 1fbx−1fb1 | 1fbx_E | 1.000 | 0.208 |
| 511 | 208 | 36 | 1fbx−1fb1 | 1gtp_J | 1.000 | 0.456 |
| 511 | 208 | 36 | 1fbx−1fb1 | 1gtp_Q | 1.000 | 0.456 |
| 511 | 208 | 36 | 1fbx−1fb1 | 1fbx_O | 1.000 | 0.216 |
| 511 | 208 | 36 | 1fbx−1fb1 | 1gtp_P | 1.000 | 0.454 |
| 511 | 208 | 36 | 1fbx−1fb1 | 1gtp_T | 1.000 | 0.456 |
| 511 | 208 | 36 | 1fbx−1fb1 | 1gtp_S | 1.000 | 0.456 |
| 512 | 482 | 36 | 1ayx−1gai | 2f6d_A | 0.977 | 0.581 |
| 512 | 482 | 36 | 1ayx−1gai | 2fba_A | 0.984 | 0.383 |
| 513 | 142 | 36 | 1apy−1ayy | 1apz_B | 1.000 | 0.263 |
| 513 | 142 | 36 | 1ayy−1apy | 1ayy_D | 0.985 | 2.302 |
| 513 | 142 | 36 | 1apy−1ayy | 1apy_D | 1.000 | 0.258 |
| 513 | 142 | 36 | 1apy−1ayy | 1apz_D | 1.000 | 0.342 |
| 516 | 331 | 36 | 1a99−1pot | 1a99_D | 0.978 | 0.116 |
| 516 | 331 | 36 | 1a99−1pot | 1a99_B | 1.000 | 0.110 |
| 516 | 331 | 36 | 1a99−1pot | 1a99_C | 1.000 | 0.098 |
| 517 | 253 | 36 | 1plq−1axc | 1plr_A | 1.000 | 0.656 |
| 519 | 215 | 36 | 1a2z−1aug | 1a2z_C | 1.000 | 0.454 |
| 519 | 215 | 36 | 1a2z−1aug | 1a2z_D | 1.000 | 0.298 |
| 519 | 215 | 36 | 1a2z−1aug | 1a2z_B | 1.000 | 0.305 |
| 521 | 101 | 36 | 1kaw−3ull | 1kaw_B | 1.000 | 0.001 |
| 521 | 101 | 36 | 1kaw−3ull | 1kaw_C | 1.000 | 0.001 |
| 521 | 101 | 36 | 3ull−1kaw | 1s3o_A | 0.815 | 1.684 |
| 521 | 101 | 36 | 1kaw−3ull | 1kaw_D | 1.000 | 0.001 |
| 522 | 102 | 36 | 1vcb−1fs1 | 1vcb_K | 1.000 | 0.122 |
| 522 | 102 | 36 | 1vcb−1fs1 | 1vcb_E | 1.000 | 0.049 |
| 522 | 102 | 36 | 1vcb−1fs1 | 1vcb_H | 1.000 | 0.132 |
| 524 | 93 | 36 | 1lky−1bqv | 1lky_C | 0.946 | 0.669 |
| 524 | 93 | 36 | 1lky−1bqv | 1lky_E | 0.946 | 0.721 |
| 525 | 686 | 36 | 1evu−1g0d | 1evu_A | 1.000 | 0.005 |
| 526 | 115 | 36 | 1bgy−1ezv | 2fyu_F | 1.000 | 1.397 |
| 526 | 115 | 36 | 1bgy−1ezv | 1bgy_R | 1.000 | 0.067 |
| 526 | 115 | 36 | 1ezv−1bgy | 1kb9_G | 1.000 | 0.065 |
| 526 | 115 | 36 | 1bgy−1ezv | 1be3_F | 1.000 | 0.126 |
| 526 | 115 | 36 | 1ezv−1bgy | 1p84_G | 1.000 | 0.125 |
| 526 | 115 | 36 | 1bgy−1ezv | 1l0l_F | 1.000 | 1.248 |
| 534 | 61 | 35 | 1gp2−1tbg | 1gg2_G | 1.000 | 0.332 |
| 534 | 61 | 35 | 1gp2−1tbg | 2bcj_G | 1.000 | 1.286 |
| 538 | 138 | 35 | 1h9m−1b9m | 1h9m_B | 1.000 | 0.210 |
| 538 | 138 | 35 | 1h9m−1b9m | 1h9j_A | 1.000 | 0.860 |
| 538 | 138 | 35 | 1h9m−1b9m | 1h9k_A | 1.000 | 0.869 |
| 538 | 138 | 35 | 1h9m−1b9m | 1h9m_A | 1.000 | 0.008 |
| 546 | 149 | 35 | 1elk−1dvp | 1elk_B | 1.000 | 0.303 |
| 547 | 575 | 35 | 1qhb−1qi9 | 1qhb_E | 0.795 | 0.093 |
| 547 | 575 | 35 | 1qhb−1qi9 | 1qhb_F | 0.921 | 0.122 |
| 547 | 575 | 35 | 1qhb−1qi9 | 1qhb_B | 0.933 | 0.099 |
| 547 | 575 | 35 | 1qhb−1qi9 | 1qhb_D | 0.988 | 0.091 |
| 547 | 575 | 35 | 1qi9−1qhb | 1qi9_B | 0.931 | 0.237 |
| 547 | 575 | 35 | 1qhb−1qi9 | 1qhb_C | 0.921 | 0.096 |
| 549 | 121 | 34 | 1bvz−1sma | 1bvz_B | 0.992 | 0.361 |
| 549 | 121 | 34 | 1bvz−1sma | 1ji2_A | 1.000 | 0.340 |
| 549 | 121 | 34 | 1bvz−1sma | 1vfk_B | 1.000 | 0.434 |
| 549 | 121 | 34 | 1bvz−1sma | 1ji2_B | 1.000 | 0.406 |
| 549 | 121 | 34 | 1sma−1bvz | 1sma_B | 1.000 | 0.004 |
| 549 | 121 | 34 | 1bvz−1sma | 1vfk_A | 0.992 | 0.426 |
| 554 | 161 | 34 | 1vhr−1mkp | 1vhr_B | 0.965 | 0.833 |
| 557 | 399 | 34 | 1hp5−1qba | 1jak_A | 1.000 | 0.284 |
| 557 | 399 | 34 | 1hp5−1qba | 1hp4_A | 1.000 | 0.241 |
| 557 | 399 | 34 | 1qba−1hp5 | 1qbb_A | 1.000 | 0.101 |
| 557 | 399 | 34 | 1hp5−1qba | 1m01_A | 1.000 | 0.248 |
| 558 | 617 | 34 | 1hc1−1lla | 1hc6_A | 0.883 | 0.208 |
| 558 | 617 | 34 | 1hc1−1lla | 1hc4_A | 0.978 | 0.275 |
| 558 | 617 | 34 | 1hc1−1lla | 1hc3_A | 0.883 | 0.232 |
| 558 | 617 | 34 | 1hc1−1lla | 1hc5_A | 0.979 | 0.230 |
| 558 | 617 | 34 | 1hc1−1lla | 1hc2_A | 0.979 | 0.273 |
| 560 | 275 | 34 | 1e2t−1gx3 | 1e2t_D | 1.000 | 0.203 |
| 560 | 275 | 34 | 1e2t−1gx3 | 1e2t_E | 1.000 | 0.179 |
| 560 | 275 | 34 | 1e2t−1gx3 | 1e2t_H | 1.000 | 0.250 |
| 560 | 275 | 34 | 1e2t−1gx3 | 1e2t_F | 1.000 | 0.258 |
| 560 | 275 | 34 | 1e2t−1gx3 | 1e2t_G | 1.000 | 0.193 |
| 560 | 275 | 34 | 1e2t−1gx3 | 1e2t_B | 1.000 | 0.331 |
| 560 | 275 | 34 | 1e2t−1gx3 | 1e2t_C | 1.000 | 0.287 |
| 561 | 220 | 34 | 1ire−2ahj | 1ugs_B | 1.000 | 0.147 |
| 561 | 220 | 34 | 2ahj−1ire | 2ahj_D | 1.000 | 0.161 |
| 561 | 220 | 34 | 2ahj−1ire | 2d0q_B | 0.995 | 0.336 |
| 561 | 220 | 34 | 2ahj−1ire | 2cz0_B | 0.995 | 0.352 |
| 561 | 220 | 34 | 2ahj−1ire | 2cz1_B | 0.995 | 0.356 |
| 561 | 220 | 34 | 1ire−2ahj | 1ugq_B | 1.000 | 0.132 |
| 561 | 220 | 34 | 2ahj−1ire | 2cz7_B | 0.995 | 0.353 |
| 561 | 220 | 34 | 2ahj−1ire | 2cyz_B | 0.995 | 0.350 |
| 561 | 220 | 34 | 2ahj−1ire | 2cz6_B | 0.995 | 0.356 |
| 561 | 220 | 34 | 1ire−2ahj | 1ugr_B | 0.995 | 0.111 |
| 561 | 220 | 34 | 2ahj−1ire | 2zcf_B | 1.000 | 0.343 |
| 562 | 330 | 34 | 1qjv−1gq8 | 1qjv_B | 0.997 | 0.330 |
| 562 | 330 | 34 | 1qjv−1gq8 | 1qjv_A | 1.000 | 0.002 |
| 563 | 214 | 34 | 1jg1−1dl5 | 1jg2_A | 1.000 | 0.157 |
| 563 | 214 | 34 | 1jg1−1dl5 | 1jg3_A | 1.000 | 0.647 |
| 563 | 214 | 34 | 1jg1−1dl5 | 1jg3_B | 1.000 | 0.635 |
| 563 | 214 | 34 | 1dl5−1jg1 | 1dl5_B | 1.000 | 0.430 |
| 563 | 214 | 34 | 1jg1−1dl5 | 1jg4_A | 1.000 | 0.553 |
| 567 | 158 | 34 | 1shk−1kag | 2shk_A | 1.000 | 0.165 |
| 571 | 350 | 34 | 1uro−1j93 | 1r3q_A | 1.000 | 0.248 |
| 573 | 475 | 33 | 2pgd−1pgj | 1pgp_A | 0.979 | 0.218 |
| 573 | 475 | 33 | 1pgj−2pgd | 1pgj_A | 1.000 | 0.339 |
| 573 | 475 | 33 | 2pgd−1pgj | 1pgn_A | 0.976 | 0.262 |
| 573 | 475 | 33 | 2pgd−1pgj | 1pgq_A | 0.976 | 0.251 |
| 573 | 475 | 33 | 2pgd−1pgj | 1pgo_A | 0.996 | 0.200 |
| 574 | 250 | 33 | 1isi−1lbe | 1ish_A | 1.000 | 0.163 |
| 574 | 250 | 33 | 1lbe−1isi | 1r15_C | 1.000 | 0.458 |
| 574 | 250 | 33 | 1lbe−1isi | 1r15_E | 1.000 | 0.509 |
| 574 | 250 | 33 | 1lbe−1isi | 1r16_A | 0.959 | 0.582 |
| 574 | 250 | 33 | 1isi−1lbe | 1isi_B | 0.988 | 0.331 |
| 574 | 250 | 33 | 1lbe−1isi | 1r15_F | 1.000 | 0.469 |
| 574 | 250 | 33 | 1isi−1lbe | 1isg_A | 0.988 | 0.223 |
| 574 | 250 | 33 | 1lbe−1isi | 1r15_G | 1.000 | 0.482 |
| 574 | 250 | 33 | 1lbe−1isi | 1r15_H | 1.000 | 0.478 |
| 574 | 250 | 33 | 1isi−1lbe | 1ish_B | 0.971 | 0.297 |
| 574 | 250 | 33 | 1lbe−1isi | 1r15_B | 1.000 | 0.458 |
| 574 | 250 | 33 | 1isi−1lbe | 1isf_B | 0.988 | 0.334 |
| 574 | 250 | 33 | 1lbe−1isi | 1r12_A | 0.959 | 0.552 |
| 574 | 250 | 33 | 1isi−1lbe | 1ism_B | 0.988 | 0.348 |

n: internal numbering; length of alignment; % sequence ID (cf. Homstrad); refA−refB: reference alignment; altA: alternative for struc. A; SP: sum−of−pairs score of alt. alignment w/r reference

| n | length | %ID | refA−refB | altA | SP | RMSD |
|---|---|---|---|---|---|---|
| 574 | 250 | 33 | 1lbe−1isi | 1r16_B | 0.971 | 0.553 |
| 574 | 250 | 33 | 1lbe−1isi | 1r12_B | 0.992 | 0.552 |
| 574 | 250 | 33 | 1lbe−1isi | 1r15_D | 1.000 | 0.451 |
| 574 | 250 | 33 | 1isi−1lbe | 1isj_A | 1.000 | 0.201 |
| 574 | 250 | 33 | 1lbe−1isi | 1r15_A | 1.000 | 0.483 |
| 574 | 250 | 33 | 1isi−1lbe | 1isg_B | 0.988 | 0.354 |
| 574 | 250 | 33 | 1isi−1lbe | 1isf_A | 1.000 | 0.212 |
| 574 | 250 | 33 | 1isi−1lbe | 1isj_B | 0.988 | 0.389 |
| 574 | 250 | 33 | 1isi−1lbe | 1ism_A | 0.967 | 0.300 |
| 587 | 146 | 33 | 1gof−1euu | 2eie_A | 1.000 | 0.252 |
| 587 | 146 | 33 | 1euu−1gof | 1eut_A | 1.000 | 0.174 |
| 587 | 146 | 33 | 1gof−1euu | 1gog_A | 1.000 | 0.112 |
| 587 | 146 | 33 | 1gof−1euu | 1goh_A | 1.000 | 0.143 |
| 588 | 205 | 33 | 1bf8−1qpx | 1ze3_C | 0.963 | 4.071 |
| 591 | 52 | 33 | 1kzu−1lgh | 1nkz_A | 0.979 | 0.394 |
| 591 | 52 | 33 | 1kzu−1lgh | 2fkw_R | 0.979 | 0.530 |
| 591 | 52 | 33 | 1kzu−1lgh | 2fkw_I | 0.979 | 0.512 |
| 591 | 52 | 33 | 1kzu−1lgh | 2fkw_E | 0.979 | 0.522 |
| 591 | 52 | 33 | 1kzu−1lgh | 2fkw_G | 0.979 | 0.538 |
| 591 | 52 | 33 | 1kzu−1lgh | 2fkw_K | 0.979 | 0.519 |
| 591 | 52 | 33 | 1kzu−1lgh | 1nkz_C | 0.979 | 0.364 |
| 591 | 52 | 33 | 1kzu−1lgh | 1nkz_E | 0.979 | 0.380 |
| 591 | 52 | 33 | 1kzu−1lgh | 2fkw_C | 0.979 | 0.516 |
| 591 | 52 | 33 | 1kzu−1lgh | 2fkw_M | 0.979 | 0.520 |
| 591 | 52 | 33 | 1kzu−1lgh | 2fkw_A | 0.979 | 0.513 |
| 591 | 52 | 33 | 1kzu−1lgh | 2fkw_O | 0.979 | 0.522 |
| 593 | 135 | 33 | 1eyv−1ey1 | 1eyv_B | 1.000 | 1.001 |
| 594 | 102 | 33 | 1f39−1umu | 1f39_B | 1.000 | 0.557 |
| 594 | 102 | 33 | 1umu−1f39 | 1umu_B | 1.000 | 0.306 |
| 595 | 223 | 33 | 1auo−1fj2 | 1aur_A | 1.000 | 0.201 |
| 595 | 223 | 33 | 1fj2−1auo | 1fj2_B | 0.995 | 0.364 |
| 595 | 223 | 33 | 1auo−1fj2 | 1aur_B | 0.995 | 0.488 |
| 595 | 223 | 33 | 1auo−1fj2 | 1auo_B | 0.995 | 0.484 |
| 600 | 158 | 33 | 1gz0−1ipa | 1gz0_D | 0.953 | 0.402 |
| 600 | 158 | 33 | 1gz0−1ipa | 1gz0_E | 0.960 | 0.704 |
| 600 | 158 | 33 | 1gz0−1ipa | 1gz0_H | 0.953 | 0.746 |
| 600 | 158 | 33 | 1gz0−1ipa | 1gz0_F | 0.953 | 0.454 |
| 600 | 158 | 33 | 1gz0−1ipa | 1gz0_G | 0.960 | 0.720 |
| 600 | 158 | 33 | 1gz0−1ipa | 1gz0_B | 0.953 | 0.442 |
| 600 | 158 | 33 | 1gz0−1ipa | 1gz0_C | 0.953 | 0.393 |
| 602 | 526 | 33 | 1l0v−1chu | 1kf6_A | 0.978 | 0.384 |
| 602 | 526 | 33 | 1l0v−1chu | 1l0v_M | 0.773 | 0.765 |
| 602 | 526 | 33 | 1l0v−1chu | 1kf6_M | 0.955 | 0.486 |
| 602 | 526 | 33 | 1l0v−1chu | 1kfy_M | 0.989 | 0.452 |
| 602 | 526 | 33 | 1l0v−1chu | 1kfy_A | 0.773 | 0.384 |
| 603 | 253 | 33 | 1qop−1geq | 1qop_A | 1.000 | 0.027 |
| 603 | 253 | 33 | 1geq−1qop | 1wdw_C | 1.000 | 0.710 |
| 605 | 464 | 32 | 1ed9−1ew2 | 1ew8_B | 0.984 | 0.334 |
| 605 | 464 | 32 | 1ed9−1ew2 | 1ew9_A | 0.955 | 0.176 |
| 605 | 464 | 32 | 1ed9−1ew2 | 1y6v_B | 0.966 | 0.295 |
| 605 | 464 | 32 | 1ed9−1ew2 | 1ed8_A | 0.942 | 0.166 |
| 605 | 464 | 32 | 1ed9−1ew2 | 1ew9_B | 0.966 | 0.300 |
| 605 | 464 | 32 | 1ed9−1ew2 | 1y6v_A | 1.000 | 0.260 |
| 605 | 464 | 32 | 1ed9−1ew2 | 1ew8_A | 1.000 | 0.191 |
| 607 | 121 | 32 | 1rav−1pts | 1rav_B | 0.991 | 0.818 |
| 608 | 67 | 32 | 1fse−1a04 | 1fse_B | 0.938 | 1.878 |
| 608 | 67 | 32 | 1fse−1a04 | 1fse_C | 0.938 | 1.672 |
| 608 | 67 | 32 | 1a04−1fse | 1a04_B | 1.000 | 0.469 |
| 617 | 486 | 32 | 1dpg−1qki | 1dpg_B | 0.979 | 0.723 |
| 619 | 195 | 32 | 2mjp−1k7k | 1b78_A | 1.000 | 0.198 |
| 619 | 195 | 32 | 2mjp−1k7k | 2mjp_B | 1.000 | 0.714 |
| 619 | 195 | 32 | 2mjp−1k7k | 1b78_B | 1.000 | 0.683 |
| 628 | 191 | 32 | 1ile−1qu2 | 1jzs_A | 0.950 | 0.468 |
| 628 | 191 | 32 | 1ile−1qu2 | 1jzq_A | 0.978 | 0.344 |
| 628 | 191 | 32 | 1qu2−1ile | 1qu3_A | 0.823 | 1.479 |
| 632 | 325 | 31 | 6mht−1dct | 9mht_A | 0.990 | 0.249 |
| 632 | 325 | 31 | 6mht−1dct | 7mht_A | 0.977 | 0.191 |
| 632 | 325 | 31 | 6mht−1dct | 10mh_A | 0.963 | 0.220 |
| 632 | 325 | 31 | 6mht−1dct | 2c7p_A | 0.967 | 0.761 |
| 632 | 325 | 31 | 6mht−1dct | 1hmy_A | 0.920 | 3.766 |
| 632 | 325 | 31 | 6mht−1dct | 4mht_A | 0.967 | 0.618 |

| n | length | %ID | refA−refB | altA | SP | RMSD |
|---|---|---|---|---|---|---|
| 632 | 325 | 31 | 6mht−1dct | 8mht_A | 0.993 | 0.225 |
| 632 | 325 | 31 | 6mht−1dct | 2c7o_A | 0.967 | 0.754 |
| 632 | 325 | 31 | 6mht−1dct | 2c7q_A | 0.967 | 0.805 |
| 632 | 325 | 31 | 6mht−1dct | 2hr1_A | 0.967 | 0.727 |
| 632 | 325 | 31 | 6mht−1dct | 3mht_A | 0.967 | 0.638 |
| 632 | 325 | 31 | 6mht−1dct | 5mht_A | 0.980 | 0.205 |
| 632 | 325 | 31 | 6mht−1dct | 1m0e_A | 0.967 | 0.747 |
| 632 | 325 | 31 | 1dct−6mht | 1dct_B | 0.960 | 0.343 |
| 632 | 325 | 31 | 6mht−1dct | 2hmy_B | 0.917 | 3.820 |
| 632 | 325 | 31 | 6mht−1dct | 1skm_A | 0.963 | 0.650 |
| 632 | 325 | 31 | 6mht−1dct | 1mht_A | 0.967 | 0.617 |
| 636 | 529 | 31 | 1epw−3bta | 1g9b_A | 0.909 | 0.203 |
| 636 | 529 | 31 | 1epw−3bta | 1g9c_A | 0.914 | 0.359 |
| 636 | 529 | 31 | 1epw−3bta | 1s0b_A | 0.903 | 0.263 |
| 636 | 529 | 31 | 1epw−3bta | 1g9d_A | 0.911 | 0.282 |
| 636 | 529 | 31 | 1epw−3bta | 1s0d_A | 0.924 | 0.269 |
| 636 | 529 | 31 | 1epw−3bta | 1s0f_A | 0.914 | 0.356 |
| 636 | 529 | 31 | 1epw−3bta | 1i1e_A | 0.920 | 0.236 |
| 636 | 529 | 31 | 1epw−3bta | 1s0e_A | 0.877 | 0.200 |
| 636 | 529 | 31 | 1epw−3bta | 1g9a_A | 0.928 | 0.225 |
| 636 | 529 | 31 | 1epw−3bta | 1s0c_A | 0.907 | 0.190 |
| 642 | 97 | 31 | 1n0v−1dar | 1zm9_E | 1.000 | 0.806 |
| 642 | 97 | 31 | 1n0v−1dar | 2npf_A | 1.000 | 0.679 |
| 642 | 97 | 31 | 1n0v−1dar | 1zm2_C | 1.000 | 0.632 |
| 642 | 97 | 31 | 1n0v−1dar | 1zm4_A | 1.000 | 0.655 |
| 642 | 97 | 31 | 1n0v−1dar | 1zm3_C | 1.000 | 0.640 |
| 642 | 97 | 31 | 1dar−1n0v | 1ktv_A | 1.000 | 0.597 |
| 642 | 97 | 31 | 1n0v−1dar | 1zm9_C | 1.000 | 0.649 |
| 642 | 97 | 31 | 1dar−1n0v | 1ktv_B | 1.000 | 0.588 |
| 642 | 97 | 31 | 1n0v−1dar | 2e1r_A | 1.000 | 0.458 |
| 642 | 97 | 31 | 1n0v−1dar | 1zm2_A | 1.000 | 0.686 |
| 642 | 97 | 31 | 1n0v−1dar | 1zm4_C | 1.000 | 0.597 |
| 642 | 97 | 31 | 1n0v−1dar | 1zm9_A | 1.000 | 0.716 |
| 642 | 97 | 31 | 1n0v−1dar | 1zm3_E | 1.000 | 0.812 |
| 642 | 97 | 31 | 1n0v−1dar | 1n0v_D | 1.000 | 0.541 |
| 642 | 97 | 31 | 1n0v−1dar | 1zm3_A | 1.000 | 0.698 |
| 642 | 97 | 31 | 1n0v−1dar | 1zm4_E | 1.000 | 0.819 |
| 642 | 97 | 31 | 1dar−1n0v | 1elo_A | 1.000 | 0.570 |
| 642 | 97 | 31 | 1n0v−1dar | 1zm2_E | 1.000 | 0.818 |
| 646 | 119 | 31 | 1ipi−1hh1 | 1ipi_B | 1.000 | 1.126 |
| 646 | 119 | 31 | 1ipi−1hh1 | 1gef_A | 1.000 | 1.412 |
| 646 | 119 | 31 | 1ipi−1hh1 | 1gef_B | 0.981 | 1.591 |
| 646 | 119 | 31 | 1ipi−1hh1 | 1gef_E | 0.916 | 1.638 |
| 648 | 146 | 31 | 1e3m−1ewq | 1e3m_B | 0.992 | 0.439 |
| 649 | 130 | 31 | 1bvp−1ahs | 1bvp_4 | 1.000 | 0.001 |
| 649 | 130 | 31 | 1bvp−1ahs | 1bvp_5 | 1.000 | 0.001 |
| 649 | 130 | 31 | 1ahs−1bvp | 1ahs_C | 1.000 | 0.364 |
| 649 | 130 | 31 | 1ahs−1bvp | 1ahs_B | 1.000 | 0.373 |
| 649 | 130 | 31 | 1bvp−1ahs | 1bvp_3 | 1.000 | 0.001 |
| 650 | 107 | 31 | 1g28−1byw | 1g28_D | 1.000 | 0.015 |
| 650 | 107 | 31 | 1g28−1byw | 1jnu_A | 1.000 | 0.275 |
| 650 | 107 | 31 | 1g28−1byw | 1jnu_D | 1.000 | 0.274 |
| 650 | 107 | 31 | 1g28−1byw | 1jnu_B | 1.000 | 0.274 |
| 650 | 107 | 31 | 1g28−1byw | 1jnu_C | 1.000 | 0.276 |
| 650 | 107 | 31 | 1g28−1byw | 1g28_B | 1.000 | 0.015 |
| 650 | 107 | 31 | 1g28−1byw | 1g28_C | 1.000 | 0.013 |
| 653 | 281 | 31 | 1toa−1psz | 1toa_B | 1.000 | 0.176 |
| 654 | 285 | 31 | 1gym−2plc | 1ptd_A | 0.996 | 0.506 |
| 654 | 285 | 31 | 1gym−2plc | 1ptg_A | 0.996 | 0.479 |
| 655 | 170 | 31 | 1g2a−1jym | 1lru_A | 1.000 | 0.684 |
| 655 | 170 | 31 | 1g2a−1jym | 1bs6_A | 1.000 | 0.736 |
| 655 | 170 | 31 | 1g2a−1jym | 1bs7_A | 1.000 | 0.576 |
| 655 | 170 | 31 | 1g2a−1jym | 1xeo_A | 0.887 | 0.373 |
| 655 | 170 | 31 | 1g2a−1jym | 1bsk_A | 0.893 | 0.620 |
| 655 | 170 | 31 | 1g2a−1jym | 2ai8_C | 0.887 | 0.274 |
| 655 | 170 | 31 | 1g2a−1jym | 2ai8_A | 0.899 | 0.153 |
| 655 | 170 | 31 | 1g2a−1jym | 1bs8_A | 0.987 | 0.639 |
| 655 | 170 | 31 | 1g2a−1jym | 1g2a_B | 0.987 | 0.279 |
| 655 | 170 | 31 | 1g2a−1jym | 1g27_A | 0.987 | 0.269 |
| 655 | 170 | 31 | 1jym−1g2a | 1jym_B | 0.899 | 0.571 |
| 655 | 170 | 31 | 1g2a−1jym | 1bs4_A | 0.887 | 0.503 |

| n | length | %ID | refA−refB | altA | SP | RMSD |
|---|---|---|---|---|---|---|
| 655 | 170 | 31 | 1g2a−1jym | 1g27_C | 0.987 | 0.585 |
| 655 | 170 | 31 | 1g2a−1jym | 1icj_A | 0.887 | 0.482 |
| 655 | 170 | 31 | 1g2a−1jym | 1bsj_A | 0.981 | 0.624 |
| 655 | 170 | 31 | 1g2a−1jym | 1lru_B | 0.987 | 0.248 |
| 655 | 170 | 31 | 1g2a−1jym | 1g27_B | 0.994 | 0.468 |
| 655 | 170 | 31 | 1g2a−1jym | 2ai8_B | 0.887 | 0.603 |
| 655 | 170 | 31 | 1g2a−1jym | 1bs5_A | 0.899 | 0.565 |
| 655 | 170 | 31 | 1g2a−1jym | 1xem_A | 0.887 | 0.345 |
| 655 | 170 | 31 | 1g2a−1jym | 1g2a_C | 0.887 | 0.523 |
| 655 | 170 | 31 | 1g2a−1jym | 1xen_A | 0.887 | 0.366 |
| 655 | 170 | 31 | 1g2a−1jym | 1bsz_A | 0.899 | 0.500 |
| 656 | 113 | 31 | 1h4x−1buz | 1h4x_B | 0.991 | 0.707 |
| 657 | 124 | 31 | 1f7u−1iq0 | 1bs2_A | 0.958 | 1.761 |
| 657 | 124 | 31 | 1f7u−1iq0 | 1f7v_A | 1.000 | 0.206 |
| 659 | 195 | 31 | 1ig0−1ig3 | 1ig0_B | 0.887 | 0.843 |
| 660 | 100 | 31 | 1qsd−1h7c | 1qsd_B | 1.000 | 0.216 |
| 661 | 364 | 30 | 1pjc−1f8g | 1say_A | 0.967 | 0.630 |
| 661 | 364 | 30 | 1pjc−1f8g | 1pjb_A | 0.967 | 0.612 |
| 662 | 171 | 30 | 1pjc−1f8g | 1pjb_A | 0.987 | 0.830 |
| 662 | 171 | 30 | 1pjc−1f8g | 1say_A | 0.987 | 0.851 |
| 663 | 71 | 30 | 1xxa−1b4b | 1xxc_F | 0.986 | 0.688 |
| 663 | 71 | 30 | 1xxa−1b4b | 1xxa_D | 0.929 | 0.931 |
| 663 | 71 | 30 | 1xxa−1b4b | 1xxa_E | 1.000 | 0.536 |
| 663 | 71 | 30 | 1xxa−1b4b | 1xxc_B | 0.929 | 0.580 |
| 663 | 71 | 30 | 1xxa−1b4b | 1xxb_C | 1.000 | 0.533 |
| 663 | 71 | 30 | 1xxa−1b4b | 1xxb_E | 1.000 | 0.465 |
| 663 | 71 | 30 | 1xxa−1b4b | 1xxc_D | 0.929 | 0.638 |
| 663 | 71 | 30 | 1xxa−1b4b | 1xxb_D | 0.986 | 0.517 |
| 663 | 71 | 30 | 1xxa−1b4b | 1xxc_C | 0.929 | 0.587 |
| 663 | 71 | 30 | 1xxa−1b4b | 1xxb_B | 1.000 | 0.533 |
| 663 | 71 | 30 | 1xxa−1b4b | 1xxc_E | 1.000 | 0.632 |
| 663 | 71 | 30 | 1xxa−1b4b | 1xxa_B | 1.000 | 0.577 |
| 663 | 71 | 30 | 1xxa−1b4b | 1xxc_A | 1.000 | 0.619 |
| 663 | 71 | 30 | 1xxa−1b4b | 1xxb_A | 1.000 | 0.453 |
| 663 | 71 | 30 | 1xxa−1b4b | 1xxb_F | 0.986 | 0.532 |
| 663 | 71 | 30 | 1b4b−1xxa | 1b4b_B | 0.929 | 0.048 |
| 663 | 71 | 30 | 1xxa−1b4b | 1xxa_C | 0.986 | 0.414 |
| 663 | 71 | 30 | 1b4b−1xxa | 1b4b_C | 1.000 | 0.048 |
| 663 | 71 | 30 | 1xxa−1b4b | 1xxa_F | 1.000 | 0.769 |
| 664 | 92 | 30 | 1in1−1cdz | 1imo_A | 1.000 | 0.334 |
| 674 | 310 | 30 | 1bf6−1psc | 1bf6_B | 0.979 | 0.149 |
| 674 | 310 | 30 | 1psc−1bf6 | 1psc_B | 0.965 | 0.133 |
| 675 | 214 | 30 | 1lm7−1lm5 | 1lm7_B | 1.000 | 0.228 |
| 677 | 103 | 30 | 2ffh−1qb2 | 2ffh_C | 1.000 | 0.189 |
| 677 | 103 | 30 | 2ffh−1qb2 | 2ffh_B | 1.000 | 0.201 |
| 677 | 103 | 30 | 1qb2−2ffh | 1qb2_B | 1.000 | 1.353 |
| 679 | 125 | 30 | 1du3−1ext | 1du3_H | 0.944 | 0.733 |
| 679 | 125 | 30 | 1du3−1ext | 1du3_I | 0.989 | 0.545 |
| 679 | 125 | 30 | 1du3−1ext | 1du3_C | 1.000 | 0.740 |
| 680 | 88 | 30 | 1e32−1cz5 | 1s3s_C | 0.850 | 0.353 |
| 680 | 88 | 30 | 1e32−1cz5 | 1s3s_D | 0.988 | 0.541 |
| 680 | 88 | 30 | 1cz5−1e32 | 1cz4_A | 0.712 | 3.273 |
| 680 | 88 | 30 | 1e32−1cz5 | 1s3s_E | 0.875 | 0.396 |
| 680 | 88 | 30 | 1e32−1cz5 | 1s3s_F | 0.850 | 0.362 |
| 680 | 88 | 30 | 1e32−1cz5 | 1s3s_A | 0.962 | 0.446 |
| 680 | 88 | 30 | 1e32−1cz5 | 1s3s_B | 0.800 | 0.445 |
| 684 | 412 | 29 | 1kor−1k92 | 1kh3_A | 0.926 | 0.174 |
| 684 | 412 | 29 | 1kor−1k92 | 1j21_D | 0.995 | 0.248 |
| 684 | 412 | 29 | 1k92−1kor | 1k92_A | 1.000 | 0.426 |
| 684 | 412 | 29 | 1kor−1k92 | 1j21_B | 0.960 | 0.398 |
| 684 | 412 | 29 | 1kor−1k92 | 1kh1_A | 0.926 | 0.330 |
| 684 | 412 | 29 | 1kor−1k92 | 1j21_A | 1.000 | 0.187 |
| 684 | 412 | 29 | 1kor−1k92 | 1j21_C | 0.981 | 0.313 |
| 684 | 412 | 29 | 1kor−1k92 | 1kh2_A | 0.995 | 0.348 |
| 686 | 112 | 29 | 1exj−1jbg | 1exi_A | 1.000 | 0.373 |
| 686 | 112 | 29 | 1exj−1jbg | 1r8e_A | 1.000 | 0.617 |
| 686 | 112 | 29 | 1jbg−1exj | 1r8d_A | 0.953 | 1.359 |
| 686 | 112 | 29 | 1jbg−1exj | 1r8d_B | 0.953 | 1.302 |
| 694 | 94 | 29 | 1ef1−1gg3 | 1ef1_B | 1.000 | 0.040 |
| 694 | 94 | 29 | 1gg3−1ef1 | 1gg3_B | 0.943 | 1.077 |
| 694 | 94 | 29 | 1gg3−1ef1 | 1gg3_C | 0.989 | 0.734 |

| n | length | %ID | refA−refB | altA | SP | RMSD |
|---|---|---|---|---|---|---|
| 695 | 351 | 29 | 1evi−1c0p | 1evi_B | 0.987 | 0.269 |
| 695 | 351 | 29 | 1c0p−1evi | 1c0i_A | 0.987 | 0.354 |
| 695 | 351 | 29 | 1c0p−1evi | 1c0l_A | 0.987 | 0.225 |
| 695 | 351 | 29 | 1evi−1c0p | 1an9_A | 1.000 | 0.348 |
| 695 | 351 | 29 | 1evi−1c0p | 1an9_B | 0.984 | 0.435 |
| 695 | 351 | 29 | 1c0p−1evi | 1c0k_A | 0.987 | 0.217 |
| 697 | 239 | 29 | 1fx8−1ih5 | 1ldi_A | 0.657 | 0.161 |
| 697 | 239 | 29 | 1ih5−1fx8 | 1fqy_A | 0.737 | 3.437 |
| 697 | 239 | 29 | 1ih5−1fx8 | 1h6i_A | 0.671 | 3.444 |
| 697 | 239 | 29 | 1fx8−1ih5 | 1lda_A | 0.643 | 0.173 |
| 698 | 528 | 29 | 1aoz−1a65 | 1asq_B | 0.989 | 0.310 |
| 698 | 528 | 29 | 1aoz−1a65 | 1aso_A | 0.974 | 0.133 |
| 698 | 528 | 29 | 1aoz−1a65 | 1asp_A | 0.974 | 0.147 |
| 698 | 528 | 29 | 1aoz−1a65 | 1asp_B | 0.979 | 0.309 |
| 698 | 528 | 29 | 1aoz−1a65 | 1aso_B | 0.991 | 0.303 |
| 698 | 528 | 29 | 1aoz−1a65 | 1asq_A | 0.987 | 0.145 |
| 698 | 528 | 29 | 1aoz−1a65 | 1aoz_B | 0.972 | 0.300 |
| 700 | 206 | 29 | 1bk7−1bol | 1ucd_A | 0.971 | 0.561 |
| 700 | 206 | 29 | 1bk7−1bol | 1uca_A | 0.959 | 0.104 |
| 700 | 206 | 29 | 1bk7−1bol | 1ucc_A | 0.959 | 0.169 |
| 701 | 138 | 29 | 1jj2−1ily | 1k73_O | 1.000 | 0.037 |
| 701 | 138 | 29 | 1jj2−1ily | 1k8a_O | 1.000 | 0.081 |
| 701 | 138 | 29 | 1jj2−1ily | 1k9m_O | 0.944 | 0.081 |
| 701 | 138 | 29 | 1jj2−1ily | 1kc8_O | 1.000 | 0.068 |
| 701 | 138 | 29 | 1jj2−1ily | 1kd1_O | 0.956 | 0.094 |
| 701 | 138 | 29 | 1jj2−1ily | 1kqs_M | 1.000 | 0.090 |
| 701 | 138 | 29 | 1jj2−1ily | 1m1k_O | 1.000 | 0.077 |
| 701 | 138 | 29 | 1jj2−1ily | 1m90_O | 1.000 | 0.063 |
| 701 | 138 | 29 | 1jj2−1ily | 1n8r_O | 1.000 | 0.100 |
| 701 | 138 | 29 | 1jj2−1ily | 1nji_O | 0.956 | 0.054 |
| 701 | 138 | 29 | 1jj2−1ily | 1q7y_O | 1.000 | 0.149 |
| 701 | 138 | 29 | 1jj2−1ily | 1q81_O | 1.000 | 0.157 |
| 701 | 138 | 29 | 1jj2−1ily | 1q82_O | 1.000 | 0.085 |
| 701 | 138 | 29 | 1jj2−1ily | 1q86_O | 1.000 | 0.113 |
| 701 | 138 | 29 | 1jj2−1ily | 1qvf_M | 1.000 | 0.050 |
| 701 | 138 | 29 | 1jj2−1ily | 1qvg_M | 1.000 | 0.129 |
| 701 | 138 | 29 | 1jj2−1ily | 1w2b_M | 1.000 | 0.223 |
| 702 | 284 | 29 | 1j8f−1ici | 1j8f_B | 1.000 | 0.772 |
| 705 | 286 | 29 | 1ig0−1ig3 | 1ig0_B | 0.933 | 0.722 |
| 706 | 207 | 29 | 1tmk−4tmk | 1tmk_B | 0.978 | 0.271 |
| 706 | 207 | 29 | 1tmk−4tmk | 2tmk_A | 0.978 | 0.355 |
| 706 | 207 | 29 | 4tmk−1tmk | 4tmk_A | 1.000 | 0.011 |
| 706 | 207 | 29 | 1tmk−4tmk | 2tmk_B | 0.989 | 0.377 |
| 706 | 207 | 29 | 4tmk−1tmk | 5tmp_A | 0.978 | 0.209 |
| 713 | 450 | 28 | 1ock−1m22 | 1ocl_B | 0.993 | 0.263 |
| 713 | 450 | 28 | 1ock−1m22 | 1ock_B | 0.980 | 0.271 |
| 713 | 450 | 28 | 1ock−1m22 | 1ocl_A | 0.980 | 0.156 |
| 713 | 450 | 28 | 1m22−1ock | 1m22_B | 0.968 | 0.016 |
| 713 | 450 | 28 | 1ock−1m22 | 1ocm_A | 0.980 | 0.178 |
| 713 | 450 | 28 | 1ock−1m22 | 1ocm_B | 0.963 | 0.257 |
| 716 | 196 | 28 | 1ejh−1ap8 | 1ejh_B | 0.989 | 1.580 |
| 716 | 196 | 28 | 1ejh−1ap8 | 1ejh_C | 0.989 | 1.565 |
| 717 | 174 | 28 | 1g63−1e20 | 1g63_I | 0.975 | 0.360 |
| 717 | 174 | 28 | 1g63−1e20 | 1g63_G | 0.975 | 0.359 |
| 717 | 174 | 28 | 1g63−1e20 | 1g63_J | 0.975 | 0.412 |
| 717 | 174 | 28 | 1g63−1e20 | 1g63_L | 0.975 | 0.436 |
| 717 | 174 | 28 | 1g63−1e20 | 1g63_H | 0.975 | 0.391 |
| 724 | 60 | 28 | 1hfe−1feh | 1hfe_S | 1.000 | 0.019 |
| 724 | 60 | 28 | 1hfe−1feh | 1hfe_T | 0.915 | 0.181 |
| 724 | 60 | 28 | 1feh−1hfe | 1c4a_A | 0.915 | 0.301 |
| 724 | 60 | 28 | 1feh−1hfe | 1c4c_A | 0.915 | 0.356 |
| 728 | 81 | 28 | 1acp−2af8 | 2fad_B | 0.797 | 4.658 |
| 728 | 81 | 28 | 1acp−2af8 | 1t8k_A | 0.797 | 4.564 |
| 728 | 81 | 28 | 1acp−2af8 | 2fae_A | 0.797 | 4.739 |
| 728 | 81 | 28 | 1acp−2af8 | 2fae_B | 0.797 | 4.692 |
| 728 | 81 | 28 | 1acp−2af8 | 2fac_A | 0.784 | 4.720 |
| 728 | 81 | 28 | 1acp−2af8 | 2fad_A | 0.797 | 4.707 |
| 728 | 81 | 28 | 1acp−2af8 | 2fac_B | 0.797 | 4.650 |
| 733 | 142 | 28 | 1evu−1g0d | 1evu_A | 1.000 | 0.001 |
| 734 | 87 | 27 | 2abd−1hbk | 1hb8_C | 1.000 | 1.970 |
| 734 | 87 | 27 | 2abd−1hbk | 1hb6_A | 1.000 | 2.087 |

| n | length | %ID | refA−refB | altA | SP | RMSD |
|---|---|---|---|---|---|---|
| 734 | 87 | 27 | 2abd−1hbk | 1hb8_A | 1.000 | 1.955 |
| 734 | 87 | 27 | 2abd−1hbk | 1hb8_B | 1.000 | 1.967 |
| 735 | 286 | 27 | 5daa−1a3g | 5daa_B | 0.989 | 0.207 |
| 735 | 286 | 27 | 1a3g−5daa | 1a3g_B | 1.000 | 0.298 |
| 735 | 286 | 27 | 1a3g−5daa | 1a3g_C | 0.993 | 0.269 |
| 736 | 76 | 27 | 1b4a−1aoy | 1b4a_D | 0.986 | 1.317 |
| 736 | 76 | 27 | 1b4a−1aoy | 1b4a_E | 0.986 | 1.526 |
| 736 | 76 | 27 | 1b4a−1aoy | 1b4a_F | 0.986 | 0.570 |
| 736 | 76 | 27 | 1b4a−1aoy | 1b4a_B | 0.986 | 1.323 |
| 736 | 76 | 27 | 1b4a−1aoy | 1b4a_C | 0.986 | 1.471 |
| 740 | 141 | 27 | 1coz−1b6t | 1n1d_C | 0.864 | 0.771 |
| 740 | 141 | 27 | 1coz−1b6t | 1n1d_D | 1.000 | 0.867 |
| 740 | 141 | 27 | 1b6t−1coz | 1b6t_B | 0.932 | 1.082 |
| 740 | 141 | 27 | 1b6t−1coz | 1h1t_B | 0.966 | 0.843 |
| 740 | 141 | 27 | 1coz−1b6t | 1n1d_A | 0.958 | 0.888 |
| 740 | 141 | 27 | 1b6t−1coz | 1gn8_A | 0.966 | 0.506 |
| 740 | 141 | 27 | 1b6t−1coz | 1h1t_A | 0.966 | 0.222 |
| 740 | 141 | 27 | 1coz−1b6t | 1n1d_B | 0.992 | 0.823 |
| 740 | 141 | 27 | 1b6t−1coz | 1gn8_B | 0.966 | 1.005 |
| 743 | 334 | 27 | 1hrk−1doz | 1hrk_B | 0.980 | 0.166 |
| 743 | 334 | 27 | 1hrk−1doz | 2hrc_A | 0.951 | 0.626 |
| 744 | 347 | 27 | 2nac−1gdh | 2nac_B | 0.997 | 0.363 |
| 744 | 347 | 27 | 2nac−1gdh | 2nad_B | 0.987 | 1.190 |
| 744 | 347 | 27 | 1gdh−2nac | 1gdh_B | 0.977 | 0.337 |
| 744 | 347 | 27 | 2nac−1gdh | 2nad_A | 0.987 | 1.180 |
| 746 | 132 | 27 | 1shs−1gme | 1shs_D | 0.946 | 0.029 |
| 746 | 132 | 27 | 1shs−1gme | 1shs_E | 0.865 | 0.029 |
| 746 | 132 | 27 | 1shs−1gme | 1shs_H | 0.865 | 0.027 |
| 746 | 132 | 27 | 1gme−1shs | 1gme_C | 1.000 | 1.041 |
| 746 | 132 | 27 | 1shs−1gme | 1shs_F | 0.865 | 0.030 |
| 746 | 132 | 27 | 1shs−1gme | 1shs_G | 0.865 | 0.031 |
| 746 | 132 | 27 | 1shs−1gme | 1shs_B | 0.865 | 0.028 |
| 746 | 132 | 27 | 1shs−1gme | 1shs_C | 0.865 | 0.027 |
| 751 | 91 | 27 | 1ig3−1ig0 | 1ig3_B | 0.929 | 0.158 |
| 751 | 91 | 27 | 1ig0−1ig3 | 1ig0_B | 1.000 | 0.217 |
| 752 | 182 | 27 | 1etp−1fcd | 1m6z_D | 0.994 | 0.501 |
| 752 | 182 | 27 | 1etp−1fcd | 1m6z_A | 1.000 | 0.620 |
| 752 | 182 | 27 | 1etp−1fcd | 1m70_A | 1.000 | 0.672 |
| 752 | 182 | 27 | 1etp−1fcd | 1m70_D | 0.994 | 0.504 |
| 752 | 182 | 27 | 1etp−1fcd | 1m70_B | 1.000 | 0.407 |
| 752 | 182 | 27 | 1etp−1fcd | 1etp_B | 1.000 | 0.477 |
| 752 | 182 | 27 | 1etp−1fcd | 1m6z_B | 0.994 | 0.358 |
| 752 | 182 | 27 | 1fcd−1etp | 1fcd_D | 0.994 | 0.552 |
| 752 | 182 | 27 | 1etp−1fcd | 1m6z_C | 1.000 | 0.511 |
| 752 | 182 | 27 | 1etp−1fcd | 1m70_C | 1.000 | 0.522 |
| 753 | 181 | 27 | 1e32−1cz5 | 1s3s_F | 0.970 | 0.452 |
| 753 | 181 | 27 | 1cz5−1e32 | 1cz4_A | 0.946 | 3.042 |
| 757 | 98 | 26 | 1exh−2xbd | 1exg_A | 1.000 | 0.405 |
| 761 | 154 | 26 | 1qgh−1dps | 1qgh_B | 0.993 | 0.160 |
| 761 | 154 | 26 | 1qgh−1dps | 1qgh_J | 0.993 | 0.141 |
| 761 | 154 | 26 | 1qgh−1dps | 1qgh_I | 0.993 | 0.173 |
| 761 | 154 | 26 | 1qgh−1dps | 1qgh_F | 0.993 | 0.144 |
| 761 | 154 | 26 | 1qgh−1dps | 1qgh_H | 0.993 | 0.128 |
| 761 | 154 | 26 | 1qgh−1dps | 1qgh_K | 0.993 | 0.174 |
| 761 | 154 | 26 | 1qgh−1dps | 1qgh_D | 1.000 | 0.202 |
| 761 | 154 | 26 | 1qgh−1dps | 1qgh_E | 0.993 | 0.141 |
| 761 | 154 | 26 | 1qgh−1dps | 1qgh_G | 0.993 | 0.110 |
| 761 | 154 | 26 | 1qgh−1dps | 1qgh_C | 0.993 | 0.134 |
| 761 | 154 | 26 | 1qgh−1dps | 1qgh_L | 0.986 | 0.176 |
| 763 | 231 | 26 | 1wod−1atg | 1amf_A | 0.960 | 0.065 |
| 764 | 241 | 26 | 1mml−1vrt | 1qai_B | 1.000 | 2.250 |
| 764 | 241 | 26 | 1vrt−1mml | 1rt1_A | 0.947 | 1.579 |
| 764 | 241 | 26 | 1vrt−1mml | 1rt7_A | 0.921 | 1.529 |
| 764 | 241 | 26 | 1vrt−1mml | 1dtq_A | 0.991 | 0.606 |
| 764 | 241 | 26 | 1vrt−1mml | 1rti_A | 0.978 | 1.253 |
| 764 | 241 | 26 | 1vrt−1mml | 1rt6_A | 0.938 | 1.500 |
| 764 | 241 | 26 | 1vrt−1mml | 1rtj_A | 0.943 | 1.526 |
| 764 | 241 | 26 | 1vrt−1mml | 1c0u_A | 0.934 | 1.514 |
| 764 | 241 | 26 | 1vrt−1mml | 1rev_A | 1.000 | 0.604 |
| 764 | 241 | 26 | 1vrt−1mml | 1rt2_A | 0.925 | 1.533 |
| 764 | 241 | 26 | 1vrt−1mml | 1rth_A | 0.947 | 1.356 |

| n | length | %ID | refA−refB | altA | SP | RMSD |
|---|---|---|---|---|---|---|
| 764 | 241 | 26 | 1vrt−1mml | 1vru_A | 1.000 | 0.897 |
| 764 | 241 | 26 | 1vrt−1mml | 1klm_A | 0.934 | 1.515 |
| 764 | 241 | 26 | 1mml−1vrt | 1qai_A | 0.952 | 1.607 |
| 767 | 82 | 26 | 1fip−1ntc | 1fip_B | 0.722 | 0.453 |
| 768 | 247 | 26 | 1gz0−1ipa | 1gz0_F | 0.710 | 0.867 |
| 768 | 247 | 26 | 1gz0−1ipa | 1gz0_H | 0.959 | 1.426 |
| 768 | 247 | 26 | 1gz0−1ipa | 1gz0_B | 0.787 | 2.157 |
| 768 | 247 | 26 | 1gz0−1ipa | 1gz0_D | 0.710 | 2.416 |
| 768 | 247 | 26 | 1gz0−1ipa | 1gz0_C | 0.923 | 0.481 |
| 773 | 85 | 26 | 1i42−1h8c | 1jru_A | 0.688 | 1.166 |
| 775 | 246 | 26 | 1ca1−1ah7 | 1gyg_A | 0.900 | 3.628 |
| 775 | 246 | 26 | 1ca1−1ah7 | 1gyg_B | 0.900 | 3.648 |
| 777 | 86 | 25 | 1fs0−1e79 | 1bsn_A | 1.000 | 3.696 |
| 777 | 86 | 25 | 1e79−1fs0 | 1h8e_H | 1.000 | 0.299 |
| 777 | 86 | 25 | 1e79−1fs0 | 2v7q_H | 1.000 | 0.542 |
| 778 | 512 | 25 | 1jet−1dpe | 1jev_A | 0.996 | 0.165 |
| 778 | 512 | 25 | 1jet−1dpe | 1b32_A | 0.979 | 0.130 |
| 778 | 512 | 25 | 1jet−1dpe | 1b3f_A | 0.996 | 0.215 |
| 778 | 512 | 25 | 1jet−1dpe | 1b3l_A | 0.979 | 0.450 |
| 778 | 512 | 25 | 1jet−1dpe | 2rkm_A | 0.998 | 0.401 |
| 778 | 512 | 25 | 1jet−1dpe | 1b4z_A | 0.979 | 0.139 |
| 778 | 512 | 25 | 1jet−1dpe | 1qkb_A | 0.975 | 0.137 |
| 778 | 512 | 25 | 1jet−1dpe | 1b5i_A | 0.996 | 0.170 |
| 778 | 512 | 25 | 1jet−1dpe | 1b0h_A | 0.998 | 0.252 |
| 778 | 512 | 25 | 1jet−1dpe | 1b05_A | 0.979 | 0.171 |
| 778 | 512 | 25 | 1jet−1dpe | 2olb_A | 0.970 | 0.177 |
| 778 | 512 | 25 | 1jet−1dpe | 1b3g_A | 0.979 | 0.212 |
| 778 | 512 | 25 | 1jet−1dpe | 1b3h_A | 0.996 | 0.227 |
| 778 | 512 | 25 | 1jet−1dpe | 1b7h_A | 0.996 | 0.341 |
| 778 | 512 | 25 | 1jet−1dpe | 1b46_A | 0.985 | 0.154 |
| 778 | 512 | 25 | 1jet−1dpe | 1b9j_A | 0.979 | 0.203 |
| 778 | 512 | 25 | 1jet−1dpe | 1b5j_A | 0.981 | 0.159 |
| 778 | 512 | 25 | 1jet−1dpe | 1b1h_A | 0.979 | 0.245 |
| 778 | 512 | 25 | 1jet−1dpe | 1rkm_A | 0.979 | 3.154 |
| 778 | 512 | 25 | 1jet−1dpe | 1b51_A | 0.998 | 0.188 |
| 778 | 512 | 25 | 1jet−1dpe | 1b3l_C | 0.985 | 0.535 |
| 778 | 512 | 25 | 1jet−1dpe | 1b40_A | 0.979 | 0.224 |
| 778 | 512 | 25 | 1jet−1dpe | 1b4h_A | 0.998 | 0.148 |
| 778 | 512 | 25 | 1jet−1dpe | 1b5h_A | 0.979 | 0.128 |
| 778 | 512 | 25 | 1jet−1dpe | 1b6h_A | 0.998 | 0.131 |
| 778 | 512 | 25 | 1jet−1dpe | 1b2h_A | 0.977 | 0.179 |
| 778 | 512 | 25 | 1jet−1dpe | 1qka_A | 0.996 | 0.177 |
| 778 | 512 | 25 | 1jet−1dpe | 1jeu_A | 0.994 | 0.366 |
| 778 | 512 | 25 | 1jet−1dpe | 1olc_A | 0.996 | 0.318 |
| 778 | 512 | 25 | 1jet−1dpe | 1b58_A | 0.985 | 0.334 |
| 778 | 512 | 25 | 1jet−1dpe | 1ola_A | 0.996 | 0.505 |
| 780 | 214 | 25 | 1jdi−1fua | 1jdi_D | 0.990 | 0.042 |
| 780 | 214 | 25 | 1jdi−1fua | 1jdi_E | 0.990 | 0.042 |
| 780 | 214 | 25 | 1fua−1jdi | 2fua_A | 0.990 | 0.151 |
| 780 | 214 | 25 | 1fua−1jdi | 4fua_A | 0.975 | 0.577 |
| 780 | 214 | 25 | 1jdi−1fua | 1jdi_F | 1.000 | 0.037 |
| 780 | 214 | 25 | 1fua−1jdi | 1dzy_P | 0.985 | 0.170 |
| 780 | 214 | 25 | 1jdi−1fua | 1jdi_B | 0.990 | 0.038 |
| 780 | 214 | 25 | 1fua−1jdi | 3fua_A | 0.990 | 0.489 |
| 780 | 214 | 25 | 1jdi−1fua | 1jdi_C | 0.990 | 0.040 |
| 782 | 325 | 25 | 1b63−1h7s | 1nhi_A | 0.975 | 0.223 |
| 785 | 369 | 25 | 2tmd−1oyc | 1o94_B | 0.976 | 0.182 |
| 785 | 369 | 25 | 2tmd−1oyc | 1o95_A | 0.948 | 0.278 |
| 785 | 369 | 25 | 2tmd−1oyc | 1djn_A | 1.000 | 0.137 |
| 785 | 369 | 25 | 2tmd−1oyc | 1o94_A | 0.979 | 0.178 |
| 785 | 369 | 25 | 2tmd−1oyc | 1o95_B | 0.945 | 0.289 |
| 785 | 369 | 25 | 2tmd−1oyc | 1djn_B | 0.970 | 0.155 |
| 785 | 369 | 25 | 1oyc−2tmd | 1oya_A | 0.951 | 0.142 |
| 785 | 369 | 25 | 2tmd−1oyc | 2tmd_B | 0.963 | 0.141 |
| 785 | 369 | 25 | 1oyc−2tmd | 1oyb_A | 0.970 | 0.178 |
| 786 | 112 | 25 | 1ew4−1ekg | 2p1x_A | 1.000 | 0.799 |
| 786 | 112 | 25 | 1ew4−1ekg | 2eff_A | 1.000 | 0.673 |
| 787 | 298 | 25 | 1bgl−1bhg | 1bgm_O | 0.965 | 0.276 |
| 787 | 298 | 25 | 1bgl−1bhg | 1bgl_D | 1.000 | 0.283 |
| 787 | 298 | 25 | 1bgl−1bhg | 1gho_L | 1.000 | 0.258 |
| 787 | 298 | 25 | 1bgl−1bhg | 1f49_H | 0.958 | 0.258 |

n: internal numbering; length of alignment; % sequence ID (cf. Homstrad); refA−refB: reference alignment; altA: alternative for struc. A; SP: sum−of−pairs score of alt. alignment w/r reference

| n | length | %ID | refA−refB | altA | SP | RMSD |
|---|---|---|---|---|---|---|
| 787 | 298 | 25 | 1bgl−1bhg | 1gho_M | 0.977 | 0.258 |
| 787 | 298 | 25 | 1bgl−1bhg | 1f49_D | 0.958 | 0.258 |
| 787 | 298 | 25 | 1bgl−1bhg | 1bgm_K | 0.969 | 0.347 |
| 787 | 298 | 25 | 1bgl−1bhg | 1gho_O | 0.969 | 0.258 |
| 787 | 298 | 25 | 1bgl−1bhg | 1f49_F | 0.977 | 0.258 |
| 787 | 298 | 25 | 1bgl−1bhg | 1bgl_B | 0.985 | 0.268 |
| 787 | 298 | 25 | 1bgl−1bhg | 1gho_P | 0.992 | 0.258 |
| 787 | 298 | 25 | 1bgl−1bhg | 1bgm_I | 1.000 | 0.251 |
| 787 | 298 | 25 | 1bgl−1bhg | 1bgm_N | 1.000 | 0.295 |
| 787 | 298 | 25 | 1bgl−1bhg | 1f49_C | 0.965 | 0.258 |
| 787 | 298 | 25 | 1bgl−1bhg | 1bgl_F | 0.977 | 0.311 |
| 787 | 298 | 25 | 1bgl−1bhg | 1bgl_G | 0.969 | 0.254 |
| 787 | 298 | 25 | 1bgl−1bhg | 1bgm_J | 1.000 | 0.321 |
| 787 | 298 | 25 | 1bgl−1bhg | 1f49_G | 0.977 | 0.258 |
| 787 | 298 | 25 | 1bgl−1bhg | 1jz1_P | 0.992 | 0.299 |
| 787 | 298 | 25 | 1bgl−1bhg | 1jz2_B | 1.000 | 0.342 |
| 787 | 298 | 25 | 1bgl−1bhg | 1bgl_C | 0.965 | 0.401 |
| 787 | 298 | 25 | 1bgl−1bhg | 1f49_E | 0.992 | 0.258 |
| 787 | 298 | 25 | 1bhg−1bgl | 1bhg_B | 0.938 | 0.465 |
| 787 | 298 | 25 | 1bgl−1bhg | 1gho_K | 1.000 | 0.258 |
| 787 | 298 | 25 | 1bgl−1bhg | 1jz1_O | 1.000 | 0.299 |
| 787 | 298 | 25 | 1bgl−1bhg | 1f49_A | 0.977 | 0.258 |
| 787 | 298 | 25 | 1bgl−1bhg | 1bgm_P | 0.977 | 0.361 |
| 787 | 298 | 25 | 1bgl−1bhg | 1gho_J | 0.938 | 0.258 |
| 787 | 298 | 25 | 1bgl−1bhg | 1jz2_C | 1.000 | 0.371 |
| 787 | 298 | 25 | 1bgl−1bhg | 1bgl_E | 0.985 | 0.261 |
| 787 | 298 | 25 | 1bgl−1bhg | 1bgm_L | 0.973 | 0.274 |
| 787 | 298 | 25 | 1bgl−1bhg | 1gho_I | 0.969 | 0.258 |
| 787 | 298 | 25 | 1bgl−1bhg | 1jz2_A | 0.965 | 0.342 |
| 787 | 298 | 25 | 1bgl−1bhg | 1bgl_H | 0.977 | 0.264 |
| 787 | 298 | 25 | 1bgl−1bhg | 1bgm_M | 1.000 | 0.313 |
| 787 | 298 | 25 | 1bgl−1bhg | 1f49_B | 1.000 | 0.258 |
| 787 | 298 | 25 | 1bgl−1bhg | 1jz2_D | 0.969 | 0.350 |
| 787 | 298 | 25 | 1bgl−1bhg | 1jz1_K | 1.000 | 0.299 |
| 787 | 298 | 25 | 1bgl−1bhg | 1jz1_L | 0.981 | 0.299 |
| 787 | 298 | 25 | 1bgl−1bhg | 1gho_N | 0.969 | 0.258 |
| 787 | 298 | 25 | 1bgl−1bhg | 1jz1_M | 0.969 | 0.299 |
| 787 | 298 | 25 | 1bgl−1bhg | 1jz1_N | 0.969 | 0.299 |
| 787 | 298 | 25 | 1bgl−1bhg | 1jyy_A | 0.992 | 0.280 |
| 787 | 298 | 25 | 1bgl−1bhg | 1jyy_B | 0.969 | 0.280 |
| 787 | 298 | 25 | 1bgl−1bhg | 1jyy_C | 0.977 | 0.280 |
| 787 | 298 | 25 | 1bgl−1bhg | 1jyy_D | 0.969 | 0.280 |
| 787 | 298 | 25 | 1bgl−1bhg | 1jyy_E | 0.969 | 0.280 |
| 787 | 298 | 25 | 1bgl−1bhg | 1jyy_F | 0.992 | 0.280 |
| 787 | 298 | 25 | 1bgl−1bhg | 1jyy_G | 0.969 | 0.280 |
| 787 | 298 | 25 | 1bgl−1bhg | 1jyy_H | 1.000 | 0.280 |
| 787 | 298 | 25 | 1bgl−1bhg | 1jyz_I | 0.977 | 0.280 |
| 787 | 298 | 25 | 1bgl−1bhg | 1jyz_J | 0.961 | 0.280 |
| 787 | 298 | 25 | 1bgl−1bhg | 1jyz_K | 0.977 | 0.280 |
| 787 | 298 | 25 | 1bgl−1bhg | 1jyz_L | 1.000 | 0.280 |
| 787 | 298 | 25 | 1bgl−1bhg | 1jyz_M | 0.958 | 0.280 |
| 787 | 298 | 25 | 1bgl−1bhg | 1jyz_N | 0.969 | 0.280 |
| 787 | 298 | 25 | 1bgl−1bhg | 1jyz_O | 0.977 | 0.280 |
| 787 | 298 | 25 | 1bgl−1bhg | 1jyz_P | 0.961 | 0.280 |
| 787 | 298 | 25 | 1bgl−1bhg | 1jz0_A | 0.977 | 0.299 |
| 787 | 298 | 25 | 1bgl−1bhg | 1jz0_B | 0.969 | 0.299 |
| 787 | 298 | 25 | 1bgl−1bhg | 1jz0_C | 0.965 | 0.299 |
| 787 | 298 | 25 | 1bgl−1bhg | 1jz0_D | 0.992 | 0.299 |
| 787 | 298 | 25 | 1bgl−1bhg | 1jz0_E | 0.965 | 0.299 |
| 787 | 298 | 25 | 1bgl−1bhg | 1jz0_F | 1.000 | 0.299 |
| 787 | 298 | 25 | 1bgl−1bhg | 1jz0_G | 0.965 | 0.299 |
| 787 | 298 | 25 | 1bgl−1bhg | 1jz0_H | 0.992 | 0.299 |
| 787 | 298 | 25 | 1bgl−1bhg | 1jz1_I | 0.977 | 0.299 |
| 787 | 298 | 25 | 1bgl−1bhg | 1jz1_J | 0.977 | 0.299 |
| 794 | 91 | 25 | 1flt−1pdg | 1flt_W | 1.000 | 0.640 |
| 797 | 353 | 25 | 1k4g−1iq8 | 1q2r_B | 0.982 | 0.544 |
| 797 | 353 | 25 | 1iq8−1k4g | 1it8_A | 0.991 | 0.280 |
| 797 | 353 | 25 | 1k4g−1iq8 | 1n2v_A | 0.997 | 0.243 |
| 797 | 353 | 25 | 1iq8−1k4g | 1it7_B | 0.988 | 0.371 |
| 797 | 353 | 25 | 1k4g−1iq8 | 1q2s_D | 0.985 | 0.418 |
| 797 | 353 | 25 | 1k4g−1iq8 | 1q2s_A | 0.991 | 0.802 |

| n | length | %ID | refA−refB | altA | SP | RMSD |
|---|---|---|---|---|---|---|
| 797 | 353 | 25 | 1k4g−1iq8 | 1f3e_A | 1.000 | 0.157 |
| 797 | 353 | 25 | 1k4g−1iq8 | 1q2s_C | 0.991 | 0.802 |
| 797 | 353 | 25 | 1k4g−1iq8 | 1q2r_A | 0.991 | 0.815 |
| 797 | 353 | 25 | 1k4g−1iq8 | 1q2r_D | 0.982 | 0.529 |
| 797 | 353 | 25 | 1k4g−1iq8 | 1pud_A | 0.973 | 0.382 |
| 797 | 353 | 25 | 1iq8−1k4g | 1it8_B | 1.000 | 0.389 |
| 797 | 353 | 25 | 1k4g−1iq8 | 1enu_A | 0.982 | 0.340 |
| 797 | 353 | 25 | 1k4g−1iq8 | 1k4h_A | 0.988 | 0.105 |
| 797 | 353 | 25 | 1iq8−1k4g | 1iq8_B | 0.988 | 0.350 |
| 797 | 353 | 25 | 1k4g−1iq8 | 1q2s_B | 0.985 | 0.445 |
| 797 | 353 | 25 | 1k4g−1iq8 | 1q2r_C | 0.991 | 0.816 |
| 797 | 353 | 25 | 1iq8−1k4g | 1it7_A | 0.997 | 0.254 |
| 800 | 369 | 25 | 1l8a−1trk | 1rp7_B | 0.963 | 0.452 |
| 800 | 369 | 25 | 1l8a−1trk | 2g28_A | 0.959 | 0.257 |
| 800 | 369 | 25 | 1l8a−1trk | 2g67_B | 0.983 | 0.497 |
| 800 | 369 | 25 | 1l8a−1trk | 2g28_B | 0.963 | 0.361 |
| 800 | 369 | 25 | 1l8a−1trk | 2g67_A | 0.993 | 0.414 |
| 800 | 369 | 25 | 1trk−1l8a | 1gpu_A | 0.980 | 0.278 |
| 800 | 369 | 25 | 1trk−1l8a | 1gpu_B | 0.973 | 0.292 |
| 800 | 369 | 25 | 1trk−1l8a | 1trk_B | 1.000 | 0.190 |
| 800 | 369 | 25 | 1l8a−1trk | 1l8a_B | 0.963 | 0.460 |
| 800 | 369 | 25 | 1l8a−1trk | 2iea_A | 0.990 | 0.099 |
| 800 | 369 | 25 | 1l8a−1trk | 2iea_B | 0.963 | 0.478 |
| 800 | 369 | 25 | 1trk−1l8a | 1ngs_A | 0.993 | 0.174 |
| 800 | 369 | 25 | 1l8a−1trk | 1rp7_A | 1.000 | 0.199 |
| 800 | 369 | 25 | 1trk−1l8a | 1ngs_B | 1.000 | 0.184 |
| 801 | 93 | 25 | 1e32−1cz5 | 1s3s_F | 1.000 | 0.474 |
| 801 | 93 | 25 | 1cz5−1e32 | 1cz4_A | 1.000 | 1.844 |
| 808 | 291 | 24 | 1dhp−1nal | 1yxd_A | 0.986 | 0.253 |
| 808 | 291 | 24 | 1dhp−1nal | 1dhp_B | 0.986 | 0.208 |
| 808 | 291 | 24 | 1dhp−1nal | 2ats_B | 0.986 | 0.297 |
| 808 | 291 | 24 | 1dhp−1nal | 1yxd_B | 0.986 | 0.284 |
| 808 | 291 | 24 | 1nal−1dhp | 1nal_3 | 1.000 | 0.279 |
| 808 | 291 | 24 | 1nal−1dhp | 1nal_4 | 0.997 | 0.236 |
| 808 | 291 | 24 | 1dhp−1nal | 1yxc_B | 0.986 | 0.278 |
| 808 | 291 | 24 | 1dhp−1nal | 2ats_A | 1.000 | 0.260 |
| 808 | 291 | 24 | 1nal−1dhp | 1nal_2 | 0.997 | 0.265 |
| 808 | 291 | 24 | 1dhp−1nal | 1yxc_A | 1.000 | 0.245 |
| 813 | 144 | 24 | 1ofg−1gcu | 1ofg_D | 1.000 | 0.001 |
| 813 | 144 | 24 | 1ofg−1gcu | 1ofg_E | 1.000 | 0.001 |
| 813 | 144 | 24 | 1ofg−1gcu | 1ofg_F | 1.000 | 0.001 |
| 813 | 144 | 24 | 1ofg−1gcu | 1ofg_B | 1.000 | 0.001 |
| 813 | 144 | 24 | 1ofg−1gcu | 1ofg_C | 1.000 | 0.001 |
| 816 | 269 | 24 | 1egu−1cb8 | 1ojp_A | 0.996 | 0.096 |
| 816 | 269 | 24 | 1egu−1cb8 | 1w3y_A | 1.000 | 0.096 |
| 816 | 269 | 24 | 1egu−1cb8 | 2brw_A | 0.992 | 0.220 |
| 816 | 269 | 24 | 1egu−1cb8 | 1c82_A | 0.996 | 0.100 |
| 816 | 269 | 24 | 1egu−1cb8 | 2brp_A | 1.000 | 0.091 |
| 816 | 269 | 24 | 1egu−1cb8 | 2brv_X | 0.992 | 0.432 |
| 816 | 269 | 24 | 1egu−1cb8 | 2brw_B | 0.992 | 0.225 |
| 816 | 269 | 24 | 1egu−1cb8 | 1f9g_A | 1.000 | 0.169 |
| 817 | 146 | 24 | 1vsd−1biz | 1vsf_A | 0.968 | 0.229 |
| 817 | 146 | 24 | 1vsd−1biz | 1vse_A | 1.000 | 0.264 |
| 818 | 209 | 24 | 1jj2−1dmg | 1k73_E | 1.000 | 0.046 |
| 818 | 209 | 24 | 1jj2−1dmg | 1k8a_E | 0.986 | 0.093 |
| 818 | 209 | 24 | 1jj2−1dmg | 1k9m_E | 1.000 | 0.084 |
| 818 | 209 | 24 | 1jj2−1dmg | 1kc8_E | 0.986 | 0.071 |
| 818 | 209 | 24 | 1jj2−1dmg | 1kd1_E | 1.000 | 0.087 |
| 818 | 209 | 24 | 1jj2−1dmg | 1kqs_C | 1.000 | 0.087 |
| 818 | 209 | 24 | 1jj2−1dmg | 1m1k_E | 0.986 | 0.090 |
| 818 | 209 | 24 | 1jj2−1dmg | 1m90_E | 0.986 | 0.067 |
| 818 | 209 | 24 | 1jj2−1dmg | 1n8r_E | 1.000 | 0.094 |
| 818 | 209 | 24 | 1jj2−1dmg | 1nji_E | 1.000 | 0.065 |
| 818 | 209 | 24 | 1jj2−1dmg | 1q7y_E | 1.000 | 0.157 |
| 818 | 209 | 24 | 1jj2−1dmg | 1q81_E | 0.986 | 0.146 |
| 818 | 209 | 24 | 1jj2−1dmg | 1q82_E | 0.986 | 0.096 |
| 818 | 209 | 24 | 1jj2−1dmg | 1q86_E | 0.986 | 0.117 |
| 818 | 209 | 24 | 1jj2−1dmg | 1qvf_C | 0.986 | 0.058 |
| 818 | 209 | 24 | 1jj2−1dmg | 1qvg_C | 0.986 | 0.094 |
| 818 | 209 | 24 | 1jj2−1dmg | 1s72_C | 1.000 | 0.023 |
| 818 | 209 | 24 | 1jj2−1dmg | 1vq4_C | 1.000 | 0.161 |

| n | length | %ID | refA−refB | altA | SP | RMSD |
|---|---|---|---|---|---|---|
| 818 | 209 | 24 | 1jj2−1dmg | 1vq5_C | 1.000 | 0.145 |
| 818 | 209 | 24 | 1jj2−1dmg | 1vq6_C | 0.986 | 0.169 |
| 818 | 209 | 24 | 1jj2−1dmg | 1vq7_C | 0.986 | 0.149 |
| 818 | 209 | 24 | 1jj2−1dmg | 1vq8_C | 0.986 | 0.173 |
| 818 | 209 | 24 | 1jj2−1dmg | 1vq9_C | 1.000 | 0.188 |
| 818 | 209 | 24 | 1jj2−1dmg | 1vqk_C | 1.000 | 0.153 |
| 818 | 209 | 24 | 1jj2−1dmg | 1vql_C | 0.986 | 0.167 |
| 818 | 209 | 24 | 1jj2−1dmg | 1vqm_C | 0.986 | 0.169 |
| 818 | 209 | 24 | 1jj2−1dmg | 1vqn_C | 0.986 | 0.164 |
| 818 | 209 | 24 | 1jj2−1dmg | 1vqo_C | 0.986 | 0.146 |
| 818 | 209 | 24 | 1jj2−1dmg | 1vqp_C | 0.986 | 0.147 |
| 818 | 209 | 24 | 1jj2−1dmg | 1w2b_C | 1.000 | 0.217 |
| 818 | 209 | 24 | 1jj2−1dmg | 1yhq_C | 1.000 | 0.119 |
| 818 | 209 | 24 | 1jj2−1dmg | 1yi2_C | 0.986 | 0.120 |
| 818 | 209 | 24 | 1jj2−1dmg | 1yij_C | 0.986 | 0.142 |
| 818 | 209 | 24 | 1jj2−1dmg | 1yit_C | 0.986 | 0.171 |
| 818 | 209 | 24 | 1jj2−1dmg | 1yj9_C | 0.986 | 0.227 |
| 818 | 209 | 24 | 1jj2−1dmg | 1yjn_C | 0.986 | 0.188 |
| 818 | 209 | 24 | 1jj2−1dmg | 1yjw_C | 0.986 | 0.188 |
| 818 | 209 | 24 | 1jj2−1dmg | 2otj_C | 0.986 | 0.150 |
| 818 | 209 | 24 | 1jj2−1dmg | 2otl_C | 0.986 | 0.161 |
| 819 | 168 | 24 | 1jj2−1rl6 | 1n8r_G | 1.000 | 0.087 |
| 819 | 168 | 24 | 1jj2−1rl6 | 1m90_G | 1.000 | 0.054 |
| 819 | 168 | 24 | 1jj2−1rl6 | 1nji_G | 1.000 | 0.051 |
| 819 | 168 | 24 | 1jj2−1rl6 | 1q86_G | 1.000 | 0.117 |
| 819 | 168 | 24 | 1jj2−1rl6 | 1k9m_G | 1.000 | 0.058 |
| 819 | 168 | 24 | 1jj2−1rl6 | 1q81_G | 1.000 | 0.122 |
| 819 | 168 | 24 | 1rl6−1jj2 | 1yl3_H | 0.938 | 2.561 |
| 819 | 168 | 24 | 1jj2−1rl6 | 1kc8_G | 1.000 | 0.051 |
| 819 | 168 | 24 | 1jj2−1rl6 | 1qvf_E | 1.000 | 0.053 |
| 819 | 168 | 24 | 1jj2−1rl6 | 1k73_G | 1.000 | 0.034 |
| 819 | 168 | 24 | 1jj2−1rl6 | 1m1k_G | 1.000 | 0.082 |
| 819 | 168 | 24 | 1rl6−1jj2 | 2b9n_H | 0.938 | 2.577 |
| 819 | 168 | 24 | 1jj2−1rl6 | 1q7y_G | 1.000 | 0.121 |
| 819 | 168 | 24 | 1jj2−1rl6 | 1qvg_E | 1.000 | 0.127 |
| 819 | 168 | 24 | 1jj2−1rl6 | 1kd1_G | 1.000 | 0.064 |
| 819 | 168 | 24 | 1rl6−1jj2 | 2b9p_H | 0.938 | 2.562 |
| 819 | 168 | 24 | 1jj2−1rl6 | 1w2b_E | 1.000 | 0.215 |
| 819 | 168 | 24 | 1jj2−1rl6 | 1kqs_E | 1.000 | 0.084 |
| 819 | 168 | 24 | 1jj2−1rl6 | 1q82_G | 1.000 | 0.076 |
| 819 | 168 | 24 | 1rl6−1jj2 | 2b66_H | 0.938 | 2.607 |
| 819 | 168 | 24 | 1jj2−1rl6 | 1k8a_G | 1.000 | 0.064 |
| 819 | 168 | 24 | 1rl6−1jj2 | 1c04_B | 1.000 | 2.286 |
| 821 | 326 | 24 | 1gln−1qtq | 1g59_A | 0.989 | 1.139 |
| 821 | 326 | 24 | 1qtq−1gln | 1gtr_A | 0.978 | 0.238 |
| 821 | 326 | 24 | 1qtq−1gln | 1zjw_A | 0.974 | 0.185 |
| 821 | 326 | 24 | 1gln−1qtq | 1g59_C | 0.989 | 1.037 |
| 821 | 326 | 24 | 1qtq−1gln | 1gts_A | 0.956 | 0.319 |
| 823 | 597 | 24 | 1f7u−1iq0 | 1f7v_A | 0.948 | 1.420 |
| 830 | 137 | 23 | 1k6k−1khy | 1r6o_A | 1.000 | 1.608 |
| 830 | 137 | 23 | 1k6k−1khy | 1r6c_X | 1.000 | 0.472 |
| 830 | 137 | 23 | 1k6k−1khy | 1r6q_A | 1.000 | 1.618 |
| 830 | 137 | 23 | 1khy−1k6k | 1khy_B | 1.000 | 0.451 |
| 830 | 137 | 23 | 1k6k−1khy | 1r6q_B | 1.000 | 1.547 |
| 831 | 267 | 23 | 1poi−1k6d | 1poi_C | 1.000 | 0.295 |
| 832 | 276 | 23 | 1eg2−1boo | 1eg2_A | 1.000 | 0.008 |
| 832 | 276 | 23 | 1eg2−1boo | 1nw5_A | 0.979 | 0.158 |
| 836 | 248 | 23 | 1chk−1qgi | 1chk_B | 0.969 | 0.800 |
| 837 | 450 | 23 | 1tf4−1clc | 1tf4_A | 0.968 | 0.082 |
| 837 | 450 | 23 | 1tf4−1clc | 1js4_A | 0.968 | 0.122 |
| 837 | 450 | 23 | 1tf4−1clc | 4tf4_B | 0.968 | 0.217 |
| 837 | 450 | 23 | 1tf4−1clc | 3tf4_B | 0.988 | 0.216 |
| 837 | 450 | 23 | 1tf4−1clc | 1js4_B | 0.968 | 0.136 |
| 837 | 450 | 23 | 1tf4−1clc | 3tf4_A | 0.978 | 0.223 |
| 837 | 450 | 23 | 1tf4−1clc | 4tf4_A | 0.968 | 0.238 |
| 840 | 147 | 23 | 1a6j−1a3a | 1a6j_B | 0.993 | 0.695 |
| 840 | 147 | 23 | 1a3a−1a6j | 1a3a_C | 1.000 | 0.753 |
| 844 | 324 | 23 | 1bx4−1rkd | 2i6a_C | 0.902 | 5.241 |
| 844 | 324 | 23 | 1rkd−1bx4 | 1gqt_C | 0.963 | 0.548 |
| 844 | 324 | 23 | 1bx4−1rkd | 2i6a_A | 0.902 | 5.271 |
| 844 | 324 | 23 | 1bx4−1rkd | 2i6a_D | 0.881 | 5.222 |

n: internal numbering; length of alignment; % sequence ID (cf. Homstrad); refA−refB: reference alignment; altA: alternative for struc. A; SP: sum−of−pairs score of alt. alignment w/r reference

| n | length | %ID | refA−refB | altA | SP | RMSD |
|---|---|---|---|---|---|---|
| 844 | 324 | 23 | 1rkd−1bx4 | 1rk2_C | 1.000 | 0.488 |
| 844 | 324 | 23 | 1bx4−1rkd | 2i6a_B | 0.902 | 5.267 |
| 845 | 129 | 23 | 1jj2−1bxe | 1k73_S | 1.000 | 0.044 |
| 845 | 129 | 23 | 1bxe−1jj2 | 2b66_W | 0.981 | 1.194 |
| 845 | 129 | 23 | 1jj2−1bxe | 1k8a_S | 1.000 | 0.092 |
| 845 | 129 | 23 | 1bxe−1jj2 | 2b9p_W | 0.981 | 1.230 |
| 845 | 129 | 23 | 1jj2−1bxe | 1k9m_S | 1.000 | 0.100 |
| 845 | 129 | 23 | 1jj2−1bxe | 1kc8_S | 1.000 | 0.065 |
| 845 | 129 | 23 | 1jj2−1bxe | 1kd1_S | 1.000 | 0.108 |
| 845 | 129 | 23 | 1bxe−1jj2 | 1yl3_S | 0.981 | 1.122 |
| 845 | 129 | 23 | 1jj2−1bxe | 1kqs_Q | 1.000 | 0.085 |
| 845 | 129 | 23 | 1bxe−1jj2 | 2b9n_W | 0.981 | 1.263 |
| 845 | 129 | 23 | 1jj2−1bxe | 1m1k_S | 1.000 | 0.088 |
| 845 | 129 | 23 | 1jj2−1bxe | 1m90_S | 1.000 | 0.067 |
| 845 | 129 | 23 | 1jj2−1bxe | 1n8r_S | 1.000 | 0.099 |
| 845 | 129 | 23 | 1jj2−1bxe | 1nji_S | 1.000 | 0.057 |
| 845 | 129 | 23 | 1jj2−1bxe | 1q7y_S | 1.000 | 0.146 |
| 845 | 129 | 23 | 1jj2−1bxe | 1q81_S | 1.000 | 0.154 |
| 845 | 129 | 23 | 1jj2−1bxe | 1q82_S | 1.000 | 0.088 |
| 845 | 129 | 23 | 1jj2−1bxe | 1q86_S | 1.000 | 0.116 |
| 845 | 129 | 23 | 1jj2−1bxe | 1qvf_Q | 1.000 | 0.055 |
| 845 | 129 | 23 | 1jj2−1bxe | 1qvg_Q | 1.000 | 0.130 |
| 845 | 129 | 23 | 1jj2−1bxe | 1w2b_Q | 1.000 | 0.217 |
| 847 | 358 | 23 | 1f7u−1iq0 | 1bs2_A | 0.889 | 2.690 |
| 847 | 358 | 23 | 1f7u−1iq0 | 1f7v_A | 0.914 | 1.715 |
| 849 | 137 | 23 | 1ak4−2eia | 1ak4_D | 0.968 | 1.126 |
| 849 | 137 | 23 | 2eia−1ak4 | 2eia_B | 0.968 | 0.379 |
| 850 | 265 | 22 | 1xo1−1bgx | 1xo1_B | 0.816 | 0.237 |
| 851 | 445 | 22 | 1l5j−1c96 | 1l5j_B | 0.970 | 0.214 |
| 857 | 286 | 22 | 1fo6−1ems | 1fo6_C | 0.958 | 0.421 |
| 857 | 286 | 22 | 1fo6−1ems | 1fo6_B | 0.977 | 0.438 |
| 857 | 286 | 22 | 1ems−1fo6 | 1ems_B | 0.939 | 1.009 |
| 857 | 286 | 22 | 1fo6−1ems | 1fo6_D | 0.977 | 0.243 |
| 858 | 180 | 22 | 1bpy−1jqr | 1zqj_A | 0.916 | 2.164 |
| 858 | 180 | 22 | 1bpy−1jqr | 7icj_A | 0.952 | 2.114 |
| 858 | 180 | 22 | 1bpy−1jqr | 1mq2_A | 0.994 | 1.964 |
| 858 | 180 | 22 | 1bpy−1jqr | 1tv9_A | 0.952 | 1.480 |
| 858 | 180 | 22 | 1bpy−1jqr | 7ict_A | 0.976 | 2.139 |
| 858 | 180 | 22 | 1bpy−1jqr | 1zqb_A | 0.970 | 2.122 |
| 858 | 180 | 22 | 1bpy−1jqr | 7ics_A | 0.952 | 2.140 |
| 858 | 180 | 22 | 1bpy−1jqr | 1zqe_A | 0.916 | 2.122 |
| 858 | 180 | 22 | 1bpy−1jqr | 7icv_A | 0.976 | 2.141 |
| 858 | 180 | 22 | 1bpy−1jqr | 8ici_A | 0.940 | 2.080 |
| 858 | 180 | 22 | 1bpy−1jqr | 7ico_A | 0.952 | 2.184 |
| 858 | 180 | 22 | 1bpy−1jqr | 1zqn_A | 0.946 | 2.133 |
| 858 | 180 | 22 | 1bpy−1jqr | 1mq3_A | 0.964 | 0.834 |
| 858 | 180 | 22 | 1bpy−1jqr | 7ick_A | 0.976 | 2.136 |
| 858 | 180 | 22 | 1bpy−1jqr | 1zqg_A | 0.916 | 2.124 |
| 858 | 180 | 22 | 1bpy−1jqr | 7icm_A | 0.946 | 2.132 |
| 858 | 180 | 22 | 1bpy−1jqr | 1zqh_A | 0.934 | 2.173 |
| 858 | 180 | 22 | 1bpy−1jqr | 1zqf_A | 0.976 | 2.131 |
| 858 | 180 | 22 | 1bpy−1jqr | 1zqp_A | 0.952 | 2.143 |
| 858 | 180 | 22 | 1bpy−1jqr | 1zqm_A | 0.946 | 2.126 |
| 858 | 180 | 22 | 1bpy−1jqr | 7icn_A | 0.952 | 2.116 |
| 858 | 180 | 22 | 1bpy−1jqr | 8ica_A | 0.946 | 2.042 |
| 858 | 180 | 22 | 1bpy−1jqr | 7icq_A | 0.976 | 2.119 |
| 858 | 180 | 22 | 1bpy−1jqr | 1tva_A | 0.964 | 1.642 |
| 858 | 180 | 22 | 1bpy−1jqr | 7icl_A | 0.940 | 2.150 |
| 858 | 180 | 22 | 1bpy−1jqr | 1zqa_A | 0.970 | 2.151 |
| 858 | 180 | 22 | 1bpy−1jqr | 2fmp_A | 0.964 | 0.674 |
| 858 | 180 | 22 | 1bpy−1jqr | 1zqd_A | 0.940 | 2.121 |
| 858 | 180 | 22 | 1bpy−1jqr | 7icp_A | 0.952 | 2.149 |
| 858 | 180 | 22 | 1bpy−1jqr | 1bpz_A | 0.952 | 2.015 |
| 858 | 180 | 22 | 1bpy−1jqr | 1zql_A | 0.946 | 2.138 |
| 858 | 180 | 22 | 1bpy−1jqr | 1zqi_A | 0.952 | 2.139 |
| 858 | 180 | 22 | 1bpy−1jqr | 1zqk_A | 0.976 | 2.129 |
| 858 | 180 | 22 | 1bpy−1jqr | 1zqr_A | 0.970 | 2.119 |
| 858 | 180 | 22 | 1bpy−1jqr | 1zqc_A | 0.970 | 2.133 |
| 858 | 180 | 22 | 1bpy−1jqr | 8icb_A | 0.952 | 2.118 |
| 858 | 180 | 22 | 1bpy−1jqr | 1zqo_A | 0.952 | 2.132 |
| 858 | 180 | 22 | 1bpy−1jqr | 2fms_A | 0.904 | 0.768 |

| n | length | %ID | refA−refB | altA | SP | RMSD |
|---|---|---|---|---|---|---|
| 858 | 180 | 22 | 1bpy−1jqr | 7icu_A | 0.952 | 2.145 |
| 858 | 180 | 22 | 1bpy−1jqr | 7icr_A | 0.946 | 2.133 |
| 858 | 180 | 22 | 1bpy−1jqr | 2fmq_A | 0.946 | 0.745 |
| 858 | 180 | 22 | 1bpy−1jqr | 1zqt_A | 0.916 | 2.108 |
| 858 | 180 | 22 | 1bpy−1jqr | 1zqq_A | 0.916 | 2.097 |
| 858 | 180 | 22 | 1bpy−1jqr | 1zqs_A | 0.916 | 2.135 |
| 858 | 180 | 22 | 1bpy−1jqr | 8icc_A | 0.976 | 2.065 |
| 858 | 180 | 22 | 1bpy−1jqr | 2iso_A | 0.940 | 0.782 |
| 858 | 180 | 22 | 1bpy−1jqr | 2isp_A | 0.904 | 0.772 |
| 858 | 180 | 22 | 1bpy−1jqr | 8ice_A | 0.970 | 2.017 |
| 858 | 180 | 22 | 1bpy−1jqr | 2pxi_A | 0.940 | 0.787 |
| 858 | 180 | 22 | 1bpy−1jqr | 8icf_A | 0.970 | 2.091 |
| 858 | 180 | 22 | 1bpy−1jqr | 7ice_A | 0.976 | 2.133 |
| 858 | 180 | 22 | 1bpy−1jqr | 7icf_A | 0.952 | 2.176 |
| 858 | 180 | 22 | 1bpy−1jqr | 8icg_A | 0.976 | 2.096 |
| 858 | 180 | 22 | 1bpy−1jqr | 7icg_A | 0.952 | 2.125 |
| 858 | 180 | 22 | 1bpy−1jqr | 7ich_A | 0.976 | 2.135 |
| 858 | 180 | 22 | 1bpy−1jqr | 7ici_A | 0.976 | 2.126 |
| 858 | 180 | 22 | 1bpy−1jqr | 8ich_A | 0.946 | 2.092 |
| 858 | 180 | 22 | 1bpy−1jqr | 8icj_A | 0.976 | 2.115 |
| 858 | 180 | 22 | 1bpy−1jqr | 8ick_A | 0.970 | 2.041 |
| 858 | 180 | 22 | 1bpy−1jqr | 8icl_A | 0.976 | 2.086 |
| 858 | 180 | 22 | 1bpy−1jqr | 8icm_A | 0.946 | 2.093 |
| 858 | 180 | 22 | 1bpy−1jqr | 8icn_A | 0.946 | 2.074 |
| 858 | 180 | 22 | 1bpy−1jqr | 8ico_A | 0.970 | 2.041 |
| 858 | 180 | 22 | 1bpy−1jqr | 8icp_A | 0.970 | 2.023 |
| 858 | 180 | 22 | 1bpy−1jqr | 8icq_A | 0.970 | 2.125 |
| 858 | 180 | 22 | 1bpy−1jqr | 8icr_A | 0.970 | 2.059 |
| 858 | 180 | 22 | 1bpy−1jqr | 8ics_A | 0.952 | 2.096 |
| 858 | 180 | 22 | 1bpy−1jqr | 8ict_A | 0.934 | 1.988 |
| 858 | 180 | 22 | 1bpy−1jqr | 8icu_A | 0.916 | 2.013 |
| 858 | 180 | 22 | 1bpy−1jqr | 8icv_A | 0.970 | 2.060 |
| 858 | 180 | 22 | 1bpy−1jqr | 8icw_A | 0.916 | 2.023 |
| 858 | 180 | 22 | 1bpy−1jqr | 8icx_A | 0.976 | 2.046 |
| 858 | 180 | 22 | 1bpy−1jqr | 8icy_A | 0.976 | 2.020 |
| 858 | 180 | 22 | 1bpy−1jqr | 8icz_A | 0.946 | 2.090 |
| 858 | 180 | 22 | 1bpy−1jqr | 9ica_A | 0.952 | 2.108 |
| 858 | 180 | 22 | 1bpy−1jqr | 9icb_A | 0.946 | 2.082 |
| 858 | 180 | 22 | 1bpy−1jqr | 9icc_A | 0.940 | 2.042 |
| 858 | 180 | 22 | 1bpy−1jqr | 1bpx_A | 0.976 | 2.019 |
| 858 | 180 | 22 | 1bpy−1jqr | 9ice_A | 0.976 | 2.055 |
| 858 | 180 | 22 | 1bpy−1jqr | 9icf_A | 0.976 | 2.039 |
| 858 | 180 | 22 | 1bpy−1jqr | 9icg_A | 0.970 | 2.060 |
| 858 | 180 | 22 | 1bpy−1jqr | 9ich_A | 0.946 | 2.099 |
| 858 | 180 | 22 | 1bpy−1jqr | 9ici_A | 0.952 | 2.035 |
| 858 | 180 | 22 | 1bpy−1jqr | 9icj_A | 0.970 | 2.072 |
| 858 | 180 | 22 | 1bpy−1jqr | 9ick_A | 0.976 | 2.136 |
| 858 | 180 | 22 | 1bpy−1jqr | 9icl_A | 0.946 | 2.117 |
| 858 | 180 | 22 | 1bpy−1jqr | 9icm_A | 0.952 | 2.115 |
| 858 | 180 | 22 | 1bpy−1jqr | 9icn_A | 0.976 | 2.053 |
| 858 | 180 | 22 | 1bpy−1jqr | 9ico_A | 0.976 | 2.123 |
| 858 | 180 | 22 | 1bpy−1jqr | 9icp_A | 0.940 | 2.125 |
| 858 | 180 | 22 | 1bpy−1jqr | 9icq_A | 0.946 | 2.106 |
| 858 | 180 | 22 | 1bpy−1jqr | 9icr_A | 0.946 | 2.087 |
| 858 | 180 | 22 | 1bpy−1jqr | 9ics_A | 0.976 | 2.046 |
| 858 | 180 | 22 | 1bpy−1jqr | 9ict_A | 0.970 | 2.113 |
| 858 | 180 | 22 | 1bpy−1jqr | 9icu_A | 0.976 | 2.079 |
| 858 | 180 | 22 | 1bpy−1jqr | 9icv_A | 0.976 | 2.051 |
| 858 | 180 | 22 | 1bpy−1jqr | 9icw_A | 0.946 | 2.100 |
| 858 | 180 | 22 | 1bpy−1jqr | 9icx_A | 0.946 | 2.108 |
| 858 | 180 | 22 | 1bpy−1jqr | 9icy_A | 0.940 | 2.080 |
| 860 | 422 | 22 | 1g6s−1ejd | 2aay_A | 0.980 | 0.179 |
| 860 | 422 | 22 | 1g6s−1ejd | 1g6t_A | 0.975 | 0.103 |
| 860 | 422 | 22 | 1g6s−1ejd | 1x8r_A | 0.975 | 0.179 |
| 860 | 422 | 22 | 1ejd−1g6s | 1ybg_D | 0.995 | 0.629 |
| 860 | 422 | 22 | 1g6s−1ejd | 1x8t_A | 0.975 | 0.468 |
| 860 | 422 | 22 | 1ejd−1g6s | 1ybg_C | 1.000 | 0.631 |
| 860 | 422 | 22 | 1ejd−1g6s | 1ejc_A | 0.927 | 1.263 |
| 860 | 422 | 22 | 1ejd−1g6s | 1eyn_A | 0.955 | 0.954 |
| 860 | 422 | 22 | 1ejd−1g6s | 1ybg_B | 0.975 | 0.680 |
| 860 | 422 | 22 | 1ejd−1g6s | 1ejd_B | 0.952 | 0.693 |

| n | length | %ID | refA−refB | altA | SP | RMSD |
|---|---|---|---|---|---|---|
| 860 | 422 | 22 | 1ejd−1g6s | 1ybg_A | 0.977 | 0.689 |
| 860 | 422 | 22 | 1g6s−1ejd | 2aa9_A | 1.000 | 0.151 |
| 861 | 396 | 22 | 1qay−1dqa | 1qax_A | 0.989 | 0.281 |
| 863 | 54 | 22 | 1ihv−1c1a | 1ihv_B | 1.000 | 0.001 |
| 869 | 252 | 22 | 1dqw−1dbt | 1dqx_C | 0.856 | 1.807 |
| 869 | 252 | 22 | 1dqw−1dbt | 1dqx_A | 0.917 | 1.806 |
| 869 | 252 | 22 | 1dqw−1dbt | 1dqx_B | 0.912 | 1.806 |
| 869 | 252 | 22 | 1dqw−1dbt | 1dqw_C | 1.000 | 0.062 |
| 869 | 252 | 22 | 1dqw−1dbt | 1dqw_D | 0.940 | 0.070 |
| 869 | 252 | 22 | 1dqw−1dbt | 1dqw_B | 0.995 | 0.080 |
| 869 | 252 | 22 | 1dqw−1dbt | 1dqx_D | 0.880 | 1.805 |
| 870 | 498 | 22 | 1dqr−2pgi | 1koj_A | 0.973 | 0.372 |
| 870 | 498 | 22 | 1dqr−2pgi | 1koj_B | 0.930 | 0.526 |
| 870 | 498 | 22 | 2pgi−1dqr | 1c7q_A | 0.964 | 0.218 |
| 870 | 498 | 22 | 1dqr−2pgi | 1dqr_B | 0.969 | 0.548 |
| 876 | 205 | 22 | 1jss−1em2 | 1jss_B | 1.000 | 0.312 |
| 878 | 176 | 22 | 1qsa−154l | 1qte_A | 1.000 | 0.121 |
| 878 | 176 | 22 | 154l−1qsa | 153l_A | 1.000 | 0.153 |
| 880 | 344 | 22 | 1got−1erj | 1b9x_A | 0.967 | 1.372 |
| 880 | 344 | 22 | 1got−1erj | 2trc_B | 0.967 | 1.358 |
| 880 | 344 | 22 | 1got−1erj | 1b9y_A | 0.973 | 1.384 |
| 885 | 202 | 21 | 1bs9−1cex | 1g66_A | 0.994 | 0.234 |
| 887 | 363 | 21 | 1jg8−1b9i | 1jg8_B | 0.968 | 0.650 |
| 887 | 363 | 21 | 1jg8−1b9i | 1jg8_D | 0.946 | 0.760 |
| 888 | 182 | 21 | 1j54−1fxx | 1j53_A | 0.970 | 0.068 |
| 888 | 182 | 21 | 1j54−1fxx | 2ido_C | 0.958 | 1.420 |
| 890 | 140 | 21 | 1hp5−1qba | 1m01_A | 0.953 | 0.217 |
| 890 | 140 | 21 | 1hp5−1qba | 1hp4_A | 0.945 | 0.244 |
| 890 | 140 | 21 | 1qba−1hp5 | 1qbb_A | 0.984 | 0.137 |
| 890 | 140 | 21 | 1hp5−1qba | 1jak_A | 0.953 | 0.279 |
| 891 | 245 | 21 | 1qo2−1thf | 1qo2_B | 0.973 | 0.728 |
| 894 | 70 | 21 | 1f3m−1ej5 | 1f3m_B | 0.731 | 6.294 |
| 901 | 102 | 21 | 2spc−1aj3 | 2spc_B | 0.269 | 1.095 |
| 902 | 133 | 21 | 1lnq−1id1 | 1lnq_C | 1.000 | 0.010 |
| 902 | 133 | 21 | 1lnq−1id1 | 1lnq_G | 1.000 | 0.023 |
| 902 | 133 | 21 | 1lnq−1id1 | 1lnq_B | 1.000 | 0.025 |
| 902 | 133 | 21 | 1lnq−1id1 | 1lnq_D | 1.000 | 0.024 |
| 902 | 133 | 21 | 1lnq−1id1 | 1lnq_H | 1.000 | 0.021 |
| 902 | 133 | 21 | 1lnq−1id1 | 1lnq_E | 1.000 | 0.022 |
| 902 | 133 | 21 | 1lnq−1id1 | 1lnq_F | 1.000 | 0.021 |
| 903 | 138 | 21 | 1gmw−1eb0 | 1gmw_B | 0.977 | 0.704 |
| 903 | 138 | 21 | 1gmw−1eb0 | 1gmw_C | 0.992 | 1.271 |
| 906 | 279 | 20 | 1bt3−1js8 | 1bt1_B | 0.971 | 0.255 |
| 906 | 279 | 20 | 1bt3−1js8 | 1bt2_A | 0.884 | 0.341 |
| 906 | 279 | 20 | 1bt3−1js8 | 1bug_A | 0.983 | 0.301 |
| 906 | 279 | 20 | 1bt3−1js8 | 1bt2_B | 0.896 | 0.344 |
| 906 | 279 | 20 | 1js8−1bt3 | 1js8_B | 1.000 | 0.363 |
| 906 | 279 | 20 | 1bt3−1js8 | 1bt1_A | 0.967 | 0.236 |
| 906 | 279 | 20 | 1bt3−1js8 | 1bug_B | 0.979 | 0.286 |
| 909 | 120 | 20 | 1dhn−1b9l | 2nm2_D | 0.965 | 0.490 |
| 909 | 120 | 20 | 1dhn−1b9l | 1u68_A | 1.000 | 0.126 |
| 909 | 120 | 20 | 1dhn−1b9l | 2dhn_A | 1.000 | 0.202 |
| 909 | 120 | 20 | 1dhn−1b9l | 2nm2_B | 0.982 | 0.449 |
| 909 | 120 | 20 | 1dhn−1b9l | 1rs2_A | 1.000 | 0.236 |
| 909 | 120 | 20 | 1dhn−1b9l | 1rry_A | 1.000 | 0.229 |
| 909 | 120 | 20 | 1b9l−1dhn | 1b9l_E | 0.982 | 0.273 |
| 909 | 120 | 20 | 1dhn−1b9l | 2nm3_A | 1.000 | 0.267 |
| 909 | 120 | 20 | 1dhn−1b9l | 1rri_A | 1.000 | 0.115 |
| 909 | 120 | 20 | 1b9l−1dhn | 1b9l_G | 0.982 | 0.341 |
| 909 | 120 | 20 | 1dhn−1b9l | 2nm2_C | 1.000 | 0.435 |
| 909 | 120 | 20 | 1b9l−1dhn | 1b9l_C | 0.982 | 0.324 |
| 909 | 120 | 20 | 1dhn−1b9l | 1rrw_A | 0.991 | 0.232 |
| 909 | 120 | 20 | 1dhn−1b9l | 1rs4_A | 1.000 | 0.133 |
| 909 | 120 | 20 | 1b9l−1dhn | 1b9l_F | 0.982 | 0.336 |
| 909 | 120 | 20 | 1dhn−1b9l | 1rsi_A | 0.982 | 0.186 |
| 909 | 120 | 20 | 1dhn−1b9l | 2nm2_A | 0.947 | 0.499 |
| 909 | 120 | 20 | 1b9l−1dhn | 1b9l_H | 0.982 | 0.288 |
| 909 | 120 | 20 | 1b9l−1dhn | 1b9l_D | 0.982 | 0.482 |
| 909 | 120 | 20 | 1dhn−1b9l | 1rsd_A | 1.000 | 0.049 |
| 909 | 120 | 20 | 1b9l−1dhn | 1b9l_B | 0.982 | 0.285 |
| 910 | 146 | 20 | 1dar−1n0v | 1ktv_B | 1.000 | 0.915 |

n: internal numbering; length of alignment; % sequence ID (cf. Homstrad); refA−refB: reference alignment; altA: alternative for struc. A; SP: sum−of−pairs score of alt. alignment w/r reference

| n | length | %ID | refA−refB | altA | SP | RMSD |
|---|---|---|---|---|---|---|
| 910 | 146 | 20 | 1n0v−1dar | 1n0v_D | 1.000 | 0.274 |
| 910 | 146 | 20 | 1dar−1n0v | 1elo_A | 1.000 | 0.807 |
| 910 | 146 | 20 | 1n0v−1dar | 2e1r_A | 1.000 | 0.899 |
| 910 | 146 | 20 | 1n0v−1dar | 2p8w_T | 1.000 | 0.572 |
| 910 | 146 | 20 | 1n0v−1dar | 2npf_A | 1.000 | 1.012 |
| 910 | 146 | 20 | 1n0v−1dar | 2npf_B | 1.000 | 1.015 |
| 910 | 146 | 20 | 1n0v−1dar | 1s1h_T | 1.000 | 0.617 |
| 910 | 146 | 20 | 1dar−1n0v | 2om7_L | 0.915 | 3.287 |
| 910 | 146 | 20 | 1n0v−1dar | 1n0u_A | 1.000 | 0.617 |
| 910 | 146 | 20 | 1dar−1n0v | 1ktv_A | 1.000 | 0.909 |
| 911 | 218 | 20 | 1mun−2abk | 1mud_A | 0.981 | 0.329 |
| 911 | 218 | 20 | 1mun−2abk | 1mun_A | 0.956 | 0.164 |
| 914 | 205 | 20 | 1dp0−1bhg | 1jz4_B | 1.000 | 0.190 |
| 914 | 205 | 20 | 1dp0−1bhg | 1jz7_D | 1.000 | 0.364 |
| 914 | 205 | 20 | 1dp0−1bhg | 1hn1_A | 0.958 | 0.322 |
| 914 | 205 | 20 | 1dp0−1bhg | 1hn1_B | 0.964 | 0.313 |
| 914 | 205 | 20 | 1dp0−1bhg | 1jz5_B | 1.000 | 0.181 |
| 914 | 205 | 20 | 1dp0−1bhg | 1jyx_A | 1.000 | 0.154 |
| 914 | 205 | 20 | 1dp0−1bhg | 1jz6_B | 1.000 | 0.295 |
| 914 | 205 | 20 | 1dp0−1bhg | 1dp0_C | 1.000 | 0.150 |
| 914 | 205 | 20 | 1dp0−1bhg | 1jz5_D | 0.945 | 0.379 |
| 914 | 205 | 20 | 1dp0−1bhg | 1jz7_C | 1.000 | 0.159 |
| 914 | 205 | 20 | 1dp0−1bhg | 1hn1_C | 1.000 | 0.304 |
| 914 | 205 | 20 | 1dp0−1bhg | 1jz3_C | 1.000 | 0.155 |
| 914 | 205 | 20 | 1dp0−1bhg | 1jz7_A | 1.000 | 0.086 |
| 914 | 205 | 20 | 1dp0−1bhg | 1jyx_C | 1.000 | 0.158 |
| 914 | 205 | 20 | 1dp0−1bhg | 1jz3_B | 1.000 | 0.171 |
| 914 | 205 | 20 | 1dp0−1bhg | 1jz6_D | 1.000 | 0.408 |
| 914 | 205 | 20 | 1bhg−1dp0 | 1bhg_B | 0.939 | 0.945 |
| 914 | 205 | 20 | 1dp0−1bhg | 1jz5_C | 1.000 | 0.164 |
| 914 | 205 | 20 | 1dp0−1bhg | 1jz3_A | 1.000 | 0.063 |
| 914 | 205 | 20 | 1dp0−1bhg | 1jz4_A | 1.000 | 0.119 |
| 914 | 205 | 20 | 1dp0−1bhg | 1hn1_D | 1.000 | 0.340 |
| 914 | 205 | 20 | 1dp0−1bhg | 1jz4_C | 1.000 | 0.197 |
| 914 | 205 | 20 | 1dp0−1bhg | 1jyx_D | 1.000 | 0.299 |
| 914 | 205 | 20 | 1dp0−1bhg | 1dp0_B | 1.000 | 0.162 |
| 914 | 205 | 20 | 1dp0−1bhg | 1jz6_A | 0.970 | 0.171 |
| 914 | 205 | 20 | 1dp0−1bhg | 1dp0_D | 0.958 | 0.370 |
| 914 | 205 | 20 | 1dp0−1bhg | 1jz7_B | 1.000 | 0.167 |
| 914 | 205 | 20 | 1dp0−1bhg | 1jz4_D | 0.964 | 0.386 |
| 914 | 205 | 20 | 1dp0−1bhg | 1jz3_D | 1.000 | 0.369 |
| 914 | 205 | 20 | 1dp0−1bhg | 1jz5_A | 0.964 | 0.107 |
| 914 | 205 | 20 | 1dp0−1bhg | 1jyx_B | 0.994 | 0.182 |
| 914 | 205 | 20 | 1dp0−1bhg | 1jz6_C | 1.000 | 0.201 |
| 915 | 120 | 20 | 4rhn−4fit | 5rhn_A | 0.887 | 0.334 |
| 915 | 120 | 20 | 4rhn−4fit | 6rhn_A | 0.849 | 0.296 |
| 915 | 120 | 20 | 4rhn−4fit | 3rhn_A | 0.840 | 0.157 |
| 917 | 304 | 20 | 1bk0−1dcs | 1obn_A | 0.973 | 0.103 |
| 917 | 304 | 20 | 1bk0−1dcs | 1oc1_A | 0.965 | 0.270 |
| 917 | 304 | 20 | 1bk0−1dcs | 1w03_A | 0.973 | 0.126 |
| 917 | 304 | 20 | 1bk0−1dcs | 1w06_A | 0.969 | 0.228 |
| 917 | 304 | 20 | 1bk0−1dcs | 2ivj_A | 0.965 | 0.074 |
| 917 | 304 | 20 | 1bk0−1dcs | 1hb2_A | 0.973 | 0.157 |
| 917 | 304 | 20 | 1dcs−1bk0 | 1dcs_A | 0.973 | 0.089 |
| 917 | 304 | 20 | 1bk0−1dcs | 1odm_A | 0.984 | 0.125 |
| 917 | 304 | 20 | 1bk0−1dcs | 1uzw_A | 0.984 | 0.164 |
| 917 | 304 | 20 | 1bk0−1dcs | 1w3x_A | 0.992 | 0.103 |
| 917 | 304 | 20 | 1bk0−1dcs | 1hb3_A | 0.973 | 0.109 |
| 917 | 304 | 20 | 1bk0−1dcs | 2bu9_A | 0.973 | 0.131 |
| 917 | 304 | 20 | 1bk0−1dcs | 2jb4_A | 0.973 | 0.119 |
| 917 | 304 | 20 | 1bk0−1dcs | 2ivi_B | 0.973 | 0.084 |
| 917 | 304 | 20 | 1bk0−1dcs | 1hb4_A | 0.965 | 0.123 |
| 917 | 304 | 20 | 1bk0−1dcs | 1w05_A | 0.973 | 0.299 |
| 917 | 304 | 20 | 1bk0−1dcs | 1w04_A | 0.992 | 0.170 |
| 917 | 304 | 20 | 1bk0−1dcs | 1w3v_A | 0.992 | 0.123 |
| 926 | 66 | 19 | 1jt6−2tct | 1jt6_E | 0.875 | 0.753 |
| 926 | 66 | 19 | 1jt6−2tct | 1rpw_A | 0.875 | 0.964 |
| 926 | 66 | 19 | 1jt6−2tct | 1rpw_D | 0.875 | 0.779 |
| 926 | 66 | 19 | 1jt6−2tct | 1jt6_B | 0.875 | 0.809 |
| 926 | 66 | 19 | 1jt6−2tct | 1rpw_B | 0.938 | 0.240 |
| 926 | 66 | 19 | 1jt6−2tct | 1rpw_C | 0.938 | 0.880 |
| 926 | 66 | 19 | 1jt6−2tct | 1jt6_D | 0.875 | 0.781 |
| 927 | 123 | 19 | 1cgp−1rgs | 1cgp_B | 0.991 | 0.535 |
| 927 | 123 | 19 | 1rgs−1cgp | 1rl3_A | 0.784 | 2.914 |
| 928 | 294 | 19 | 1fo4−1ffv | 1vdv_A | 1.000 | 0.210 |
| 928 | 294 | 19 | 1ffv−1fo4 | 1ffv_F | 1.000 | 0.218 |
| 930 | 135 | 19 | 1qun−1pdk | 1klf_L | 0.891 | 1.343 |
| 930 | 135 | 19 | 1qun−1pdk | 1klf_D | 0.916 | 0.686 |
| 930 | 135 | 19 | 1qun−1pdk | 1qun_D | 0.950 | 0.043 |
| 930 | 135 | 19 | 1qun−1pdk | 1klf_J | 0.891 | 1.343 |
| 930 | 135 | 19 | 1qun−1pdk | 1qun_N | 0.958 | 0.985 |
| 930 | 135 | 19 | 1qun−1pdk | 1klf_H | 0.916 | 0.686 |
| 930 | 135 | 19 | 1qun−1pdk | 1klf_N | 0.891 | 1.344 |
| 930 | 135 | 19 | 1qun−1pdk | 1qun_F | 1.000 | 0.043 |
| 930 | 135 | 19 | 1qun−1pdk | 1qun_J | 0.866 | 0.985 |
| 930 | 135 | 19 | 1qun−1pdk | 1qun_L | 0.857 | 0.999 |
| 930 | 135 | 19 | 1qun−1pdk | 1qun_P | 0.857 | 0.999 |
| 930 | 135 | 19 | 1qun−1pdk | 1klf_F | 0.916 | 0.686 |
| 930 | 135 | 19 | 1qun−1pdk | 1klf_B | 0.916 | 0.686 |
| 930 | 135 | 19 | 1qun−1pdk | 1klf_P | 0.891 | 1.343 |
| 931 | 470 | 19 | 1h83−1f8s | 1b5q_C | 0.947 | 0.210 |
| 931 | 470 | 19 | 1f8s−1h83 | 1f8s_F | 0.928 | 0.025 |
| 931 | 470 | 19 | 1f8s−1h83 | 1f8s_G | 0.904 | 0.026 |
| 931 | 470 | 19 | 1f8s−1h83 | 2iid_B | 0.844 | 0.278 |
| 931 | 470 | 19 | 1h83−1f8s | 1b37_A | 0.876 | 0.062 |
| 931 | 470 | 19 | 1f8s−1h83 | 1f8s_A | 1.000 | 0.004 |
| 931 | 470 | 19 | 1h83−1f8s | 1h82_C | 0.871 | 0.211 |
| 931 | 470 | 19 | 1h83−1f8s | 1h86_B | 0.876 | 0.438 |
| 931 | 470 | 19 | 1f8s−1h83 | 1f8s_C | 0.873 | 0.028 |
| 931 | 470 | 19 | 1h83−1f8s | 1h81_C | 0.885 | 0.418 |
| 931 | 470 | 19 | 1h83−1f8s | 1h84_B | 0.816 | 0.411 |
| 931 | 470 | 19 | 1f8s−1h83 | 1f8s_H | 0.876 | 0.027 |
| 931 | 470 | 19 | 1f8s−1h83 | 2iid_A | 0.888 | 0.265 |
| 931 | 470 | 19 | 1h83−1f8s | 1h84_A | 0.859 | 0.119 |
| 931 | 470 | 19 | 1f8s−1h83 | 1f8r_A | 0.876 | 0.210 |
| 931 | 470 | 19 | 1h83−1f8s | 1b37_B | 0.844 | 0.405 |
| 931 | 470 | 19 | 1h83−1f8s | 1h81_B | 0.830 | 0.528 |
| 931 | 470 | 19 | 1h83−1f8s | 1h82_A | 0.878 | 0.121 |
| 931 | 470 | 19 | 1h83−1f8s | 1h81_A | 0.938 | 0.375 |
| 931 | 470 | 19 | 1f8s−1h83 | 1f8s_D | 0.878 | 0.030 |
| 931 | 470 | 19 | 1f8s−1h83 | 1f8r_B | 0.888 | 0.248 |
| 931 | 470 | 19 | 1h83−1f8s | 1h83_C | 0.830 | 0.204 |
| 931 | 470 | 19 | 1h83−1f8s | 1h84_C | 0.883 | 0.224 |
| 931 | 470 | 19 | 1f8s−1h83 | 2iid_C | 0.904 | 0.215 |
| 931 | 470 | 19 | 1f8s−1h83 | 2iid_D | 0.878 | 0.210 |
| 931 | 470 | 19 | 1f8s−1h83 | 1f8r_C | 0.888 | 0.201 |
| 931 | 470 | 19 | 1f8s−1h83 | 1f8s_B | 0.897 | 0.026 |
| 931 | 470 | 19 | 1f8s−1h83 | 1f8s_E | 0.928 | 0.027 |
| 931 | 470 | 19 | 1h83−1f8s | 1h82_B | 0.880 | 0.422 |
| 931 | 470 | 19 | 1h83−1f8s | 1b37_C | 0.856 | 0.210 |
| 931 | 470 | 19 | 1h83−1f8s | 1b5q_A | 0.876 | 0.081 |
| 931 | 470 | 19 | 1f8s−1h83 | 1f8r_D | 0.828 | 0.201 |
| 931 | 470 | 19 | 1h83−1f8s | 1h86_A | 0.959 | 0.169 |
| 931 | 470 | 19 | 1h83−1f8s | 1h86_C | 0.880 | 0.259 |
| 931 | 470 | 19 | 1h83−1f8s | 1b5q_B | 0.847 | 0.408 |
| 931 | 470 | 19 | 1h83−1f8s | 1h83_B | 0.856 | 0.415 |
| 932 | 250 | 19 | 1hyo−1gtt | 2hzy_B | 1.000 | 0.549 |
| 932 | 250 | 19 | 1gtt−1hyo | 1i7o_C | 1.000 | 0.304 |
| 932 | 250 | 19 | 1gtt−1hyo | 1i7o_A | 1.000 | 0.186 |
| 932 | 250 | 19 | 1hyo−1gtt | 2hzy_A | 0.995 | 0.174 |
| 932 | 250 | 19 | 1gtt−1hyo | 1gtt_B | 1.000 | 0.131 |
| 932 | 250 | 19 | 1gtt−1hyo | 1gtt_D | 1.000 | 0.236 |
| 932 | 250 | 19 | 1gtt−1hyo | 1i7o_B | 1.000 | 0.237 |
| 932 | 250 | 19 | 1gtt−1hyo | 1i7o_D | 1.000 | 0.319 |
| 932 | 250 | 19 | 1gtt−1hyo | 1gtt_C | 1.000 | 0.229 |
| 934 | 266 | 19 | 1ga8−1ll3 | 1g9r_A | 0.921 | 0.158 |
| 935 | 184 | 19 | 1a9n−1d0b | 1a9n_C | 1.000 | 0.160 |
| 936 | 426 | 19 | 1jki−1gr0 | 1p1j_A | 1.000 | 0.648 |
| 936 | 426 | 19 | 1jki−1gr0 | 1rm0_A | 0.997 | 0.575 |
| 936 | 426 | 19 | 1jki−1gr0 | 1p1k_A | 1.000 | 0.506 |
| 937 | 336 | 19 | 1ofg−1gcu | 1ofg_D | 0.972 | 0.001 |
| 937 | 336 | 19 | 1ofg−1gcu | 1ofg_E | 0.986 | 0.001 |
| 937 | 336 | 19 | 1ofg−1gcu | 1ofg_F | 1.000 | 0.001 |
| 937 | 336 | 19 | 1ofg−1gcu | 1ofg_B | 0.986 | 0.001 |
| 937 | 336 | 19 | 1ofg−1gcu | 1ofg_C | 0.986 | 0.001 |
| 938 | 107 | 19 | 1jj2−1bxy | 1k73_X | 1.000 | 0.042 |
| 938 | 107 | 19 | 1jj2−1bxy | 1k8a_X | 1.000 | 0.086 |
| 938 | 107 | 19 | 1jj2−1bxy | 1k9m_X | 1.000 | 0.073 |
| 938 | 107 | 19 | 1jj2−1bxy | 1kc8_X | 1.000 | 0.068 |
| 938 | 107 | 19 | 1jj2−1bxy | 1kd1_X | 1.000 | 0.086 |
| 938 | 107 | 19 | 1jj2−1bxy | 1kqs_V | 1.000 | 0.085 |
| 938 | 107 | 19 | 1jj2−1bxy | 1m1k_X | 1.000 | 0.081 |
| 938 | 107 | 19 | 1jj2−1bxy | 1m90_X | 1.000 | 0.083 |
| 938 | 107 | 19 | 1jj2−1bxy | 1n8r_X | 1.000 | 0.107 |
| 938 | 107 | 19 | 1jj2−1bxy | 1nji_X | 1.000 | 0.065 |
| 938 | 107 | 19 | 1jj2−1bxy | 1q7y_X | 1.000 | 0.141 |
| 938 | 107 | 19 | 1jj2−1bxy | 1q81_X | 1.000 | 0.127 |
| 938 | 107 | 19 | 1jj2−1bxy | 1q82_X | 1.000 | 0.088 |
| 938 | 107 | 19 | 1jj2−1bxy | 1q86_X | 1.000 | 0.114 |
| 938 | 107 | 19 | 1jj2−1bxy | 1qvf_V | 1.000 | 0.054 |
| 938 | 107 | 19 | 1jj2−1bxy | 1qvg_V | 1.000 | 0.130 |
| 938 | 107 | 19 | 1jj2−1bxy | 1s72_W | 1.000 | 0.026 |
| 938 | 107 | 19 | 1jj2−1bxy | 1vq4_W | 1.000 | 0.138 |
| 938 | 107 | 19 | 1jj2−1bxy | 1vq5_W | 1.000 | 0.139 |
| 938 | 107 | 19 | 1jj2−1bxy | 1vq6_W | 1.000 | 0.147 |
| 938 | 107 | 19 | 1jj2−1bxy | 1vq7_W | 1.000 | 0.150 |
| 938 | 107 | 19 | 1jj2−1bxy | 1vq8_W | 1.000 | 0.246 |
| 938 | 107 | 19 | 1jj2−1bxy | 1vq9_W | 1.000 | 0.256 |
| 938 | 107 | 19 | 1jj2−1bxy | 1vqk_W | 1.000 | 0.222 |
| 938 | 107 | 19 | 1jj2−1bxy | 1vql_W | 1.000 | 0.208 |
| 938 | 107 | 19 | 1jj2−1bxy | 1vqm_W | 1.000 | 0.226 |
| 938 | 107 | 19 | 1jj2−1bxy | 1vqn_W | 1.000 | 0.234 |
| 938 | 107 | 19 | 1jj2−1bxy | 1vqo_W | 1.000 | 0.216 |
| 938 | 107 | 19 | 1jj2−1bxy | 1vqp_W | 1.000 | 0.188 |
| 938 | 107 | 19 | 1jj2−1bxy | 1w2b_V | 1.000 | 0.198 |
| 938 | 107 | 19 | 1jj2−1bxy | 1yhq_W | 1.000 | 0.118 |
| 938 | 107 | 19 | 1jj2−1bxy | 1yi2_W | 1.000 | 0.149 |
| 938 | 107 | 19 | 1jj2−1bxy | 1yij_W | 1.000 | 0.155 |
| 938 | 107 | 19 | 1jj2−1bxy | 1yit_W | 1.000 | 0.187 |
| 938 | 107 | 19 | 1jj2−1bxy | 1yj9_W | 1.000 | 0.203 |
| 938 | 107 | 19 | 1jj2−1bxy | 1yjn_W | 1.000 | 0.216 |
| 938 | 107 | 19 | 1jj2−1bxy | 1yjw_W | 1.000 | 0.203 |
| 938 | 107 | 19 | 1jj2−1bxy | 2otj_W | 1.000 | 0.150 |
| 938 | 107 | 19 | 1jj2−1bxy | 2otl_W | 1.000 | 0.155 |
| 938 | 107 | 19 | 1bxy−1jj2 | 1bxy_B | 1.000 | 0.967 |
| 938 | 107 | 19 | 1bxy−1jj2 | 1vsa_X | 1.000 | 1.620 |
| 938 | 107 | 19 | 1bxy−1jj2 | 1vsp_X | 1.000 | 1.293 |
| 938 | 107 | 19 | 1bxy−1jj2 | 1yl3_X | 0.983 | 1.152 |
| 938 | 107 | 19 | 1bxy−1jj2 | 2b66_3 | 0.983 | 1.122 |
| 938 | 107 | 19 | 1bxy−1jj2 | 2b9n_3 | 0.983 | 1.122 |
| 938 | 107 | 19 | 1bxy−1jj2 | 2b9p_3 | 0.983 | 1.122 |
| 939 | 114 | 19 | 1f7u−1iq0 | 1f7v_A | 1.000 | 0.631 |
| 941 | 244 | 19 | 1kez−1jmk | 1kez_C | 0.995 | 0.683 |
| 941 | 244 | 19 | 1kez−1jmk | 1kez_B | 0.841 | 1.613 |
| 945 | 113 | 18 | 1qts−1e42 | 1ky6_A | 1.000 | 0.405 |
| 945 | 113 | 18 | 1qts−1e42 | 1kyd_A | 1.000 | 0.196 |
| 945 | 113 | 18 | 1qts−1e42 | 1kyf_A | 1.000 | 0.169 |
| 945 | 113 | 18 | 1qts−1e42 | 1ky7_A | 0.991 | 0.202 |
| 945 | 113 | 18 | 1qts−1e42 | 1kyu_A | 0.991 | 0.533 |
| 945 | 113 | 18 | 1e42−1qts | 1e42_B | 1.000 | 0.131 |
| 945 | 113 | 18 | 1e42−1qts | 2g30_A | 1.000 | 0.430 |
| 946 | 240 | 18 | 1b74−1jfl | 1b73_A | 0.995 | 0.348 |
| 946 | 240 | 18 | 1jfl−1b74 | 1jfl_B | 0.976 | 0.463 |
| 951 | 174 | 18 | 1h72−1fi4 | 1fwk_D | 1.000 | 0.376 |
| 951 | 174 | 18 | 1h72−1fi4 | 1fwl_C | 0.986 | 0.326 |
| 951 | 174 | 18 | 1h72−1fi4 | 1h74_B | 0.993 | 0.507 |
| 951 | 174 | 18 | 1h72−1fi4 | 1h74_D | 0.939 | 0.290 |
| 951 | 174 | 18 | 1h72−1fi4 | 1fwk_A | 1.000 | 0.483 |
| 951 | 174 | 18 | 1h72−1fi4 | 1fwl_A | 0.986 | 0.706 |
| 951 | 174 | 18 | 1h72−1fi4 | 1fwl_B | 0.946 | 0.891 |
| 951 | 174 | 18 | 1h72−1fi4 | 1fwl_D | 1.000 | 0.481 |
| 951 | 174 | 18 | 1h72−1fi4 | 1h74_A | 1.000 | 0.433 |
| 951 | 174 | 18 | 1h72−1fi4 | 1h74_C | 0.986 | 0.338 |

n: internal numbering; length of alignment; % sequence ID (cf. Homstrad); refA−refB: reference alignment; altA: alternative for struc. A; SP: sum−of−pairs score of alt. alignment w/r reference

| n | length | %ID | refA−refB | altA | SP | RMSD |
|---|---|---|---|---|---|---|
| 951 | 174 | 18 | 1h72−1fi4 | 1fwk_B | 0.946 | 0.766 |
| 951 | 174 | 18 | 1h72−1fi4 | 1h72_C | 1.000 | 0.009 |
| 951 | 174 | 18 | 1h72−1fi4 | 1h73_A | 1.000 | 0.482 |
| 951 | 174 | 18 | 1h72−1fi4 | 1fwk_C | 1.000 | 0.343 |
| 957 | 254 | 18 | 1n2z−1efd | 1n2z_B | 0.889 | 0.760 |
| 958 | 339 | 18 | 1egu−1cb8 | 2brw_B | 0.993 | 1.783 |
| 958 | 339 | 18 | 1egu−1cb8 | 2brp_A | 0.997 | 0.357 |
| 958 | 339 | 18 | 1egu−1cb8 | 1ojp_A | 0.997 | 0.260 |
| 958 | 339 | 18 | 1egu−1cb8 | 1c82_A | 0.983 | 0.172 |
| 958 | 339 | 18 | 1egu−1cb8 | 1w3y_A | 0.997 | 0.373 |
| 958 | 339 | 18 | 1egu−1cb8 | 1f9g_A | 0.997 | 0.307 |
| 958 | 339 | 18 | 1egu−1cb8 | 2brw_A | 0.993 | 1.821 |
| 959 | 404 | 18 | 1khv−1rdr | 1khw_A | 0.977 | 0.315 |
| 961 | 733 | 18 | 1m2o−1m2v | 1m2o_C | 0.970 | 0.292 |
| 962 | 367 | 18 | 1ju3−1lns | 1ju4_A | 0.973 | 0.429 |
| 966 | 190 | 17 | 1pbw−1tx4 | 1pbw_B | 0.961 | 1.234 |
| 971 | 419 | 17 | 1pbe−1foh | 1phh_A | 0.970 | 0.815 |
| 971 | 419 | 17 | 1pbe−1foh | 2phh_A | 0.995 | 0.336 |
| 971 | 419 | 17 | 1foh−1pbe | 1foh_B | 1.000 | 0.205 |
| 971 | 419 | 17 | 1pbe−1foh | 1pdh_A | 0.989 | 0.219 |
| 972 | 292 | 17 | 1esm−1a7j | 1esn_A | 0.938 | 2.910 |
| 973 | 269 | 17 | 1n1m−1h2w | 1n1m_B | 1.000 | 0.334 |
| 973 | 269 | 17 | 1n1m−1h2w | 1tkr_A | 0.984 | 0.214 |
| 973 | 269 | 17 | 1n1m−1h2w | 1tkr_B | 0.984 | 0.367 |
| 973 | 269 | 17 | 1n1m−1h2w | 1w1i_C | 1.000 | 0.356 |
| 973 | 269 | 17 | 1n1m−1h2w | 2bgn_B | 0.957 | 0.357 |
| 973 | 269 | 17 | 1n1m−1h2w | 1nu8_B | 1.000 | 0.301 |
| 973 | 269 | 17 | 1n1m−1h2w | 2ogz_A | 1.000 | 0.269 |
| 973 | 269 | 17 | 1h2w−1n1m | 1qfs_A | 1.000 | 0.126 |
| 973 | 269 | 17 | 1n1m−1h2w | 1rwq_B | 1.000 | 0.293 |
| 973 | 269 | 17 | 1n1m−1h2w | 2bub_B | 0.984 | 0.361 |
| 973 | 269 | 17 | 1n1m−1h2w | 1w1i_A | 1.000 | 0.354 |
| 973 | 269 | 17 | 1n1m−1h2w | 1w1i_B | 1.000 | 0.355 |
| 973 | 269 | 17 | 1n1m−1h2w | 2oqi_B | 1.000 | 0.224 |
| 973 | 269 | 17 | 1n1m−1h2w | 2ajl_J | 1.000 | 0.335 |
| 973 | 269 | 17 | 1n1m−1h2w | 2oqi_C | 1.000 | 0.274 |
| 973 | 269 | 17 | 1n1m−1h2w | 2ogz_B | 1.000 | 0.284 |
| 973 | 269 | 17 | 1n1m−1h2w | 1tk3_A | 1.000 | 0.199 |
| 973 | 269 | 17 | 1n1m−1h2w | 1rwq_A | 1.000 | 0.285 |
| 973 | 269 | 17 | 1n1m−1h2w | 2bgn_C | 1.000 | 0.357 |
| 973 | 269 | 17 | 1n1m−1h2w | 2oqi_D | 0.984 | 0.274 |
| 973 | 269 | 17 | 1n1m−1h2w | 1w1i_D | 1.000 | 0.358 |
| 973 | 269 | 17 | 1n1m−1h2w | 2ajl_I | 1.000 | 0.292 |
| 973 | 269 | 17 | 1n1m−1h2w | 2bgn_A | 1.000 | 0.356 |
| 973 | 269 | 17 | 1n1m−1h2w | 2bub_A | 0.992 | 0.365 |
| 973 | 269 | 17 | 1n1m−1h2w | 2oqi_A | 1.000 | 0.222 |
| 973 | 269 | 17 | 1n1m−1h2w | 1tk3_B | 1.000 | 0.370 |
| 973 | 269 | 17 | 1n1m−1h2w | 1nu8_A | 1.000 | 0.290 |
| 973 | 269 | 17 | 1n1m−1h2w | 1nu6_B | 1.000 | 0.276 |
| 973 | 269 | 17 | 1n1m−1h2w | 2bgn_D | 0.957 | 0.358 |
| 973 | 269 | 17 | 1n1m−1h2w | 1nu6_A | 1.000 | 0.283 |
| 974 | 89 | 17 | 1gz0−1ipa | 1gz0_D | 1.000 | 0.808 |
| 974 | 89 | 17 | 1gz0−1ipa | 1gz0_H | 1.000 | 0.607 |
| 974 | 89 | 17 | 1gz0−1ipa | 1gz0_B | 0.944 | 1.045 |
| 974 | 89 | 17 | 1gz0−1ipa | 1gz0_F | 1.000 | 0.882 |
| 974 | 89 | 17 | 1gz0−1ipa | 1gz0_C | 1.000 | 0.435 |
| 976 | 400 | 17 | 1kas−1afw | 1b3n_A | 0.932 | 0.233 |
| 978 | 360 | 17 | 2vsg−1vsg | 2vsg_B | 0.934 | 0.732 |
| 978 | 360 | 17 | 1vsg−2vsg | 1vsg_B | 0.964 | 0.384 |
| 979 | 240 | 16 | 1qts−1e42 | 1ky7_A | 1.000 | 0.214 |
| 979 | 240 | 16 | 1qts−1e42 | 1kyu_A | 0.981 | 0.611 |
| 979 | 240 | 16 | 1qts−1e42 | 1kyd_A | 0.995 | 0.223 |
| 979 | 240 | 16 | 1qts−1e42 | 1kyf_A | 0.995 | 0.199 |
| 979 | 240 | 16 | 1e42−1qts | 1e42_B | 0.995 | 0.212 |
| 979 | 240 | 16 | 1qts−1e42 | 1ky6_A | 0.991 | 0.514 |
| 979 | 240 | 16 | 1e42−1qts | 2g30_A | 0.991 | 0.484 |
| 981 | 169 | 16 | 1lam−1gyt | 1lcp_B | 1.000 | 0.153 |
| 981 | 169 | 16 | 1gyt−1lam | 1gyt_C | 0.972 | 0.294 |
| 981 | 169 | 16 | 1gyt−1lam | 1gyt_L | 0.958 | 0.358 |
| 981 | 169 | 16 | 1gyt−1lam | 1gyt_I | 1.000 | 0.353 |
| 981 | 169 | 16 | 1gyt−1lam | 1gyt_K | 1.000 | 0.342 |

| n | length | %ID | refA−refB | altA | SP | RMSD |
|---|---|---|---|---|---|---|
| 981 | 169 | 16 | 1gyt−1lam | 1gyt_E | 0.965 | 0.281 |
| 981 | 169 | 16 | 1gyt−1lam | 1gyt_F | 1.000 | 0.392 |
| 981 | 169 | 16 | 1lam−1gyt | 1lan_A | 1.000 | 0.186 |
| 981 | 169 | 16 | 1gyt−1lam | 1gyt_H | 0.972 | 0.336 |
| 981 | 169 | 16 | 1gyt−1lam | 1gyt_B | 0.965 | 0.275 |
| 981 | 169 | 16 | 1gyt−1lam | 1gyt_D | 0.972 | 0.256 |
| 981 | 169 | 16 | 1gyt−1lam | 1gyt_G | 1.000 | 0.296 |
| 981 | 169 | 16 | 1lam−1gyt | 1lcp_A | 1.000 | 0.184 |
| 981 | 169 | 16 | 1gyt−1lam | 1gyt_J | 1.000 | 0.410 |
| 984 | 367 | 16 | 1f0k−1iir | 1f0k_B | 0.960 | 1.176 |
| 986 | 143 | 16 | 1cs0−1b93 | 1c3o_G | 1.000 | 0.234 |
| 986 | 143 | 16 | 1cs0−1b93 | 1kee_C | 0.993 | 0.224 |
| 986 | 143 | 16 | 1b93−1cs0 | 1egh_C | 0.993 | 0.643 |
| 986 | 143 | 16 | 1b93−1cs0 | 1egh_D | 0.993 | 0.635 |
| 986 | 143 | 16 | 1b93−1cs0 | 1ik4_A | 0.993 | 0.703 |
| 986 | 143 | 16 | 1cs0−1b93 | 1cs0_E | 1.000 | 0.221 |
| 986 | 143 | 16 | 1b93−1cs0 | 1ik4_F | 0.993 | 0.703 |
| 986 | 143 | 16 | 1cs0−1b93 | 1bxr_E | 1.000 | 0.323 |
| 986 | 143 | 16 | 1cs0−1b93 | 1kee_E | 1.000 | 0.218 |
| 986 | 143 | 16 | 1b93−1cs0 | 1b93_B | 1.000 | 0.624 |
| 986 | 143 | 16 | 1cs0−1b93 | 1t36_G | 1.000 | 0.275 |
| 986 | 143 | 16 | 1cs0−1b93 | 1c30_A | 1.000 | 0.164 |
| 986 | 143 | 16 | 1cs0−1b93 | 1bxr_A | 1.000 | 0.253 |
| 986 | 143 | 16 | 1b93−1cs0 | 1egh_E | 0.993 | 0.658 |
| 986 | 143 | 16 | 1b93−1cs0 | 1egh_F | 0.993 | 0.643 |
| 986 | 143 | 16 | 1cs0−1b93 | 1c30_E | 1.000 | 0.249 |
| 986 | 143 | 16 | 1b93−1cs0 | 1ik4_D | 0.993 | 0.696 |
| 986 | 143 | 16 | 1cs0−1b93 | 1kee_G | 1.000 | 0.259 |
| 986 | 143 | 16 | 1cs0−1b93 | 1t36_E | 1.000 | 0.236 |
| 986 | 143 | 16 | 1cs0−1b93 | 1bxr_G | 1.000 | 0.367 |
| 986 | 143 | 16 | 1cs0−1b93 | 1cs0_C | 1.000 | 0.153 |
| 986 | 143 | 16 | 1b93−1cs0 | 1egh_A | 0.993 | 0.643 |
| 986 | 143 | 16 | 1cs0−1b93 | 1kee_A | 1.000 | 0.143 |
| 986 | 143 | 16 | 1cs0−1b93 | 1c3o_C | 1.000 | 0.171 |
| 986 | 143 | 16 | 1b93−1cs0 | 1ik4_B | 0.993 | 0.702 |
| 986 | 143 | 16 | 1b93−1cs0 | 1ik4_C | 0.993 | 0.697 |
| 986 | 143 | 16 | 1b93−1cs0 | 1ik4_E | 0.993 | 0.719 |
| 986 | 143 | 16 | 1cs0−1b93 | 1cs0_G | 1.000 | 0.257 |
| 986 | 143 | 16 | 1cs0−1b93 | 1c30_G | 1.000 | 0.300 |
| 986 | 143 | 16 | 1b93−1cs0 | 1egh_B | 0.993 | 0.642 |
| 986 | 143 | 16 | 1cs0−1b93 | 1c30_C | 0.993 | 0.208 |
| 986 | 143 | 16 | 1cs0−1b93 | 1c3o_A | 1.000 | 0.111 |
| 986 | 143 | 16 | 1cs0−1b93 | 1c3o_E | 1.000 | 0.214 |
| 986 | 143 | 16 | 1cs0−1b93 | 1t36_C | 1.000 | 0.209 |
| 986 | 143 | 16 | 1cs0−1b93 | 1bxr_C | 1.000 | 0.271 |
| 986 | 143 | 16 | 1cs0−1b93 | 1t36_A | 1.000 | 0.169 |
| 987 | 140 | 16 | 1ofg−1gcu | 1ofg_D | 1.000 | 0.001 |
| 987 | 140 | 16 | 1ofg−1gcu | 1ofg_E | 1.000 | 0.001 |
| 987 | 140 | 16 | 1ofg−1gcu | 1ofg_F | 1.000 | 0.001 |
| 987 | 140 | 16 | 1ofg−1gcu | 1ofg_B | 1.000 | 0.001 |
| 987 | 140 | 16 | 1ofg−1gcu | 1ofg_C | 1.000 | 0.001 |
| 988 | 122 | 16 | 1dqe−1c3z | 1dqe_B | 0.843 | 1.006 |
| 992 | 157 | 16 | 1ez3−1fio | 1ez3_C | 0.991 | 1.056 |
| 993 | 585 | 16 | 1ju3−1lns | 1ju4_A | 0.985 | 0.349 |
| 995 | 228 | 15 | 1h7e−1ezi | 1h7h_A | 0.937 | 0.224 |
| 995 | 228 | 15 | 1h7e−1ezi | 1h7g_A | 0.974 | 0.496 |
| 995 | 228 | 15 | 1h7e−1ezi | 1h7t_A | 0.974 | 0.130 |
| 995 | 228 | 15 | 1h7e−1ezi | 1h7f_A | 0.974 | 0.177 |
| 997 | 112 | 15 | 1h83−1f8s | 1h81_C | 0.880 | 0.305 |
| 997 | 112 | 15 | 1h83−1f8s | 1h84_C | 1.000 | 0.209 |
| 997 | 112 | 15 | 1f8s−1h83 | 1f8s_F | 0.963 | 0.020 |
| 997 | 112 | 15 | 1h83−1f8s | 1h83_C | 0.954 | 0.189 |
| 997 | 112 | 15 | 1f8s−1h83 | 1f8s_H | 1.000 | 0.019 |
| 997 | 112 | 15 | 1h83−1f8s | 1b37_B | 0.963 | 0.187 |
| 997 | 112 | 15 | 1f8s−1h83 | 2iid_D | 0.963 | 0.203 |
| 997 | 112 | 15 | 1f8s−1h83 | 1f8r_C | 1.000 | 0.105 |
| 997 | 112 | 15 | 1h83−1f8s | 1b5q_C | 1.000 | 0.197 |
| 997 | 112 | 15 | 1h83−1f8s | 1h81_A | 0.880 | 0.240 |
| 997 | 112 | 15 | 1f8s−1h83 | 1f8s_C | 1.000 | 0.021 |
| 997 | 112 | 15 | 1f8s−1h83 | 2iid_C | 0.963 | 0.194 |
| 997 | 112 | 15 | 1f8s−1h83 | 1f8r_D | 0.880 | 0.105 |

| n | length | %ID | refA−refB | altA | SP | RMSD |
|---|---|---|---|---|---|---|
| 997 | 112 | 15 | 1h83−1f8s | 1h84_A | 0.963 | 0.087 |
| 997 | 112 | 15 | 1f8s−1h83 | 2iid_B | 0.963 | 0.205 |
| 997 | 112 | 15 | 1h83−1f8s | 1b5q_B | 0.926 | 0.192 |
| 997 | 112 | 15 | 1h83−1f8s | 1h82_B | 0.963 | 0.202 |
| 997 | 112 | 15 | 1h83−1f8s | 1h82_A | 0.954 | 0.137 |
| 997 | 112 | 15 | 1h83−1f8s | 1h86_A | 1.000 | 0.115 |
| 997 | 112 | 15 | 1f8s−1h83 | 1f8s_D | 1.000 | 0.018 |
| 997 | 112 | 15 | 1h83−1f8s | 1b37_C | 0.963 | 0.187 |
| 997 | 112 | 15 | 1h83−1f8s | 1b5q_A | 0.963 | 0.074 |
| 997 | 112 | 15 | 1h83−1f8s | 1h84_B | 0.926 | 0.187 |
| 997 | 112 | 15 | 1h83−1f8s | 1h82_C | 0.963 | 0.198 |
| 997 | 112 | 15 | 1f8s−1h83 | 1f8r_B | 1.000 | 0.109 |
| 997 | 112 | 15 | 1f8s−1h83 | 1f8s_B | 0.833 | 0.018 |
| 997 | 112 | 15 | 1h83−1f8s | 1h83_B | 0.963 | 0.190 |
| 997 | 112 | 15 | 1f8s−1h83 | 1f8s_E | 0.880 | 0.020 |
| 997 | 112 | 15 | 1h83−1f8s | 1h81_B | 0.963 | 0.294 |
| 997 | 112 | 15 | 1h83−1f8s | 1h86_C | 0.963 | 0.221 |
| 997 | 112 | 15 | 1h83−1f8s | 1h86_B | 0.880 | 0.282 |
| 997 | 112 | 15 | 1f8s−1h83 | 1f8s_G | 1.000 | 0.020 |
| 997 | 112 | 15 | 1f8s−1h83 | 2iid_A | 0.963 | 0.154 |
| 997 | 112 | 15 | 1h83−1f8s | 1b37_A | 1.000 | 0.054 |
| 997 | 112 | 15 | 1f8s−1h83 | 1f8r_A | 1.000 | 0.108 |
| 998 | 176 | 15 | 1i0r−1eje | 1i0s_B | 0.968 | 1.754 |
| 998 | 176 | 15 | 1i0r−1eje | 1i0r_B | 0.924 | 1.691 |
| 998 | 176 | 15 | 1i0r−1eje | 1i0s_A | 0.994 | 0.227 |
| 999 | 339 | 15 | 1h72−1fi4 | 1fwl_C | 0.880 | 0.511 |
| 999 | 339 | 15 | 1h72−1fi4 | 1h73_A | 0.996 | 0.417 |
| 999 | 339 | 15 | 1h72−1fi4 | 1fwk_A | 0.924 | 0.681 |
| 999 | 339 | 15 | 1h72−1fi4 | 1fwl_B | 0.920 | 1.043 |
| 999 | 339 | 15 | 1h72−1fi4 | 1fwl_D | 0.902 | 0.555 |
| 999 | 339 | 15 | 1h72−1fi4 | 1h74_B | 0.935 | 0.777 |
| 999 | 339 | 15 | 1h72−1fi4 | 1fwk_B | 0.902 | 0.896 |
| 999 | 339 | 15 | 1h72−1fi4 | 1h74_D | 0.928 | 0.430 |
| 999 | 339 | 15 | 1h72−1fi4 | 1fwk_D | 0.928 | 0.491 |
| 999 | 339 | 15 | 1h72−1fi4 | 1fwl_A | 0.899 | 0.815 |
| 999 | 339 | 15 | 1h72−1fi4 | 1h74_C | 0.899 | 0.437 |
| 999 | 339 | 15 | 1h72−1fi4 | 1h74_A | 0.942 | 0.534 |
| 999 | 339 | 15 | 1h72−1fi4 | 1fwk_C | 0.870 | 0.600 |
| 999 | 339 | 15 | 1h72−1fi4 | 1h72_C | 1.000 | 0.009 |
| 1002 | 220 | 15 | 1nba−1yac | 1nba_C | 1.000 | 0.227 |
| 1002 | 220 | 15 | 1nba−1yac | 1nba_D | 1.000 | 0.862 |
| 1002 | 220 | 15 | 1yac−1nba | 1yac_B | 1.000 | 0.085 |
| 1006 | 90 | 14 | 1psd−1phz | 1psd_B | 0.940 | 0.309 |
| 1006 | 90 | 14 | 1phz−1psd | 2phm_A | 1.000 | 0.325 |
| 1007 | 126 | 14 | 1e42−1qts | 2g30_A | 0.955 | 0.398 |
| 1007 | 126 | 14 | 1qts−1e42 | 1kyd_A | 1.000 | 0.213 |
| 1007 | 126 | 14 | 1qts−1e42 | 1kyu_A | 0.982 | 0.501 |
| 1007 | 126 | 14 | 1qts−1e42 | 1ky6_A | 0.991 | 0.539 |
| 1007 | 126 | 14 | 1qts−1e42 | 1ky7_A | 1.000 | 0.204 |
| 1007 | 126 | 14 | 1qts−1e42 | 1kyf_A | 1.000 | 0.210 |
| 1007 | 126 | 14 | 1e42−1qts | 1e42_B | 1.000 | 0.160 |
| 1010 | 120 | 14 | 1b63−1h7s | 1nhi_A | 1.000 | 0.204 |
| 1011 | 208 | 14 | 1h6k−1hu3 | 1h6k_B | 0.967 | 0.147 |
| 1011 | 208 | 14 | 1h6k−1hu3 | 1h6k_C | 0.967 | 0.207 |
| 1013 | 229 | 13 | 1bd0−1ct5 | 1sft_A | 1.000 | 0.262 |
| 1013 | 229 | 13 | 1bd0−1ct5 | 1sft_B | 0.995 | 0.359 |
| 1013 | 229 | 13 | 1bd0−1ct5 | 1bd0_B | 0.995 | 0.301 |
| 1019 | 129 | 13 | 1qqr−2sak | 1qqr_C | 1.000 | 1.246 |
| 1019 | 129 | 13 | 1qqr−2sak | 1qqr_D | 0.982 | 1.919 |
| 1020 | 218 | 13 | 1ju3−1lns | 1ju4_A | 0.953 | 0.138 |
| 1023 | 321 | 12 | 1d2r−2ts1 | 1d2r_D | 0.946 | 0.491 |
| 1023 | 321 | 12 | 1d2r−2ts1 | 1d2r_E | 0.957 | 0.599 |
| 1023 | 321 | 12 | 1d2r−2ts1 | 1d2r_F | 0.988 | 0.550 |
| 1023 | 321 | 12 | 2ts1−1d2r | 3ts1_A | 1.000 | 0.246 |
| 1023 | 321 | 12 | 1d2r−2ts1 | 1d2r_B | 0.888 | 0.326 |
| 1023 | 321 | 12 | 1d2r−2ts1 | 1d2r_C | 0.965 | 0.569 |
| 1025 | 646 | 11 | 3pfl−1b8b | 1h18_B | 0.970 | 0.290 |
| 1025 | 646 | 11 | 3pfl−1b8b | 2pfl_B | 0.978 | 0.315 |
| 1025 | 646 | 11 | 3pfl−1b8b | 3pfl_B | 0.980 | 0.261 |
| 1025 | 646 | 11 | 3pfl−1b8b | 1h16_A | 0.972 | 0.337 |
| 1025 | 646 | 11 | 3pfl−1b8b | 1h17_A | 0.985 | 0.337 |

| n | length | %ID | refA−refB | altA | SP | RMSD |
|---|---|---|---|---|---|---|
| 1025 | 646 | 11 | 3pfl−1b8b | 1h18_A | 0.972 | 0.280 |
| 1025 | 646 | 11 | 3pfl−1b8b | 2pfl_A | 0.967 | 0.175 |
| 1028 | 165 | 10 | 1h72−1fi4 | 1fwk_D | 0.820 | 0.448 |
| 1028 | 165 | 10 | 1h72−1fi4 | 1fwl_A | 0.781 | 0.637 |
| 1028 | 165 | 10 | 1h72−1fi4 | 1fwl_D | 0.984 | 0.566 |
| 1028 | 165 | 10 | 1h72−1fi4 | 1h74_C | 0.781 | 0.381 |
| 1028 | 165 | 10 | 1h72−1fi4 | 1fwk_A | 0.938 | 0.625 |
| 1028 | 165 | 10 | 1h72−1fi4 | 1fwl_B | 0.820 | 0.633 |
| 1028 | 165 | 10 | 1h72−1fi4 | 1fwl_C | 0.945 | 0.560 |
| 1028 | 165 | 10 | 1h72−1fi4 | 1h74_A | 0.820 | 0.407 |
| 1028 | 165 | 10 | 1h72−1fi4 | 1fwk_B | 0.750 | 0.646 |
| 1028 | 165 | 10 | 1h72−1fi4 | 1h74_D | 0.781 | 0.421 |
| 1028 | 165 | 10 | 1h72−1fi4 | 1h72_C | 1.000 | 0.008 |
| 1028 | 165 | 10 | 1h72−1fi4 | 1h73_A | 1.000 | 0.157 |
| 1028 | 165 | 10 | 1h72−1fi4 | 1h74_B | 0.828 | 0.625 |
| 1028 | 165 | 10 | 1h72−1fi4 | 1fwk_C | 0.945 | 0.600 |

n: internal numbering; length of alignment; % sequence ID (cf. Homstrad); refA−refB: reference alignment; altA: alternative for struc. A; SP: sum−of−pairs score of alt. alignment w/r reference 

Supplementary Material Table S2b: Details of aligned Simulation Snapshots −− The Meaning of Alignment (Pirovano, Feenstra & Heringa)

| n | length | %ID | refA−refB | altA | SP | RMSD |
|---|---|---|---|---|---|---|
| 9 | 98 | 87 | 1lri−1ljp | 1lri_1 | 1.000 | 1.710 |
| 9 | 98 | 87 | 1lri−1ljp | 1lri_2 | 1.000 | 1.931 |
| 9 | 98 | 87 | 1lri−1ljp | 1lri_3 | 0.878 | 2.191 |
| 9 | 98 | 87 | 1lri−1ljp | 1lri_4 | 0.888 | 1.981 |
| 9 | 98 | 87 | 1lri−1ljp | 1lri_5 | 0.929 | 1.786 |
| 9 | 98 | 87 | 1lri−1ljp | 1lri_6 | 1.000 | 1.896 |
| 9 | 98 | 87 | 1lri−1ljp | 1lri_7 | 0.929 | 2.102 |
| 9 | 98 | 87 | 1lri−1ljp | 1lri_8 | 0.929 | 2.160 |
| 9 | 98 | 87 | 1lri−1ljp | 1lri_9 | 1.000 | 2.220 |
| 9 | 98 | 87 | 1lri−1ljp | 1lri_10 | 1.000 | 2.228 |
| 9 | 98 | 87 | 1ljp−1lri | 1ljp_1 | 1.000 | 1.761 |
| 9 | 98 | 87 | 1ljp−1lri | 1ljp_2 | 1.000 | 2.243 |
| 9 | 98 | 87 | 1ljp−1lri | 1ljp_3 | 1.000 | 2.136 |
| 9 | 98 | 87 | 1ljp−1lri | 1ljp_4 | 1.000 | 1.986 |
| 9 | 98 | 87 | 1ljp−1lri | 1ljp_5 | 1.000 | 2.223 |
| 9 | 98 | 87 | 1ljp−1lri | 1ljp_6 | 1.000 | 2.434 |
| 10 | 146 | 87 | 1de3−1aqz | 1de3_1 | 0.944 | 2.381 |
| 10 | 146 | 87 | 1de3−1aqz | 1de3_2 | 0.951 | 3.049 |
| 10 | 146 | 87 | 1de3−1aqz | 1de3_3 | 0.901 | 3.445 |
| 10 | 146 | 87 | 1de3−1aqz | 1de3_4 | 0.944 | 3.741 |
| 10 | 146 | 87 | 1de3−1aqz | 1de3_5 | 0.965 | 4.161 |
| 10 | 146 | 87 | 1de3−1aqz | 1de3_6 | 0.965 | 3.544 |
| 10 | 146 | 87 | 1de3−1aqz | 1de3_7 | 0.958 | 3.789 |
| 10 | 146 | 87 | 1de3−1aqz | 1de3_8 | 0.958 | 4.590 |
| 10 | 146 | 87 | 1de3−1aqz | 1de3_9 | 0.923 | 4.676 |
| 10 | 146 | 87 | 1de3−1aqz | 1de3_10 | 1.000 | 4.596 |
| 11 | 197 | 86 | 1bc9−1pbv | 1bc9_1 | 0.837 | 4.047 |
| 11 | 197 | 86 | 1bc9−1pbv | 1bc9_2 | 0.811 | 5.655 |
| 11 | 197 | 86 | 1bc9−1pbv | 1bc9_3 | 0.905 | 6.463 |
| 11 | 197 | 86 | 1bc9−1pbv | 1bc9_4 | 0.653 | 6.498 |
| 11 | 197 | 86 | 1bc9−1pbv | 1bc9_5 | 0.642 | 6.624 |
| 11 | 197 | 86 | 1bc9−1pbv | 1bc9_6 | 0.905 | 7.030 |
| 11 | 197 | 86 | 1bc9−1pbv | 1bc9_7 | 0.611 | 7.180 |
| 11 | 197 | 86 | 1bc9−1pbv | 1bc9_8 | 0.847 | 7.526 |
| 11 | 197 | 86 | 1bc9−1pbv | 1bc9_9 | 0.916 | 7.761 |
| 11 | 197 | 86 | 1bc9−1pbv | 1bc9_10 | 0.863 | 7.246 |
| 11 | 197 | 86 | 1pbv−1bc9 | 1pbv_1 | 0.963 | 2.976 |
| 11 | 197 | 86 | 1pbv−1bc9 | 1pbv_2 | 0.937 | 3.322 |
| 11 | 197 | 86 | 1pbv−1bc9 | 1pbv_3 | 0.858 | 3.683 |
| 11 | 197 | 86 | 1pbv−1bc9 | 1pbv_4 | 0.932 | 4.048 |
| 11 | 197 | 86 | 1pbv−1bc9 | 1pbv_5 | 0.963 | 4.382 |
| 11 | 197 | 86 | 1pbv−1bc9 | 1pbv_6 | 0.874 | 4.020 |
| 11 | 197 | 86 | 1pbv−1bc9 | 1pbv_7 | 0.858 | 4.600 |
| 11 | 197 | 86 | 1pbv−1bc9 | 1pbv_8 | 0.911 | 4.496 |
| 11 | 197 | 86 | 1pbv−1bc9 | 1pbv_9 | 0.789 | 4.735 |
| 14 | 88 | 85 | 1l5d−2bn2 | 1l5d_1 | 0.951 | 4.337 |
| 14 | 88 | 85 | 1l5d−2bn2 | 1l5d_2 | 0.902 | 5.315 |
| 14 | 88 | 85 | 1l5d−2bn2 | 1l5d_3 | 0.805 | 4.931 |
| 14 | 88 | 85 | 1l5d−2bn2 | 1l5d_4 | 0.793 | 5.478 |
| 14 | 88 | 85 | 1l5d−2bn2 | 1l5d_5 | 0.756 | 5.827 |
| 14 | 88 | 85 | 1l5d−2bn2 | 1l5d_6 | 0.890 | 4.800 |
| 14 | 88 | 85 | 1l5d−2bn2 | 1l5d_7 | 0.732 | 5.426 |
| 14 | 88 | 85 | 1l5d−2bn2 | 1l5d_8 | 0.720 | 5.439 |
| 14 | 88 | 85 | 1l5d−2bn2 | 1l5d_9 | 0.805 | 5.190 |
| 14 | 88 | 85 | 1l5d−2bn2 | 1l5d_10 | 0.683 | 4.940 |
| 15 | 65 | 84 | 1azp−1c8c | 1azp_1 | 0.714 | 3.429 |
| 15 | 65 | 84 | 1azp−1c8c | 1azp_2 | 0.714 | 3.969 |
| 15 | 65 | 84 | 1azp−1c8c | 1azp_3 | 0.714 | 3.536 |
| 15 | 65 | 84 | 1azp−1c8c | 1azp_4 | 0.714 | 3.622 |
| 15 | 65 | 84 | 1azp−1c8c | 1azp_5 | 0.698 | 4.206 |
| 15 | 65 | 84 | 1azp−1c8c | 1azp_6 | 0.698 | 4.167 |
| 15 | 65 | 84 | 1azp−1c8c | 1azp_7 | 0.714 | 4.309 |
| 15 | 65 | 84 | 1azp−1c8c | 1azp_8 | 0.714 | 5.053 |
| 15 | 65 | 84 | 1azp−1c8c | 1azp_9 | 0.698 | 3.431 |
| 15 | 65 | 84 | 1azp−1c8c | 1azp_10 | 0.698 | 4.674 |
| 16 | 285 | 84 | 1qqf−1c3d | 1qqf_1 | 1.000 | 1.878 |
| 16 | 285 | 84 | 1qqf−1c3d | 1qqf_2 | 1.000 | 1.775 |
| 16 | 285 | 84 | 1qqf−1c3d | 1qqf_3 | 0.996 | 2.032 |
| 16 | 285 | 84 | 1qqf−1c3d | 1qqf_4 | 0.996 | 2.271 |
| 16 | 285 | 84 | 1qqf−1c3d | 1qqf_5 | 0.975 | 2.592 |
| 16 | 285 | 84 | 1qqf−1c3d | 1qqf_6 | 0.993 | 2.670 |
| 16 | 285 | 84 | 1qqf−1c3d | 1qqf_7 | 0.975 | 2.865 |
| 16 | 285 | 84 | 1qqf−1c3d | 1qqf_8 | 0.975 | 2.852 |
| 16 | 285 | 84 | 1qqf−1c3d | 1qqf_9 | 0.975 | 2.628 |
| 16 | 285 | 84 | 1qqf−1c3d | 1qqf_10 | 0.960 | 2.568 |
| 16 | 285 | 84 | 1c3d−1qqf | 1c3d_1 | 1.000 | 1.616 |
| 16 | 285 | 84 | 1c3d−1qqf | 1c3d_2 | 1.000 | 1.975 |
| 16 | 285 | 84 | 1c3d−1qqf | 1c3d_3 | 1.000 | 2.078 |
| 16 | 285 | 84 | 1c3d−1qqf | 1c3d_4 | 0.982 | 2.336 |
| 16 | 285 | 84 | 1c3d−1qqf | 1c3d_5 | 0.942 | 2.337 |
| 16 | 285 | 84 | 1c3d−1qqf | 1c3d_6 | 0.946 | 2.466 |
| 16 | 285 | 84 | 1c3d−1qqf | 1c3d_7 | 0.957 | 2.354 |
| 16 | 285 | 84 | 1c3d−1qqf | 1c3d_8 | 0.931 | 2.724 |
| 16 | 285 | 84 | 1c3d−1qqf | 1c3d_9 | 0.957 | 2.576 |
| 16 | 285 | 84 | 1c3d−1qqf | 1c3d_10 | 0.938 | 2.694 |
| 18 | 317 | 84 | 4lip−1cvl | 4lip_1 | 0.935 | 2.012 |
| 18 | 317 | 84 | 4lip−1cvl | 4lip_2 | 0.938 | 2.278 |
| 18 | 317 | 84 | 4lip−1cvl | 4lip_3 | 0.896 | 2.281 |
| 18 | 317 | 84 | 4lip−1cvl | 4lip_4 | 0.938 | 2.557 |
| 18 | 317 | 84 | 4lip−1cvl | 4lip_5 | 0.893 | 2.914 |
| 18 | 317 | 84 | 4lip−1cvl | 4lip_6 | 0.844 | 3.214 |
| 18 | 317 | 84 | 4lip−1cvl | 4lip_7 | 0.834 | 3.900 |
| 19 | 68 | 84 | 1a7w−1b67 | 1a7w_1 | 1.000 | 2.282 |
| 19 | 68 | 84 | 1a7w−1b67 | 1a7w_2 | 0.765 | 6.033 |
| 19 | 68 | 84 | 1a7w−1b67 | 1a7w_3 | 1.000 | 5.289 |
| 19 | 68 | 84 | 1a7w−1b67 | 1a7w_4 | 0.368 | 8.834 |
| 19 | 68 | 84 | 1a7w−1b67 | 1a7w_5 | 0.250 | 9.344 |
| 19 | 68 | 84 | 1a7w−1b67 | 1a7w_6 | 0.118 | 9.825 |
| 19 | 68 | 84 | 1a7w−1b67 | 1a7w_9 | 0.235 | 9.280 |
| 20 | 213 | 83 | 1n45−1dvg | 1n45_1 | 0.929 | 2.993 |
| 20 | 213 | 83 | 1n45−1dvg | 1n45_2 | 0.925 | 3.397 |
| 20 | 213 | 83 | 1n45−1dvg | 1n45_3 | 0.953 | 3.436 |
| 25 | 124 | 82 | 1grw−1msp | 1grw_1 | 0.960 | 1.658 |
| 26 | 511 | 82 | 1mty−1mhy | 1mty_1 | 1.000 | 1.627 |
| 26 | 511 | 82 | 1mhy−1mty | 1mhy_1 | 1.000 | 1.677 |
| 26 | 511 | 82 | 1mhy−1mty | 1mhy_2 | 0.990 | 1.747 |
| 26 | 511 | 82 | 1mhy−1mty | 1mhy_3 | 0.980 | 1.897 |
| 26 | 511 | 82 | 1mhy−1mty | 1mhy_4 | 0.990 | 1.940 |
| 28 | 68 | 82 | 1c9s−1wap | 1c9s_1 | 1.000 | 1.949 |
| 28 | 68 | 82 | 1c9s−1wap | 1c9s_2 | 1.000 | 2.656 |
| 28 | 68 | 82 | 1c9s−1wap | 1c9s_3 | 1.000 | 2.750 |
| 28 | 68 | 82 | 1c9s−1wap | 1c9s_4 | 1.000 | 2.852 |
| 28 | 68 | 82 | 1c9s−1wap | 1c9s_5 | 1.000 | 2.835 |
| 28 | 68 | 82 | 1c9s−1wap | 1c9s_6 | 1.000 | 2.844 |
| 28 | 68 | 82 | 1c9s−1wap | 1c9s_7 | 1.000 | 2.988 |
| 28 | 68 | 82 | 1c9s−1wap | 1c9s_8 | 1.000 | 3.341 |
| 28 | 68 | 82 | 1c9s−1wap | 1c9s_9 | 1.000 | 2.941 |
| 28 | 68 | 82 | 1c9s−1wap | 1c9s_10 | 1.000 | 2.771 |
| 28 | 68 | 82 | 1wap−1c9s | 1wap_1 | 1.000 | 0.927 |
| 28 | 68 | 82 | 1wap−1c9s | 1wap_2 | 1.000 | 0.959 |
| 28 | 68 | 82 | 1wap−1c9s | 1wap_3 | 1.000 | 1.061 |
| 28 | 68 | 82 | 1wap−1c9s | 1wap_4 | 1.000 | 0.968 |
| 28 | 68 | 82 | 1wap−1c9s | 1wap_5 | 1.000 | 1.046 |
| 28 | 68 | 82 | 1wap−1c9s | 1wap_6 | 1.000 | 0.912 |
| 29 | 123 | 81 | 1i6z−1hx1 | 1i6z_1 | 0.991 | 6.919 |
| 29 | 123 | 81 | 1i6z−1hx1 | 1i6z_2 | 0.991 | 8.948 |
| 29 | 123 | 81 | 1i6z−1hx1 | 1i6z_3 | 0.929 | 9.886 |
| 29 | 123 | 81 | 1i6z−1hx1 | 1i6z_4 | 0.991 | 9.314 |
| 30 | 60 | 81 | 1f4n−1rpo | 1f4n_1 | 1.000 | 1.671 |
| 30 | 60 | 81 | 1f4n−1rpo | 1f4n_2 | 1.000 | 1.714 |
| 30 | 60 | 81 | 1f4n−1rpo | 1f4n_3 | 1.000 | 1.834 |
| 30 | 60 | 81 | 1f4n−1rpo | 1f4n_4 | 1.000 | 1.604 |
| 30 | 60 | 81 | 1f4n−1rpo | 1f4n_5 | 1.000 | 1.925 |
| 30 | 60 | 81 | 1f4n−1rpo | 1f4n_6 | 1.000 | 1.687 |
| 30 | 60 | 81 | 1f4n−1rpo | 1f4n_7 | 0.898 | 1.679 |
| 30 | 60 | 81 | 1f4n−1rpo | 1f4n_8 | 1.000 | 1.663 |
| 30 | 60 | 81 | 1f4n−1rpo | 1f4n_9 | 1.000 | 1.614 |
| 30 | 60 | 81 | 1f4n−1rpo | 1f4n_10 | 0.915 | 2.050 |
| 30 | 60 | 81 | 1rpo−1f4n | 1rpo_1 | 0.983 | 2.958 |
| 30 | 60 | 81 | 1rpo−1f4n | 1rpo_2 | 0.898 | 4.047 |
| 30 | 60 | 81 | 1rpo−1f4n | 1rpo_3 | 0.458 | 3.012 |
| 30 | 60 | 81 | 1rpo−1f4n | 1rpo_4 | 0.898 | 2.922 |
| 30 | 60 | 81 | 1rpo−1f4n | 1rpo_5 | 0.915 | 3.693 |
| 30 | 60 | 81 | 1rpo−1f4n | 1rpo_6 | 0.898 | 4.211 |
| 30 | 60 | 81 | 1rpo−1f4n | 1rpo_7 | 0.729 | 4.169 |
| 30 | 60 | 81 | 1rpo−1f4n | 1rpo_8 | 0.695 | 4.793 |
| 30 | 60 | 81 | 1rpo−1f4n | 1rpo_9 | 0.508 | 4.244 |
| 30 | 60 | 81 | 1rpo−1f4n | 1rpo_10 | 0.915 | 4.458 |
| 32 | 133 | 80 | 1jac−1jot | 1jac_1 | 1.000 | 1.219 |
| 32 | 133 | 80 | 1jac−1jot | 1jac_2 | 1.000 | 1.349 |
| 32 | 133 | 80 | 1jot−1jac | 1jot_1 | 1.000 | 1.056 |
| 32 | 133 | 80 | 1jot−1jac | 1jot_2 | 1.000 | 1.150 |
| 32 | 133 | 80 | 1jot−1jac | 1jot_3 | 1.000 | 1.313 |
| 32 | 133 | 80 | 1jot−1jac | 1jot_4 | 1.000 | 1.536 |
| 32 | 133 | 80 | 1jot−1jac | 1jot_5 | 1.000 | 1.915 |
| 32 | 133 | 80 | 1jot−1jac | 1jot_6 | 1.000 | 1.710 |
| 32 | 133 | 80 | 1jot−1jac | 1jot_7 | 1.000 | 1.927 |
| 32 | 133 | 80 | 1jot−1jac | 1jot_8 | 1.000 | 1.810 |
| 33 | 353 | 79 | 1ksa−4bcl | 1ksa_1 | 0.997 | 2.657 |
| 33 | 353 | 79 | 1ksa−4bcl | 1ksa_2 | 0.997 | 2.940 |
| 34 | 312 | 79 | 2mas−1ezr | 2mas_1 | 1.000 | 1.577 |
| 37 | 345 | 79 | 2lbp−2liv | 2lbp_1 | 0.983 | 2.369 |
| 37 | 345 | 79 | 2lbp−2liv | 2lbp_2 | 0.983 | 3.168 |
| 37 | 345 | 79 | 2lbp−2liv | 2lbp_3 | 0.930 | 3.578 |
| 37 | 345 | 79 | 2liv−2lbp | 2liv_1 | 1.000 | 2.302 |
| 37 | 345 | 79 | 2liv−2lbp | 2liv_2 | 0.985 | 2.620 |
| 37 | 345 | 79 | 2liv−2lbp | 2liv_3 | 0.968 | 4.211 |
| 46 | 60 | 76 | 1otf−1bjp | 1otf_1 | 1.000 | 1.411 |
| 46 | 60 | 76 | 1otf−1bjp | 1otf_2 | 1.000 | 1.549 |
| 46 | 60 | 76 | 1otf−1bjp | 1otf_3 | 1.000 | 1.915 |
| 46 | 60 | 76 | 1otf−1bjp | 1otf_4 | 1.000 | 1.946 |
| 46 | 60 | 76 | 1otf−1bjp | 1otf_5 | 0.983 | 2.004 |
| 46 | 60 | 76 | 1otf−1bjp | 1otf_6 | 0.966 | 1.977 |
| 46 | 60 | 76 | 1otf−1bjp | 1otf_7 | 0.966 | 2.333 |
| 46 | 60 | 76 | 1otf−1bjp | 1otf_8 | 1.000 | 2.354 |
| 46 | 60 | 76 | 1otf−1bjp | 1otf_9 | 0.881 | 2.369 |
| 46 | 60 | 76 | 1otf−1bjp | 1otf_10 | 1.000 | 2.398 |
| 46 | 60 | 76 | 1bjp−1otf | 1bjp_1 | 1.000 | 3.138 |
| 48 | 65 | 74 | 1g72−1h4i | 1g72_1 | 1.000 | 1.678 |
| 48 | 65 | 74 | 1g72−1h4i | 1g72_2 | 0.333 | 3.230 |
| 51 | 100 | 74 | 2kau−1ubp | 2kau_1 | 1.000 | 1.541 |
| 53 | 837 | 73 | 1yge−1byt | 1yge_1 | 0.964 | 2.309 |
| 54 | 86 | 73 | 1ycq−1ycr | 1ycq_1 | 1.000 | 1.961 |
| 54 | 86 | 73 | 1ycq−1ycr | 1ycq_2 | 1.000 | 2.436 |
| 54 | 86 | 73 | 1ycq−1ycr | 1ycq_3 | 1.000 | 2.243 |
| 54 | 86 | 73 | 1ycq−1ycr | 1ycq_4 | 1.000 | 2.075 |
| 54 | 86 | 73 | 1ycq−1ycr | 1ycq_5 | 1.000 | 2.160 |
| 54 | 86 | 73 | 1ycq−1ycr | 1ycq_6 | 1.000 | 2.022 |
| 54 | 86 | 73 | 1ycq−1ycr | 1ycq_7 | 1.000 | 2.216 |
| 54 | 86 | 73 | 1ycq−1ycr | 1ycq_8 | 1.000 | 2.244 |
| 54 | 86 | 73 | 1ycq−1ycr | 1ycq_9 | 0.859 | 2.166 |
| 54 | 86 | 73 | 1ycq−1ycr | 1ycq_10 | 0.929 | 2.134 |
| 54 | 86 | 73 | 1ycr−1ycq | 1ycr_1 | 1.000 | 1.642 |
| 54 | 86 | 73 | 1ycr−1ycq | 1ycr_2 | 1.000 | 1.490 |
| 54 | 86 | 73 | 1ycr−1ycq | 1ycr_3 | 1.000 | 1.587 |
| 54 | 86 | 73 | 1ycr−1ycq | 1ycr_4 | 1.000 | 1.523 |
| 54 | 86 | 73 | 1ycr−1ycq | 1ycr_5 | 1.000 | 1.463 |
| 54 | 86 | 73 | 1ycr−1ycq | 1ycr_6 | 1.000 | 1.152 |
| 54 | 86 | 73 | 1ycr−1ycq | 1ycr_7 | 1.000 | 1.309 |
| 54 | 86 | 73 | 1ycr−1ycq | 1ycr_8 | 1.000 | 1.313 |
| 54 | 86 | 73 | 1ycr−1ycq | 1ycr_9 | 1.000 | 1.605 |
| 54 | 86 | 73 | 1ycr−1ycq | 1ycr_10 | 1.000 | 1.708 |
| 55 | 282 | 73 | 1dqz−1f0n | 1dqz_1 | 1.000 | 1.578 |
| 55 | 282 | 73 | 1dqz−1f0n | 1dqz_2 | 1.000 | 1.622 |
| 55 | 282 | 73 | 1dqz−1f0n | 1dqz_3 | 1.000 | 1.767 |
| 55 | 282 | 73 | 1dqz−1f0n | 1dqz_4 | 0.979 | 1.863 |
| 55 | 282 | 73 | 1f0n−1dqz | 1f0n_1 | 0.979 | 1.737 |
| 55 | 282 | 73 | 1f0n−1dqz | 1f0n_2 | 0.979 | 1.864 |
| 55 | 282 | 73 | 1f0n−1dqz | 1f0n_3 | 0.975 | 1.976 |
| 55 | 282 | 73 | 1f0n−1dqz | 1f0n_4 | 0.982 | 2.105 |
| 55 | 282 | 73 | 1f0n−1dqz | 1f0n_5 | 0.996 | 2.499 |
| 55 | 282 | 73 | 1f0n−1dqz | 1f0n_6 | 0.971 | 2.276 |
| 55 | 282 | 73 | 1f0n−1dqz | 1f0n_7 | 0.996 | 2.435 |

n: internal numbering; length of alignment; % sequence%ID (cf. Homstrad); refA−refB: reference alignment; altA: alternative snapshot for A; SP: sum−of−pairs score of alt. alignment w/r reference

| n | length | %ID | refA-refB | altA | SP | RMSD |
|---|---|---|---|---|---|---|
| 55 | 282 | 73 | 1f0n-1dqz | 1f0n_8 | 0.954 | 2.753 |
| 55 | 282 | 73 | 1f0n-1dqz | 1f0n_9 | 0.950 | 3.169 |
| 55 | 282 | 73 | 1f0n-1dqz | 1f0n_10 | 0.950 | 3.127 |
| 56 | 98 | 73 | 1jo0-1ln4 | 1jo0_1 | 1.000 | 2.456 |
| 56 | 98 | 73 | 1jo0-1ln4 | 1jo0_2 | 1.000 | 2.398 |
| 56 | 98 | 73 | 1jo0-1ln4 | 1jo0_3 | 1.000 | 2.128 |
| 56 | 98 | 73 | 1jo0-1ln4 | 1jo0_4 | 1.000 | 2.188 |
| 56 | 98 | 73 | 1jo0-1ln4 | 1jo0_5 | 1.000 | 2.311 |
| 56 | 98 | 73 | 1ln4-1jo0 | 1ln4_1 | 1.000 | 1.809 |
| 56 | 98 | 73 | 1ln4-1jo0 | 1ln4_2 | 1.000 | 1.746 |
| 56 | 98 | 73 | 1ln4-1jo0 | 1ln4_3 | 1.000 | 1.549 |
| 56 | 98 | 73 | 1ln4-1jo0 | 1ln4_4 | 1.000 | 2.031 |
| 56 | 98 | 73 | 1ln4-1jo0 | 1ln4_5 | 1.000 | 2.065 |
| 56 | 98 | 73 | 1ln4-1jo0 | 1ln4_6 | 1.000 | 2.143 |
| 56 | 98 | 73 | 1ln4-1jo0 | 1ln4_7 | 0.825 | 2.693 |
| 56 | 98 | 73 | 1ln4-1jo0 | 1ln4_8 | 0.825 | 3.082 |
| 56 | 98 | 73 | 1ln4-1jo0 | 1ln4_9 | 0.825 | 3.871 |
| 56 | 98 | 73 | 1ln4-1jo0 | 1ln4_10 | 0.948 | 2.794 |
| 57 | 107 | 73 | 1evh-1qc6 | 1evh_1 | 1.000 | 2.005 |
| 57 | 107 | 73 | 1evh-1qc6 | 1evh_2 | 0.990 | 2.113 |
| 57 | 107 | 73 | 1evh-1qc6 | 1evh_3 | 0.933 | 2.127 |
| 57 | 107 | 73 | 1evh-1qc6 | 1evh_4 | 0.933 | 2.097 |
| 57 | 107 | 73 | 1evh-1qc6 | 1evh_5 | 0.933 | 2.271 |
| 57 | 107 | 73 | 1evh-1qc6 | 1evh_6 | 0.942 | 2.439 |
| 57 | 107 | 73 | 1evh-1qc6 | 1evh_7 | 0.942 | 2.601 |
| 57 | 107 | 73 | 1evh-1qc6 | 1evh_8 | 0.990 | 2.433 |
| 57 | 107 | 73 | 1evh-1qc6 | 1evh_9 | 0.942 | 2.563 |
| 57 | 107 | 73 | 1evh-1qc6 | 1evh_10 | 0.942 | 2.492 |
| 58 | 140 | 72 | 1anu-1aoh | 1anu_5 | 0.957 | 2.016 |
| 58 | 140 | 72 | 1anu-1aoh | 1anu_6 | 1.000 | 2.221 |
| 58 | 140 | 72 | 1anu-1aoh | 1anu_7 | 0.928 | 2.174 |
| 58 | 140 | 72 | 1anu-1aoh | 1anu_8 | 0.971 | 2.158 |
| 58 | 140 | 72 | 1anu-1aoh | 1anu_9 | 0.935 | 2.216 |
| 58 | 140 | 72 | 1anu-1aoh | 1anu_10 | 0.928 | 2.515 |
| 58 | 140 | 72 | 1anu-1aoh | 1anu_1 | 1.000 | 1.488 |
| 58 | 140 | 72 | 1anu-1aoh | 1anu_2 | 1.000 | 2.002 |
| 58 | 140 | 72 | 1anu-1aoh | 1anu_3 | 1.000 | 1.790 |
| 58 | 140 | 72 | 1anu-1aoh | 1anu_4 | 0.957 | 2.069 |
| 60 | 213 | 71 | 1bif-1k6m | 1bif_1 | 0.967 | 2.331 |
| 60 | 213 | 71 | 1bif-1k6m | 1bif_2 | 0.967 | 2.840 |
| 60 | 213 | 71 | 1bif-1k6m | 1bif_3 | 0.967 | 3.340 |
| 60 | 213 | 71 | 1bif-1k6m | 1bif_4 | 0.967 | 3.618 |
| 60 | 213 | 71 | 1bif-1k6m | 1bif_5 | 0.873 | 3.411 |
| 60 | 213 | 71 | 1bif-1k6m | 1bif_6 | 0.873 | 4.106 |
| 60 | 213 | 71 | 1bif-1k6m | 1bif_7 | 0.840 | 5.097 |
| 60 | 213 | 71 | 1k6m-1bif | 1k6m_1 | 1.000 | 2.333 |
| 61 | 250 | 71 | 1hcz-1cfm | 1hcz_1 | 0.976 | 3.132 |
| 61 | 250 | 71 | 1hcz-1cfm | 1hcz_2 | 0.996 | 3.357 |
| 61 | 250 | 71 | 1hcz-1cfm | 1hcz_3 | 1.000 | 3.376 |
| 61 | 250 | 71 | 1hcz-1cfm | 1hcz_4 | 0.940 | 3.180 |
| 61 | 250 | 71 | 1cfm-1hcz | 1cfm_1 | 1.000 | 3.117 |
| 62 | 553 | 71 | 1eex-1iwp | 1eex_1 | 1.000 | 1.819 |
| 66 | 69 | 71 | 1ctf-1dd3 | 1ctf_1 | 1.000 | 1.425 |
| 66 | 69 | 71 | 1ctf-1dd3 | 1ctf_2 | 1.000 | 1.445 |
| 66 | 69 | 71 | 1ctf-1dd3 | 1ctf_3 | 1.000 | 1.814 |
| 66 | 69 | 71 | 1ctf-1dd3 | 1ctf_4 | 0.926 | 1.213 |
| 66 | 69 | 71 | 1ctf-1dd3 | 1ctf_5 | 0.926 | 1.341 |
| 66 | 69 | 71 | 1ctf-1dd3 | 1ctf_6 | 1.000 | 1.462 |
| 66 | 69 | 71 | 1ctf-1dd3 | 1ctf_7 | 1.000 | 1.175 |
| 66 | 69 | 71 | 1ctf-1dd3 | 1ctf_8 | 0.926 | 1.548 |
| 66 | 69 | 71 | 1ctf-1dd3 | 1ctf_9 | 1.000 | 1.407 |
| 66 | 69 | 71 | 1ctf-1dd3 | 1ctf_10 | 1.000 | 1.309 |
| 68 | 210 | 70 | 1yer-1ah6 | 1yer_1 | 0.966 | 2.586 |
| 68 | 210 | 70 | 1yer-1ah6 | 1yer_2 | 0.966 | 2.996 |
| 68 | 210 | 70 | 1yer-1ah6 | 1yer_3 | 0.937 | 3.131 |
| 68 | 210 | 70 | 1yer-1ah6 | 1yer_4 | 0.932 | 3.109 |
| 68 | 210 | 70 | 1yer-1ah6 | 1yer_5 | 0.937 | 3.104 |
| 68 | 210 | 70 | 1yer-1ah6 | 1yer_6 | 0.937 | 2.794 |
| 68 | 210 | 70 | 1yer-1ah6 | 1yer_7 | 0.937 | 3.050 |
| 68 | 210 | 70 | 1yer-1ah6 | 1yer_8 | 0.942 | 2.804 |
| 68 | 210 | 70 | 1yer-1ah6 | 1yer_9 | 0.971 | 2.746 |

| n | length | %ID | refA-refB | altA | SP | RMSD |
|---|---|---|---|---|---|---|
| 68 | 210 | 70 | 1yer-1ah6 | 1yer_10 | 0.913 | 2.711 |
| 68 | 210 | 70 | 1ah6-1yer | 1ah6_1 | 1.000 | 2.239 |
| 68 | 210 | 70 | 1ah6-1yer | 1ah6_2 | 0.971 | 3.083 |
| 68 | 210 | 70 | 1ah6-1yer | 1ah6_3 | 0.961 | 2.783 |
| 68 | 210 | 70 | 1ah6-1yer | 1ah6_4 | 0.976 | 2.820 |
| 68 | 210 | 70 | 1ah6-1yer | 1ah6_5 | 0.971 | 2.712 |
| 68 | 210 | 70 | 1ah6-1yer | 1ah6_7 | 0.966 | 3.327 |
| 68 | 210 | 70 | 1ah6-1yer | 1ah6_8 | 0.971 | 3.734 |
| 68 | 210 | 70 | 1ah6-1yer | 1ah6_9 | 0.942 | 2.957 |
| 68 | 210 | 70 | 1ah6-1yer | 1ah6_10 | 0.971 | 2.929 |
| 70 | 277 | 70 | 2nip-1cp2 | 2nip_1 | 0.963 | 2.035 |
| 70 | 277 | 70 | 2nip-1cp2 | 2nip_2 | 0.929 | 2.089 |
| 70 | 277 | 70 | 2nip-1cp2 | 2nip_3 | 0.914 | 2.416 |
| 70 | 277 | 70 | 1cp2-2nip | 1cp2_1 | 0.993 | 2.368 |
| 70 | 277 | 70 | 1cp2-2nip | 1cp2_2 | 0.933 | 2.836 |
| 70 | 277 | 70 | 1cp2-2nip | 1cp2_3 | 0.922 | 2.819 |
| 70 | 277 | 70 | 1cp2-2nip | 1cp2_4 | 0.918 | 3.088 |
| 70 | 277 | 70 | 1cp2-2nip | 1cp2_5 | 0.881 | 3.291 |
| 70 | 277 | 70 | 1cp2-2nip | 1cp2_6 | 0.922 | 3.163 |
| 73 | 52 | 69 | 1tih-4sgb | 1tih_1 | 1.000 | 2.415 |
| 73 | 52 | 69 | 1tih-4sgb | 1tih_2 | 1.000 | 2.970 |
| 73 | 52 | 69 | 1tih-4sgb | 1tih_3 | 1.000 | 3.119 |
| 73 | 52 | 69 | 1tih-4sgb | 1tih_4 | 1.000 | 3.136 |
| 73 | 52 | 69 | 1tih-4sgb | 1tih_5 | 1.000 | 3.767 |
| 73 | 52 | 69 | 1tih-4sgb | 1tih_6 | 0.765 | 4.319 |
| 73 | 52 | 69 | 1tih-4sgb | 1tih_7 | 1.000 | 4.442 |
| 73 | 52 | 69 | 1tih-4sgb | 1tih_8 | 1.000 | 3.917 |
| 73 | 52 | 69 | 1tih-4sgb | 1tih_9 | 1.000 | 4.409 |
| 73 | 52 | 69 | 1tih-4sgb | 1tih_10 | 0.863 | 4.275 |
| 73 | 52 | 69 | 4sgb-1tih | 4sgb_1 | 1.000 | 1.659 |
| 73 | 52 | 69 | 4sgb-1tih | 4sgb_2 | 1.000 | 2.567 |
| 73 | 52 | 69 | 4sgb-1tih | 4sgb_3 | 1.000 | 2.256 |
| 73 | 52 | 69 | 4sgb-1tih | 4sgb_4 | 1.000 | 2.502 |
| 73 | 52 | 69 | 4sgb-1tih | 4sgb_5 | 1.000 | 2.462 |
| 73 | 52 | 69 | 4sgb-1tih | 4sgb_6 | 0.843 | 2.474 |
| 73 | 52 | 69 | 4sgb-1tih | 4sgb_7 | 1.000 | 2.221 |
| 73 | 52 | 69 | 4sgb-1tih | 4sgb_8 | 0.843 | 2.088 |
| 73 | 52 | 69 | 4sgb-1tih | 4sgb_9 | 0.843 | 2.493 |
| 76 | 150 | 68 | 1wyk-1vcp | 1wyk_1 | 0.987 | 1.399 |
| 76 | 150 | 68 | 1wyk-1vcp | 1wyk_2 | 0.966 | 1.937 |
| 76 | 150 | 68 | 1wyk-1vcp | 1wyk_3 | 1.000 | 2.116 |
| 76 | 150 | 68 | 1wyk-1vcp | 1wyk_4 | 1.000 | 2.262 |
| 76 | 150 | 68 | 1wyk-1vcp | 1wyk_5 | 1.000 | 2.318 |
| 76 | 150 | 68 | 1wyk-1vcp | 1wyk_6 | 0.953 | 2.301 |
| 76 | 150 | 68 | 1vcp-1wyk | 1vcp_1 | 0.966 | 2.116 |
| 77 | 69 | 68 | 1kjs-1c5a | 1kjs_1 | 0.641 | 2.831 |
| 77 | 69 | 68 | 1kjs-1c5a | 1kjs_2 | 0.641 | 3.206 |
| 77 | 69 | 68 | 1kjs-1c5a | 1kjs_3 | 0.625 | 3.620 |
| 77 | 69 | 68 | 1kjs-1c5a | 1kjs_4 | 0.422 | 4.077 |
| 77 | 69 | 68 | 1kjs-1c5a | 1kjs_5 | 0.469 | 4.486 |
| 77 | 69 | 68 | 1kjs-1c5a | 1kjs_6 | 0.516 | 4.412 |
| 77 | 69 | 68 | 1kjs-1c5a | 1kjs_7 | 0.562 | 4.459 |
| 77 | 69 | 68 | 1kjs-1c5a | 1kjs_8 | 0.344 | 4.471 |
| 77 | 69 | 68 | 1kjs-1c5a | 1kjs_9 | 0.406 | 4.566 |
| 77 | 69 | 68 | 1kjs-1c5a | 1kjs_10 | 0.156 | 4.783 |
| 78 | 126 | 68 | 2lis-3lyn | 2lis_1 | 1.000 | 3.261 |
| 78 | 126 | 68 | 2lis-3lyn | 2lis_2 | 0.951 | 3.816 |
| 78 | 126 | 68 | 2lis-3lyn | 2lis_3 | 0.943 | 3.984 |
| 78 | 126 | 68 | 2lis-3lyn | 2lis_4 | 0.959 | 4.370 |
| 78 | 126 | 68 | 2lis-3lyn | 2lis_5 | 0.959 | 4.594 |
| 78 | 126 | 68 | 2lis-3lyn | 2lis_6 | 0.959 | 4.360 |
| 78 | 126 | 68 | 2lis-3lyn | 2lis_7 | 0.926 | 4.618 |
| 78 | 126 | 68 | 2lis-3lyn | 2lis_8 | 1.000 | 4.593 |
| 78 | 126 | 68 | 2lis-3lyn | 2lis_9 | 0.951 | 4.596 |
| 78 | 126 | 68 | 2lis-3lyn | 2lis_10 | 1.000 | 3.801 |
| 78 | 126 | 68 | 3lyn-2lis | 3lyn_1 | 1.000 | 1.904 |
| 78 | 126 | 68 | 3lyn-2lis | 3lyn_2 | 1.000 | 2.596 |
| 78 | 126 | 68 | 3lyn-2lis | 3lyn_3 | 0.951 | 2.915 |
| 78 | 126 | 68 | 3lyn-2lis | 3lyn_4 | 0.943 | 3.646 |
| 78 | 126 | 68 | 3lyn-2lis | 3lyn_5 | 0.943 | 3.543 |
| 78 | 126 | 68 | 3lyn-2lis | 3lyn_6 | 0.934 | 3.421 |

| n | length | %ID | refA-refB | altA | SP | RMSD |
|---|---|---|---|---|---|---|
| 78 | 126 | 68 | 3lyn-2lis | 3lyn_7 | 0.926 | 4.211 |
| 78 | 126 | 68 | 3lyn-2lis | 3lyn_8 | 0.902 | 5.154 |
| 78 | 126 | 68 | 3lyn-2lis | 3lyn_9 | 0.803 | 6.166 |
| 78 | 126 | 68 | 3lyn-2lis | 3lyn_10 | 0.869 | 6.814 |
| 80 | 142 | 68 | 1vlt-2asr | 1vlt_1 | 0.971 | 2.706 |
| 80 | 142 | 68 | 1vlt-2asr | 1vlt_2 | 0.878 | 2.947 |
| 80 | 142 | 68 | 1vlt-2asr | 1vlt_3 | 0.906 | 3.051 |
| 80 | 142 | 68 | 1vlt-2asr | 1vlt_4 | 0.734 | 3.425 |
| 80 | 142 | 68 | 1vlt-2asr | 1vlt_5 | 0.878 | 2.989 |
| 80 | 142 | 68 | 2asr-1vlt | 2asr_1 | 0.993 | 1.442 |
| 80 | 142 | 68 | 2asr-1vlt | 2asr_2 | 0.964 | 2.195 |
| 80 | 142 | 68 | 2asr-1vlt | 2asr_3 | 0.971 | 2.217 |
| 80 | 142 | 68 | 2asr-1vlt | 2asr_4 | 0.935 | 2.685 |
| 80 | 142 | 68 | 2asr-1vlt | 2asr_5 | 0.906 | 2.844 |
| 80 | 142 | 68 | 2asr-1vlt | 2asr_6 | 0.914 | 2.605 |
| 80 | 142 | 68 | 2asr-1vlt | 2asr_7 | 0.906 | 2.782 |
| 80 | 142 | 68 | 2asr-1vlt | 2asr_8 | 0.935 | 2.610 |
| 80 | 142 | 68 | 2asr-1vlt | 2asr_9 | 0.942 | 2.604 |
| 80 | 142 | 68 | 2asr-1vlt | 2asr_10 | 0.906 | 2.730 |
| 81 | 118 | 68 | 1kfq-3pmg | 1kfq_1 | 1.000 | 1.215 |
| 85 | 421 | 67 | 1gff-1cd3 | 1gff_1 | 0.844 | 5.598 |
| 85 | 421 | 67 | 1gff-1cd3 | 1gff_2 | 0.921 | 6.082 |
| 87 | 102 | 67 | 1m42-1lyq | 1m42_1 | 0.830 | 2.894 |
| 87 | 102 | 67 | 1m42-1lyq | 1m42_2 | 0.920 | 2.991 |
| 87 | 102 | 67 | 1m42-1lyq | 1m42_3 | 0.990 | 3.756 |
| 87 | 102 | 67 | 1m42-1lyq | 1m42_4 | 0.870 | 3.539 |
| 87 | 102 | 67 | 1m42-1lyq | 1m42_5 | 0.790 | 3.507 |
| 87 | 102 | 67 | 1m42-1lyq | 1m42_6 | 0.900 | 3.374 |
| 87 | 102 | 67 | 1m42-1lyq | 1m42_7 | 0.900 | 3.960 |
| 87 | 102 | 67 | 1m42-1lyq | 1m42_8 | 0.850 | 3.521 |
| 87 | 102 | 67 | 1m42-1lyq | 1m42_9 | 0.930 | 3.772 |
| 87 | 102 | 67 | 1m42-1lyq | 1m42_10 | 0.900 | 4.237 |
| 88 | 544 | 67 | 1nir-1aoq | 1nir_1 | 0.957 | 2.064 |
| 88 | 544 | 67 | 1nir-1aoq | 1nir_2 | 0.959 | 3.128 |
| 91 | 163 | 67 | 1f1m-1g5z | 1f1m_1 | 0.988 | 1.766 |
| 91 | 163 | 67 | 1g5z-1f1m | 1g5z_1 | 0.944 | 3.173 |
| 91 | 163 | 67 | 1g5z-1f1m | 1g5z_2 | 0.900 | 4.047 |
| 91 | 163 | 67 | 1g5z-1f1m | 1g5z_3 | 0.863 | 3.805 |
| 91 | 163 | 67 | 1g5z-1f1m | 1g5z_4 | 0.881 | 4.112 |
| 91 | 163 | 67 | 1g5z-1f1m | 1g5z_5 | 0.944 | 4.599 |
| 91 | 163 | 67 | 1g5z-1f1m | 1g5z_6 | 0.738 | 6.005 |
| 91 | 163 | 67 | 1g5z-1f1m | 1g5z_7 | 0.738 | 5.674 |
| 91 | 163 | 67 | 1g5z-1f1m | 1g5z_8 | 0.738 | 5.629 |
| 91 | 163 | 67 | 1g5z-1f1m | 1g5z_9 | 0.738 | 6.167 |
| 91 | 163 | 67 | 1g5z-1f1m | 1g5z_10 | 0.688 | 6.148 |
| 94 | 548 | 67 | 1gnl-1gnt | 1gnl_1 | 0.991 | 2.152 |
| 94 | 548 | 67 | 1gnt-1gnl | 1gnt_1 | 0.978 | 2.529 |
| 94 | 548 | 67 | 1gnt-1gnl | 1gnt_2 | 0.961 | 2.753 |
| 94 | 548 | 67 | 1gnt-1gnl | 1gnt_3 | 0.959 | 2.985 |
| 94 | 548 | 67 | 1gnt-1gnl | 1gnt_4 | 0.939 | 3.310 |
| 94 | 548 | 67 | 1gnt-1gnl | 1gnt_5 | 0.931 | 3.211 |
| 96 | 132 | 66 | 1ayo-1edy | 1ayo_1 | 0.977 | 1.902 |
| 96 | 132 | 66 | 1ayo-1edy | 1ayo_2 | 0.938 | 1.655 |
| 96 | 132 | 66 | 1ayo-1edy | 1ayo_3 | 0.953 | 1.557 |
| 96 | 132 | 66 | 1ayo-1edy | 1ayo_4 | 0.953 | 1.696 |
| 96 | 132 | 66 | 1ayo-1edy | 1ayo_5 | 0.946 | 1.570 |
| 96 | 132 | 66 | 1ayo-1edy | 1ayo_6 | 0.953 | 1.586 |
| 96 | 132 | 66 | 1ayo-1edy | 1ayo_7 | 0.946 | 1.904 |
| 96 | 132 | 66 | 1ayo-1edy | 1ayo_8 | 0.946 | 1.982 |
| 96 | 132 | 66 | 1ayo-1edy | 1ayo_9 | 0.946 | 2.074 |
| 96 | 132 | 66 | 1ayo-1edy | 1ayo_10 | 0.992 | 2.436 |
| 96 | 132 | 66 | 1edy-1ayo | 1edy_1 | 0.946 | 1.485 |
| 96 | 132 | 66 | 1edy-1ayo | 1edy_2 | 0.992 | 1.617 |
| 96 | 132 | 66 | 1edy-1ayo | 1edy_3 | 0.992 | 1.959 |
| 96 | 132 | 66 | 1edy-1ayo | 1edy_4 | 0.946 | 1.962 |
| 98 | 242 | 66 | 1dxj-2baa | 1dxj_1 | 0.946 | 2.561 |
| 98 | 242 | 66 | 1dxj-2baa | 1dxj_2 | 0.975 | 2.414 |
| 98 | 242 | 66 | 1dxj-2baa | 1dxj_3 | 0.954 | 2.542 |
| 98 | 242 | 66 | 1dxj-2baa | 1dxj_4 | 0.975 | 2.751 |
| 98 | 242 | 66 | 1dxj-2baa | 1dxj_5 | 0.942 | 2.569 |
| 98 | 242 | 66 | 1dxj-2baa | 1dxj_6 | 0.975 | 2.501 |

n: internal numbering; length of alignment; % sequence%ID (cf. Homstrad); refA-refB: reference alignment; altA: alternative snapshot for A; SP: sum-of-pairs score of alt. alignment w/r reference

| n | length | %ID | refA−refB | altA | SP | RMSD |
|---|---|---|---|---|---|---|
| 98 | 242 | 66 | 1dxj−2baa | 1dxj_7 | 0.942 | 2.328 |
| 98 | 242 | 66 | 1dxj−2baa | 1dxj_8 | 0.967 | 2.626 |
| 98 | 242 | 66 | 1dxj−2baa | 1dxj_9 | 0.971 | 2.697 |
| 98 | 242 | 66 | 1dxj−2baa | 1dxj_10 | 1.000 | 2.843 |
| 100 | 212 | 66 | 1wab−1fxw | 1wab_1 | 1.000 | 1.989 |
| 100 | 212 | 66 | 1wab−1fxw | 1wab_2 | 1.000 | 1.774 |
| 100 | 212 | 66 | 1wab−1fxw | 1wab_3 | 1.000 | 2.293 |
| 100 | 212 | 66 | 1wab−1fxw | 1wab_4 | 1.000 | 2.185 |
| 100 | 212 | 66 | 1wab−1fxw | 1wab_5 | 0.967 | 2.449 |
| 100 | 212 | 66 | 1wab−1fxw | 1wab_6 | 0.943 | 2.797 |
| 100 | 212 | 66 | 1wab−1fxw | 1wab_7 | 0.976 | 2.743 |
| 100 | 212 | 66 | 1wab−1fxw | 1wab_8 | 0.976 | 2.533 |
| 100 | 212 | 66 | 1wab−1fxw | 1wab_9 | 0.976 | 2.530 |
| 100 | 212 | 66 | 1wab−1fxw | 1wab_10 | 0.976 | 2.732 |
| 102 | 316 | 65 | 4pah−1toh | 4pah_1 | 1.000 | 2.006 |
| 102 | 316 | 65 | 4pah−1toh | 4pah_2 | 1.000 | 2.521 |
| 102 | 316 | 65 | 4pah−1toh | 4pah_3 | 1.000 | 2.541 |
| 102 | 316 | 65 | 4pah−1toh | 4pah_4 | 1.000 | 2.755 |
| 102 | 316 | 65 | 4pah−1toh | 4pah_5 | 1.000 | 2.772 |
| 102 | 316 | 65 | 4pah−1toh | 4pah_6 | 0.966 | 2.695 |
| 102 | 316 | 65 | 4pah−1toh | 4pah_7 | 0.980 | 2.661 |
| 102 | 316 | 65 | 4pah−1toh | 4pah_8 | 0.983 | 3.103 |
| 102 | 316 | 65 | 4pah−1toh | 4pah_9 | 1.000 | 2.959 |
| 102 | 316 | 65 | 4pah−1toh | 4pah_10 | 1.000 | 2.895 |
| 105 | 110 | 65 | 1imu−1l4s | 1imu_1 | 0.867 | 3.204 |
| 105 | 110 | 65 | 1imu−1l4s | 1imu_2 | 0.867 | 3.966 |
| 105 | 110 | 65 | 1imu−1l4s | 1imu_3 | 0.867 | 6.068 |
| 105 | 110 | 65 | 1imu−1l4s | 1imu_4 | 0.829 | 6.288 |
| 105 | 110 | 65 | 1imu−1l4s | 1imu_5 | 0.867 | 6.365 |
| 105 | 110 | 65 | 1imu−1l4s | 1imu_6 | 0.848 | 6.202 |
| 105 | 110 | 65 | 1imu−1l4s | 1imu_7 | 0.867 | 6.653 |
| 105 | 110 | 65 | 1imu−1l4s | 1imu_8 | 0.829 | 7.165 |
| 105 | 110 | 65 | 1imu−1l4s | 1imu_9 | 0.829 | 7.635 |
| 105 | 110 | 65 | 1imu−1l4s | 1imu_10 | 0.829 | 7.333 |
| 105 | 110 | 65 | 1l4s−1imu | 1l4s_1 | 0.819 | 7.228 |
| 105 | 110 | 65 | 1l4s−1imu | 1l4s_2 | 0.629 | 8.324 |
| 105 | 110 | 65 | 1l4s−1imu | 1l4s_3 | 0.619 | 7.421 |
| 105 | 110 | 65 | 1l4s−1imu | 1l4s_4 | 0.629 | 9.705 |
| 105 | 110 | 65 | 1l4s−1imu | 1l4s_5 | 0.619 | 9.466 |
| 108 | 434 | 64 | 1pdz−1one | 1pdz_1 | 0.998 | 1.812 |
| 108 | 434 | 64 | 1pdz−1one | 1pdz_2 | 0.981 | 2.162 |
| 108 | 434 | 64 | 1pdz−1one | 1pdz_3 | 0.993 | 2.267 |
| 114 | 103 | 63 | 2pii−2gnk | 2pii_1 | 0.863 | 3.620 |
| 114 | 103 | 63 | 2pii−2gnk | 2pii_2 | 0.853 | 6.280 |
| 114 | 103 | 63 | 2pii−2gnk | 2pii_3 | 0.811 | 6.114 |
| 114 | 103 | 63 | 2pii−2gnk | 2pii_4 | 0.874 | 6.041 |
| 114 | 103 | 63 | 2pii−2gnk | 2pii_5 | 0.842 | 6.378 |
| 114 | 103 | 63 | 2pii−2gnk | 2pii_6 | 0.863 | 5.955 |
| 115 | 87 | 63 | 1e91−1g1e | 1e91_1 | 0.916 | 3.304 |
| 115 | 87 | 63 | 1e91−1g1e | 1e91_2 | 0.880 | 4.067 |
| 115 | 87 | 63 | 1e91−1g1e | 1e91_3 | 0.892 | 4.626 |
| 115 | 87 | 63 | 1e91−1g1e | 1e91_4 | 0.843 | 4.616 |
| 115 | 87 | 63 | 1e91−1g1e | 1e91_5 | 0.843 | 5.165 |
| 115 | 87 | 63 | 1e91−1g1e | 1e91_6 | 0.892 | 5.378 |
| 115 | 87 | 63 | 1e91−1g1e | 1e91_7 | 0.855 | 4.757 |
| 115 | 87 | 63 | 1e91−1g1e | 1e91_8 | 0.867 | 5.513 |
| 115 | 87 | 63 | 1e91−1g1e | 1e91_9 | 0.855 | 5.559 |
| 115 | 87 | 63 | 1e91−1g1e | 1e91_10 | 0.867 | 4.844 |
| 115 | 87 | 63 | 1g1e−1e91 | 1g1e_1 | 0.928 | 3.200 |
| 115 | 87 | 63 | 1g1e−1e91 | 1g1e_2 | 0.819 | 3.107 |
| 115 | 87 | 63 | 1g1e−1e91 | 1g1e_3 | 0.916 | 3.363 |
| 115 | 87 | 63 | 1g1e−1e91 | 1g1e_4 | 0.916 | 3.029 |
| 115 | 87 | 63 | 1g1e−1e91 | 1g1e_5 | 0.976 | 3.442 |
| 115 | 87 | 63 | 1g1e−1e91 | 1g1e_6 | 0.867 | 2.941 |
| 115 | 87 | 63 | 1g1e−1e91 | 1g1e_7 | 0.867 | 2.963 |
| 115 | 87 | 63 | 1g1e−1e91 | 1g1e_8 | 0.867 | 4.117 |
| 115 | 87 | 63 | 1g1e−1e91 | 1g1e_9 | 0.867 | 3.971 |
| 115 | 87 | 63 | 1g1e−1e91 | 1g1e_10 | 0.807 | 4.121 |
| 116 | 140 | 63 | 1k5d−1rrp | 1k5d_1 | 0.985 | 2.719 |
| 116 | 140 | 63 | 1rrp−1k5d | 1rrp_1 | 1.000 | 2.528 |
| 116 | 140 | 63 | 1rrp−1k5d | 1rrp_2 | 0.896 | 2.663 |
| 119 | 71 | 63 | 1kil−1l4a | 1kil_1 | 0.000 | 2.887 |
| 120 | 56 | 63 | 1r1b−1fyj | 1r1b_1 | 0.056 | 2.818 |
| 120 | 56 | 63 | 1r1b−1fyj | 1r1b_2 | 0.093 | 2.907 |
| 120 | 56 | 63 | 1r1b−1fyj | 1r1b_3 | 0.093 | 3.565 |
| 120 | 56 | 63 | 1r1b−1fyj | 1r1b_4 | 0.093 | 3.670 |
| 120 | 56 | 63 | 1r1b−1fyj | 1r1b_5 | 0.093 | 4.534 |
| 120 | 56 | 63 | 1r1b−1fyj | 1r1b_6 | 0.537 | 5.112 |
| 120 | 56 | 63 | 1r1b−1fyj | 1r1b_7 | 0.481 | 5.190 |
| 120 | 56 | 63 | 1fyj−1r1b | 1fyj_1 | 1.000 | 2.016 |
| 120 | 56 | 63 | 1fyj−1r1b | 1fyj_2 | 0.093 | 3.104 |
| 120 | 56 | 63 | 1fyj−1r1b | 1fyj_3 | 1.000 | 2.528 |
| 120 | 56 | 63 | 1fyj−1r1b | 1fyj_4 | 0.648 | 3.906 |
| 120 | 56 | 63 | 1fyj−1r1b | 1fyj_5 | 0.352 | 4.039 |
| 120 | 56 | 63 | 1fyj−1r1b | 1fyj_6 | 0.463 | 3.646 |
| 120 | 56 | 63 | 1fyj−1r1b | 1fyj_7 | 0.093 | 3.651 |
| 120 | 56 | 63 | 1fyj−1r1b | 1fyj_8 | 0.259 | 4.254 |
| 120 | 56 | 63 | 1fyj−1r1b | 1fyj_9 | 0.685 | 4.282 |
| 120 | 56 | 63 | 1fyj−1r1b | 1fyj_10 | 0.944 | 4.560 |
| 122 | 361 | 62 | 1clq−1noz | 1clq_1 | 0.931 | 2.435 |
| 126 | 68 | 62 | 1qa6−1mms | 1qa6_1 | 0.909 | 1.857 |
| 126 | 68 | 62 | 1qa6−1mms | 1qa6_2 | 0.530 | 4.705 |
| 126 | 68 | 62 | 1qa6−1mms | 1qa6_3 | 0.470 | 4.842 |
| 126 | 68 | 62 | 1qa6−1mms | 1qa6_4 | 0.606 | 4.862 |
| 126 | 68 | 62 | 1qa6−1mms | 1qa6_5 | 0.470 | 5.257 |
| 126 | 68 | 62 | 1qa6−1mms | 1qa6_6 | 0.379 | 6.130 |
| 126 | 68 | 62 | 1qa6−1mms | 1qa6_7 | 0.394 | 5.951 |
| 126 | 68 | 62 | 1qa6−1mms | 1qa6_8 | 0.439 | 5.873 |
| 126 | 68 | 62 | 1qa6−1mms | 1qa6_9 | 0.379 | 6.124 |
| 126 | 68 | 62 | 1qa6−1mms | 1qa6_10 | 0.470 | 5.609 |
| 126 | 68 | 62 | 1mms−1qa6 | 1mms_1 | 0.955 | 2.268 |
| 126 | 68 | 62 | 1mms−1qa6 | 1mms_2 | 1.000 | 2.226 |
| 126 | 68 | 62 | 1mms−1qa6 | 1mms_3 | 1.000 | 2.672 |
| 127 | 133 | 62 | 1sei−1an7 | 1sei_1 | 0.992 | 2.817 |
| 127 | 133 | 62 | 1sei−1an7 | 1sei_2 | 0.992 | 3.247 |
| 127 | 133 | 62 | 1sei−1an7 | 1sei_3 | 1.000 | 3.040 |
| 127 | 133 | 62 | 1sei−1an7 | 1sei_4 | 1.000 | 3.595 |
| 127 | 133 | 62 | 1an7−1sei | 1an7_1 | 1.000 | 2.061 |
| 127 | 133 | 62 | 1an7−1sei | 1an7_2 | 1.000 | 2.332 |
| 127 | 133 | 62 | 1an7−1sei | 1an7_3 | 1.000 | 2.140 |
| 127 | 133 | 62 | 1an7−1sei | 1an7_4 | 1.000 | 2.657 |
| 127 | 133 | 62 | 1an7−1sei | 1an7_5 | 1.000 | 3.005 |
| 127 | 133 | 62 | 1an7−1sei | 1an7_6 | 0.969 | 3.234 |
| 127 | 133 | 62 | 1an7−1sei | 1an7_7 | 0.953 | 3.916 |
| 127 | 133 | 62 | 1an7−1sei | 1an7_8 | 0.867 | 3.971 |
| 127 | 133 | 62 | 1an7−1sei | 1an7_9 | 0.969 | 4.164 |
| 127 | 133 | 62 | 1an7−1sei | 1an7_10 | 0.914 | 3.753 |
| 129 | 68 | 62 | 1c4q−2bos | 1c4q_1 | 1.000 | 1.424 |
| 129 | 68 | 62 | 1c4q−2bos | 1c4q_2 | 1.000 | 1.223 |
| 129 | 68 | 62 | 1c4q−2bos | 1c4q_3 | 1.000 | 1.127 |
| 129 | 68 | 62 | 1c4q−2bos | 1c4q_4 | 1.000 | 1.049 |
| 129 | 68 | 62 | 1c4q−2bos | 1c4q_5 | 1.000 | 1.203 |
| 129 | 68 | 62 | 1c4q−2bos | 1c4q_6 | 1.000 | 1.176 |
| 129 | 68 | 62 | 1c4q−2bos | 1c4q_7 | 1.000 | 0.985 |
| 129 | 68 | 62 | 1c4q−2bos | 1c4q_8 | 1.000 | 1.040 |
| 129 | 68 | 62 | 1c4q−2bos | 1c4q_9 | 1.000 | 1.297 |
| 129 | 68 | 62 | 1c4q−2bos | 1c4q_10 | 1.000 | 1.101 |
| 129 | 68 | 62 | 2bos−1c4q | 2bos_1 | 1.000 | 0.867 |
| 129 | 68 | 62 | 2bos−1c4q | 2bos_2 | 1.000 | 0.967 |
| 129 | 68 | 62 | 2bos−1c4q | 2bos_3 | 1.000 | 1.031 |
| 129 | 68 | 62 | 2bos−1c4q | 2bos_4 | 1.000 | 1.186 |
| 129 | 68 | 62 | 2bos−1c4q | 2bos_5 | 1.000 | 0.927 |
| 131 | 196 | 61 | 1ezv−1bgy | 1ezv_1 | 0.974 | 2.312 |
| 134 | 635 | 61 | 1fce−1l1y | 1fce_1 | 0.989 | 1.715 |
| 134 | 635 | 61 | 1fce−1l1y | 1fce_2 | 0.989 | 1.804 |
| 134 | 635 | 61 | 1fce−1l1y | 1fce_3 | 0.976 | 2.001 |
| 134 | 635 | 61 | 1fce−1l1y | 1fce_4 | 0.977 | 2.153 |
| 137 | 128 | 61 | 6pax−1pdn | 6pax_1 | 0.479 | 9.346 |
| 142 | 123 | 60 | 1d9c−1fyh | 1d9c_1 | 1.000 | 2.878 |
| 142 | 123 | 60 | 1d9c−1fyh | 1d9c_2 | 0.983 | 3.091 |
| 142 | 123 | 60 | 1d9c−1fyh | 1d9c_3 | 0.967 | 3.181 |
| 142 | 123 | 60 | 1d9c−1fyh | 1d9c_4 | 0.917 | 3.198 |
| 142 | 123 | 60 | 1d9c−1fyh | 1d9c_5 | 0.933 | 3.360 |
| 142 | 123 | 60 | 1d9c−1fyh | 1d9c_6 | 0.933 | 3.508 |
| 142 | 123 | 60 | 1d9c−1fyh | 1d9c_7 | 0.942 | 3.678 |
| 142 | 123 | 60 | 1d9c−1fyh | 1d9c_8 | 0.892 | 4.011 |
| 142 | 123 | 60 | 1d9c−1fyh | 1d9c_9 | 0.842 | 4.155 |
| 142 | 123 | 60 | 1d9c−1fyh | 1d9c_10 | 0.767 | 4.170 |
| 143 | 271 | 60 | 1llq−1qr6 | 1llq_1 | 0.976 | 2.283 |
| 148 | 86 | 60 | 1a32−1ab3 | 1a32_1 | 0.694 | 4.877 |
| 148 | 86 | 60 | 1a32−1ab3 | 1a32_2 | 0.835 | 6.840 |
| 148 | 86 | 60 | 1a32−1ab3 | 1a32_3 | 0.824 | 8.385 |
| 148 | 86 | 60 | 1a32−1ab3 | 1a32_4 | 0.729 | 9.780 |
| 148 | 86 | 60 | 1ab3−1a32 | 1ab3_1 | 0.871 | 4.096 |
| 148 | 86 | 60 | 1ab3−1a32 | 1ab3_2 | 1.000 | 5.088 |
| 148 | 86 | 60 | 1ab3−1a32 | 1ab3_3 | 0.894 | 5.448 |
| 148 | 86 | 60 | 1ab3−1a32 | 1ab3_4 | 0.906 | 5.071 |
| 148 | 86 | 60 | 1ab3−1a32 | 1ab3_5 | 1.000 | 5.617 |
| 148 | 86 | 60 | 1ab3−1a32 | 1ab3_6 | 0.800 | 5.476 |
| 148 | 86 | 60 | 1ab3−1a32 | 1ab3_7 | 0.718 | 5.479 |
| 148 | 86 | 60 | 1ab3−1a32 | 1ab3_8 | 0.718 | 5.367 |
| 148 | 86 | 60 | 1ab3−1a32 | 1ab3_9 | 0.000 | 5.833 |
| 148 | 86 | 60 | 1ab3−1a32 | 1ab3_10 | 0.000 | 5.591 |
| 149 | 264 | 60 | 2aai−1abr | 2aai_1 | 1.000 | 1.508 |
| 149 | 264 | 60 | 2aai−1abr | 2aai_2 | 1.000 | 1.684 |
| 149 | 264 | 60 | 1abr−2aai | 1abr_1 | 0.977 | 2.086 |
| 149 | 264 | 60 | 1abr−2aai | 1abr_2 | 0.996 | 2.419 |
| 149 | 264 | 60 | 1abr−2aai | 1abr_3 | 1.000 | 2.348 |
| 149 | 264 | 60 | 1abr−2aai | 1abr_4 | 1.000 | 2.529 |
| 149 | 264 | 60 | 1abr−2aai | 1abr_5 | 0.954 | 2.570 |
| 149 | 264 | 60 | 1abr−2aai | 1abr_6 | 0.977 | 2.565 |
| 149 | 264 | 60 | 1abr−2aai | 1abr_7 | 0.958 | 2.977 |
| 149 | 264 | 60 | 1abr−2aai | 1abr_8 | 0.958 | 2.707 |
| 149 | 264 | 60 | 1abr−2aai | 1abr_9 | 0.981 | 3.189 |
| 149 | 264 | 60 | 1abr−2aai | 1abr_10 | 0.981 | 2.894 |
| 151 | 108 | 59 | 1lm0−1liz | 1lm0_1 | 0.920 | 2.578 |
| 151 | 108 | 59 | 1lm0−1liz | 1lm0_2 | 0.800 | 3.234 |
| 151 | 108 | 59 | 1lm0−1liz | 1lm0_3 | 0.800 | 3.233 |
| 151 | 108 | 59 | 1lm0−1liz | 1lm0_4 | 0.980 | 3.223 |
| 151 | 108 | 59 | 1lm0−1liz | 1lm0_5 | 0.960 | 3.660 |
| 151 | 108 | 59 | 1lm0−1liz | 1lm0_6 | 0.930 | 3.639 |
| 151 | 108 | 59 | 1lm0−1liz | 1lm0_7 | 0.960 | 3.569 |
| 151 | 108 | 59 | 1lm0−1liz | 1lm0_8 | 0.870 | 3.634 |
| 151 | 108 | 59 | 1lm0−1liz | 1lm0_9 | 0.920 | 3.770 |
| 151 | 108 | 59 | 1lm0−1liz | 1lm0_10 | 0.950 | 3.877 |
| 151 | 108 | 59 | 1liz−1lm0 | 1liz_1 | 0.970 | 4.053 |
| 151 | 108 | 59 | 1liz−1lm0 | 1liz_2 | 0.960 | 3.865 |
| 151 | 108 | 59 | 1liz−1lm0 | 1liz_3 | 0.960 | 4.306 |
| 151 | 108 | 59 | 1liz−1lm0 | 1liz_4 | 0.970 | 4.051 |
| 151 | 108 | 59 | 1liz−1lm0 | 1liz_5 | 0.970 | 4.038 |
| 151 | 108 | 59 | 1liz−1lm0 | 1liz_6 | 0.970 | 4.462 |
| 151 | 108 | 59 | 1liz−1lm0 | 1liz_7 | 0.970 | 4.309 |
| 151 | 108 | 59 | 1liz−1lm0 | 1liz_8 | 0.960 | 4.657 |
| 151 | 108 | 59 | 1liz−1lm0 | 1liz_9 | 0.990 | 4.833 |
| 151 | 108 | 59 | 1liz−1lm0 | 1liz_10 | 0.980 | 4.539 |
| 156 | 159 | 59 | 1bv1−1e09 | 1bv1_1 | 1.000 | 1.963 |
| 156 | 159 | 59 | 1bv1−1e09 | 1bv1_2 | 0.962 | 2.382 |
| 156 | 159 | 59 | 1bv1−1e09 | 1bv1_3 | 0.925 | 2.665 |
| 156 | 159 | 59 | 1bv1−1e09 | 1bv1_4 | 0.925 | 2.729 |
| 156 | 159 | 59 | 1bv1−1e09 | 1bv1_5 | 0.925 | 2.531 |
| 156 | 159 | 59 | 1bv1−1e09 | 1bv1_6 | 0.962 | 2.796 |
| 156 | 159 | 59 | 1bv1−1e09 | 1bv1_7 | 0.962 | 2.564 |
| 156 | 159 | 59 | 1bv1−1e09 | 1bv1_8 | 0.899 | 2.885 |
| 156 | 159 | 59 | 1bv1−1e09 | 1bv1_9 | 0.931 | 2.513 |
| 156 | 159 | 59 | 1bv1−1e09 | 1bv1_10 | 0.899 | 2.547 |
| 156 | 159 | 59 | 1e09−1bv1 | 1e09_1 | 0.969 | 2.078 |
| 156 | 159 | 59 | 1e09−1bv1 | 1e09_2 | 0.969 | 1.892 |
| 156 | 159 | 59 | 1e09−1bv1 | 1e09_3 | 0.969 | 2.298 |
| 156 | 159 | 59 | 1e09−1bv1 | 1e09_4 | 0.969 | 2.472 |
| 156 | 159 | 59 | 1e09−1bv1 | 1e09_5 | 0.969 | 1.979 |
| 156 | 159 | 59 | 1e09−1bv1 | 1e09_6 | 0.969 | 1.997 |
| 156 | 159 | 59 | 1e09−1bv1 | 1e09_7 | 0.969 | 2.071 |
| 156 | 159 | 59 | 1e09−1bv1 | 1e09_8 | 0.962 | 2.677 |

n: internal numbering; length of alignment; % sequence%ID (cf. Homstrad); refA−refB: reference alignment; altA: alternative snapshot for A; SP: sum−of−pairs score of alt. alignment w/r reference

Supplementary Material Table S2b: Details of aligned Simulation Snapshots −− The Meaning of Alignment (Pirovano, Feenstra & Heringa)

| n | length | %ID | refA-refB | altA | SP | RMSD |
|---|---|---|---|---|---|---|
| 156 | 159 | 59 | 1e09-1bv1 | 1e09_9 | 0.918 | 2.910 |
| 156 | 159 | 59 | 1e09-1bv1 | 1e09_10 | 0.925 | 2.819 |
| 157 | 298 | 59 | 1nfk-1a3q | 1nfk_1 | 0.954 | 3.192 |
| 159 | 319 | 59 | 1onr-1f05 | 1onr_1 | 0.965 | 2.441 |
| 159 | 319 | 59 | 1onr-1f05 | 1onr_2 | 1.000 | 2.631 |
| 159 | 319 | 59 | 1onr-1f05 | 1onr_3 | 0.934 | 3.079 |
| 159 | 319 | 59 | 1onr-1f05 | 1onr_4 | 0.965 | 3.204 |
| 159 | 319 | 59 | 1f05-1onr | 1f05_1 | 0.962 | 2.135 |
| 159 | 319 | 59 | 1f05-1onr | 1f05_2 | 0.927 | 2.291 |
| 159 | 319 | 59 | 1f05-1onr | 1f05_3 | 0.918 | 2.708 |
| 159 | 319 | 59 | 1f05-1onr | 1f05_4 | 0.934 | 2.941 |
| 160 | 450 | 58 | 1gc5-1l2l | 1gc5_1 | 0.988 | 2.723 |
| 160 | 450 | 58 | 1gc5-1l2l | 1gc5_2 | 0.979 | 2.790 |
| 160 | 450 | 58 | 1gc5-1l2l | 1gc5_3 | 0.977 | 2.757 |
| 160 | 450 | 58 | 1gc5-1l2l | 1gc5_4 | 0.979 | 2.812 |
| 160 | 450 | 58 | 1gc5-1l2l | 1gc5_5 | 0.991 | 2.526 |
| 160 | 450 | 58 | 1gc5-1l2l | 1gc5_6 | 0.991 | 2.786 |
| 160 | 450 | 58 | 1gc5-1l2l | 1gc5_7 | 0.965 | 3.087 |
| 160 | 450 | 58 | 1gc5-1l2l | 1gc5_8 | 0.977 | 2.890 |
| 160 | 450 | 58 | 1gc5-1l2l | 1gc5_9 | 0.979 | 3.071 |
| 160 | 450 | 58 | 1gc5-1l2l | 1gc5_10 | 0.967 | 2.925 |
| 163 | 273 | 58 | 1dea-1ne7 | 1dea_1 | 0.996 | 2.337 |
| 163 | 273 | 58 | 1dea-1ne7 | 1dea_2 | 0.996 | 3.161 |
| 163 | 273 | 58 | 1dea-1ne7 | 1dea_3 | 0.932 | 3.419 |
| 165 | 116 | 58 | 1eo6-1gnu | 1eo6_1 | 0.991 | 1.431 |
| 165 | 116 | 58 | 1eo6-1gnu | 1eo6_2 | 1.000 | 1.723 |
| 165 | 116 | 58 | 1eo6-1gnu | 1eo6_3 | 1.000 | 2.267 |
| 165 | 116 | 58 | 1eo6-1gnu | 1eo6_4 | 1.000 | 2.161 |
| 165 | 116 | 58 | 1eo6-1gnu | 1eo6_5 | 1.000 | 2.343 |
| 165 | 116 | 58 | 1eo6-1gnu | 1eo6_6 | 0.879 | 3.158 |
| 165 | 116 | 58 | 1eo6-1gnu | 1eo6_7 | 0.862 | 3.187 |
| 165 | 116 | 58 | 1eo6-1gnu | 1eo6_8 | 0.871 | 3.422 |
| 165 | 116 | 58 | 1eo6-1gnu | 1eo6_9 | 0.871 | 3.309 |
| 165 | 116 | 58 | 1eo6-1gnu | 1eo6_10 | 0.845 | 3.752 |
| 165 | 116 | 58 | 1gnu-1eo6 | 1gnu_1 | 1.000 | 1.562 |
| 165 | 116 | 58 | 1gnu-1eo6 | 1gnu_2 | 1.000 | 2.039 |
| 165 | 116 | 58 | 1gnu-1eo6 | 1gnu_3 | 1.000 | 1.845 |
| 165 | 116 | 58 | 1gnu-1eo6 | 1gnu_4 | 1.000 | 2.394 |
| 165 | 116 | 58 | 1gnu-1eo6 | 1gnu_5 | 1.000 | 2.171 |
| 165 | 116 | 58 | 1gnu-1eo6 | 1gnu_6 | 1.000 | 1.888 |
| 165 | 116 | 58 | 1gnu-1eo6 | 1gnu_7 | 1.000 | 2.108 |
| 165 | 116 | 58 | 1gnu-1eo6 | 1gnu_8 | 1.000 | 2.203 |
| 165 | 116 | 58 | 1gnu-1eo6 | 1gnu_9 | 1.000 | 2.252 |
| 165 | 116 | 58 | 1gnu-1eo6 | 1gnu_10 | 1.000 | 2.060 |
| 166 | 76 | 58 | 1fjg-1pkp | 1fjg_1 | 1.000 | 2.119 |
| 166 | 76 | 58 | 1pkp-1fjg | 1pkp_1 | 1.000 | 1.187 |
| 166 | 76 | 58 | 1pkp-1fjg | 1pkp_2 | 1.000 | 2.397 |
| 166 | 76 | 58 | 1pkp-1fjg | 1pkp_3 | 1.000 | 2.573 |
| 166 | 76 | 58 | 1pkp-1fjg | 1pkp_4 | 1.000 | 2.262 |
| 166 | 76 | 58 | 1pkp-1fjg | 1pkp_5 | 1.000 | 2.190 |
| 166 | 76 | 58 | 1pkp-1fjg | 1pkp_6 | 1.000 | 2.439 |
| 166 | 76 | 58 | 1pkp-1fjg | 1pkp_7 | 1.000 | 2.291 |
| 166 | 76 | 58 | 1pkp-1fjg | 1pkp_8 | 1.000 | 2.219 |
| 166 | 76 | 58 | 1pkp-1fjg | 1pkp_9 | 0.930 | 2.303 |
| 166 | 76 | 58 | 1pkp-1fjg | 1pkp_10 | 0.915 | 2.439 |
| 170 | 287 | 57 | 1kph-1kpi | 1kph_1 | 0.954 | 1.528 |
| 176 | 621 | 57 | 1jjy-1jqk | 1jjy_1 | 0.967 | 2.291 |
| 176 | 621 | 57 | 1jjy-1jqk | 1jjy_2 | 0.909 | 2.242 |
| 176 | 621 | 57 | 1jjy-1jqk | 1jjy_3 | 0.941 | 2.422 |
| 177 | 885 | 57 | 1kbl-1h6z | 1kbl_1 | 0.965 | 2.649 |
| 179 | 469 | 57 | 1kap-1sat | 1kap_1 | 0.947 | 2.927 |
| 179 | 469 | 57 | 1kap-1sat | 1kap_2 | 0.927 | 3.490 |
| 180 | 313 | 57 | 1qtr-1azw | 1qtr_1 | 0.990 | 2.592 |
| 180 | 313 | 57 | 1qtr-1azw | 1qtr_2 | 0.990 | 2.311 |
| 180 | 313 | 57 | 1qtr-1azw | 1qtr_3 | 0.990 | 2.878 |
| 180 | 313 | 57 | 1qtr-1azw | 1qtr_4 | 0.974 | 2.980 |
| 180 | 313 | 57 | 1qtr-1azw | 1qtr_5 | 0.965 | 3.096 |
| 180 | 313 | 57 | 1qtr-1azw | 1qtr_6 | 0.987 | 2.950 |
| 180 | 313 | 57 | 1qtr-1azw | 1qtr_7 | 0.971 | 2.964 |
| 180 | 313 | 57 | 1qtr-1azw | 1qtr_8 | 0.987 | 3.127 |
| 180 | 313 | 57 | 1qtr-1azw | 1qtr_9 | 0.987 | 3.142 |
| 180 | 313 | 57 | 1qtr-1azw | 1qtr_10 | 0.965 | 3.139 |
| 180 | 313 | 57 | 1azw-1qtr | 1azw_1 | 1.000 | 1.623 |
| 180 | 313 | 57 | 1azw-1qtr | 1azw_2 | 1.000 | 1.918 |
| 180 | 313 | 57 | 1azw-1qtr | 1azw_3 | 0.997 | 1.894 |
| 180 | 313 | 57 | 1azw-1qtr | 1azw_4 | 0.978 | 2.683 |
| 180 | 313 | 57 | 1azw-1qtr | 1azw_5 | 0.984 | 2.796 |
| 180 | 313 | 57 | 1azw-1qtr | 1azw_6 | 0.984 | 2.672 |
| 183 | 159 | 56 | 1q0n-1cbk | 1q0n_1 | 0.949 | 2.523 |
| 183 | 159 | 56 | 1q0n-1cbk | 1q0n_2 | 0.949 | 2.362 |
| 183 | 159 | 56 | 1q0n-1cbk | 1q0n_3 | 0.917 | 2.858 |
| 183 | 159 | 56 | 1q0n-1cbk | 1q0n_4 | 0.917 | 3.219 |
| 183 | 159 | 56 | 1q0n-1cbk | 1q0n_5 | 0.949 | 3.010 |
| 183 | 159 | 56 | 1q0n-1cbk | 1q0n_6 | 0.949 | 2.788 |
| 183 | 159 | 56 | 1q0n-1cbk | 1q0n_7 | 0.949 | 2.917 |
| 183 | 159 | 56 | 1q0n-1cbk | 1q0n_8 | 0.962 | 3.036 |
| 183 | 159 | 56 | 1q0n-1cbk | 1q0n_9 | 0.962 | 2.689 |
| 183 | 159 | 56 | 1q0n-1cbk | 1q0n_10 | 0.968 | 2.787 |
| 183 | 159 | 56 | 1cbk-1q0n | 1cbk_1 | 0.936 | 1.448 |
| 183 | 159 | 56 | 1cbk-1q0n | 1cbk_2 | 0.968 | 1.659 |
| 183 | 159 | 56 | 1cbk-1q0n | 1cbk_3 | 0.936 | 1.748 |
| 183 | 159 | 56 | 1cbk-1q0n | 1cbk_4 | 0.936 | 1.858 |
| 183 | 159 | 56 | 1cbk-1q0n | 1cbk_5 | 0.968 | 1.717 |
| 183 | 159 | 56 | 1cbk-1q0n | 1cbk_6 | 0.936 | 1.822 |
| 183 | 159 | 56 | 1cbk-1q0n | 1cbk_7 | 1.000 | 2.020 |
| 183 | 159 | 56 | 1cbk-1q0n | 1cbk_8 | 0.968 | 2.123 |
| 183 | 159 | 56 | 1cbk-1q0n | 1cbk_9 | 1.000 | 2.061 |
| 183 | 159 | 56 | 1cbk-1q0n | 1cbk_10 | 1.000 | 2.037 |
| 184 | 541 | 56 | 1g8f-1i2d | 1g8f_1 | 0.955 | 5.181 |
| 189 | 107 | 56 | 2irf-1if1 | 2irf_1 | 0.854 | 2.864 |
| 194 | 73 | 56 | 1ccd-1utg | 1ccd_1 | 1.000 | 5.043 |
| 194 | 73 | 56 | 1ccd-1utg | 1ccd_2 | 0.700 | 5.653 |
| 194 | 73 | 56 | 1ccd-1utg | 1ccd_3 | 0.957 | 7.046 |
| 194 | 73 | 56 | 1ccd-1utg | 1ccd_4 | 0.557 | 7.137 |
| 194 | 73 | 56 | 1ccd-1utg | 1ccd_5 | 0.329 | 7.774 |
| 194 | 73 | 56 | 1ccd-1utg | 1ccd_6 | 0.300 | 8.382 |
| 194 | 73 | 56 | 1ccd-1utg | 1ccd_7 | 0.371 | 8.105 |
| 194 | 73 | 56 | 1ccd-1utg | 1ccd_8 | 0.343 | 8.196 |
| 194 | 73 | 56 | 1ccd-1utg | 1ccd_9 | 0.329 | 8.365 |
| 194 | 73 | 56 | 1utg-1ccd | 1utg_1 | 0.914 | 3.563 |
| 194 | 73 | 56 | 1utg-1ccd | 1utg_2 | 0.914 | 4.857 |
| 194 | 73 | 56 | 1utg-1ccd | 1utg_3 | 0.900 | 5.164 |
| 194 | 73 | 56 | 1utg-1ccd | 1utg_4 | 0.686 | 5.885 |
| 194 | 73 | 56 | 1utg-1ccd | 1utg_5 | 0.686 | 5.847 |
| 194 | 73 | 56 | 1utg-1ccd | 1utg_6 | 0.886 | 6.396 |
| 194 | 73 | 56 | 1utg-1ccd | 1utg_7 | 0.386 | 6.145 |
| 194 | 73 | 56 | 1utg-1ccd | 1utg_8 | 0.557 | 6.524 |
| 194 | 73 | 56 | 1utg-1ccd | 1utg_9 | 0.686 | 6.503 |
| 194 | 73 | 56 | 1utg-1ccd | 1utg_10 | 0.500 | 6.402 |
| 195 | 213 | 56 | 1b43-1a77 | 1b43_1 | 0.963 | 2.520 |
| 195 | 213 | 56 | 1b43-1a77 | 1b43_2 | 0.952 | 3.043 |
| 195 | 213 | 56 | 1b43-1a77 | 1b43_3 | 0.957 | 3.900 |
| 195 | 213 | 56 | 1b43-1a77 | 1b43_4 | 0.915 | 3.695 |
| 195 | 213 | 56 | 1b43-1a77 | 1b43_5 | 0.910 | 4.047 |
| 195 | 213 | 56 | 1b43-1a77 | 1b43_6 | 0.856 | 4.301 |
| 195 | 213 | 56 | 1b43-1a77 | 1b43_7 | 0.856 | 4.452 |
| 195 | 213 | 56 | 1a77-1b43 | 1a77_1 | 0.883 | 3.788 |
| 195 | 213 | 56 | 1a77-1b43 | 1a77_2 | 0.883 | 5.095 |
| 195 | 213 | 56 | 1a77-1b43 | 1a77_3 | 0.819 | 5.147 |
| 195 | 213 | 56 | 1a77-1b43 | 1a77_4 | 0.862 | 5.452 |
| 195 | 213 | 56 | 1a77-1b43 | 1a77_5 | 0.851 | 5.063 |
| 195 | 213 | 56 | 1a77-1b43 | 1a77_6 | 0.830 | 4.525 |
| 195 | 213 | 56 | 1a77-1b43 | 1a77_7 | 0.830 | 4.488 |
| 195 | 213 | 56 | 1a77-1b43 | 1a77_8 | 0.851 | 5.109 |
| 195 | 213 | 56 | 1a77-1b43 | 1a77_9 | 0.830 | 5.135 |
| 195 | 213 | 56 | 1a77-1b43 | 1a77_10 | 0.851 | 5.041 |
| 206 | 98 | 54 | 1aps-2acy | 1aps_1 | 0.949 | 2.574 |
| 206 | 98 | 54 | 1aps-2acy | 1aps_2 | 1.000 | 3.112 |
| 206 | 98 | 54 | 1aps-2acy | 1aps_3 | 1.000 | 3.394 |
| 206 | 98 | 54 | 1aps-2acy | 1aps_4 | 0.939 | 3.669 |
| 206 | 98 | 54 | 1aps-2acy | 1aps_5 | 0.949 | 3.280 |
| 206 | 98 | 54 | 1aps-2acy | 1aps_6 | 0.929 | 3.536 |
| 206 | 98 | 54 | 1aps-2acy | 1aps_7 | 0.949 | 3.389 |
| 206 | 98 | 54 | 1aps-2acy | 1aps_8 | 0.949 | 3.480 |
| 206 | 98 | 54 | 1aps-2acy | 1aps_9 | 0.949 | 3.708 |
| 206 | 98 | 54 | 1aps-2acy | 1aps_10 | 0.939 | 3.751 |
| 206 | 98 | 54 | 2acy-1aps | 2acy_1 | 1.000 | 1.147 |
| 206 | 98 | 54 | 2acy-1aps | 2acy_2 | 1.000 | 1.617 |
| 206 | 98 | 54 | 2acy-1aps | 2acy_3 | 1.000 | 1.630 |
| 206 | 98 | 54 | 2acy-1aps | 2acy_4 | 0.949 | 1.770 |
| 206 | 98 | 54 | 2acy-1aps | 2acy_5 | 1.000 | 1.998 |
| 206 | 98 | 54 | 2acy-1aps | 2acy_6 | 1.000 | 2.610 |
| 206 | 98 | 54 | 2acy-1aps | 2acy_7 | 1.000 | 3.348 |
| 206 | 98 | 54 | 2acy-1aps | 2acy_8 | 0.898 | 3.670 |
| 206 | 98 | 54 | 2acy-1aps | 2acy_9 | 0.847 | 3.500 |
| 206 | 98 | 54 | 2acy-1aps | 2acy_10 | 0.857 | 3.565 |
| 209 | 50 | 54 | 1wjd-1e0e | 1wjd_1 | 0.913 | 6.515 |
| 209 | 50 | 54 | 1wjd-1e0e | 1wjd_2 | 0.652 | 8.706 |
| 209 | 50 | 54 | 1wjd-1e0e | 1wjd_3 | 0.739 | 8.207 |
| 209 | 50 | 54 | 1wjd-1e0e | 1wjd_4 | 0.804 | 8.907 |
| 209 | 50 | 54 | 1wjd-1e0e | 1wjd_6 | 0.674 | 8.979 |
| 209 | 50 | 54 | 1wjd-1e0e | 1wjd_9 | 0.652 | 9.842 |
| 209 | 50 | 54 | 1wjd-1e0e | 1wjd_10 | 0.652 | 9.815 |
| 209 | 50 | 54 | 1e0e-1wjd | 1e0e_1 | 1.000 | 1.901 |
| 209 | 50 | 54 | 1e0e-1wjd | 1e0e_2 | 1.000 | 3.004 |
| 209 | 50 | 54 | 1e0e-1wjd | 1e0e_3 | 0.761 | 3.085 |
| 209 | 50 | 54 | 1e0e-1wjd | 1e0e_4 | 0.761 | 2.627 |
| 209 | 50 | 54 | 1e0e-1wjd | 1e0e_5 | 0.761 | 3.414 |
| 209 | 50 | 54 | 1e0e-1wjd | 1e0e_6 | 0.870 | 3.344 |
| 209 | 50 | 54 | 1e0e-1wjd | 1e0e_7 | 0.761 | 3.631 |
| 209 | 50 | 54 | 1e0e-1wjd | 1e0e_8 | 0.870 | 3.637 |
| 209 | 50 | 54 | 1e0e-1wjd | 1e0e_9 | 0.891 | 3.426 |
| 209 | 50 | 54 | 1e0e-1wjd | 1e0e_10 | 0.717 | 3.158 |
| 214 | 216 | 54 | 1efy-1gs0 | 1efy_1 | 0.991 | 1.633 |
| 214 | 216 | 54 | 1efy-1gs0 | 1efy_2 | 0.930 | 1.712 |
| 214 | 216 | 54 | 1efy-1gs0 | 1efy_3 | 0.986 | 1.625 |
| 214 | 216 | 54 | 1efy-1gs0 | 1efy_4 | 0.986 | 1.604 |
| 214 | 216 | 54 | 1efy-1gs0 | 1efy_5 | 0.986 | 1.694 |
| 214 | 216 | 54 | 1efy-1gs0 | 1efy_6 | 0.991 | 1.740 |
| 214 | 216 | 54 | 1efy-1gs0 | 1efy_7 | 1.000 | 1.583 |
| 214 | 216 | 54 | 1efy-1gs0 | 1efy_8 | 0.995 | 2.361 |
| 214 | 216 | 54 | 1efy-1gs0 | 1efy_9 | 0.991 | 2.274 |
| 214 | 216 | 54 | 1efy-1gs0 | 1efy_10 | 0.991 | 2.200 |
| 215 | 61 | 54 | 1g9l-1i2t | 1g9l_1 | 1.000 | 3.327 |
| 215 | 61 | 54 | 1g9l-1i2t | 1g9l_2 | 1.000 | 2.817 |
| 215 | 61 | 54 | 1g9l-1i2t | 1g9l_3 | 1.000 | 2.739 |
| 215 | 61 | 54 | 1g9l-1i2t | 1g9l_4 | 1.000 | 2.952 |
| 215 | 61 | 54 | 1g9l-1i2t | 1g9l_5 | 1.000 | 2.792 |
| 215 | 61 | 54 | 1g9l-1i2t | 1g9l_6 | 1.000 | 2.803 |
| 215 | 61 | 54 | 1g9l-1i2t | 1g9l_7 | 1.000 | 3.182 |
| 215 | 61 | 54 | 1g9l-1i2t | 1g9l_8 | 0.918 | 2.965 |
| 215 | 61 | 54 | 1g9l-1i2t | 1g9l_9 | 1.000 | 2.844 |
| 215 | 61 | 54 | 1g9l-1i2t | 1g9l_10 | 1.000 | 2.954 |
| 215 | 61 | 54 | 1i2t-1g9l | 1i2t_1 | 1.000 | 0.950 |
| 215 | 61 | 54 | 1i2t-1g9l | 1i2t_2 | 0.754 | 4.237 |
| 215 | 61 | 54 | 1i2t-1g9l | 1i2t_3 | 1.000 | 5.557 |
| 215 | 61 | 54 | 1i2t-1g9l | 1i2t_4 | 0.459 | 7.821 |
| 215 | 61 | 54 | 1i2t-1g9l | 1i2t_5 | 0.459 | 8.774 |
| 215 | 61 | 54 | 1i2t-1g9l | 1i2t_6 | 0.721 | 9.177 |
| 215 | 61 | 54 | 1i2t-1g9l | 1i2t_9 | 0.787 | 8.977 |
| 215 | 61 | 54 | 1i2t-1g9l | 1i2t_10 | 0.508 | 8.335 |
| 220 | 218 | 53 | 1ois-1a35 | 1ois_3 | 0.883 | 4.356 |
| 220 | 218 | 53 | 1ois-1a35 | 1ois_5 | 0.911 | 5.547 |
| 220 | 218 | 53 | 1ois-1a35 | 1ois_7 | 0.860 | 5.978 |
| 220 | 218 | 53 | 1ois-1a35 | 1ois_8 | 0.836 | 6.613 |
| 220 | 218 | 53 | 1ois-1a35 | 1ois_9 | 0.930 | 6.089 |
| 220 | 218 | 53 | 1ois-1a35 | 1ois_10 | 0.925 | 5.814 |
| 221 | 114 | 53 | 1ed1-1hiw | 1ed1_1 | 0.956 | 2.168 |
| 221 | 114 | 53 | 1ed1-1hiw | 1ed1_2 | 0.770 | 2.729 |
| 221 | 114 | 53 | 1ed1-1hiw | 1ed1_3 | 0.770 | 2.808 |
| 221 | 114 | 53 | 1ed1-1hiw | 1ed1_4 | 0.788 | 2.358 |
| 221 | 114 | 53 | 1ed1-1hiw | 1ed1_5 | 0.912 | 2.288 |
| 221 | 114 | 53 | 1ed1-1hiw | 1ed1_6 | 0.947 | 2.230 |

n: internal numbering; length of alignment; % sequence%ID (cf. Homstrad); refA−refB: reference alignment; altA: alternative snapshot for A; SP: sum−of−pairs score of alt. alignment w/r reference

| n | length | %ID | refA−refB | altA | SP | RMSD |
|---|---|---|---|---|---|---|
| 221 | 114 | 53 | 1ed1−1hiw | 1ed1_7 | 0.841 | 2.428 |
| 221 | 114 | 53 | 1ed1−1hiw | 1ed1_8 | 0.841 | 2.480 |
| 221 | 114 | 53 | 1ed1−1hiw | 1ed1_9 | 0.805 | 2.469 |
| 221 | 114 | 53 | 1ed1−1hiw | 1ed1_10 | 0.850 | 2.989 |
| 225 | 554 | 53 | 1mvm−4dpv | 1mvm_1 | 0.953 | 3.635 |
| 233 | 59 | 52 | 1prb−1gjt | 1prb_1 | 0.712 | 4.409 |
| 233 | 59 | 52 | 1prb−1gjt | 1prb_2 | 0.712 | 4.501 |
| 233 | 59 | 52 | 1prb−1gjt | 1prb_3 | 0.712 | 4.838 |
| 233 | 59 | 52 | 1prb−1gjt | 1prb_4 | 0.712 | 4.753 |
| 233 | 59 | 52 | 1prb−1gjt | 1prb_5 | 0.712 | 4.028 |
| 233 | 59 | 52 | 1prb−1gjt | 1prb_6 | 0.712 | 3.932 |
| 233 | 59 | 52 | 1prb−1gjt | 1prb_7 | 0.712 | 5.702 |
| 233 | 59 | 52 | 1prb−1gjt | 1prb_8 | 0.731 | 5.680 |
| 233 | 59 | 52 | 1prb−1gjt | 1prb_9 | 0.712 | 5.216 |
| 233 | 59 | 52 | 1prb−1gjt | 1prb_10 | 0.346 | 6.071 |
| 233 | 59 | 52 | 1gjt−1prb | 1gjt_1 | 0.827 | 8.880 |
| 233 | 59 | 52 | 1gjt−1prb | 1gjt_2 | 0.827 | 8.613 |
| 233 | 59 | 52 | 1gjt−1prb | 1gjt_3 | 0.827 | 9.038 |
| 233 | 59 | 52 | 1gjt−1prb | 1gjt_4 | 0.827 | 8.648 |
| 233 | 59 | 52 | 1gjt−1prb | 1gjt_5 | 0.827 | 9.045 |
| 233 | 59 | 52 | 1gjt−1prb | 1gjt_6 | 0.827 | 9.304 |
| 233 | 59 | 52 | 1gjt−1prb | 1gjt_7 | 0.827 | 9.044 |
| 233 | 59 | 52 | 1gjt−1prb | 1gjt_8 | 0.827 | 9.109 |
| 233 | 59 | 52 | 1gjt−1prb | 1gjt_9 | 0.827 | 8.724 |
| 233 | 59 | 52 | 1gjt−1prb | 1gjt_10 | 0.808 | 8.405 |
| 237 | 306 | 52 | 1aq0−1ghs | 1aq0_1 | 0.997 | 1.385 |
| 237 | 306 | 52 | 1aq0−1ghs | 1aq0_2 | 0.997 | 1.412 |
| 237 | 306 | 52 | 1aq0−1ghs | 1aq0_3 | 0.993 | 1.890 |
| 237 | 306 | 52 | 1aq0−1ghs | 1aq0_4 | 0.997 | 1.629 |
| 237 | 306 | 52 | 1aq0−1ghs | 1aq0_5 | 0.997 | 1.671 |
| 237 | 306 | 52 | 1aq0−1ghs | 1aq0_6 | 0.997 | 1.590 |
| 237 | 306 | 52 | 1aq0−1ghs | 1aq0_7 | 0.997 | 1.456 |
| 237 | 306 | 52 | 1aq0−1ghs | 1aq0_8 | 0.997 | 1.573 |
| 237 | 306 | 52 | 1ghs−1aq0 | 1ghs_1 | 0.997 | 1.432 |
| 237 | 306 | 52 | 1ghs−1aq0 | 1ghs_2 | 0.993 | 1.739 |
| 237 | 306 | 52 | 1ghs−1aq0 | 1ghs_3 | 0.997 | 2.010 |
| 237 | 306 | 52 | 1ghs−1aq0 | 1ghs_4 | 1.000 | 1.963 |
| 243 | 205 | 52 | 1b09−1sac | 1b09_1 | 0.995 | 1.651 |
| 243 | 205 | 52 | 1b09−1sac | 1b09_2 | 0.995 | 1.660 |
| 243 | 205 | 52 | 1b09−1sac | 1b09_3 | 0.995 | 1.804 |
| 243 | 205 | 52 | 1sac−1b09 | 1sac_1 | 1.000 | 1.361 |
| 243 | 205 | 52 | 1sac−1b09 | 1sac_2 | 1.000 | 1.259 |
| 247 | 184 | 52 | 1xbr−1h6f | 1xbr_1 | 0.922 | 3.534 |
| 247 | 184 | 52 | 1h6f−1xbr | 1h6f_1 | 0.922 | 3.151 |
| 247 | 184 | 52 | 1h6f−1xbr | 1h6f_2 | 0.933 | 3.634 |
| 247 | 184 | 52 | 1h6f−1xbr | 1h6f_3 | 0.939 | 4.486 |
| 253 | 166 | 50 | 1laj−1f15 | 1laj_1 | 0.955 | 4.094 |
| 253 | 166 | 50 | 1laj−1f15 | 1laj_2 | 0.942 | 4.867 |
| 253 | 166 | 50 | 1f15−1laj | 1f15_1 | 0.955 | 2.991 |
| 262 | 240 | 50 | 2erc−1yub | 2erc_1 | 0.917 | 4.825 |
| 262 | 240 | 50 | 2erc−1yub | 2erc_2 | 0.870 | 5.907 |
| 262 | 240 | 50 | 2erc−1yub | 2erc_3 | 0.830 | 6.091 |
| 262 | 240 | 50 | 1yub−2erc | 1yub_1 | 0.817 | 3.615 |
| 262 | 240 | 50 | 1yub−2erc | 1yub_2 | 0.635 | 5.011 |
| 262 | 240 | 50 | 1yub−2erc | 1yub_3 | 0.691 | 5.903 |
| 268 | 183 | 49 | 1ep0−1dzr | 1ep0_1 | 0.967 | 2.089 |
| 268 | 183 | 49 | 1ep0−1dzr | 1ep0_2 | 0.961 | 2.567 |
| 268 | 183 | 49 | 1ep0−1dzr | 1ep0_3 | 0.939 | 2.836 |
| 268 | 183 | 49 | 1ep0−1dzr | 1ep0_4 | 0.961 | 2.566 |
| 268 | 183 | 49 | 1ep0−1dzr | 1ep0_5 | 0.961 | 2.712 |
| 268 | 183 | 49 | 1ep0−1dzr | 1ep0_6 | 0.961 | 2.781 |
| 268 | 183 | 49 | 1ep0−1dzr | 1ep0_7 | 0.934 | 3.403 |
| 276 | 115 | 49 | 1a6f−1d6t | 1a6f_1 | 0.646 | 4.236 |
| 276 | 115 | 49 | 1a6f−1d6t | 1a6f_2 | 0.628 | 4.965 |
| 276 | 115 | 49 | 1a6f−1d6t | 1a6f_3 | 0.558 | 5.818 |
| 276 | 115 | 49 | 1a6f−1d6t | 1a6f_4 | 0.619 | 6.586 |
| 276 | 115 | 49 | 1a6f−1d6t | 1a6f_5 | 0.619 | 6.401 |
| 276 | 115 | 49 | 1a6f−1d6t | 1a6f_6 | 0.575 | 6.753 |
| 276 | 115 | 49 | 1a6f−1d6t | 1a6f_7 | 0.522 | 7.712 |
| 276 | 115 | 49 | 1a6f−1d6t | 1a6f_8 | 0.646 | 7.693 |
| 276 | 115 | 49 | 1a6f−1d6t | 1a6f_9 | 0.637 | 7.643 |

| n | length | %ID | refA−refB | altA | SP | RMSD |
|---|---|---|---|---|---|---|
| 276 | 115 | 49 | 1a6f−1d6t | 1a6f_10 | 0.575 | 8.776 |
| 278 | 127 | 48 | 1gr3−1c28 | 1gr3_1 | 1.000 | 1.347 |
| 278 | 127 | 48 | 1gr3−1c28 | 1gr3_2 | 1.000 | 1.435 |
| 278 | 127 | 48 | 1gr3−1c28 | 1gr3_3 | 1.000 | 1.713 |
| 278 | 127 | 48 | 1gr3−1c28 | 1gr3_4 | 1.000 | 1.915 |
| 278 | 127 | 48 | 1gr3−1c28 | 1gr3_5 | 0.951 | 1.899 |
| 278 | 127 | 48 | 1gr3−1c28 | 1gr3_6 | 0.992 | 1.868 |
| 278 | 127 | 48 | 1gr3−1c28 | 1gr3_7 | 0.992 | 1.890 |
| 278 | 127 | 48 | 1gr3−1c28 | 1gr3_8 | 1.000 | 2.161 |
| 278 | 127 | 48 | 1gr3−1c28 | 1gr3_9 | 1.000 | 1.962 |
| 278 | 127 | 48 | 1gr3−1c28 | 1gr3_10 | 1.000 | 1.845 |
| 279 | 387 | 48 | 1kij−1ei1 | 1kij_1 | 0.982 | 1.803 |
| 279 | 387 | 48 | 1kij−1ei1 | 1kij_2 | 0.968 | 1.867 |
| 279 | 387 | 48 | 1ei1−1kij | 1ei1_1 | 0.995 | 1.937 |
| 279 | 387 | 48 | 1ei1−1kij | 1ei1_2 | 0.976 | 2.451 |
| 279 | 387 | 48 | 1ei1−1kij | 1ei1_3 | 0.976 | 2.828 |
| 279 | 387 | 48 | 1ei1−1kij | 1ei1_4 | 0.995 | 2.500 |
| 279 | 387 | 48 | 1ei1−1kij | 1ei1_5 | 0.974 | 2.720 |
| 282 | 365 | 48 | 1jq5−1kq3 | 1jq5_1 | 0.934 | 1.982 |
| 282 | 365 | 48 | 1jq5−1kq3 | 1jq5_2 | 0.970 | 1.818 |
| 282 | 365 | 48 | 1jq5−1kq3 | 1jq5_3 | 0.912 | 2.264 |
| 282 | 365 | 48 | 1jq5−1kq3 | 1jq5_4 | 0.909 | 2.455 |
| 282 | 365 | 48 | 1jq5−1kq3 | 1jq5_5 | 0.884 | 2.618 |
| 282 | 365 | 48 | 1jq5−1kq3 | 1jq5_6 | 0.870 | 2.688 |
| 282 | 365 | 48 | 1jq5−1kq3 | 1jq5_7 | 0.862 | 2.775 |
| 282 | 365 | 48 | 1jq5−1kq3 | 1jq5_8 | 0.903 | 2.725 |
| 282 | 365 | 48 | 1jq5−1kq3 | 1jq5_9 | 0.934 | 2.703 |
| 282 | 365 | 48 | 1jq5−1kq3 | 1jq5_10 | 0.890 | 2.992 |
| 282 | 365 | 48 | 1kq3−1jq5 | 1kq3_1 | 0.959 | 2.035 |
| 282 | 365 | 48 | 1kq3−1jq5 | 1kq3_2 | 0.972 | 2.731 |
| 282 | 365 | 48 | 1kq3−1jq5 | 1kq3_3 | 0.956 | 2.777 |
| 282 | 365 | 48 | 1kq3−1jq5 | 1kq3_4 | 0.983 | 2.859 |
| 282 | 365 | 48 | 1kq3−1jq5 | 1kq3_5 | 0.972 | 2.751 |
| 282 | 365 | 48 | 1kq3−1jq5 | 1kq3_6 | 0.961 | 2.971 |
| 282 | 365 | 48 | 1kq3−1jq5 | 1kq3_7 | 0.953 | 3.113 |
| 292 | 89 | 48 | 1tig−2ife | 1tig_1 | 1.000 | 1.362 |
| 292 | 89 | 48 | 1tig−2ife | 1tig_2 | 1.000 | 1.930 |
| 292 | 89 | 48 | 1tig−2ife | 1tig_3 | 0.886 | 2.388 |
| 292 | 89 | 48 | 1tig−2ife | 1tig_4 | 0.932 | 2.829 |
| 292 | 89 | 48 | 1tig−2ife | 1tig_5 | 0.932 | 2.708 |
| 292 | 89 | 48 | 1tig−2ife | 1tig_6 | 0.932 | 2.618 |
| 292 | 89 | 48 | 1tig−2ife | 1tig_7 | 0.875 | 2.755 |
| 292 | 89 | 48 | 1tig−2ife | 1tig_8 | 0.875 | 2.687 |
| 292 | 89 | 48 | 1tig−2ife | 1tig_9 | 0.852 | 2.669 |
| 292 | 89 | 48 | 1tig−2ife | 1tig_10 | 0.841 | 2.980 |
| 292 | 89 | 48 | 2ife−1tig | 2ife_1 | 0.932 | 2.857 |
| 292 | 89 | 48 | 2ife−1tig | 2ife_2 | 0.932 | 2.442 |
| 292 | 89 | 48 | 2ife−1tig | 2ife_3 | 0.659 | 3.543 |
| 292 | 89 | 48 | 2ife−1tig | 2ife_4 | 0.682 | 3.696 |
| 292 | 89 | 48 | 2ife−1tig | 2ife_5 | 0.682 | 3.884 |
| 292 | 89 | 48 | 2ife−1tig | 2ife_6 | 0.614 | 4.228 |
| 292 | 89 | 48 | 2ife−1tig | 2ife_7 | 0.636 | 4.790 |
| 292 | 89 | 48 | 2ife−1tig | 2ife_8 | 0.580 | 4.573 |
| 292 | 89 | 48 | 2ife−1tig | 2ife_9 | 0.636 | 4.745 |
| 292 | 89 | 48 | 2ife−1tig | 2ife_10 | 0.580 | 5.707 |
| 294 | 310 | 47 | 1b7b−1e19 | 1b7b_1 | 0.957 | 2.603 |
| 294 | 310 | 47 | 1e19−1b7b | 1e19_1 | 1.000 | 1.920 |
| 294 | 310 | 47 | 1e19−1b7b | 1e19_2 | 0.980 | 1.997 |
| 294 | 310 | 47 | 1e19−1b7b | 1e19_3 | 1.000 | 2.384 |
| 296 | 158 | 47 | 1bcf−1jgc | 1bcf_1 | 0.987 | 1.913 |
| 296 | 158 | 47 | 1jgc−1bcf | 1jgc_1 | 1.000 | 3.351 |
| 296 | 158 | 47 | 1jgc−1bcf | 1jgc_2 | 0.968 | 4.270 |
| 296 | 158 | 47 | 1jgc−1bcf | 1jgc_3 | 0.841 | 6.468 |
| 298 | 76 | 47 | 1xbl−1hdj | 1xbl_1 | 0.919 | 3.585 |
| 298 | 76 | 47 | 1xbl−1hdj | 1xbl_2 | 0.892 | 3.751 |
| 298 | 76 | 47 | 1xbl−1hdj | 1xbl_3 | 0.905 | 4.448 |
| 298 | 76 | 47 | 1xbl−1hdj | 1xbl_4 | 0.919 | 4.926 |
| 298 | 76 | 47 | 1xbl−1hdj | 1xbl_5 | 0.811 | 4.907 |
| 298 | 76 | 47 | 1xbl−1hdj | 1xbl_6 | 0.878 | 4.818 |
| 298 | 76 | 47 | 1xbl−1hdj | 1xbl_7 | 0.851 | 4.956 |
| 298 | 76 | 47 | 1xbl−1hdj | 1xbl_8 | 0.851 | 4.670 |

| n | length | %ID | refA−refB | altA | SP | RMSD |
|---|---|---|---|---|---|---|
| 298 | 76 | 47 | 1xbl−1hdj | 1xbl_9 | 0.892 | 4.755 |
| 298 | 76 | 47 | 1xbl−1hdj | 1xbl_10 | 0.959 | 4.861 |
| 298 | 76 | 47 | 1hdj−1xbl | 1hdj_1 | 0.959 | 1.939 |
| 298 | 76 | 47 | 1hdj−1xbl | 1hdj_2 | 0.959 | 3.410 |
| 298 | 76 | 47 | 1hdj−1xbl | 1hdj_3 | 1.000 | 2.918 |
| 298 | 76 | 47 | 1hdj−1xbl | 1hdj_4 | 0.946 | 3.422 |
| 298 | 76 | 47 | 1hdj−1xbl | 1hdj_5 | 0.905 | 3.835 |
| 298 | 76 | 47 | 1hdj−1xbl | 1hdj_6 | 0.892 | 4.193 |
| 298 | 76 | 47 | 1hdj−1xbl | 1hdj_7 | 0.946 | 3.852 |
| 298 | 76 | 47 | 1hdj−1xbl | 1hdj_8 | 0.973 | 3.912 |
| 298 | 76 | 47 | 1hdj−1xbl | 1hdj_9 | 0.973 | 3.888 |
| 298 | 76 | 47 | 1hdj−1xbl | 1hdj_10 | 0.959 | 3.654 |
| 299 | 172 | 47 | 1qqh−1dto | 1qqh_1 | 0.925 | 2.942 |
| 299 | 172 | 47 | 1qqh−1dto | 1qqh_2 | 0.963 | 2.959 |
| 299 | 172 | 47 | 1qqh−1dto | 1qqh_3 | 0.955 | 3.009 |
| 299 | 172 | 47 | 1qqh−1dto | 1qqh_4 | 0.963 | 3.117 |
| 299 | 172 | 47 | 1qqh−1dto | 1qqh_5 | 0.836 | 3.179 |
| 299 | 172 | 47 | 1qqh−1dto | 1qqh_6 | 0.963 | 3.421 |
| 299 | 172 | 47 | 1qqh−1dto | 1qqh_7 | 0.799 | 3.822 |
| 299 | 172 | 47 | 1qqh−1dto | 1qqh_8 | 0.776 | 3.757 |
| 299 | 172 | 47 | 1qqh−1dto | 1qqh_9 | 0.955 | 3.568 |
| 299 | 172 | 47 | 1qqh−1dto | 1qqh_10 | 0.791 | 3.777 |
| 300 | 105 | 47 | 2hgs−1m0w | 2hgs_1 | 1.000 | 1.350 |
| 300 | 105 | 47 | 2hgs−1m0w | 2hgs_2 | 0.961 | 1.828 |
| 300 | 105 | 47 | 2hgs−1m0w | 2hgs_3 | 0.951 | 2.007 |
| 305 | 443 | 47 | 1bj4−1dfo | 1bj4_1 | 0.995 | 6.044 |
| 305 | 443 | 47 | 1bj4−1dfo | 1bj4_2 | 0.983 | 7.432 |
| 305 | 443 | 47 | 1bj4−1dfo | 1bj4_3 | 0.986 | 9.081 |
| 305 | 443 | 47 | 1bj4−1dfo | 1bj4_4 | 0.952 | 9.022 |
| 309 | 210 | 46 | 1k4i−1g57 | 1k4i_1 | 0.975 | 2.480 |
| 309 | 210 | 46 | 1k4i−1g57 | 1k4i_2 | 0.959 | 2.617 |
| 309 | 210 | 46 | 1k4i−1g57 | 1k4i_3 | 0.949 | 2.853 |
| 309 | 210 | 46 | 1k4i−1g57 | 1k4i_4 | 0.944 | 3.324 |
| 309 | 210 | 46 | 1k4i−1g57 | 1k4i_5 | 0.975 | 3.620 |
| 309 | 210 | 46 | 1k4i−1g57 | 1k4i_6 | 0.970 | 4.018 |
| 309 | 210 | 46 | 1k4i−1g57 | 1k4i_7 | 0.944 | 4.238 |
| 309 | 210 | 46 | 1k4i−1g57 | 1k4i_8 | 0.939 | 3.998 |
| 309 | 210 | 46 | 1k4i−1g57 | 1k4i_9 | 0.929 | 3.950 |
| 309 | 210 | 46 | 1k4i−1g57 | 1k4i_10 | 0.934 | 3.947 |
| 310 | 360 | 46 | 1bt4−1bjo | 1bt4_1 | 0.980 | 1.970 |
| 310 | 360 | 46 | 1bt4−1bjo | 1bt4_2 | 0.978 | 2.709 |
| 310 | 360 | 46 | 1bt4−1bjo | 1bt4_3 | 0.975 | 3.869 |
| 310 | 360 | 46 | 1bt4−1bjo | 1bt4_4 | 0.989 | 3.680 |
| 310 | 360 | 46 | 1bt4−1bjo | 1bt4_5 | 0.955 | 3.948 |
| 310 | 360 | 46 | 1bt4−1bjo | 1bt4_6 | 0.961 | 3.987 |
| 310 | 360 | 46 | 1bt4−1bjo | 1bt4_7 | 0.978 | 4.223 |
| 310 | 360 | 46 | 1bt4−1bjo | 1bt4_8 | 0.941 | 4.251 |
| 310 | 360 | 46 | 1bt4−1bjo | 1bt4_9 | 0.941 | 4.779 |
| 310 | 360 | 46 | 1bt4−1bjo | 1bt4_10 | 0.953 | 4.968 |
| 311 | 117 | 46 | 1bl0−1d5y | 1bl0_1 | 0.948 | 4.852 |
| 311 | 117 | 46 | 1bl0−1d5y | 1bl0_2 | 0.638 | 6.698 |
| 311 | 117 | 46 | 1bl0−1d5y | 1bl0_3 | 0.302 | 6.134 |
| 311 | 117 | 46 | 1bl0−1d5y | 1bl0_4 | 0.431 | 6.488 |
| 311 | 117 | 46 | 1bl0−1d5y | 1bl0_5 | 0.405 | 6.205 |
| 311 | 117 | 46 | 1bl0−1d5y | 1bl0_6 | 0.698 | 6.484 |
| 311 | 117 | 46 | 1bl0−1d5y | 1bl0_7 | 0.534 | 5.671 |
| 311 | 117 | 46 | 1bl0−1d5y | 1bl0_8 | 0.457 | 5.821 |
| 311 | 117 | 46 | 1bl0−1d5y | 1bl0_9 | 0.353 | 6.773 |
| 311 | 117 | 46 | 1bl0−1d5y | 1bl0_10 | 0.543 | 6.825 |
| 316 | 123 | 46 | 1ijx−1ijy | 1ijx_1 | 0.975 | 2.700 |
| 316 | 123 | 46 | 1ijy−1ijx | 1ijy_1 | 0.924 | 2.241 |
| 316 | 123 | 46 | 1ijy−1ijx | 1ijy_2 | 0.933 | 2.127 |
| 316 | 123 | 46 | 1ijy−1ijx | 1ijy_3 | 0.916 | 2.507 |
| 316 | 123 | 46 | 1ijy−1ijx | 1ijy_4 | 0.975 | 2.610 |
| 316 | 123 | 46 | 1ijy−1ijx | 1ijy_5 | 0.975 | 2.410 |
| 316 | 123 | 46 | 1ijy−1ijx | 1ijy_6 | 0.975 | 2.360 |
| 321 | 586 | 46 | 1bvz−1sma | 1bvz_1 | 0.983 | 1.582 |
| 321 | 586 | 46 | 1bvz−1sma | 1bvz_2 | 0.972 | 1.652 |
| 322 | 176 | 46 | 1d4o−1e3t | 1d4o_1 | 0.862 | 2.134 |
| 322 | 176 | 46 | 1d4o−1e3t | 1d4o_2 | 0.977 | 1.920 |
| 322 | 176 | 46 | 1d4o−1e3t | 1d4o_3 | 0.977 | 2.136 |

| n | length | %ID | refA−refB | altA | SP | RMSD |
|---|---|---|---|---|---|---|
| 322 | 176 | 46 | 1d4o−1e3t | 1d4o_4 | 0.908 | 2.728 |
| 322 | 176 | 46 | 1d4o−1e3t | 1d4o_5 | 0.908 | 2.969 |
| 322 | 176 | 46 | 1d4o−1e3t | 1d4o_6 | 0.925 | 3.421 |
| 322 | 176 | 46 | 1d4o−1e3t | 1d4o_7 | 0.931 | 3.259 |
| 322 | 176 | 46 | 1d4o−1e3t | 1d4o_8 | 0.977 | 3.902 |
| 322 | 176 | 46 | 1d4o−1e3t | 1d4o_9 | 0.845 | 3.679 |
| 322 | 176 | 46 | 1d4o−1e3t | 1d4o_10 | 0.845 | 3.744 |
| 322 | 176 | 46 | 1e3t−1d4o | 1e3t_1 | 0.879 | 3.681 |
| 322 | 176 | 46 | 1e3t−1d4o | 1e3t_2 | 0.828 | 4.147 |
| 322 | 176 | 46 | 1e3t−1d4o | 1e3t_3 | 0.851 | 4.244 |
| 322 | 176 | 46 | 1e3t−1d4o | 1e3t_4 | 0.891 | 4.194 |
| 322 | 176 | 46 | 1e3t−1d4o | 1e3t_5 | 0.856 | 4.163 |
| 322 | 176 | 46 | 1e3t−1d4o | 1e3t_6 | 0.851 | 4.125 |
| 322 | 176 | 46 | 1e3t−1d4o | 1e3t_7 | 0.885 | 4.114 |
| 322 | 176 | 46 | 1e3t−1d4o | 1e3t_8 | 0.845 | 4.404 |
| 322 | 176 | 46 | 1e3t−1d4o | 1e3t_9 | 0.816 | 4.333 |
| 322 | 176 | 46 | 1e3t−1d4o | 1e3t_10 | 0.856 | 4.378 |
| 323 | 182 | 46 | 1erx−1euo | 1erx_1 | 0.933 | 2.075 |
| 323 | 182 | 46 | 1erx−1euo | 1erx_2 | 0.983 | 2.506 |
| 323 | 182 | 46 | 1erx−1euo | 1erx_3 | 0.983 | 2.955 |
| 323 | 182 | 46 | 1erx−1euo | 1erx_4 | 0.983 | 3.150 |
| 323 | 182 | 46 | 1erx−1euo | 1erx_5 | 0.983 | 3.474 |
| 323 | 182 | 46 | 1erx−1euo | 1erx_6 | 0.927 | 3.646 |
| 323 | 182 | 46 | 1erx−1euo | 1erx_7 | 0.983 | 3.983 |
| 323 | 182 | 46 | 1erx−1euo | 1erx_8 | 0.916 | 3.794 |
| 323 | 182 | 46 | 1erx−1euo | 1erx_9 | 0.983 | 3.836 |
| 323 | 182 | 46 | 1erx−1euo | 1erx_10 | 0.983 | 4.035 |
| 323 | 182 | 46 | 1euo−1erx | 1euo_1 | 0.989 | 2.101 |
| 323 | 182 | 46 | 1euo−1erx | 1euo_2 | 0.978 | 2.219 |
| 323 | 182 | 46 | 1euo−1erx | 1euo_3 | 0.983 | 2.019 |
| 323 | 182 | 46 | 1euo−1erx | 1euo_4 | 0.972 | 2.251 |
| 323 | 182 | 46 | 1euo−1erx | 1euo_5 | 0.972 | 2.005 |
| 323 | 182 | 46 | 1euo−1erx | 1euo_6 | 0.961 | 2.056 |
| 323 | 182 | 46 | 1euo−1erx | 1euo_7 | 0.949 | 2.545 |
| 323 | 182 | 46 | 1euo−1erx | 1euo_8 | 0.933 | 2.681 |
| 323 | 182 | 46 | 1euo−1erx | 1euo_9 | 0.955 | 2.683 |
| 323 | 182 | 46 | 1euo−1erx | 1euo_10 | 0.955 | 2.759 |
| 325 | 60 | 46 | 1ef4−1i50 | 1ef4_1 | 0.519 | 3.464 |
| 326 | 88 | 46 | 1lfd−1rlf | 1lfd_1 | 0.815 | 1.905 |
| 326 | 88 | 46 | 1lfd−1rlf | 1lfd_2 | 0.827 | 2.287 |
| 326 | 88 | 46 | 1rlf−1lfd | 1rlf_1 | 0.753 | 3.263 |
| 326 | 88 | 46 | 1rlf−1lfd | 1rlf_2 | 0.753 | 3.832 |
| 326 | 88 | 46 | 1rlf−1lfd | 1rlf_3 | 0.679 | 3.985 |
| 326 | 88 | 46 | 1rlf−1lfd | 1rlf_4 | 0.580 | 4.278 |
| 326 | 88 | 46 | 1rlf−1lfd | 1rlf_5 | 0.630 | 4.094 |
| 326 | 88 | 46 | 1rlf−1lfd | 1rlf_6 | 0.716 | 4.730 |
| 326 | 88 | 46 | 1rlf−1lfd | 1rlf_7 | 0.654 | 3.826 |
| 326 | 88 | 46 | 1rlf−1lfd | 1rlf_8 | 0.728 | 4.453 |
| 326 | 88 | 46 | 1rlf−1lfd | 1rlf_9 | 0.630 | 4.588 |
| 326 | 88 | 46 | 1rlf−1lfd | 1rlf_10 | 0.494 | 4.374 |
| 337 | 65 | 45 | 1vfy−1dvp | 1vfy_1 | 0.906 | 5.116 |
| 337 | 65 | 45 | 1vfy−1dvp | 1vfy_2 | 0.844 | 6.314 |
| 337 | 65 | 45 | 1vfy−1dvp | 1vfy_3 | 0.406 | 7.213 |
| 337 | 65 | 45 | 1vfy−1dvp | 1vfy_4 | 0.781 | 7.882 |
| 337 | 65 | 45 | 1vfy−1dvp | 1vfy_5 | 0.906 | 7.864 |
| 337 | 65 | 45 | 1vfy−1dvp | 1vfy_6 | 0.906 | 7.830 |
| 337 | 65 | 45 | 1vfy−1dvp | 1vfy_7 | 0.516 | 7.765 |
| 337 | 65 | 45 | 1vfy−1dvp | 1vfy_8 | 0.672 | 7.729 |
| 337 | 65 | 45 | 1vfy−1dvp | 1vfy_9 | 0.469 | 9.245 |
| 337 | 65 | 45 | 1vfy−1dvp | 1vfy_10 | 0.656 | 8.794 |
| 359 | 275 | 44 | 1k4v−1lzj | 1k4v_1 | 0.992 | 1.629 |
| 360 | 392 | 44 | 1feh−1hfe | 1feh_1 | 0.936 | 2.396 |
| 360 | 392 | 44 | 1feh−1hfe | 1feh_2 | 0.947 | 2.681 |
| 360 | 392 | 44 | 1feh−1hfe | 1feh_3 | 0.920 | 2.770 |
| 360 | 392 | 44 | 1feh−1hfe | 1feh_4 | 0.952 | 3.245 |
| 360 | 392 | 44 | 1feh−1hfe | 1feh_5 | 0.923 | 2.946 |
| 360 | 392 | 44 | 1feh−1hfe | 1feh_6 | 0.918 | 2.874 |
| 364 | 168 | 44 | 1l58−1k28 | 1l58_1 | 0.976 | 2.007 |
| 364 | 168 | 44 | 1l58−1k28 | 1l58_2 | 0.976 | 2.133 |
| 364 | 168 | 44 | 1l58−1k28 | 1l58_3 | 0.976 | 2.216 |
| 364 | 168 | 44 | 1l58−1k28 | 1l58_4 | 0.982 | 2.555 |

| n | length | %ID | refA−refB | altA | SP | RMSD |
|---|---|---|---|---|---|---|
| 364 | 168 | 44 | 1l58−1k28 | 1l58_5 | 0.994 | 2.296 |
| 364 | 168 | 44 | 1l58−1k28 | 1l58_6 | 0.945 | 2.156 |
| 364 | 168 | 44 | 1l58−1k28 | 1l58_7 | 0.927 | 3.030 |
| 364 | 168 | 44 | 1l58−1k28 | 1l58_8 | 0.963 | 3.004 |
| 364 | 168 | 44 | 1l58−1k28 | 1l58_9 | 0.933 | 3.371 |
| 364 | 168 | 44 | 1l58−1k28 | 1l58_10 | 0.933 | 3.357 |
| 367 | 74 | 44 | 1dzf−1eik | 1dzf_1 | 0.930 | 2.021 |
| 367 | 74 | 44 | 1dzf−1eik | 1dzf_2 | 0.930 | 2.697 |
| 367 | 74 | 44 | 1dzf−1eik | 1dzf_3 | 0.930 | 3.049 |
| 367 | 74 | 44 | 1dzf−1eik | 1dzf_4 | 0.930 | 3.104 |
| 372 | 286 | 44 | 1b0a−1a4i | 1b0a_1 | 0.993 | 2.835 |
| 372 | 286 | 44 | 1b0a−1a4i | 1b0a_2 | 0.993 | 2.913 |
| 372 | 286 | 44 | 1b0a−1a4i | 1b0a_3 | 0.996 | 3.344 |
| 372 | 286 | 44 | 1b0a−1a4i | 1b0a_4 | 0.996 | 3.068 |
| 372 | 286 | 44 | 1b0a−1a4i | 1b0a_5 | 0.964 | 3.365 |
| 372 | 286 | 44 | 1b0a−1a4i | 1b0a_6 | 0.975 | 3.332 |
| 372 | 286 | 44 | 1b0a−1a4i | 1b0a_7 | 0.978 | 3.466 |
| 372 | 286 | 44 | 1b0a−1a4i | 1b0a_8 | 0.975 | 3.742 |
| 372 | 286 | 44 | 1b0a−1a4i | 1b0a_9 | 0.975 | 3.607 |
| 372 | 286 | 44 | 1b0a−1a4i | 1b0a_10 | 0.957 | 3.055 |
| 377 | 303 | 43 | 1d3v−2cev | 1d3v_1 | 0.966 | 2.277 |
| 377 | 303 | 43 | 1d3v−2cev | 1d3v_2 | 0.932 | 2.623 |
| 381 | 143 | 43 | 1d0i−2dhq | 1d0i_1 | 0.904 | 2.043 |
| 381 | 143 | 43 | 1d0i−2dhq | 1d0i_2 | 0.956 | 1.954 |
| 384 | 94 | 43 | 1hkt−3hts | 1hkt_1 | 0.890 | 4.255 |
| 384 | 94 | 43 | 1hkt−3hts | 1hkt_2 | 1.000 | 5.443 |
| 384 | 94 | 43 | 1hkt−3hts | 1hkt_3 | 0.939 | 6.118 |
| 384 | 94 | 43 | 1hkt−3hts | 1hkt_4 | 0.951 | 5.875 |
| 384 | 94 | 43 | 1hkt−3hts | 1hkt_5 | 0.951 | 6.296 |
| 384 | 94 | 43 | 1hkt−3hts | 1hkt_6 | 0.939 | 5.605 |
| 384 | 94 | 43 | 1hkt−3hts | 1hkt_7 | 0.878 | 5.537 |
| 384 | 94 | 43 | 1hkt−3hts | 1hkt_8 | 1.000 | 5.251 |
| 384 | 94 | 43 | 1hkt−3hts | 1hkt_9 | 1.000 | 4.785 |
| 384 | 94 | 43 | 1hkt−3hts | 1hkt_10 | 0.890 | 4.342 |
| 393 | 73 | 43 | 1b4f−1b0x | 1b4f_1 | 1.000 | 2.273 |
| 393 | 73 | 43 | 1b0x−1b4f | 1b0x_1 | 1.000 | 2.424 |
| 393 | 73 | 43 | 1b0x−1b4f | 1b0x_2 | 1.000 | 2.365 |
| 393 | 73 | 43 | 1b0x−1b4f | 1b0x_3 | 1.000 | 2.334 |
| 393 | 73 | 43 | 1b0x−1b4f | 1b0x_4 | 1.000 | 2.268 |
| 393 | 73 | 43 | 1b0x−1b4f | 1b0x_5 | 0.917 | 2.684 |
| 393 | 73 | 43 | 1b0x−1b4f | 1b0x_6 | 1.000 | 2.819 |
| 393 | 73 | 43 | 1b0x−1b4f | 1b0x_7 | 1.000 | 2.537 |
| 393 | 73 | 43 | 1b0x−1b4f | 1b0x_8 | 1.000 | 2.732 |
| 393 | 73 | 43 | 1b0x−1b4f | 1b0x_9 | 1.000 | 2.769 |
| 393 | 73 | 43 | 1b0x−1b4f | 1b0x_10 | 1.000 | 2.816 |
| 394 | 436 | 43 | 1brw−2tpt | 1brw_1 | 0.979 | 2.238 |
| 394 | 436 | 43 | 1brw−2tpt | 1brw_2 | 0.995 | 2.315 |
| 394 | 436 | 43 | 2tpt−1brw | 2tpt_1 | 0.965 | 2.517 |
| 394 | 436 | 43 | 2tpt−1brw | 2tpt_2 | 0.975 | 3.040 |
| 394 | 436 | 43 | 2tpt−1brw | 2tpt_3 | 0.970 | 3.096 |
| 394 | 436 | 43 | 2tpt−1brw | 2tpt_4 | 0.848 | 3.050 |
| 394 | 436 | 43 | 2tpt−1brw | 2tpt_5 | 0.979 | 3.030 |
| 394 | 436 | 43 | 2tpt−1brw | 2tpt_6 | 0.848 | 3.167 |
| 394 | 436 | 43 | 2tpt−1brw | 2tpt_7 | 0.827 | 3.303 |
| 394 | 436 | 43 | 2tpt−1brw | 2tpt_8 | 0.838 | 3.595 |
| 394 | 436 | 43 | 2tpt−1brw | 2tpt_9 | 0.915 | 3.664 |
| 394 | 436 | 43 | 2tpt−1brw | 2tpt_10 | 0.824 | 3.871 |
| 395 | 181 | 43 | 1br9−1uea | 1br9_1 | 0.942 | 3.153 |
| 395 | 181 | 43 | 1br9−1uea | 1br9_2 | 0.948 | 3.171 |
| 395 | 181 | 43 | 1br9−1uea | 1br9_3 | 0.942 | 3.571 |
| 395 | 181 | 43 | 1br9−1uea | 1br9_4 | 0.954 | 3.848 |
| 395 | 181 | 43 | 1br9−1uea | 1br9_5 | 0.884 | 3.876 |
| 395 | 181 | 43 | 1br9−1uea | 1br9_6 | 0.913 | 3.754 |
| 395 | 181 | 43 | 1br9−1uea | 1br9_7 | 0.948 | 4.391 |
| 395 | 181 | 43 | 1br9−1uea | 1br9_8 | 0.931 | 4.574 |
| 395 | 181 | 43 | 1br9−1uea | 1br9_9 | 0.896 | 4.823 |
| 395 | 181 | 43 | 1br9−1uea | 1br9_10 | 0.925 | 4.284 |
| 400 | 324 | 42 | 1lam−1gyt | 1lam_1 | 0.975 | 1.930 |
| 400 | 324 | 42 | 1lam−1gyt | 1lam_2 | 0.966 | 1.656 |
| 402 | 159 | 42 | 1czt−1d7p | 1czt_1 | 1.000 | 1.717 |
| 402 | 159 | 42 | 1czt−1d7p | 1czt_2 | 0.962 | 1.939 |

| n | length | %ID | refA−refB | altA | SP | RMSD |
|---|---|---|---|---|---|---|
| 402 | 159 | 42 | 1czt−1d7p | 1czt_3 | 1.000 | 1.684 |
| 402 | 159 | 42 | 1czt−1d7p | 1czt_4 | 1.000 | 1.764 |
| 402 | 159 | 42 | 1czt−1d7p | 1czt_5 | 0.994 | 2.150 |
| 402 | 159 | 42 | 1czt−1d7p | 1czt_6 | 1.000 | 1.910 |
| 402 | 159 | 42 | 1czt−1d7p | 1czt_7 | 0.994 | 1.984 |
| 402 | 159 | 42 | 1czt−1d7p | 1czt_8 | 0.987 | 1.908 |
| 402 | 159 | 42 | 1czt−1d7p | 1czt_9 | 1.000 | 2.019 |
| 402 | 159 | 42 | 1czt−1d7p | 1czt_10 | 1.000 | 1.942 |
| 402 | 159 | 42 | 1d7p−1czt | 1d7p_1 | 0.994 | 1.414 |
| 402 | 159 | 42 | 1d7p−1czt | 1d7p_2 | 0.994 | 2.038 |
| 402 | 159 | 42 | 1d7p−1czt | 1d7p_3 | 0.994 | 1.830 |
| 402 | 159 | 42 | 1d7p−1czt | 1d7p_4 | 0.994 | 1.866 |
| 402 | 159 | 42 | 1d7p−1czt | 1d7p_5 | 0.994 | 1.840 |
| 402 | 159 | 42 | 1d7p−1czt | 1d7p_6 | 1.000 | 1.857 |
| 402 | 159 | 42 | 1d7p−1czt | 1d7p_7 | 0.994 | 2.291 |
| 402 | 159 | 42 | 1d7p−1czt | 1d7p_8 | 1.000 | 2.596 |
| 402 | 159 | 42 | 1d7p−1czt | 1d7p_9 | 1.000 | 2.590 |
| 402 | 159 | 42 | 1d7p−1czt | 1d7p_10 | 0.994 | 2.373 |
| 406 | 689 | 42 | 1gqi−1k9d | 1gqi_1 | 0.940 | 1.834 |
| 406 | 689 | 42 | 1gqi−1k9d | 1gqi_2 | 0.936 | 1.686 |
| 408 | 74 | 42 | 1ghc−1hst | 1ghc_1 | 0.753 | 5.685 |
| 408 | 74 | 42 | 1ghc−1hst | 1ghc_2 | 0.342 | 5.300 |
| 408 | 74 | 42 | 1ghc−1hst | 1ghc_3 | 0.384 | 6.003 |
| 408 | 74 | 42 | 1ghc−1hst | 1ghc_4 | 0.699 | 5.918 |
| 408 | 74 | 42 | 1ghc−1hst | 1ghc_5 | 0.534 | 5.949 |
| 408 | 74 | 42 | 1ghc−1hst | 1ghc_6 | 0.329 | 5.806 |
| 408 | 74 | 42 | 1ghc−1hst | 1ghc_7 | 0.521 | 6.067 |
| 408 | 74 | 42 | 1ghc−1hst | 1ghc_8 | 0.301 | 6.536 |
| 408 | 74 | 42 | 1ghc−1hst | 1ghc_9 | 0.055 | 7.111 |
| 408 | 74 | 42 | 1ghc−1hst | 1ghc_10 | 0.055 | 6.792 |
| 412 | 286 | 42 | 1qpo−1qap | 1qpo_1 | 0.971 | 1.702 |
| 413 | 127 | 42 | 1whi−1jj2 | 1whi_1 | 0.941 | 3.035 |
| 413 | 127 | 42 | 1whi−1jj2 | 1whi_2 | 0.992 | 3.281 |
| 413 | 127 | 42 | 1whi−1jj2 | 1whi_3 | 0.992 | 3.105 |
| 413 | 127 | 42 | 1whi−1jj2 | 1whi_4 | 0.941 | 3.832 |
| 413 | 127 | 42 | 1whi−1jj2 | 1whi_5 | 0.949 | 3.666 |
| 413 | 127 | 42 | 1whi−1jj2 | 1whi_6 | 0.941 | 3.958 |
| 413 | 127 | 42 | 1whi−1jj2 | 1whi_7 | 0.941 | 3.671 |
| 413 | 127 | 42 | 1whi−1jj2 | 1whi_8 | 0.975 | 4.117 |
| 413 | 127 | 42 | 1whi−1jj2 | 1whi_9 | 0.941 | 4.445 |
| 413 | 127 | 42 | 1whi−1jj2 | 1whi_10 | 0.975 | 4.198 |
| 415 | 73 | 42 | 2eia−1baj | 2eia_1 | 1.000 | 1.135 |
| 415 | 73 | 42 | 2eia−1baj | 2eia_2 | 1.000 | 2.555 |
| 415 | 73 | 42 | 2eia−1baj | 2eia_3 | 1.000 | 3.236 |
| 415 | 73 | 42 | 2eia−1baj | 2eia_4 | 1.000 | 3.681 |
| 415 | 73 | 42 | 2eia−1baj | 2eia_5 | 1.000 | 4.080 |
| 415 | 73 | 42 | 2eia−1baj | 2eia_6 | 1.000 | 3.116 |
| 415 | 73 | 42 | 1baj−2eia | 1baj_1 | 1.000 | 3.268 |
| 415 | 73 | 42 | 1baj−2eia | 1baj_2 | 0.930 | 2.963 |
| 415 | 73 | 42 | 1baj−2eia | 1baj_3 | 0.845 | 3.285 |
| 415 | 73 | 42 | 1baj−2eia | 1baj_4 | 0.930 | 3.509 |
| 415 | 73 | 42 | 1baj−2eia | 1baj_5 | 0.930 | 3.279 |
| 415 | 73 | 42 | 1baj−2eia | 1baj_6 | 1.000 | 3.285 |
| 415 | 73 | 42 | 1baj−2eia | 1baj_7 | 1.000 | 3.268 |
| 415 | 73 | 42 | 1baj−2eia | 1baj_8 | 0.845 | 3.489 |
| 415 | 73 | 42 | 1baj−2eia | 1baj_9 | 0.930 | 3.206 |
| 415 | 73 | 42 | 1baj−2eia | 1baj_10 | 0.803 | 3.275 |
| 416 | 55 | 41 | 1l1c−1auu | 1l1c_1 | 0.963 | 1.729 |
| 416 | 55 | 41 | 1l1c−1auu | 1l1c_2 | 0.981 | 1.787 |
| 416 | 55 | 41 | 1l1c−1auu | 1l1c_3 | 0.981 | 1.711 |
| 416 | 55 | 41 | 1l1c−1auu | 1l1c_4 | 0.981 | 1.693 |
| 416 | 55 | 41 | 1l1c−1auu | 1l1c_5 | 0.981 | 2.102 |
| 416 | 55 | 41 | 1l1c−1auu | 1l1c_6 | 0.981 | 1.839 |
| 416 | 55 | 41 | 1l1c−1auu | 1l1c_7 | 1.000 | 2.168 |
| 416 | 55 | 41 | 1l1c−1auu | 1l1c_8 | 0.963 | 1.774 |
| 416 | 55 | 41 | 1l1c−1auu | 1l1c_9 | 0.981 | 2.135 |
| 416 | 55 | 41 | 1l1c−1auu | 1l1c_10 | 0.981 | 2.302 |
| 416 | 55 | 41 | 1auu−1l1c | 1auu_1 | 0.796 | 4.068 |
| 416 | 55 | 41 | 1auu−1l1c | 1auu_2 | 0.981 | 3.869 |
| 416 | 55 | 41 | 1auu−1l1c | 1auu_3 | 0.815 | 4.124 |
| 416 | 55 | 41 | 1auu−1l1c | 1auu_4 | 0.778 | 4.723 |

n: internal numbering; length of alignment; % sequence%ID (cf. Homstrad); refA−refB: reference alignment; altA: alternative snapshot for A; SP: sum−of−pairs score of alt. alignment w/r reference

| n | length | %ID | refA−refB | altA | SP | RMSD |
|---|---|---|---|---|---|---|
| 416 | 55 | 41 | 1auu−1l1c | 1auu_5 | 0.685 | 4.954 |
| 416 | 55 | 41 | 1auu−1l1c | 1auu_6 | 0.741 | 5.404 |
| 416 | 55 | 41 | 1auu−1l1c | 1auu_7 | 0.870 | 5.368 |
| 416 | 55 | 41 | 1auu−1l1c | 1auu_8 | 0.796 | 5.322 |
| 416 | 55 | 41 | 1auu−1l1c | 1auu_9 | 0.852 | 5.776 |
| 416 | 55 | 41 | 1auu−1l1c | 1auu_10 | 0.852 | 5.894 |
| 432 | 52 | 40 | 1pdc−2fn2 | 1pdc_1 | 1.000 | 2.766 |
| 432 | 52 | 40 | 1pdc−2fn2 | 1pdc_2 | 0.933 | 2.454 |
| 432 | 52 | 40 | 1pdc−2fn2 | 1pdc_3 | 0.911 | 2.600 |
| 432 | 52 | 40 | 1pdc−2fn2 | 1pdc_4 | 0.911 | 2.890 |
| 432 | 52 | 40 | 1pdc−2fn2 | 1pdc_5 | 0.933 | 2.492 |
| 432 | 52 | 40 | 1pdc−2fn2 | 1pdc_6 | 0.933 | 3.102 |
| 432 | 52 | 40 | 1pdc−2fn2 | 1pdc_7 | 0.933 | 2.693 |
| 432 | 52 | 40 | 1pdc−2fn2 | 1pdc_8 | 0.933 | 2.833 |
| 432 | 52 | 40 | 1pdc−2fn2 | 1pdc_9 | 0.933 | 2.681 |
| 432 | 52 | 40 | 1pdc−2fn2 | 1pdc_10 | 0.933 | 2.605 |
| 432 | 52 | 40 | 2fn2−1pdc | 2fn2_1 | 0.978 | 1.794 |
| 432 | 52 | 40 | 2fn2−1pdc | 2fn2_2 | 0.978 | 2.307 |
| 432 | 52 | 40 | 2fn2−1pdc | 2fn2_3 | 0.978 | 2.605 |
| 432 | 52 | 40 | 2fn2−1pdc | 2fn2_4 | 0.956 | 2.655 |
| 432 | 52 | 40 | 2fn2−1pdc | 2fn2_5 | 0.933 | 2.539 |
| 432 | 52 | 40 | 2fn2−1pdc | 2fn2_6 | 0.933 | 1.831 |
| 432 | 52 | 40 | 2fn2−1pdc | 2fn2_7 | 0.978 | 1.945 |
| 432 | 52 | 40 | 2fn2−1pdc | 2fn2_8 | 0.978 | 1.658 |
| 432 | 52 | 40 | 2fn2−1pdc | 2fn2_9 | 0.933 | 2.200 |
| 432 | 52 | 40 | 2fn2−1pdc | 2fn2_10 | 0.978 | 2.262 |
| 436 | 55 | 40 | 1h9f−1jei | 1h9f_1 | 0.980 | 5.211 |
| 436 | 55 | 40 | 1h9f−1jei | 1h9f_2 | 0.980 | 4.875 |
| 436 | 55 | 40 | 1h9f−1jei | 1h9f_3 | 0.440 | 6.403 |
| 436 | 55 | 40 | 1h9f−1jei | 1h9f_4 | 0.800 | 7.079 |
| 436 | 55 | 40 | 1h9f−1jei | 1h9f_5 | 0.800 | 6.495 |
| 436 | 55 | 40 | 1h9f−1jei | 1h9f_6 | 0.360 | 6.538 |
| 436 | 55 | 40 | 1h9f−1jei | 1h9f_7 | 0.400 | 7.650 |
| 436 | 55 | 40 | 1h9f−1jei | 1h9f_8 | 0.500 | 7.133 |
| 436 | 55 | 40 | 1h9f−1jei | 1h9f_9 | 0.300 | 6.776 |
| 436 | 55 | 40 | 1h9f−1jei | 1h9f_10 | 0.540 | 6.396 |
| 436 | 55 | 40 | 1jei−1h9f | 1jei_1 | 0.540 | 4.817 |
| 436 | 55 | 40 | 1jei−1h9f | 1jei_2 | 0.840 | 4.964 |
| 436 | 55 | 40 | 1jei−1h9f | 1jei_3 | 0.840 | 5.006 |
| 436 | 55 | 40 | 1jei−1h9f | 1jei_4 | 0.840 | 4.923 |
| 436 | 55 | 40 | 1jei−1h9f | 1jei_5 | 1.000 | 4.809 |
| 436 | 55 | 40 | 1jei−1h9f | 1jei_6 | 1.000 | 5.351 |
| 436 | 55 | 40 | 1jei−1h9f | 1jei_7 | 1.000 | 5.052 |
| 436 | 55 | 40 | 1jei−1h9f | 1jei_8 | 0.840 | 5.212 |
| 436 | 55 | 40 | 1jei−1h9f | 1jei_9 | 0.900 | 4.802 |
| 436 | 55 | 40 | 1jei−1h9f | 1jei_10 | 0.880 | 5.129 |
| 438 | 452 | 40 | 1cb5−1gcb | 1cb5_1 | 0.948 | 4.364 |
| 438 | 452 | 40 | 1gcb−1cb5 | 1gcb_1 | 0.993 | 2.595 |
| 438 | 452 | 40 | 1gcb−1cb5 | 1gcb_2 | 0.946 | 2.629 |
| 438 | 452 | 40 | 1gcb−1cb5 | 1gcb_3 | 0.943 | 3.230 |
| 438 | 452 | 40 | 1gcb−1cb5 | 1gcb_4 | 0.989 | 3.112 |
| 438 | 452 | 40 | 1gcb−1cb5 | 1gcb_5 | 0.984 | 3.268 |
| 443 | 119 | 40 | 1c44−1ikt | 1c44_1 | 0.875 | 2.871 |
| 443 | 119 | 40 | 1c44−1ikt | 1c44_2 | 0.884 | 4.064 |
| 443 | 119 | 40 | 1c44−1ikt | 1c44_3 | 0.821 | 4.314 |
| 443 | 119 | 40 | 1c44−1ikt | 1c44_4 | 0.866 | 4.806 |
| 443 | 119 | 40 | 1c44−1ikt | 1c44_5 | 0.857 | 5.085 |
| 443 | 119 | 40 | 1c44−1ikt | 1c44_6 | 0.857 | 4.789 |
| 443 | 119 | 40 | 1c44−1ikt | 1c44_7 | 0.848 | 5.020 |
| 443 | 119 | 40 | 1c44−1ikt | 1c44_8 | 0.866 | 4.399 |
| 443 | 119 | 40 | 1c44−1ikt | 1c44_9 | 0.848 | 4.806 |
| 443 | 119 | 40 | 1c44−1ikt | 1c44_10 | 0.893 | 4.644 |
| 445 | 108 | 40 | 1a1x−1jsg | 1a1x_1 | 0.943 | 1.591 |
| 445 | 108 | 40 | 1a1x−1jsg | 1a1x_2 | 0.943 | 2.311 |
| 445 | 108 | 40 | 1a1x−1jsg | 1a1x_3 | 0.877 | 2.530 |
| 445 | 108 | 40 | 1a1x−1jsg | 1a1x_4 | 0.877 | 2.741 |
| 445 | 108 | 40 | 1a1x−1jsg | 1a1x_5 | 0.811 | 2.831 |
| 445 | 108 | 40 | 1a1x−1jsg | 1a1x_6 | 0.811 | 3.190 |
| 445 | 108 | 40 | 1a1x−1jsg | 1a1x_7 | 0.811 | 3.117 |
| 445 | 108 | 40 | 1a1x−1jsg | 1a1x_8 | 0.811 | 3.108 |
| 445 | 108 | 40 | 1a1x−1jsg | 1a1x_9 | 0.877 | 3.264 |
| 445 | 108 | 40 | 1a1x−1jsg | 1a1x_10 | 0.877 | 3.226 |
| 446 | 53 | 40 | 1tfi−1qyp | 1tfi_1 | 0.959 | 3.104 |
| 446 | 53 | 40 | 1tfi−1qyp | 1tfi_2 | 0.816 | 2.262 |
| 446 | 53 | 40 | 1tfi−1qyp | 1tfi_3 | 0.959 | 3.433 |
| 446 | 53 | 40 | 1tfi−1qyp | 1tfi_4 | 0.816 | 4.798 |
| 446 | 53 | 40 | 1tfi−1qyp | 1tfi_5 | 0.000 | 5.335 |
| 446 | 53 | 40 | 1tfi−1qyp | 1tfi_6 | 0.816 | 4.852 |
| 446 | 53 | 40 | 1tfi−1qyp | 1tfi_7 | 0.816 | 5.097 |
| 446 | 53 | 40 | 1tfi−1qyp | 1tfi_8 | 0.837 | 5.368 |
| 446 | 53 | 40 | 1tfi−1qyp | 1tfi_9 | 0.980 | 4.938 |
| 446 | 53 | 40 | 1tfi−1qyp | 1tfi_10 | 0.673 | 6.057 |
| 446 | 53 | 40 | 1qyp−1tfi | 1qyp_1 | 0.980 | 3.840 |
| 446 | 53 | 40 | 1qyp−1tfi | 1qyp_2 | 0.857 | 2.743 |
| 446 | 53 | 40 | 1qyp−1tfi | 1qyp_3 | 0.980 | 3.378 |
| 446 | 53 | 40 | 1qyp−1tfi | 1qyp_4 | 0.857 | 3.933 |
| 446 | 53 | 40 | 1qyp−1tfi | 1qyp_5 | 0.776 | 4.447 |
| 446 | 53 | 40 | 1qyp−1tfi | 1qyp_6 | 0.980 | 4.905 |
| 446 | 53 | 40 | 1qyp−1tfi | 1qyp_7 | 0.816 | 4.324 |
| 446 | 53 | 40 | 1qyp−1tfi | 1qyp_8 | 0.918 | 4.679 |
| 446 | 53 | 40 | 1qyp−1tfi | 1qyp_9 | 0.980 | 3.681 |
| 446 | 53 | 40 | 1qyp−1tfi | 1qyp_10 | 0.918 | 3.382 |
| 447 | 433 | 40 | 1tubb−1tuba | 1tubb_1 | 0.895 | 3.208 |
| 447 | 433 | 40 | 1tubb−1tuba | 1tubb_2 | 0.793 | 3.513 |
| 447 | 433 | 40 | 1tubb−1tuba | 1tuba_1 | 0.912 | 2.900 |
| 447 | 433 | 40 | 1tubb−1tuba | 1tuba_2 | 0.867 | 3.349 |
| 448 | 354 | 39 | 1jdw−1bwd | 1jdw_1 | 0.977 | 1.563 |
| 448 | 354 | 39 | 1jdw−1bwd | 1jdw_2 | 0.944 | 1.694 |
| 448 | 354 | 39 | 1jdw−1bwd | 1jdw_3 | 0.962 | 1.546 |
| 448 | 354 | 39 | 1jdw−1bwd | 1jdw_4 | 0.962 | 1.770 |
| 448 | 354 | 39 | 1jdw−1bwd | 1jdw_5 | 0.968 | 1.923 |
| 448 | 354 | 39 | 1jdw−1bwd | 1jdw_6 | 0.974 | 1.674 |
| 448 | 354 | 39 | 1jdw−1bwd | 1jdw_7 | 0.942 | 1.678 |
| 448 | 354 | 39 | 1jdw−1bwd | 1jdw_8 | 0.980 | 1.756 |
| 448 | 354 | 39 | 1jdw−1bwd | 1jdw_9 | 0.977 | 1.790 |
| 448 | 354 | 39 | 1jdw−1bwd | 1jdw_10 | 0.977 | 1.863 |
| 448 | 354 | 39 | 1bwd−1jdw | 1bwd_1 | 0.971 | 2.145 |
| 448 | 354 | 39 | 1bwd−1jdw | 1bwd_2 | 0.944 | 2.319 |
| 449 | 39 | 39 | 1ica−1fjn | 1ica_1 | 0.647 | 5.202 |
| 457 | 83 | 39 | 1qk9−1ig4 | 1qk9_1 | 0.438 | 6.125 |
| 457 | 83 | 39 | 1qk9−1ig4 | 1qk9_2 | 0.781 | 5.480 |
| 457 | 83 | 39 | 1qk9−1ig4 | 1qk9_3 | 0.753 | 8.019 |
| 457 | 83 | 39 | 1qk9−1ig4 | 1qk9_4 | 0.699 | 7.628 |
| 457 | 83 | 39 | 1qk9−1ig4 | 1qk9_5 | 0.781 | 8.326 |
| 457 | 83 | 39 | 1qk9−1ig4 | 1qk9_6 | 0.753 | 7.517 |
| 457 | 83 | 39 | 1qk9−1ig4 | 1qk9_7 | 0.781 | 8.284 |
| 457 | 83 | 39 | 1qk9−1ig4 | 1qk9_8 | 0.740 | 7.707 |
| 457 | 83 | 39 | 1qk9−1ig4 | 1qk9_9 | 0.767 | 8.680 |
| 457 | 83 | 39 | 1qk9−1ig4 | 1qk9_10 | 0.808 | 7.131 |
| 458 | 72 | 39 | 1tnt−1qpm | 1tnt_1 | 0.913 | 6.239 |
| 458 | 72 | 39 | 1tnt−1qpm | 1tnt_2 | 0.928 | 7.796 |
| 458 | 72 | 39 | 1tnt−1qpm | 1tnt_3 | 0.855 | 9.017 |
| 458 | 72 | 39 | 1tnt−1qpm | 1tnt_4 | 0.942 | 8.509 |
| 458 | 72 | 39 | 1tnt−1qpm | 1tnt_5 | 0.855 | 6.373 |
| 458 | 72 | 39 | 1tnt−1qpm | 1tnt_6 | 0.855 | 8.112 |
| 458 | 72 | 39 | 1tnt−1qpm | 1tnt_7 | 0.826 | 7.768 |
| 458 | 72 | 39 | 1tnt−1qpm | 1tnt_8 | 0.841 | 7.700 |
| 458 | 72 | 39 | 1tnt−1qpm | 1tnt_9 | 0.826 | 7.104 |
| 458 | 72 | 39 | 1tnt−1qpm | 1tnt_10 | 0.841 | 7.377 |
| 458 | 72 | 39 | 1qpm−1tnt | 1qpm_1 | 0.913 | 3.045 |
| 458 | 72 | 39 | 1qpm−1tnt | 1qpm_2 | 0.913 | 3.825 |
| 458 | 72 | 39 | 1qpm−1tnt | 1qpm_3 | 0.928 | 4.650 |
| 458 | 72 | 39 | 1qpm−1tnt | 1qpm_4 | 0.957 | 4.918 |
| 458 | 72 | 39 | 1qpm−1tnt | 1qpm_5 | 0.855 | 4.716 |
| 458 | 72 | 39 | 1qpm−1tnt | 1qpm_6 | 0.928 | 4.672 |
| 458 | 72 | 39 | 1qpm−1tnt | 1qpm_7 | 0.942 | 5.043 |
| 458 | 72 | 39 | 1qpm−1tnt | 1qpm_8 | 0.913 | 4.675 |
| 458 | 72 | 39 | 1qpm−1tnt | 1qpm_9 | 0.899 | 4.757 |
| 458 | 72 | 39 | 1qpm−1tnt | 1qpm_10 | 0.884 | 4.530 |
| 462 | 118 | 39 | 1dlw−1dly | 1dlw_1 | 0.991 | 2.922 |
| 462 | 118 | 39 | 1dlw−1dly | 1dlw_2 | 0.974 | 3.187 |
| 462 | 118 | 39 | 1dlw−1dly | 1dlw_3 | 0.974 | 3.345 |
| 462 | 118 | 39 | 1dlw−1dly | 1dlw_4 | 0.966 | 3.278 |
| 462 | 118 | 39 | 1dlw−1dly | 1dlw_5 | 0.983 | 3.336 |
| 462 | 118 | 39 | 1dlw−1dly | 1dlw_6 | 0.931 | 3.422 |
| 462 | 118 | 39 | 1dlw−1dly | 1dlw_7 | 0.940 | 3.370 |
| 462 | 118 | 39 | 1dlw−1dly | 1dlw_8 | 0.897 | 3.595 |
| 462 | 118 | 39 | 1dlw−1dly | 1dlw_9 | 0.914 | 3.622 |
| 462 | 118 | 39 | 1dlw−1dly | 1dlw_10 | 0.862 | 3.209 |
| 462 | 118 | 39 | 1dly−1dlw | 1dly_1 | 0.983 | 2.321 |
| 462 | 118 | 39 | 1dly−1dlw | 1dly_2 | 1.000 | 3.548 |
| 462 | 118 | 39 | 1dly−1dlw | 1dly_3 | 1.000 | 3.424 |
| 462 | 118 | 39 | 1dly−1dlw | 1dly_4 | 1.000 | 2.897 |
| 462 | 118 | 39 | 1dly−1dlw | 1dly_5 | 0.991 | 3.750 |
| 462 | 118 | 39 | 1dly−1dlw | 1dly_6 | 1.000 | 3.627 |
| 462 | 118 | 39 | 1dly−1dlw | 1dly_7 | 0.948 | 3.259 |
| 462 | 118 | 39 | 1dly−1dlw | 1dly_8 | 1.000 | 3.820 |
| 462 | 118 | 39 | 1dly−1dlw | 1dly_9 | 0.957 | 3.933 |
| 462 | 118 | 39 | 1dly−1dlw | 1dly_10 | 0.914 | 3.332 |
| 464 | 306 | 39 | 1tco−1fjm | 1tco_1 | 0.990 | 1.891 |
| 470 | 472 | 38 | 1dnp−1qnf | 1dnp_1 | 0.954 | 1.917 |
| 470 | 472 | 38 | 1qnf−1dnp | 1qnf_1 | 0.958 | 2.299 |
| 470 | 472 | 38 | 1qnf−1dnp | 1qnf_2 | 0.962 | 2.712 |
| 470 | 472 | 38 | 1qnf−1dnp | 1qnf_3 | 0.958 | 2.635 |
| 470 | 472 | 38 | 1qnf−1dnp | 1qnf_4 | 0.918 | 2.482 |
| 470 | 472 | 38 | 1qnf−1dnp | 1qnf_5 | 0.958 | 2.520 |
| 470 | 472 | 38 | 1qnf−1dnp | 1qnf_6 | 0.927 | 2.763 |
| 472 | 212 | 38 | 1j90−1jag | 1j90_1 | 0.969 | 1.939 |
| 472 | 212 | 38 | 1j90−1jag | 1j90_2 | 0.943 | 2.375 |
| 472 | 212 | 38 | 1j90−1jag | 1j90_3 | 0.959 | 2.839 |
| 472 | 212 | 38 | 1j90−1jag | 1j90_4 | 0.954 | 3.402 |
| 473 | 273 | 38 | 1ajz−1ad1 | 1ajz_1 | 0.874 | 2.577 |
| 473 | 273 | 38 | 1ajz−1ad1 | 1ajz_2 | 0.900 | 2.657 |
| 473 | 273 | 38 | 1ajz−1ad1 | 1ajz_3 | 0.900 | 2.829 |
| 473 | 273 | 38 | 1ajz−1ad1 | 1ajz_4 | 0.858 | 3.105 |
| 473 | 273 | 38 | 1ajz−1ad1 | 1ajz_5 | 0.881 | 2.768 |
| 473 | 273 | 38 | 1ajz−1ad1 | 1ajz_6 | 0.927 | 3.018 |
| 473 | 273 | 38 | 1ajz−1ad1 | 1ajz_7 | 0.977 | 2.965 |
| 473 | 273 | 38 | 1ajz−1ad1 | 1ajz_8 | 0.847 | 3.243 |
| 473 | 273 | 38 | 1ajz−1ad1 | 1ajz_9 | 0.866 | 3.109 |
| 473 | 273 | 38 | 1ajz−1ad1 | 1ajz_10 | 0.881 | 3.239 |
| 474 | 126 | 38 | 1c0a−1g51 | 1c0a_1 | 0.958 | 1.745 |
| 474 | 126 | 38 | 1c0a−1g51 | 1c0a_2 | 0.967 | 2.008 |
| 474 | 126 | 38 | 1c0a−1g51 | 1c0a_3 | 0.958 | 2.029 |
| 474 | 126 | 38 | 1c0a−1g51 | 1c0a_4 | 0.975 | 2.238 |
| 474 | 126 | 38 | 1c0a−1g51 | 1c0a_5 | 0.983 | 2.579 |
| 474 | 126 | 38 | 1g51−1c0a | 1g51_1 | 0.992 | 1.200 |
| 477 | 107 | 38 | 1eq3−1pin | 1eq3_1 | 0.534 | 3.968 |
| 477 | 107 | 38 | 1eq3−1pin | 1eq3_2 | 0.659 | 4.500 |
| 477 | 107 | 38 | 1eq3−1pin | 1eq3_3 | 0.557 | 4.603 |
| 477 | 107 | 38 | 1eq3−1pin | 1eq3_4 | 0.534 | 5.698 |
| 477 | 107 | 38 | 1eq3−1pin | 1eq3_5 | 0.545 | 5.972 |
| 477 | 107 | 38 | 1eq3−1pin | 1eq3_6 | 0.534 | 5.729 |
| 477 | 107 | 38 | 1eq3−1pin | 1eq3_7 | 0.659 | 5.950 |
| 477 | 107 | 38 | 1eq3−1pin | 1eq3_8 | 0.580 | 6.088 |
| 477 | 107 | 38 | 1eq3−1pin | 1eq3_9 | 0.568 | 5.315 |
| 477 | 107 | 38 | 1eq3−1pin | 1eq3_10 | 0.568 | 5.917 |
| 481 | 159 | 38 | 1iq4−1jj2 | 1iq4_1 | 1.000 | 2.469 |
| 481 | 159 | 38 | 1iq4−1jj2 | 1iq4_2 | 0.958 | 3.316 |
| 481 | 159 | 38 | 1iq4−1jj2 | 1iq4_3 | 1.000 | 2.812 |
| 481 | 159 | 38 | 1iq4−1jj2 | 1iq4_4 | 0.942 | 2.603 |
| 490 | 152 | 37 | 1ulo−1cx1 | 1ulo_1 | 0.979 | 2.814 |
| 490 | 152 | 37 | 1ulo−1cx1 | 1ulo_2 | 0.924 | 2.688 |
| 490 | 152 | 37 | 1ulo−1cx1 | 1ulo_3 | 0.938 | 2.742 |
| 490 | 152 | 37 | 1ulo−1cx1 | 1ulo_4 | 0.717 | 2.825 |
| 490 | 152 | 37 | 1ulo−1cx1 | 1ulo_5 | 0.938 | 3.292 |
| 490 | 152 | 37 | 1ulo−1cx1 | 1ulo_6 | 0.697 | 3.661 |
| 490 | 152 | 37 | 1ulo−1cx1 | 1ulo_7 | 0.910 | 3.619 |
| 490 | 152 | 37 | 1ulo−1cx1 | 1ulo_8 | 0.903 | 3.685 |
| 490 | 152 | 37 | 1ulo−1cx1 | 1ulo_9 | 0.683 | 3.756 |
| 490 | 152 | 37 | 1ulo−1cx1 | 1ulo_10 | 0.717 | 4.023 |
| 490 | 152 | 37 | 1cx1−1ulo | 1cx1_1 | 0.979 | 3.247 |
| 490 | 152 | 37 | 1cx1−1ulo | 1cx1_2 | 0.986 | 3.132 |

n: internal numbering; length of alignment; % sequence%ID (cf. Homstrad); refA−refB: reference alignment; altA: alternative snapshot for A; SP: sum−of−pairs score of alt. alignment w/r reference 

| n | length | %ID | refA−refB | altA | SP | RMSD |
|---|---|---|---|---|---|---|
| 490 | 152 | 37 | 1cx1−1ulo | 1cx1_3 | 0.938 | 3.049 |
| 490 | 152 | 37 | 1cx1−1ulo | 1cx1_4 | 0.986 | 3.232 |
| 490 | 152 | 37 | 1cx1−1ulo | 1cx1_5 | 0.986 | 2.859 |
| 490 | 152 | 37 | 1cx1−1ulo | 1cx1_6 | 0.979 | 3.283 |
| 490 | 152 | 37 | 1cx1−1ulo | 1cx1_7 | 0.959 | 3.114 |
| 490 | 152 | 37 | 1cx1−1ulo | 1cx1_8 | 0.910 | 3.223 |
| 490 | 152 | 37 | 1cx1−1ulo | 1cx1_9 | 0.924 | 3.335 |
| 490 | 152 | 37 | 1cx1−1ulo | 1cx1_10 | 0.952 | 3.711 |
| 492 | 90 | 37 | 1qce−1df4 | 1qce_1 | 0.930 | 2.023 |
| 492 | 90 | 37 | 1qce−1df4 | 1qce_2 | 0.930 | 2.431 |
| 502 | 171 | 37 | 1auy−1qjz | 1auy_1 | 0.907 | 2.097 |
| 502 | 171 | 37 | 1auy−1qjz | 1auy_2 | 0.938 | 2.078 |
| 502 | 171 | 37 | 1auy−1qjz | 1auy_3 | 0.938 | 1.973 |
| 502 | 171 | 37 | 1auy−1qjz | 1auy_4 | 0.932 | 2.007 |
| 502 | 171 | 37 | 1auy−1qjz | 1auy_5 | 0.925 | 2.422 |
| 502 | 171 | 37 | 1auy−1qjz | 1auy_6 | 0.901 | 2.269 |
| 502 | 171 | 37 | 1auy−1qjz | 1auy_7 | 0.894 | 2.358 |
| 507 | 106 | 36 | 1dqi−1dfx | 1dqi_1 | 0.921 | 1.384 |
| 507 | 106 | 36 | 1dqi−1dfx | 1dqi_2 | 0.944 | 1.510 |
| 507 | 106 | 36 | 1dqi−1dfx | 1dqi_3 | 0.989 | 1.633 |
| 507 | 106 | 36 | 1dqi−1dfx | 1dqi_4 | 0.989 | 1.893 |
| 507 | 106 | 36 | 1dqi−1dfx | 1dqi_5 | 0.921 | 2.128 |
| 507 | 106 | 36 | 1dqi−1dfx | 1dqi_6 | 0.933 | 2.048 |
| 507 | 106 | 36 | 1dfx−1dqi | 1dfx_1 | 0.978 | 1.725 |
| 507 | 106 | 36 | 1dfx−1dqi | 1dfx_2 | 0.888 | 2.206 |
| 507 | 106 | 36 | 1dfx−1dqi | 1dfx_3 | 0.831 | 2.628 |
| 507 | 106 | 36 | 1dfx−1dqi | 1dfx_4 | 0.843 | 2.538 |
| 507 | 106 | 36 | 1dfx−1dqi | 1dfx_5 | 0.831 | 2.548 |
| 507 | 106 | 36 | 1dfx−1dqi | 1dfx_6 | 0.831 | 2.606 |
| 507 | 106 | 36 | 1dfx−1dqi | 1dfx_7 | 0.876 | 2.797 |
| 507 | 106 | 36 | 1dfx−1dqi | 1dfx_8 | 0.888 | 2.851 |
| 507 | 106 | 36 | 1dfx−1dqi | 1dfx_9 | 0.876 | 2.975 |
| 507 | 106 | 36 | 1dfx−1dqi | 1dfx_10 | 0.640 | 3.019 |
| 512 | 482 | 36 | 1gai−1ayx | 1gai_1 | 0.930 | 1.984 |
| 512 | 482 | 36 | 1gai−1ayx | 1gai_2 | 0.951 | 2.324 |
| 512 | 482 | 36 | 1gai−1ayx | 1gai_3 | 0.935 | 2.661 |
| 512 | 482 | 36 | 1gai−1ayx | 1gai_4 | 0.895 | 2.706 |
| 512 | 482 | 36 | 1gai−1ayx | 1gai_5 | 0.879 | 2.898 |
| 512 | 482 | 36 | 1gai−1ayx | 1gai_6 | 0.923 | 3.059 |
| 512 | 482 | 36 | 1gai−1ayx | 1gai_7 | 0.930 | 3.160 |
| 512 | 482 | 36 | 1gai−1ayx | 1gai_8 | 0.898 | 3.293 |
| 512 | 482 | 36 | 1gai−1ayx | 1gai_9 | 0.914 | 2.975 |
| 512 | 482 | 36 | 1gai−1ayx | 1gai_10 | 0.881 | 3.162 |
| 512 | 482 | 36 | 1ayx−1gai | 1ayx_1 | 0.958 | 2.008 |
| 512 | 482 | 36 | 1ayx−1gai | 1ayx_2 | 0.947 | 2.388 |
| 512 | 482 | 36 | 1ayx−1gai | 1ayx_3 | 0.949 | 2.862 |
| 512 | 482 | 36 | 1ayx−1gai | 1ayx_4 | 0.949 | 3.054 |
| 512 | 482 | 36 | 1ayx−1gai | 1ayx_5 | 0.930 | 3.146 |
| 512 | 482 | 36 | 1ayx−1gai | 1ayx_6 | 0.928 | 3.231 |
| 512 | 482 | 36 | 1ayx−1gai | 1ayx_7 | 0.884 | 3.151 |
| 512 | 482 | 36 | 1ayx−1gai | 1ayx_8 | 0.921 | 3.052 |
| 512 | 482 | 36 | 1ayx−1gai | 1ayx_9 | 0.905 | 3.112 |
| 512 | 482 | 36 | 1ayx−1gai | 1ayx_10 | 0.895 | 3.294 |
| 513 | 142 | 36 | 1apy−1ayy | 1apy_1 | 0.978 | 1.261 |
| 513 | 142 | 36 | 1apy−1ayy | 1apy_2 | 0.978 | 1.334 |
| 513 | 142 | 36 | 1apy−1ayy | 1apy_3 | 0.978 | 1.302 |
| 513 | 142 | 36 | 1apy−1ayy | 1apy_4 | 0.978 | 1.344 |
| 513 | 142 | 36 | 1apy−1ayy | 1apy_5 | 0.978 | 1.125 |
| 513 | 142 | 36 | 1apy−1ayy | 1apy_6 | 0.985 | 1.345 |
| 513 | 142 | 36 | 1apy−1ayy | 1apy_7 | 0.985 | 1.539 |
| 513 | 142 | 36 | 1apy−1ayy | 1apy_8 | 0.978 | 1.341 |
| 513 | 142 | 36 | 1apy−1ayy | 1apy_9 | 0.985 | 1.519 |
| 513 | 142 | 36 | 1apy−1ayy | 1apy_10 | 0.978 | 1.451 |
| 513 | 142 | 36 | 1ayy−1apy | 1ayy_1 | 0.948 | 1.892 |
| 513 | 142 | 36 | 1ayy−1apy | 1ayy_2 | 0.911 | 1.985 |
| 513 | 142 | 36 | 1ayy−1apy | 1ayy_3 | 0.926 | 1.974 |
| 513 | 142 | 36 | 1ayy−1apy | 1ayy_4 | 0.911 | 2.059 |
| 513 | 142 | 36 | 1ayy−1apy | 1ayy_5 | 0.919 | 2.186 |
| 513 | 142 | 36 | 1ayy−1apy | 1ayy_6 | 0.889 | 2.380 |
| 513 | 142 | 36 | 1ayy−1apy | 1ayy_7 | 0.933 | 2.249 |
| 513 | 142 | 36 | 1ayy−1apy | 1ayy_8 | 0.948 | 2.248 |
| 513 | 142 | 36 | 1ayy−1apy | 1ayy_9 | 0.993 | 2.073 |
| 513 | 142 | 36 | 1ayy−1apy | 1ayy_10 | 0.970 | 1.984 |
| 516 | 331 | 36 | 1a99−1pot | 1a99_1 | 0.984 | 1.655 |
| 516 | 331 | 36 | 1pot−1a99 | 1pot_1 | 0.981 | 2.412 |
| 516 | 331 | 36 | 1pot−1a99 | 1pot_2 | 0.956 | 3.152 |
| 516 | 331 | 36 | 1pot−1a99 | 1pot_3 | 0.925 | 3.211 |
| 516 | 331 | 36 | 1pot−1a99 | 1pot_4 | 0.960 | 3.242 |
| 516 | 331 | 36 | 1pot−1a99 | 1pot_5 | 0.966 | 2.938 |
| 516 | 331 | 36 | 1pot−1a99 | 1pot_6 | 0.950 | 3.106 |
| 516 | 331 | 36 | 1pot−1a99 | 1pot_7 | 0.913 | 3.018 |
| 516 | 331 | 36 | 1pot−1a99 | 1pot_8 | 0.903 | 3.171 |
| 516 | 331 | 36 | 1pot−1a99 | 1pot_9 | 0.885 | 3.252 |
| 516 | 331 | 36 | 1pot−1a99 | 1pot_10 | 0.894 | 3.124 |
| 517 | 253 | 36 | 1plq−1axc | 1plq_1 | 0.980 | 2.503 |
| 517 | 253 | 36 | 1plq−1axc | 1plq_2 | 0.972 | 2.939 |
| 517 | 253 | 36 | 1plq−1axc | 1plq_3 | 0.972 | 2.860 |
| 517 | 253 | 36 | 1plq−1axc | 1plq_4 | 0.972 | 3.442 |
| 517 | 253 | 36 | 1plq−1axc | 1plq_5 | 0.911 | 3.192 |
| 517 | 253 | 36 | 1plq−1axc | 1plq_6 | 0.984 | 3.198 |
| 517 | 253 | 36 | 1plq−1axc | 1plq_7 | 0.964 | 3.493 |
| 517 | 253 | 36 | 1plq−1axc | 1plq_8 | 0.964 | 3.331 |
| 517 | 253 | 36 | 1plq−1axc | 1plq_9 | 0.968 | 3.581 |
| 517 | 253 | 36 | 1plq−1axc | 1plq_10 | 0.992 | 3.158 |
| 518 | 102 | 36 | 1jiw−1smp | 1jiw_1 | 1.000 | 1.366 |
| 518 | 102 | 36 | 1jiw−1smp | 1jiw_2 | 1.000 | 1.809 |
| 518 | 102 | 36 | 1jiw−1smp | 1jiw_3 | 1.000 | 1.646 |
| 518 | 102 | 36 | 1jiw−1smp | 1jiw_4 | 0.990 | 1.863 |
| 519 | 215 | 36 | 1a2z−1aug | 1a2z_1 | 0.995 | 1.424 |
| 519 | 215 | 36 | 1a2z−1aug | 1a2z_2 | 0.990 | 1.513 |
| 519 | 215 | 36 | 1a2z−1aug | 1a2z_3 | 0.995 | 1.455 |
| 524 | 93 | 36 | 1bqv−1lky | 1bqv_1 | 0.932 | 5.358 |
| 524 | 93 | 36 | 1bqv−1lky | 1bqv_2 | 0.932 | 4.866 |
| 524 | 93 | 36 | 1bqv−1lky | 1bqv_3 | 0.797 | 5.335 |
| 524 | 93 | 36 | 1bqv−1lky | 1bqv_4 | 0.797 | 5.157 |
| 524 | 93 | 36 | 1bqv−1lky | 1bqv_5 | 0.865 | 5.774 |
| 524 | 93 | 36 | 1bqv−1lky | 1bqv_6 | 0.878 | 7.045 |
| 524 | 93 | 36 | 1bqv−1lky | 1bqv_7 | 0.865 | 7.592 |
| 524 | 93 | 36 | 1bqv−1lky | 1bqv_8 | 0.878 | 9.178 |
| 524 | 93 | 36 | 1bqv−1lky | 1bqv_9 | 0.878 | 8.758 |
| 524 | 93 | 36 | 1bqv−1lky | 1bqv_10 | 0.811 | 8.349 |
| 527 | 438 | 35 | 1a8d−3bta | 1a8d_1 | 0.969 | 2.378 |
| 527 | 438 | 35 | 1a8d−3bta | 1a8d_2 | 0.964 | 2.765 |
| 527 | 438 | 35 | 1a8d−3bta | 1a8d_3 | 0.944 | 3.349 |
| 527 | 438 | 35 | 1a8d−3bta | 1a8d_4 | 0.954 | 3.574 |
| 527 | 438 | 35 | 1a8d−3bta | 1a8d_5 | 0.939 | 3.435 |
| 527 | 438 | 35 | 1a8d−3bta | 1a8d_6 | 0.947 | 4.165 |
| 527 | 438 | 35 | 1a8d−3bta | 1a8d_7 | 0.954 | 4.228 |
| 527 | 438 | 35 | 1a8d−3bta | 1a8d_8 | 0.930 | 4.191 |
| 527 | 438 | 35 | 1a8d−3bta | 1a8d_9 | 0.920 | 4.061 |
| 531 | 580 | 35 | 1dlc−1ciy | 1dlc_1 | 0.948 | 2.170 |
| 531 | 580 | 35 | 1dlc−1ciy | 1dlc_2 | 0.939 | 2.524 |
| 531 | 580 | 35 | 1dlc−1ciy | 1dlc_3 | 0.939 | 2.497 |
| 531 | 580 | 35 | 1ciy−1dlc | 1ciy_1 | 0.977 | 1.813 |
| 531 | 580 | 35 | 1ciy−1dlc | 1ciy_2 | 0.950 | 2.486 |
| 531 | 580 | 35 | 1ciy−1dlc | 1ciy_3 | 0.962 | 2.353 |
| 534 | 61 | 35 | 1gp2−1tbg | 1gp2_1 | 0.796 | 2.626 |
| 534 | 61 | 35 | 1gp2−1tbg | 1gp2_2 | 0.704 | 3.870 |
| 534 | 61 | 35 | 1gp2−1tbg | 1gp2_3 | 0.537 | 5.155 |
| 534 | 61 | 35 | 1gp2−1tbg | 1gp2_4 | 0.833 | 4.208 |
| 534 | 61 | 35 | 1gp2−1tbg | 1gp2_5 | 0.611 | 4.834 |
| 536 | 165 | 35 | 1itf−1au1 | 1itf_1 | 0.801 | 3.046 |
| 536 | 165 | 35 | 1itf−1au1 | 1itf_2 | 0.826 | 3.192 |
| 536 | 165 | 35 | 1itf−1au1 | 1itf_3 | 0.795 | 3.416 |
| 536 | 165 | 35 | 1itf−1au1 | 1itf_4 | 0.820 | 4.090 |
| 536 | 165 | 35 | 1itf−1au1 | 1itf_5 | 0.764 | 4.182 |
| 536 | 165 | 35 | 1itf−1au1 | 1itf_6 | 0.634 | 4.544 |
| 536 | 165 | 35 | 1itf−1au1 | 1itf_7 | 0.702 | 4.651 |
| 536 | 165 | 35 | 1itf−1au1 | 1itf_8 | 0.776 | 4.726 |
| 536 | 165 | 35 | 1itf−1au1 | 1itf_9 | 0.745 | 4.854 |
| 536 | 165 | 35 | 1itf−1au1 | 1itf_10 | 0.727 | 4.892 |
| 541 | 291 | 35 | 1fts−1ffh | 1fts_1 | 0.953 | 1.888 |
| 541 | 291 | 35 | 1fts−1ffh | 1fts_2 | 0.945 | 2.325 |
| 541 | 291 | 35 | 1fts−1ffh | 1fts_3 | 0.985 | 2.529 |
| 541 | 291 | 35 | 1fts−1ffh | 1fts_4 | 0.894 | 2.868 |
| 541 | 291 | 35 | 1fts−1ffh | 1fts_5 | 0.956 | 2.759 |
| 544 | 125 | 35 | 2mbr−1hsk | 2mbr_1 | 0.811 | 1.967 |
| 544 | 125 | 35 | 2mbr−1hsk | 2mbr_2 | 0.792 | 2.333 |
| 544 | 125 | 35 | 2mbr−1hsk | 2mbr_3 | 0.783 | 2.375 |
| 544 | 125 | 35 | 2mbr−1hsk | 2mbr_4 | 0.981 | 2.537 |
| 544 | 125 | 35 | 2mbr−1hsk | 2mbr_5 | 0.774 | 3.174 |
| 544 | 125 | 35 | 1hsk−2mbr | 1hsk_1 | 0.981 | 2.100 |
| 544 | 125 | 35 | 1hsk−2mbr | 1hsk_2 | 0.783 | 2.459 |
| 544 | 125 | 35 | 1hsk−2mbr | 1hsk_3 | 0.925 | 2.381 |
| 544 | 125 | 35 | 1hsk−2mbr | 1hsk_4 | 0.943 | 2.499 |
| 544 | 125 | 35 | 1hsk−2mbr | 1hsk_5 | 0.943 | 2.430 |
| 544 | 125 | 35 | 1hsk−2mbr | 1hsk_6 | 0.934 | 2.358 |
| 544 | 125 | 35 | 1hsk−2mbr | 1hsk_7 | 0.972 | 2.433 |
| 544 | 125 | 35 | 1hsk−2mbr | 1hsk_8 | 0.981 | 2.326 |
| 544 | 125 | 35 | 1hsk−2mbr | 1hsk_9 | 0.925 | 2.463 |
| 544 | 125 | 35 | 1hsk−2mbr | 1hsk_10 | 0.868 | 2.618 |
| 546 | 149 | 35 | 1elk−1dvp | 1elk_1 | 1.000 | 1.931 |
| 547 | 575 | 35 | 1qi9−1qhb | 1qi9_1 | 0.896 | 2.070 |
| 547 | 575 | 35 | 1qi9−1qhb | 1qi9_2 | 0.880 | 2.114 |
| 547 | 575 | 35 | 1qi9−1qhb | 1qi9_3 | 0.888 | 2.349 |
| 553 | 124 | 34 | 8cho−1opy | 8cho_1 | 0.950 | 2.255 |
| 553 | 124 | 34 | 8cho−1opy | 8cho_2 | 1.000 | 2.191 |
| 553 | 124 | 34 | 8cho−1opy | 8cho_3 | 1.000 | 2.139 |
| 553 | 124 | 34 | 8cho−1opy | 8cho_4 | 0.942 | 2.368 |
| 553 | 124 | 34 | 8cho−1opy | 8cho_5 | 0.917 | 2.456 |
| 553 | 124 | 34 | 8cho−1opy | 8cho_6 | 0.992 | 2.250 |
| 553 | 124 | 34 | 8cho−1opy | 8cho_7 | 0.959 | 2.379 |
| 553 | 124 | 34 | 8cho−1opy | 8cho_8 | 0.959 | 2.650 |
| 553 | 124 | 34 | 8cho−1opy | 8cho_9 | 1.000 | 3.100 |
| 553 | 124 | 34 | 8cho−1opy | 8cho_10 | 0.950 | 3.331 |
| 554 | 161 | 34 | 1vhr−1mkp | 1vhr_1 | 0.908 | 2.150 |
| 554 | 161 | 34 | 1vhr−1mkp | 1vhr_2 | 0.901 | 2.398 |
| 554 | 161 | 34 | 1vhr−1mkp | 1vhr_3 | 0.908 | 2.350 |
| 557 | 399 | 34 | 1qba−1hp5 | 1qba_1 | 0.933 | 1.782 |
| 557 | 399 | 34 | 1qba−1hp5 | 1qba_2 | 0.951 | 1.735 |
| 557 | 399 | 34 | 1qba−1hp5 | 1qba_3 | 0.964 | 1.748 |
| 557 | 399 | 34 | 1qba−1hp5 | 1qba_4 | 0.954 | 1.967 |
| 557 | 399 | 34 | 1hp5−1qba | 1hp5_1 | 0.979 | 1.651 |
| 557 | 399 | 34 | 1hp5−1qba | 1hp5_2 | 0.957 | 1.701 |
| 557 | 399 | 34 | 1hp5−1qba | 1hp5_3 | 0.930 | 1.893 |
| 557 | 399 | 34 | 1hp5−1qba | 1hp5_4 | 0.930 | 2.032 |
| 557 | 399 | 34 | 1hp5−1qba | 1hp5_5 | 0.933 | 2.015 |
| 557 | 399 | 34 | 1hp5−1qba | 1hp5_6 | 0.936 | 1.900 |
| 560 | 275 | 34 | 1gx3−1e2t | 1gx3_1 | 0.985 | 2.621 |
| 562 | 330 | 34 | 1gq8−1qjv | 1gq8_1 | 0.935 | 2.184 |
| 562 | 330 | 34 | 1gq8−1qjv | 1gq8_2 | 0.925 | 2.260 |
| 562 | 330 | 34 | 1gq8−1qjv | 1gq8_3 | 0.938 | 2.337 |
| 562 | 330 | 34 | 1gq8−1qjv | 1gq8_4 | 0.966 | 2.232 |
| 562 | 330 | 34 | 1gq8−1qjv | 1gq8_5 | 0.928 | 2.194 |
| 562 | 330 | 34 | 1gq8−1qjv | 1gq8_6 | 0.973 | 2.360 |
| 562 | 330 | 34 | 1gq8−1qjv | 1gq8_7 | 0.945 | 2.387 |
| 562 | 330 | 34 | 1gq8−1qjv | 1gq8_8 | 0.969 | 2.568 |
| 562 | 330 | 34 | 1gq8−1qjv | 1gq8_9 | 0.990 | 2.488 |
| 562 | 330 | 34 | 1gq8−1qjv | 1gq8_10 | 0.932 | 3.304 |
| 562 | 330 | 34 | 1qjv−1gq8 | 1qjv_1 | 0.952 | 2.163 |
| 562 | 330 | 34 | 1qjv−1gq8 | 1qjv_2 | 0.949 | 2.767 |
| 569 | 101 | 34 | 1opc−1qqi | 1opc_1 | 1.000 | 2.132 |
| 569 | 101 | 34 | 1opc−1qqi | 1opc_2 | 1.000 | 2.344 |
| 569 | 101 | 34 | 1opc−1qqi | 1opc_3 | 1.000 | 2.951 |
| 569 | 101 | 34 | 1opc−1qqi | 1opc_4 | 1.000 | 2.628 |
| 569 | 101 | 34 | 1opc−1qqi | 1opc_5 | 1.000 | 3.289 |
| 569 | 101 | 34 | 1opc−1qqi | 1opc_6 | 1.000 | 2.604 |
| 569 | 101 | 34 | 1opc−1qqi | 1opc_7 | 1.000 | 3.165 |
| 569 | 101 | 34 | 1opc−1qqi | 1opc_8 | 0.990 | 3.118 |
| 569 | 101 | 34 | 1opc−1qqi | 1opc_9 | 1.000 | 3.176 |
| 569 | 101 | 34 | 1opc−1qqi | 1opc_10 | 0.990 | 3.471 |
| 569 | 101 | 34 | 1qqi−1opc | 1qqi_1 | 0.990 | 2.179 |
| 569 | 101 | 34 | 1qqi−1opc | 1qqi_2 | 0.990 | 2.753 |

| n | length | %ID | refA−refB | altA | SP | RMSD |
|---|---|---|---|---|---|---|
| 569 | 101 | 34 | 1qqi−1opc | 1qqi_3 | 0.990 | 2.824 |
| 569 | 101 | 34 | 1qqi−1opc | 1qqi_4 | 0.990 | 2.622 |
| 569 | 101 | 34 | 1qqi−1opc | 1qqi_5 | 0.980 | 2.768 |
| 569 | 101 | 34 | 1qqi−1opc | 1qqi_6 | 0.939 | 3.050 |
| 569 | 101 | 34 | 1qqi−1opc | 1qqi_7 | 0.949 | 3.117 |
| 569 | 101 | 34 | 1qqi−1opc | 1qqi_8 | 1.000 | 3.291 |
| 569 | 101 | 34 | 1qqi−1opc | 1qqi_9 | 0.939 | 3.409 |
| 569 | 101 | 34 | 1qqi−1opc | 1qqi_10 | 0.990 | 3.394 |
| 571 | 350 | 34 | 1j93−1uro | 1j93_1 | 0.891 | 2.152 |
| 571 | 350 | 34 | 1j93−1uro | 1j93_2 | 0.915 | 2.590 |
| 571 | 350 | 34 | 1j93−1uro | 1j93_3 | 0.938 | 2.613 |
| 571 | 350 | 34 | 1j93−1uro | 1j93_4 | 0.921 | 2.530 |
| 571 | 350 | 34 | 1j93−1uro | 1j93_5 | 0.886 | 2.625 |
| 571 | 350 | 34 | 1j93−1uro | 1j93_6 | 0.859 | 2.842 |
| 571 | 350 | 34 | 1j93−1uro | 1j93_7 | 0.918 | 2.719 |
| 571 | 350 | 34 | 1j93−1uro | 1j93_8 | 0.921 | 3.134 |
| 571 | 350 | 34 | 1uro−1j93 | 1uro_1 | 0.959 | 1.931 |
| 571 | 350 | 34 | 1uro−1j93 | 1uro_2 | 0.974 | 1.905 |
| 571 | 350 | 34 | 1uro−1j93 | 1uro_3 | 0.959 | 2.012 |
| 571 | 350 | 34 | 1uro−1j93 | 1uro_4 | 0.979 | 2.017 |
| 571 | 350 | 34 | 1uro−1j93 | 1uro_5 | 0.979 | 2.012 |
| 571 | 350 | 34 | 1uro−1j93 | 1uro_6 | 0.979 | 2.135 |
| 573 | 475 | 33 | 2pgd−1pgj | 2pgd_1 | 0.886 | 4.161 |
| 573 | 475 | 33 | 2pgd−1pgj | 2pgd_2 | 0.845 | 5.101 |
| 573 | 475 | 33 | 2pgd−1pgj | 2pgd_3 | 0.895 | 4.735 |
| 573 | 475 | 33 | 2pgd−1pgj | 2pgd_4 | 0.910 | 4.915 |
| 573 | 475 | 33 | 2pgd−1pgj | 2pgd_5 | 0.811 | 5.854 |
| 573 | 475 | 33 | 2pgd−1pgj | 2pgd_6 | 0.815 | 6.507 |
| 573 | 475 | 33 | 2pgd−1pgj | 2pgd_7 | 0.818 | 6.786 |
| 574 | 250 | 33 | 1isi−1lbe | 1isi_1 | 0.947 | 2.007 |
| 574 | 250 | 33 | 1isi−1lbe | 1isi_2 | 0.930 | 1.795 |
| 574 | 250 | 33 | 1isi−1lbe | 1isi_3 | 0.914 | 2.233 |
| 574 | 250 | 33 | 1isi−1lbe | 1isi_4 | 0.922 | 2.145 |
| 574 | 250 | 33 | 1isi−1lbe | 1isi_5 | 0.922 | 2.202 |
| 574 | 250 | 33 | 1lbe−1isi | 1lbe_1 | 0.926 | 2.489 |
| 574 | 250 | 33 | 1lbe−1isi | 1lbe_2 | 0.959 | 2.764 |
| 574 | 250 | 33 | 1lbe−1isi | 1lbe_3 | 0.897 | 3.663 |
| 580 | 114 | 33 | 1iow−1ehi | 1iow_1 | 0.989 | 1.134 |
| 580 | 114 | 33 | 1iow−1ehi | 1iow_2 | 1.000 | 1.831 |
| 580 | 114 | 33 | 1iow−1ehi | 1iow_3 | 0.947 | 2.050 |
| 580 | 114 | 33 | 1iow−1ehi | 1iow_4 | 0.989 | 2.188 |
| 580 | 114 | 33 | 1iow−1ehi | 1iow_5 | 0.989 | 2.072 |
| 581 | 252 | 33 | 1skf−1hd8 | 1skf_1 | 0.983 | 2.013 |
| 581 | 252 | 33 | 1skf−1hd8 | 1skf_2 | 0.961 | 2.402 |
| 581 | 252 | 33 | 1skf−1hd8 | 1skf_3 | 0.939 | 2.939 |
| 581 | 252 | 33 | 1skf−1hd8 | 1skf_4 | 0.978 | 2.927 |
| 581 | 252 | 33 | 1skf−1hd8 | 1skf_5 | 0.974 | 2.899 |
| 581 | 252 | 33 | 1skf−1hd8 | 1skf_6 | 0.944 | 3.519 |
| 581 | 252 | 33 | 1skf−1hd8 | 1skf_7 | 0.948 | 3.482 |
| 581 | 252 | 33 | 1skf−1hd8 | 1skf_8 | 0.948 | 3.914 |
| 581 | 252 | 33 | 1skf−1hd8 | 1skf_9 | 0.952 | 3.718 |
| 581 | 252 | 33 | 1skf−1hd8 | 1skf_10 | 0.944 | 3.650 |
| 584 | 224 | 33 | 1g61−1g62 | 1g61_1 | 0.986 | 1.393 |
| 584 | 224 | 33 | 1g61−1g62 | 1g61_2 | 0.986 | 1.388 |
| 584 | 224 | 33 | 1g61−1g62 | 1g61_3 | 0.986 | 1.603 |
| 584 | 224 | 33 | 1g61−1g62 | 1g61_4 | 0.991 | 1.692 |
| 584 | 224 | 33 | 1g61−1g62 | 1g61_5 | 0.991 | 1.702 |
| 584 | 224 | 33 | 1g61−1g62 | 1g61_6 | 0.973 | 1.633 |
| 584 | 224 | 33 | 1g61−1g62 | 1g61_7 | 0.968 | 1.803 |
| 587 | 146 | 33 | 1euu−1gof | 1euu_1 | 1.000 | 1.712 |
| 587 | 146 | 33 | 1euu−1gof | 1euu_2 | 1.000 | 1.691 |
| 587 | 146 | 33 | 1euu−1gof | 1euu_3 | 0.986 | 1.812 |
| 587 | 146 | 33 | 1euu−1gof | 1euu_4 | 0.964 | 1.968 |
| 587 | 146 | 33 | 1euu−1gof | 1euu_5 | 0.964 | 1.888 |
| 587 | 146 | 33 | 1gof−1euu | 1gof_1 | 1.000 | 1.359 |
| 587 | 146 | 33 | 1gof−1euu | 1gof_2 | 1.000 | 1.978 |
| 587 | 146 | 33 | 1gof−1euu | 1gof_3 | 0.964 | 1.828 |
| 587 | 146 | 33 | 1gof−1euu | 1gof_4 | 1.000 | 2.371 |
| 588 | 205 | 33 | 1bf8−1qpx | 1bf8_1 | 0.637 | 4.564 |
| 588 | 205 | 33 | 1bf8−1qpx | 1bf8_2 | 0.826 | 5.559 |
| 588 | 205 | 33 | 1bf8−1qpx | 1bf8_3 | 0.611 | 6.021 |
| 588 | 205 | 33 | 1bf8−1qpx | 1bf8_4 | 0.574 | 6.675 |
| 588 | 205 | 33 | 1bf8−1qpx | 1bf8_5 | 0.526 | 6.833 |
| 588 | 205 | 33 | 1bf8−1qpx | 1bf8_6 | 0.516 | 7.825 |
| 588 | 205 | 33 | 1bf8−1qpx | 1bf8_7 | 0.563 | 7.786 |
| 588 | 205 | 33 | 1bf8−1qpx | 1bf8_8 | 0.584 | 7.669 |
| 588 | 205 | 33 | 1bf8−1qpx | 1bf8_9 | 0.521 | 8.428 |
| 588 | 205 | 33 | 1bf8−1qpx | 1bf8_10 | 0.632 | 7.945 |
| 591 | 52 | 33 | 1kzu−1lgh | 1kzu_1 | 0.917 | 3.834 |
| 591 | 52 | 33 | 1kzu−1lgh | 1kzu_2 | 0.917 | 4.392 |
| 591 | 52 | 33 | 1kzu−1lgh | 1kzu_3 | 0.896 | 5.576 |
| 591 | 52 | 33 | 1lgh−1kzu | 1lgh_1 | 0.750 | 5.306 |
| 593 | 135 | 33 | 1eyv−1ey1 | 1eyv_1 | 0.817 | 2.253 |
| 593 | 135 | 33 | 1eyv−1ey1 | 1eyv_2 | 0.849 | 2.465 |
| 593 | 135 | 33 | 1eyv−1ey1 | 1eyv_3 | 0.738 | 2.619 |
| 593 | 135 | 33 | 1eyv−1ey1 | 1eyv_4 | 0.802 | 2.729 |
| 593 | 135 | 33 | 1eyv−1ey1 | 1eyv_5 | 0.778 | 2.698 |
| 593 | 135 | 33 | 1eyv−1ey1 | 1eyv_6 | 0.706 | 2.829 |
| 593 | 135 | 33 | 1eyv−1ey1 | 1eyv_7 | 0.683 | 3.117 |
| 593 | 135 | 33 | 1eyv−1ey1 | 1eyv_8 | 0.714 | 3.175 |
| 593 | 135 | 33 | 1eyv−1ey1 | 1eyv_9 | 0.603 | 3.235 |
| 593 | 135 | 33 | 1eyv−1ey1 | 1eyv_10 | 0.786 | 3.234 |
| 594 | 102 | 33 | 1f39−1umu | 1f39_1 | 0.953 | 1.657 |
| 594 | 102 | 33 | 1f39−1umu | 1f39_2 | 0.942 | 1.793 |
| 594 | 102 | 33 | 1f39−1umu | 1f39_3 | 0.942 | 2.175 |
| 594 | 102 | 33 | 1f39−1umu | 1f39_4 | 0.965 | 2.414 |
| 594 | 102 | 33 | 1f39−1umu | 1f39_5 | 0.977 | 2.693 |
| 594 | 102 | 33 | 1f39−1umu | 1f39_6 | 0.965 | 2.890 |
| 594 | 102 | 33 | 1f39−1umu | 1f39_7 | 0.977 | 2.862 |
| 594 | 102 | 33 | 1f39−1umu | 1f39_8 | 0.988 | 3.180 |
| 594 | 102 | 33 | 1f39−1umu | 1f39_9 | 0.930 | 2.729 |
| 594 | 102 | 33 | 1f39−1umu | 1f39_10 | 0.977 | 2.860 |
| 595 | 223 | 33 | 1fj2−1auo | 1fj2_1 | 0.958 | 1.413 |
| 595 | 223 | 33 | 1fj2−1auo | 1fj2_2 | 0.991 | 1.673 |
| 595 | 223 | 33 | 1fj2−1auo | 1fj2_3 | 0.986 | 1.690 |
| 595 | 223 | 33 | 1fj2−1auo | 1fj2_4 | 0.991 | 1.866 |
| 595 | 223 | 33 | 1fj2−1auo | 1fj2_5 | 0.967 | 1.816 |
| 595 | 223 | 33 | 1fj2−1auo | 1fj2_6 | 0.972 | 2.039 |
| 595 | 223 | 33 | 1fj2−1auo | 1fj2_7 | 0.977 | 2.100 |
| 595 | 223 | 33 | 1fj2−1auo | 1fj2_8 | 0.977 | 2.060 |
| 595 | 223 | 33 | 1fj2−1auo | 1fj2_9 | 0.944 | 1.973 |
| 595 | 223 | 33 | 1fj2−1auo | 1fj2_10 | 0.926 | 2.175 |
| 595 | 223 | 33 | 1auo−1fj2 | 1auo_1 | 0.977 | 1.459 |
| 595 | 223 | 33 | 1auo−1fj2 | 1auo_2 | 0.972 | 1.705 |
| 595 | 223 | 33 | 1auo−1fj2 | 1auo_3 | 0.977 | 1.800 |
| 595 | 223 | 33 | 1auo−1fj2 | 1auo_4 | 0.953 | 2.219 |
| 595 | 223 | 33 | 1auo−1fj2 | 1auo_5 | 0.870 | 2.756 |
| 595 | 223 | 33 | 1auo−1fj2 | 1auo_6 | 0.972 | 2.660 |
| 595 | 223 | 33 | 1auo−1fj2 | 1auo_7 | 0.967 | 2.422 |
| 595 | 223 | 33 | 1auo−1fj2 | 1auo_8 | 0.986 | 2.477 |
| 595 | 223 | 33 | 1auo−1fj2 | 1auo_9 | 0.963 | 2.600 |
| 595 | 223 | 33 | 1auo−1fj2 | 1auo_10 | 0.902 | 2.493 |
| 598 | 127 | 33 | 1rie−1rfs | 1rie_1 | 0.990 | 1.813 |
| 598 | 127 | 33 | 1rie−1rfs | 1rie_2 | 0.990 | 2.178 |
| 598 | 127 | 33 | 1rie−1rfs | 1rie_3 | 1.000 | 2.914 |
| 598 | 127 | 33 | 1rie−1rfs | 1rie_4 | 0.876 | 2.954 |
| 598 | 127 | 33 | 1rie−1rfs | 1rie_5 | 0.969 | 2.973 |
| 598 | 127 | 33 | 1rie−1rfs | 1rie_6 | 0.979 | 2.707 |
| 598 | 127 | 33 | 1rie−1rfs | 1rie_7 | 0.856 | 2.927 |
| 598 | 127 | 33 | 1rie−1rfs | 1rie_8 | 0.990 | 2.863 |
| 598 | 127 | 33 | 1rie−1rfs | 1rie_9 | 1.000 | 2.821 |
| 598 | 127 | 33 | 1rie−1rfs | 1rie_10 | 0.938 | 3.089 |
| 598 | 127 | 33 | 1rfs−1rie | 1rfs_1 | 0.835 | 1.766 |
| 598 | 127 | 33 | 1rfs−1rie | 1rfs_2 | 0.845 | 2.104 |
| 598 | 127 | 33 | 1rfs−1rie | 1rfs_3 | 0.887 | 2.110 |
| 598 | 127 | 33 | 1rfs−1rie | 1rfs_4 | 0.794 | 2.090 |
| 598 | 127 | 33 | 1rfs−1rie | 1rfs_5 | 0.907 | 2.151 |
| 598 | 127 | 33 | 1rfs−1rie | 1rfs_6 | 0.866 | 2.285 |
| 598 | 127 | 33 | 1rfs−1rie | 1rfs_7 | 0.835 | 2.278 |
| 598 | 127 | 33 | 1rfs−1rie | 1rfs_8 | 0.866 | 2.343 |
| 598 | 127 | 33 | 1rfs−1rie | 1rfs_9 | 0.876 | 2.111 |
| 598 | 127 | 33 | 1rfs−1rie | 1rfs_10 | 0.845 | 2.607 |
| 602 | 526 | 33 | 1l0v−1chu | 1l0v_1 | 0.946 | 2.358 |
| 605 | 464 | 32 | 1ed9−1ew2 | 1ed9_1 | 0.950 | 1.836 |
| 605 | 464 | 32 | 1ed9−1ew2 | 1ed9_2 | 0.940 | 2.081 |
| 607 | 121 | 32 | 1rav−1pts | 1rav_1 | 0.922 | 2.707 |
| 607 | 121 | 32 | 1rav−1pts | 1rav_2 | 0.878 | 3.141 |
| 607 | 121 | 32 | 1rav−1pts | 1rav_3 | 0.896 | 3.179 |
| 607 | 121 | 32 | 1rav−1pts | 1rav_4 | 0.878 | 2.985 |
| 607 | 121 | 32 | 1rav−1pts | 1rav_5 | 0.930 | 2.959 |
| 607 | 121 | 32 | 1rav−1pts | 1rav_6 | 0.878 | 2.838 |
| 607 | 121 | 32 | 1rav−1pts | 1rav_7 | 0.852 | 3.308 |
| 607 | 121 | 32 | 1rav−1pts | 1rav_8 | 0.913 | 3.320 |
| 612 | 40 | 32 | 2pdd−1bbl | 2pdd_1 | 0.865 | 2.626 |
| 612 | 40 | 32 | 2pdd−1bbl | 2pdd_2 | 0.784 | 2.180 |
| 612 | 40 | 32 | 2pdd−1bbl | 2pdd_3 | 0.784 | 2.402 |
| 612 | 40 | 32 | 2pdd−1bbl | 2pdd_4 | 1.000 | 4.772 |
| 612 | 40 | 32 | 2pdd−1bbl | 2pdd_5 | 1.000 | 4.659 |
| 612 | 40 | 32 | 2pdd−1bbl | 2pdd_6 | 0.784 | 4.747 |
| 612 | 40 | 32 | 2pdd−1bbl | 2pdd_7 | 0.784 | 4.926 |
| 612 | 40 | 32 | 2pdd−1bbl | 2pdd_8 | 0.784 | 5.521 |
| 612 | 40 | 32 | 2pdd−1bbl | 2pdd_9 | 0.757 | 4.868 |
| 612 | 40 | 32 | 1bbl−2pdd | 1bbl_1 | 0.622 | 3.848 |
| 612 | 40 | 32 | 1bbl−2pdd | 1bbl_2 | 0.649 | 3.597 |
| 612 | 40 | 32 | 1bbl−2pdd | 1bbl_3 | 0.703 | 3.545 |
| 612 | 40 | 32 | 1bbl−2pdd | 1bbl_4 | 0.649 | 3.994 |
| 612 | 40 | 32 | 1bbl−2pdd | 1bbl_5 | 0.486 | 4.280 |
| 612 | 40 | 32 | 1bbl−2pdd | 1bbl_6 | 0.541 | 3.912 |
| 612 | 40 | 32 | 1bbl−2pdd | 1bbl_8 | 0.730 | 4.086 |
| 612 | 40 | 32 | 1bbl−2pdd | 1bbl_9 | 0.622 | 4.398 |
| 612 | 40 | 32 | 1bbl−2pdd | 1bbl_10 | 0.432 | 4.458 |
| 613 | 85 | 32 | 2if1−1d1r | 2if1_1 | 0.923 | 2.521 |
| 613 | 85 | 32 | 2if1−1d1r | 2if1_2 | 0.949 | 2.836 |
| 613 | 85 | 32 | 2if1−1d1r | 2if1_3 | 0.885 | 2.807 |
| 613 | 85 | 32 | 2if1−1d1r | 2if1_4 | 0.885 | 2.563 |
| 613 | 85 | 32 | 2if1−1d1r | 2if1_5 | 0.744 | 2.951 |
| 614 | 82 | 32 | 1d7q−1jt8 | 1d7q_1 | 0.579 | 3.112 |
| 614 | 82 | 32 | 1d7q−1jt8 | 1d7q_2 | 0.711 | 3.303 |
| 614 | 82 | 32 | 1d7q−1jt8 | 1d7q_3 | 0.711 | 3.276 |
| 614 | 82 | 32 | 1d7q−1jt8 | 1d7q_4 | 0.645 | 3.602 |
| 614 | 82 | 32 | 1d7q−1jt8 | 1d7q_5 | 0.724 | 3.697 |
| 614 | 82 | 32 | 1d7q−1jt8 | 1d7q_6 | 0.711 | 3.735 |
| 614 | 82 | 32 | 1d7q−1jt8 | 1d7q_7 | 0.697 | 3.689 |
| 614 | 82 | 32 | 1d7q−1jt8 | 1d7q_8 | 0.803 | 3.772 |
| 614 | 82 | 32 | 1jt8−1d7q | 1jt8_1 | 0.842 | 3.458 |
| 614 | 82 | 32 | 1jt8−1d7q | 1jt8_2 | 0.750 | 3.906 |
| 614 | 82 | 32 | 1jt8−1d7q | 1jt8_3 | 0.855 | 3.692 |
| 614 | 82 | 32 | 1jt8−1d7q | 1jt8_4 | 0.816 | 3.974 |
| 614 | 82 | 32 | 1jt8−1d7q | 1jt8_5 | 0.895 | 5.192 |
| 614 | 82 | 32 | 1jt8−1d7q | 1jt8_6 | 0.868 | 7.007 |
| 614 | 82 | 32 | 1jt8−1d7q | 1jt8_7 | 0.868 | 6.561 |
| 614 | 82 | 32 | 1jt8−1d7q | 1jt8_8 | 0.816 | 6.915 |
| 614 | 82 | 32 | 1jt8−1d7q | 1jt8_9 | 0.868 | 6.651 |
| 614 | 82 | 32 | 1jt8−1d7q | 1jt8_10 | 0.868 | 6.663 |
| 617 | 486 | 32 | 1dpg−1qki | 1dpg_1 | 0.996 | 1.990 |
| 617 | 486 | 32 | 1dpg−1qki | 1dpg_2 | 0.987 | 2.090 |
| 617 | 486 | 32 | 1dpg−1qki | 1dpg_3 | 0.972 | 2.227 |
| 617 | 486 | 32 | 1dpg−1qki | 1dpg_4 | 0.949 | 2.381 |
| 619 | 195 | 32 | 2mjp−1k7k | 2mjp_1 | 0.950 | 2.161 |
| 619 | 195 | 32 | 2mjp−1k7k | 2mjp_2 | 0.944 | 2.786 |
| 619 | 195 | 32 | 2mjp−1k7k | 2mjp_3 | 0.939 | 2.415 |
| 623 | 115 | 32 | 1eo1−1o13 | 1eo1_1 | 0.856 | 3.479 |
| 623 | 115 | 32 | 1eo1−1o13 | 1eo1_2 | 0.788 | 4.513 |
| 623 | 115 | 32 | 1eo1−1o13 | 1eo1_3 | 0.827 | 5.369 |
| 623 | 115 | 32 | 1eo1−1o13 | 1eo1_4 | 0.827 | 5.031 |
| 623 | 115 | 32 | 1eo1−1o13 | 1eo1_5 | 0.779 | 5.077 |
| 623 | 115 | 32 | 1eo1−1o13 | 1eo1_6 | 0.827 | 5.549 |
| 623 | 115 | 32 | 1eo1−1o13 | 1eo1_7 | 0.788 | 5.438 |
| 623 | 115 | 32 | 1eo1−1o13 | 1eo1_8 | 0.837 | 5.661 |
| 623 | 115 | 32 | 1eo1−1o13 | 1eo1_9 | 0.788 | 5.747 |
| 623 | 115 | 32 | 1eo1−1o13 | 1eo1_10 | 0.692 | 6.166 |
| 625 | 172 | 32 | 1cfe−1qnx | 1cfe_1 | 0.962 | 1.696 |
| 625 | 172 | 32 | 1cfe−1qnx | 1cfe_2 | 0.931 | 2.671 |

n: internal numbering; length of alignment; % sequence%ID (cf. Homstrad); refA−refB: reference alignment; altA: alternative snapshot for A; SP: sum−of−pairs score of alt. alignment w/r reference 

Supplementary Material Table S2b: Details of aligned Simulation Snapshots -- The Meaning of Alignment (Pirovano, Feenstra & Heringa)

| n | length | %ID | refA-refB | altA | SP | RMSD |
|---|---|---|---|---|---|---|
| 625 | 172 | 32 | 1cfe-1qnx | 1cfe_3 | 0.924 | 2.968 |
| 625 | 172 | 32 | 1cfe-1qnx | 1cfe_4 | 0.939 | 2.857 |
| 625 | 172 | 32 | 1cfe-1qnx | 1cfe_5 | 0.878 | 3.656 |
| 625 | 172 | 32 | 1cfe-1qnx | 1cfe_6 | 0.908 | 3.075 |
| 625 | 172 | 32 | 1cfe-1qnx | 1cfe_7 | 0.832 | 3.564 |
| 625 | 172 | 32 | 1cfe-1qnx | 1cfe_8 | 0.824 | 3.350 |
| 625 | 172 | 32 | 1cfe-1qnx | 1cfe_9 | 0.756 | 4.180 |
| 625 | 172 | 32 | 1cfe-1qnx | 1cfe_10 | 0.863 | 4.170 |
| 625 | 172 | 32 | 1qnx-1cfe | 1qnx_1 | 0.992 | 1.929 |
| 625 | 172 | 32 | 1qnx-1cfe | 1qnx_2 | 1.000 | 3.220 |
| 625 | 172 | 32 | 1qnx-1cfe | 1qnx_3 | 1.000 | 3.464 |
| 625 | 172 | 32 | 1qnx-1cfe | 1qnx_4 | 0.992 | 3.788 |
| 625 | 172 | 32 | 1qnx-1cfe | 1qnx_5 | 0.916 | 3.567 |
| 625 | 172 | 32 | 1qnx-1cfe | 1qnx_6 | 0.885 | 3.637 |
| 625 | 172 | 32 | 1qnx-1cfe | 1qnx_7 | 0.954 | 3.491 |
| 625 | 172 | 32 | 1qnx-1cfe | 1qnx_8 | 0.885 | 3.996 |
| 625 | 172 | 32 | 1qnx-1cfe | 1qnx_9 | 0.847 | 3.808 |
| 625 | 172 | 32 | 1qnx-1cfe | 1qnx_10 | 0.885 | 3.257 |
| 628 | 191 | 32 | 1ile-1qu2 | 1ile_1 | 0.812 | 1.979 |
| 628 | 191 | 32 | 1ile-1qu2 | 1ile_2 | 0.829 | 2.299 |
| 628 | 191 | 32 | 1qu2-1ile | 1qu2_1 | 0.773 | 4.184 |
| 629 | 198 | 32 | 1vol-1ais | 1vol_1 | 0.953 | 2.689 |
| 629 | 198 | 32 | 1vol-1ais | 1vol_2 | 0.932 | 3.756 |
| 629 | 198 | 32 | 1vol-1ais | 1vol_3 | 0.942 | 4.131 |
| 629 | 198 | 32 | 1vol-1ais | 1vol_4 | 0.953 | 4.050 |
| 630 | 170 | 31 | 1jhj-1gqp | 1jhj_1 | 1.000 | 1.510 |
| 630 | 170 | 31 | 1jhj-1gqp | 1jhj_2 | 0.974 | 1.845 |
| 630 | 170 | 31 | 1jhj-1gqp | 1jhj_3 | 1.000 | 1.693 |
| 630 | 170 | 31 | 1jhj-1gqp | 1jhj_4 | 1.000 | 1.564 |
| 630 | 170 | 31 | 1jhj-1gqp | 1jhj_5 | 1.000 | 1.519 |
| 630 | 170 | 31 | 1jhj-1gqp | 1jhj_6 | 0.929 | 2.574 |
| 630 | 170 | 31 | 1jhj-1gqp | 1jhj_7 | 0.961 | 2.878 |
| 630 | 170 | 31 | 1jhj-1gqp | 1jhj_8 | 0.929 | 3.296 |
| 630 | 170 | 31 | 1jhj-1gqp | 1jhj_9 | 0.929 | 3.159 |
| 630 | 170 | 31 | 1jhj-1gqp | 1jhj_10 | 0.929 | 3.203 |
| 631 | 75 | 31 | 1doq-1coo | 1doq_1 | 0.559 | 3.431 |
| 631 | 75 | 31 | 1doq-1coo | 1doq_2 | 0.544 | 4.190 |
| 631 | 75 | 31 | 1doq-1coo | 1doq_3 | 0.721 | 4.874 |
| 631 | 75 | 31 | 1doq-1coo | 1doq_4 | 0.515 | 4.525 |
| 631 | 75 | 31 | 1doq-1coo | 1doq_5 | 0.529 | 4.225 |
| 631 | 75 | 31 | 1doq-1coo | 1doq_6 | 0.765 | 4.500 |
| 631 | 75 | 31 | 1doq-1coo | 1doq_7 | 0.750 | 4.667 |
| 631 | 75 | 31 | 1doq-1coo | 1doq_8 | 0.559 | 4.714 |
| 631 | 75 | 31 | 1doq-1coo | 1doq_9 | 0.750 | 4.355 |
| 631 | 75 | 31 | 1doq-1coo | 1doq_10 | 0.500 | 4.682 |
| 631 | 75 | 31 | 1coo-1doq | 1coo_1 | 0.500 | 4.227 |
| 631 | 75 | 31 | 1coo-1doq | 1coo_2 | 0.691 | 4.549 |
| 631 | 75 | 31 | 1coo-1doq | 1coo_3 | 0.691 | 4.584 |
| 631 | 75 | 31 | 1coo-1doq | 1coo_4 | 0.706 | 3.977 |
| 631 | 75 | 31 | 1coo-1doq | 1coo_5 | 0.647 | 4.091 |
| 631 | 75 | 31 | 1coo-1doq | 1coo_6 | 0.691 | 3.912 |
| 631 | 75 | 31 | 1coo-1doq | 1coo_7 | 0.735 | 3.354 |
| 631 | 75 | 31 | 1coo-1doq | 1coo_8 | 0.647 | 4.104 |
| 631 | 75 | 31 | 1coo-1doq | 1coo_9 | 0.662 | 3.654 |
| 631 | 75 | 31 | 1coo-1doq | 1coo_10 | 0.691 | 4.137 |
| 632 | 325 | 31 | 6mht-1dct | 6mht_1 | 0.823 | 2.361 |
| 632 | 325 | 31 | 6mht-1dct | 6mht_2 | 0.857 | 2.441 |
| 632 | 325 | 31 | 6mht-1dct | 6mht_3 | 0.870 | 2.345 |
| 632 | 325 | 31 | 6mht-1dct | 6mht_4 | 0.857 | 2.832 |
| 632 | 325 | 31 | 6mht-1dct | 6mht_5 | 0.857 | 2.810 |
| 632 | 325 | 31 | 6mht-1dct | 6mht_6 | 0.883 | 2.802 |
| 632 | 325 | 31 | 6mht-1dct | 6mht_7 | 0.840 | 2.684 |
| 632 | 325 | 31 | 6mht-1dct | 6mht_8 | 0.823 | 2.787 |
| 632 | 325 | 31 | 6mht-1dct | 6mht_9 | 0.850 | 2.732 |
| 632 | 325 | 31 | 6mht-1dct | 6mht_10 | 0.847 | 2.645 |
| 632 | 325 | 31 | 1dct-6mht | 1dct_1 | 0.860 | 2.147 |
| 632 | 325 | 31 | 1dct-6mht | 1dct_2 | 0.827 | 2.153 |
| 646 | 119 | 31 | 1ipi-1hh1 | 1ipi_1 | 0.991 | 2.562 |
| 646 | 119 | 31 | 1ipi-1hh1 | 1ipi_2 | 0.963 | 2.637 |
| 646 | 119 | 31 | 1ipi-1hh1 | 1ipi_3 | 0.991 | 2.700 |
| 646 | 119 | 31 | 1ipi-1hh1 | 1ipi_4 | 0.981 | 2.772 |
| 646 | 119 | 31 | 1ipi-1hh1 | 1ipi_5 | 0.953 | 2.800 |
| 646 | 119 | 31 | 1ipi-1hh1 | 1ipi_6 | 0.981 | 2.767 |
| 646 | 119 | 31 | 1ipi-1hh1 | 1ipi_7 | 0.869 | 2.995 |
| 646 | 119 | 31 | 1ipi-1hh1 | 1ipi_8 | 0.729 | 3.925 |
| 649 | 130 | 31 | 1ahs-1bvp | 1ahs_1 | 0.960 | 1.354 |
| 649 | 130 | 31 | 1ahs-1bvp | 1ahs_2 | 0.960 | 1.239 |
| 649 | 130 | 31 | 1ahs-1bvp | 1ahs_3 | 0.960 | 1.456 |
| 649 | 130 | 31 | 1ahs-1bvp | 1ahs_4 | 0.960 | 1.820 |
| 649 | 130 | 31 | 1ahs-1bvp | 1ahs_5 | 0.960 | 1.617 |
| 649 | 130 | 31 | 1ahs-1bvp | 1ahs_6 | 0.952 | 1.779 |
| 649 | 130 | 31 | 1ahs-1bvp | 1ahs_7 | 0.952 | 1.659 |
| 649 | 130 | 31 | 1ahs-1bvp | 1ahs_8 | 0.952 | 1.870 |
| 649 | 130 | 31 | 1ahs-1bvp | 1ahs_9 | 0.992 | 1.843 |
| 649 | 130 | 31 | 1ahs-1bvp | 1ahs_10 | 0.976 | 1.898 |
| 653 | 281 | 31 | 1toa-1psz | 1toa_1 | 0.970 | 2.264 |
| 653 | 281 | 31 | 1toa-1psz | 1toa_2 | 0.970 | 2.390 |
| 653 | 281 | 31 | 1psz-1toa | 1psz_1 | 0.955 | 2.310 |
| 653 | 281 | 31 | 1psz-1toa | 1psz_2 | 0.925 | 2.856 |
| 653 | 281 | 31 | 1psz-1toa | 1psz_3 | 0.951 | 3.133 |
| 653 | 281 | 31 | 1psz-1toa | 1psz_4 | 0.951 | 3.704 |
| 653 | 281 | 31 | 1psz-1toa | 1psz_5 | 0.888 | 3.728 |
| 653 | 281 | 31 | 1psz-1toa | 1psz_6 | 0.910 | 3.868 |
| 653 | 281 | 31 | 1psz-1toa | 1psz_7 | 0.910 | 3.852 |
| 653 | 281 | 31 | 1psz-1toa | 1psz_8 | 0.907 | 4.092 |
| 653 | 281 | 31 | 1psz-1toa | 1psz_9 | 0.922 | 3.938 |
| 653 | 281 | 31 | 1psz-1toa | 1psz_10 | 0.899 | 4.036 |
| 654 | 285 | 31 | 1gym-2plc | 1gym_1 | 0.945 | 1.795 |
| 654 | 285 | 31 | 1gym-2plc | 1gym_2 | 0.968 | 1.925 |
| 654 | 285 | 31 | 1gym-2plc | 1gym_3 | 0.957 | 1.825 |
| 654 | 285 | 31 | 1gym-2plc | 1gym_4 | 0.964 | 2.316 |
| 654 | 285 | 31 | 1gym-2plc | 1gym_5 | 0.988 | 2.205 |
| 654 | 285 | 31 | 1gym-2plc | 1gym_6 | 0.984 | 2.334 |
| 654 | 285 | 31 | 1gym-2plc | 1gym_7 | 0.957 | 2.400 |
| 654 | 285 | 31 | 1gym-2plc | 1gym_8 | 0.964 | 2.238 |
| 654 | 285 | 31 | 1gym-2plc | 1gym_9 | 0.968 | 2.350 |
| 654 | 285 | 31 | 1gym-2plc | 1gym_10 | 0.957 | 2.509 |
| 654 | 285 | 31 | 2plc-1gym | 2plc_1 | 0.862 | 2.000 |
| 654 | 285 | 31 | 2plc-1gym | 2plc_2 | 0.877 | 1.795 |
| 654 | 285 | 31 | 2plc-1gym | 2plc_3 | 0.885 | 2.168 |
| 654 | 285 | 31 | 2plc-1gym | 2plc_4 | 0.862 | 2.261 |
| 654 | 285 | 31 | 2plc-1gym | 2plc_5 | 0.858 | 2.519 |
| 654 | 285 | 31 | 2plc-1gym | 2plc_6 | 0.866 | 2.738 |
| 654 | 285 | 31 | 2plc-1gym | 2plc_7 | 0.877 | 2.809 |
| 655 | 170 | 31 | 1g2a-1jym | 1g2a_1 | 0.868 | 2.037 |
| 655 | 170 | 31 | 1g2a-1jym | 1g2a_2 | 0.862 | 2.502 |
| 655 | 170 | 31 | 1g2a-1jym | 1g2a_3 | 0.843 | 2.970 |
| 655 | 170 | 31 | 1g2a-1jym | 1g2a_4 | 0.843 | 3.101 |
| 655 | 170 | 31 | 1g2a-1jym | 1g2a_5 | 0.843 | 3.111 |
| 655 | 170 | 31 | 1g2a-1jym | 1g2a_6 | 0.849 | 3.415 |
| 655 | 170 | 31 | 1g2a-1jym | 1g2a_7 | 0.818 | 3.512 |
| 655 | 170 | 31 | 1g2a-1jym | 1g2a_8 | 0.836 | 3.219 |
| 656 | 113 | 31 | 1h4x-1buz | 1h4x_1 | 0.731 | 2.688 |
| 656 | 113 | 31 | 1h4x-1buz | 1h4x_2 | 0.833 | 2.293 |
| 656 | 113 | 31 | 1h4x-1buz | 1h4x_3 | 0.815 | 2.323 |
| 656 | 113 | 31 | 1h4x-1buz | 1h4x_4 | 0.787 | 2.341 |
| 656 | 113 | 31 | 1h4x-1buz | 1h4x_5 | 0.750 | 2.725 |
| 656 | 113 | 31 | 1h4x-1buz | 1h4x_6 | 0.685 | 3.086 |
| 656 | 113 | 31 | 1buz-1h4x | 1buz_1 | 0.509 | 4.216 |
| 656 | 113 | 31 | 1buz-1h4x | 1buz_2 | 0.537 | 4.228 |
| 656 | 113 | 31 | 1buz-1h4x | 1buz_3 | 0.509 | 4.437 |
| 656 | 113 | 31 | 1buz-1h4x | 1buz_4 | 0.463 | 4.197 |
| 656 | 113 | 31 | 1buz-1h4x | 1buz_5 | 0.519 | 4.322 |
| 656 | 113 | 31 | 1buz-1h4x | 1buz_6 | 0.454 | 4.171 |
| 656 | 113 | 31 | 1buz-1h4x | 1buz_7 | 0.491 | 4.087 |
| 656 | 113 | 31 | 1buz-1h4x | 1buz_8 | 0.204 | 4.122 |
| 656 | 113 | 31 | 1buz-1h4x | 1buz_9 | 0.481 | 4.055 |
| 656 | 113 | 31 | 1buz-1h4x | 1buz_10 | 0.519 | 4.465 |
| 657 | 124 | 31 | 1f7u-1iq0 | 1f7u_1 | 0.992 | 1.361 |
| 657 | 124 | 31 | 1f7u-1iq0 | 1f7u_2 | 0.983 | 1.302 |
| 657 | 124 | 31 | 1f7u-1iq0 | 1f7u_3 | 0.958 | 1.628 |
| 659 | 195 | 31 | 1ig3-1ig0 | 1ig3_1 | 0.851 | 3.811 |
| 659 | 195 | 31 | 1ig3-1ig0 | 1ig3_2 | 0.923 | 5.655 |
| 659 | 195 | 31 | 1ig3-1ig0 | 1ig3_3 | 0.929 | 5.999 |
| 661 | 364 | 30 | 1pjc-1f8g | 1pjc_1 | 0.955 | 3.852 |
| 661 | 364 | 30 | 1pjc-1f8g | 1pjc_2 | 0.964 | 5.438 |
| 661 | 364 | 30 | 1pjc-1f8g | 1pjc_3 | 0.973 | 5.020 |
| 661 | 364 | 30 | 1pjc-1f8g | 1pjc_4 | 0.964 | 4.822 |
| 661 | 364 | 30 | 1pjc-1f8g | 1pjc_5 | 0.961 | 4.443 |
| 661 | 364 | 30 | 1pjc-1f8g | 1pjc_6 | 0.976 | 4.470 |
| 661 | 364 | 30 | 1pjc-1f8g | 1pjc_7 | 0.955 | 5.676 |
| 661 | 364 | 30 | 1pjc-1f8g | 1pjc_8 | 0.952 | 5.365 |
| 661 | 364 | 30 | 1pjc-1f8g | 1pjc_9 | 0.943 | 5.991 |
| 661 | 364 | 30 | 1pjc-1f8g | 1pjc_10 | 0.946 | 5.248 |
| 663 | 71 | 30 | 1xxa-1b4b | 1xxa_1 | 0.929 | 3.099 |
| 663 | 71 | 30 | 1xxa-1b4b | 1xxa_2 | 0.929 | 3.067 |
| 663 | 71 | 30 | 1xxa-1b4b | 1xxa_3 | 1.000 | 3.114 |
| 663 | 71 | 30 | 1b4b-1xxa | 1b4b_1 | 0.929 | 1.952 |
| 663 | 71 | 30 | 1b4b-1xxa | 1b4b_2 | 0.686 | 2.458 |
| 663 | 71 | 30 | 1b4b-1xxa | 1b4b_3 | 0.600 | 3.336 |
| 663 | 71 | 30 | 1b4b-1xxa | 1b4b_4 | 0.614 | 3.650 |
| 663 | 71 | 30 | 1b4b-1xxa | 1b4b_5 | 0.614 | 3.953 |
| 663 | 71 | 30 | 1b4b-1xxa | 1b4b_6 | 0.671 | 3.937 |
| 663 | 71 | 30 | 1b4b-1xxa | 1b4b_7 | 0.671 | 3.573 |
| 663 | 71 | 30 | 1b4b-1xxa | 1b4b_8 | 0.629 | 3.849 |
| 663 | 71 | 30 | 1b4b-1xxa | 1b4b_9 | 0.614 | 3.861 |
| 663 | 71 | 30 | 1b4b-1xxa | 1b4b_10 | 0.757 | 3.476 |
| 664 | 92 | 30 | 1in1-1cdz | 1in1_1 | 0.908 | 2.498 |
| 664 | 92 | 30 | 1in1-1cdz | 1in1_2 | 0.776 | 2.825 |
| 664 | 92 | 30 | 1in1-1cdz | 1in1_3 | 0.803 | 3.410 |
| 664 | 92 | 30 | 1in1-1cdz | 1in1_4 | 0.737 | 4.142 |
| 664 | 92 | 30 | 1in1-1cdz | 1in1_5 | 0.724 | 4.172 |
| 664 | 92 | 30 | 1in1-1cdz | 1in1_6 | 0.737 | 4.015 |
| 664 | 92 | 30 | 1in1-1cdz | 1in1_7 | 0.737 | 4.010 |
| 664 | 92 | 30 | 1in1-1cdz | 1in1_8 | 0.737 | 3.869 |
| 664 | 92 | 30 | 1in1-1cdz | 1in1_9 | 0.750 | 3.860 |
| 664 | 92 | 30 | 1in1-1cdz | 1in1_10 | 0.750 | 4.092 |
| 664 | 92 | 30 | 1cdz-1in1 | 1cdz_1 | 1.000 | 2.078 |
| 664 | 92 | 30 | 1cdz-1in1 | 1cdz_2 | 0.987 | 2.494 |
| 664 | 92 | 30 | 1cdz-1in1 | 1cdz_3 | 1.000 | 1.669 |
| 664 | 92 | 30 | 1cdz-1in1 | 1cdz_4 | 0.987 | 2.106 |
| 664 | 92 | 30 | 1cdz-1in1 | 1cdz_5 | 1.000 | 2.423 |
| 664 | 92 | 30 | 1cdz-1in1 | 1cdz_6 | 0.908 | 2.629 |
| 664 | 92 | 30 | 1cdz-1in1 | 1cdz_7 | 0.868 | 4.539 |
| 664 | 92 | 30 | 1cdz-1in1 | 1cdz_8 | 0.908 | 3.816 |
| 664 | 92 | 30 | 1cdz-1in1 | 1cdz_9 | 0.961 | 3.959 |
| 664 | 92 | 30 | 1cdz-1in1 | 1cdz_10 | 1.000 | 3.683 |
| 670 | 271 | 30 | 1f20-1ja0 | 1f20_1 | 0.948 | 2.633 |
| 670 | 271 | 30 | 1f20-1ja0 | 1f20_2 | 0.968 | 3.734 |
| 670 | 271 | 30 | 1f20-1ja0 | 1f20_3 | 0.960 | 4.520 |
| 670 | 271 | 30 | 1f20-1ja0 | 1f20_4 | 0.912 | 4.406 |
| 670 | 271 | 30 | 1f20-1ja0 | 1f20_5 | 0.940 | 4.718 |
| 674 | 310 | 30 | 1bf6-1psc | 1bf6_1 | 0.961 | 1.558 |
| 674 | 310 | 30 | 1bf6-1psc | 1bf6_2 | 0.951 | 1.862 |
| 674 | 310 | 30 | 1bf6-1psc | 1bf6_3 | 0.947 | 1.815 |
| 679 | 125 | 30 | 1ext-1du3 | 1ext_1 | 0.989 | 4.267 |
| 680 | 88 | 30 | 1cz5-1e32 | 1cz5_1 | 0.837 | 3.908 |
| 680 | 88 | 30 | 1cz5-1e32 | 1cz5_2 | 0.800 | 3.946 |
| 680 | 88 | 30 | 1cz5-1e32 | 1cz5_3 | 0.775 | 3.777 |
| 680 | 88 | 30 | 1cz5-1e32 | 1cz5_4 | 0.725 | 3.899 |
| 680 | 88 | 30 | 1cz5-1e32 | 1cz5_5 | 0.725 | 4.171 |
| 680 | 88 | 30 | 1cz5-1e32 | 1cz5_6 | 0.800 | 4.198 |
| 680 | 88 | 30 | 1cz5-1e32 | 1cz5_7 | 0.788 | 3.921 |
| 680 | 88 | 30 | 1cz5-1e32 | 1cz5_8 | 0.825 | 3.839 |
| 680 | 88 | 30 | 1cz5-1e32 | 1cz5_9 | 0.800 | 3.811 |
| 680 | 88 | 30 | 1cz5-1e32 | 1cz5_10 | 0.812 | 4.054 |
| 681 | 69 | 30 | 1mof-1mg1 | 1mof_1 | 0.000 | 7.026 |
| 681 | 69 | 30 | 1mof-1mg1 | 1mof_6 | 0.698 | 9.671 |
| 681 | 69 | 30 | 1mg1-1mof | 1mg1_1 | 0.736 | 4.513 |
| 695 | 351 | 29 | 1evi-1c0p | 1evi_1 | 0.953 | 2.003 |
| 695 | 351 | 29 | 1evi-1c0p | 1evi_2 | 0.922 | 1.966 |
| 695 | 351 | 29 | 1c0p-1evi | 1c0p_1 | 0.950 | 2.861 |
| 695 | 351 | 29 | 1c0p-1evi | 1c0p_2 | 0.944 | 3.385 |

n: internal numbering; length of alignment; % sequence%ID (cf. Homstrad); refA-refB: reference alignment; altA: alternative snapshot for A; SP: sum-of-pairs score of alt. alignment w/r reference

| n | length | %ID | refA−refB | altA | SP | RMSD |
|---|---|---|---|---|---|---|
| 695 | 351 | 29 | 1c0p−1evi | 1c0p_3 | 0.959 | 3.526 |
| 695 | 351 | 29 | 1c0p−1evi | 1c0p_4 | 0.922 | 3.746 |
| 695 | 351 | 29 | 1c0p−1evi | 1c0p_5 | 0.912 | 3.949 |
| 695 | 351 | 29 | 1c0p−1evi | 1c0p_6 | 0.928 | 3.734 |
| 695 | 351 | 29 | 1c0p−1evi | 1c0p_7 | 0.937 | 3.797 |
| 697 | 239 | 29 | 1ih5−1fx8 | 1ih5_1 | 0.629 | 4.478 |
| 697 | 239 | 29 | 1ih5−1fx8 | 1ih5_2 | 0.653 | 4.631 |
| 697 | 239 | 29 | 1ih5−1fx8 | 1ih5_3 | 0.638 | 5.124 |
| 697 | 239 | 29 | 1ih5−1fx8 | 1ih5_4 | 0.437 | 5.449 |
| 697 | 239 | 29 | 1ih5−1fx8 | 1ih5_5 | 0.549 | 5.217 |
| 697 | 239 | 29 | 1ih5−1fx8 | 1ih5_6 | 0.554 | 5.901 |
| 698 | 528 | 29 | 1aoz−1a65 | 1aoz_1 | 0.949 | 2.409 |
| 698 | 528 | 29 | 1a65−1aoz | 1a65_1 | 0.928 | 1.801 |
| 698 | 528 | 29 | 1a65−1aoz | 1a65_2 | 0.898 | 2.331 |
| 698 | 528 | 29 | 1a65−1aoz | 1a65_3 | 0.887 | 2.272 |
| 698 | 528 | 29 | 1a65−1aoz | 1a65_4 | 0.870 | 2.566 |
| 698 | 528 | 29 | 1a65−1aoz | 1a65_5 | 0.887 | 2.368 |
| 698 | 528 | 29 | 1a65−1aoz | 1a65_6 | 0.877 | 2.565 |
| 698 | 528 | 29 | 1a65−1aoz | 1a65_7 | 0.902 | 2.587 |
| 698 | 528 | 29 | 1a65−1aoz | 1a65_8 | 0.885 | 2.818 |
| 698 | 528 | 29 | 1a65−1aoz | 1a65_9 | 0.881 | 2.857 |
| 698 | 528 | 29 | 1a65−1aoz | 1a65_10 | 0.870 | 2.847 |
| 700 | 206 | 29 | 1bk7−1bol | 1bk7_1 | 0.918 | 1.777 |
| 700 | 206 | 29 | 1bk7−1bol | 1bk7_2 | 0.912 | 2.395 |
| 700 | 206 | 29 | 1bk7−1bol | 1bk7_3 | 0.900 | 2.304 |
| 700 | 206 | 29 | 1bk7−1bol | 1bk7_4 | 0.859 | 2.417 |
| 700 | 206 | 29 | 1bk7−1bol | 1bk7_5 | 0.912 | 2.767 |
| 700 | 206 | 29 | 1bk7−1bol | 1bk7_6 | 0.871 | 2.409 |
| 700 | 206 | 29 | 1bk7−1bol | 1bk7_7 | 0.918 | 2.263 |
| 700 | 206 | 29 | 1bk7−1bol | 1bk7_8 | 0.853 | 2.529 |
| 700 | 206 | 29 | 1bk7−1bol | 1bk7_9 | 0.912 | 2.375 |
| 700 | 206 | 29 | 1bk7−1bol | 1bk7_10 | 0.900 | 1.967 |
| 700 | 206 | 29 | 1bol−1bk7 | 1bol_1 | 0.906 | 2.896 |
| 700 | 206 | 29 | 1bol−1bk7 | 1bol_2 | 0.959 | 2.754 |
| 700 | 206 | 29 | 1bol−1bk7 | 1bol_3 | 0.929 | 2.907 |
| 700 | 206 | 29 | 1bol−1bk7 | 1bol_4 | 0.876 | 3.206 |
| 700 | 206 | 29 | 1bol−1bk7 | 1bol_5 | 0.876 | 3.162 |
| 700 | 206 | 29 | 1bol−1bk7 | 1bol_6 | 0.853 | 3.161 |
| 700 | 206 | 29 | 1bol−1bk7 | 1bol_7 | 0.865 | 2.895 |
| 700 | 206 | 29 | 1bol−1bk7 | 1bol_8 | 0.847 | 3.101 |
| 700 | 206 | 29 | 1bol−1bk7 | 1bol_9 | 0.882 | 3.239 |
| 700 | 206 | 29 | 1bol−1bk7 | 1bol_10 | 0.841 | 3.301 |
| 701 | 138 | 29 | 1ily−1jj2 | 1ily_1 | 0.911 | 2.344 |
| 701 | 138 | 29 | 1ily−1jj2 | 1ily_2 | 0.867 | 2.572 |
| 701 | 138 | 29 | 1ily−1jj2 | 1ily_3 | 0.844 | 2.662 |
| 701 | 138 | 29 | 1ily−1jj2 | 1ily_4 | 0.833 | 2.627 |
| 701 | 138 | 29 | 1ily−1jj2 | 1ily_5 | 0.844 | 2.624 |
| 701 | 138 | 29 | 1ily−1jj2 | 1ily_6 | 0.844 | 2.831 |
| 701 | 138 | 29 | 1ily−1jj2 | 1ily_7 | 0.789 | 3.315 |
| 701 | 138 | 29 | 1ily−1jj2 | 1ily_8 | 0.856 | 3.215 |
| 701 | 138 | 29 | 1ily−1jj2 | 1ily_9 | 0.867 | 3.173 |
| 701 | 138 | 29 | 1ily−1jj2 | 1ily_10 | 0.744 | 3.818 |
| 702 | 284 | 29 | 1ici−1j8f | 1ici_1 | 0.929 | 3.334 |
| 704 | 91 | 29 | 1l8c−1f81 | 1l8c_1 | 0.812 | 2.486 |
| 704 | 91 | 29 | 1l8c−1f81 | 1l8c_2 | 0.887 | 2.719 |
| 704 | 91 | 29 | 1l8c−1f81 | 1l8c_3 | 0.875 | 3.193 |
| 704 | 91 | 29 | 1l8c−1f81 | 1l8c_4 | 0.788 | 3.383 |
| 704 | 91 | 29 | 1l8c−1f81 | 1l8c_5 | 0.762 | 3.038 |
| 704 | 91 | 29 | 1l8c−1f81 | 1l8c_6 | 0.738 | 3.596 |
| 704 | 91 | 29 | 1l8c−1f81 | 1l8c_7 | 0.750 | 3.412 |
| 704 | 91 | 29 | 1l8c−1f81 | 1l8c_8 | 0.762 | 3.225 |
| 704 | 91 | 29 | 1l8c−1f81 | 1l8c_9 | 0.738 | 3.639 |
| 704 | 91 | 29 | 1l8c−1f81 | 1l8c_10 | 0.738 | 3.679 |
| 709 | 271 | 28 | 1ako−1bix | 1ako_1 | 0.915 | 1.866 |
| 709 | 271 | 28 | 1ako−1bix | 1ako_2 | 0.944 | 1.979 |
| 709 | 271 | 28 | 1ako−1bix | 1ako_3 | 0.935 | 2.183 |
| 709 | 271 | 28 | 1ako−1bix | 1ako_4 | 0.931 | 2.555 |
| 709 | 271 | 28 | 1ako−1bix | 1ako_5 | 0.911 | 2.571 |
| 709 | 271 | 28 | 1ako−1bix | 1ako_6 | 0.915 | 2.512 |
| 709 | 271 | 28 | 1ako−1bix | 1ako_7 | 0.895 | 2.529 |
| 709 | 271 | 28 | 1ako−1bix | 1ako_8 | 0.931 | 2.684 |
| 709 | 271 | 28 | 1ako−1bix | 1ako_9 | 0.923 | 2.612 |
| 709 | 271 | 28 | 1ako−1bix | 1ako_10 | 0.927 | 3.073 |
| 709 | 271 | 28 | 1bix−1ako | 1bix_1 | 0.851 | 2.210 |
| 709 | 271 | 28 | 1bix−1ako | 1bix_2 | 0.863 | 2.850 |
| 709 | 271 | 28 | 1bix−1ako | 1bix_3 | 0.843 | 3.274 |
| 709 | 271 | 28 | 1bix−1ako | 1bix_4 | 0.883 | 3.139 |
| 709 | 271 | 28 | 1bix−1ako | 1bix_5 | 0.875 | 3.243 |
| 709 | 271 | 28 | 1bix−1ako | 1bix_6 | 0.827 | 3.202 |
| 709 | 271 | 28 | 1bix−1ako | 1bix_7 | 0.806 | 3.273 |
| 709 | 271 | 28 | 1bix−1ako | 1bix_8 | 0.867 | 3.174 |
| 709 | 271 | 28 | 1bix−1ako | 1bix_9 | 0.835 | 3.280 |
| 709 | 271 | 28 | 1bix−1ako | 1bix_10 | 0.827 | 3.267 |
| 711 | 149 | 28 | 1jlxa2−1jlxa1 | 1jlxa2_1 | 0.964 | 1.468 |
| 711 | 149 | 28 | 1jlxa2−1jlxa1 | 1jlxa2_2 | 0.920 | 1.870 |
| 711 | 149 | 28 | 1jlxa2−1jlxa1 | 1jlxa2_3 | 0.920 | 1.891 |
| 711 | 149 | 28 | 1jlxa2−1jlxa1 | 1jlxa1_1 | 0.993 | 1.168 |
| 711 | 149 | 28 | 1jlxa2−1jlxa1 | 1jlxa1_2 | 0.905 | 1.497 |
| 711 | 149 | 28 | 1jlxa2−1jlxa1 | 1jlxa1_3 | 0.956 | 1.634 |
| 717 | 174 | 28 | 1e20−1g63 | 1e20_1 | 0.932 | 2.599 |
| 717 | 174 | 28 | 1e20−1g63 | 1e20_2 | 0.963 | 3.131 |
| 717 | 174 | 28 | 1e20−1g63 | 1e20_3 | 0.938 | 3.467 |
| 717 | 174 | 28 | 1e20−1g63 | 1e20_4 | 0.944 | 3.612 |
| 717 | 174 | 28 | 1e20−1g63 | 1e20_5 | 0.901 | 3.486 |
| 717 | 174 | 28 | 1e20−1g63 | 1e20_6 | 0.864 | 3.915 |
| 717 | 174 | 28 | 1e20−1g63 | 1e20_7 | 0.901 | 3.945 |
| 717 | 174 | 28 | 1e20−1g63 | 1e20_8 | 0.938 | 3.937 |
| 717 | 174 | 28 | 1e20−1g63 | 1e20_9 | 0.920 | 4.055 |
| 717 | 174 | 28 | 1e20−1g63 | 1e20_10 | 0.914 | 3.864 |
| 718 | 291 | 28 | 1wer−1nf1 | 1wer_1 | 0.965 | 2.802 |
| 718 | 291 | 28 | 1wer−1nf1 | 1wer_2 | 0.965 | 3.185 |
| 718 | 291 | 28 | 1wer−1nf1 | 1wer_3 | 0.977 | 3.285 |
| 726 | 167 | 28 | 1lba−1j3g | 1lba_1 | 0.662 | 1.834 |
| 726 | 167 | 28 | 1lba−1j3g | 1lba_2 | 0.577 | 2.695 |
| 726 | 167 | 28 | 1lba−1j3g | 1lba_3 | 0.646 | 2.642 |
| 726 | 167 | 28 | 1lba−1j3g | 1lba_4 | 0.515 | 2.987 |
| 726 | 167 | 28 | 1lba−1j3g | 1lba_5 | 0.577 | 3.242 |
| 726 | 167 | 28 | 1lba−1j3g | 1lba_6 | 0.631 | 3.549 |
| 726 | 167 | 28 | 1lba−1j3g | 1lba_7 | 0.669 | 3.822 |
| 726 | 167 | 28 | 1lba−1j3g | 1lba_8 | 0.723 | 3.817 |
| 726 | 167 | 28 | 1lba−1j3g | 1lba_9 | 0.546 | 4.007 |
| 726 | 167 | 28 | 1lba−1j3g | 1lba_10 | 0.800 | 3.919 |
| 726 | 167 | 28 | 1j3g−1lba | 1j3g_1 | 0.777 | 3.778 |
| 726 | 167 | 28 | 1j3g−1lba | 1j3g_2 | 0.715 | 6.067 |
| 726 | 167 | 28 | 1j3g−1lba | 1j3g_3 | 0.723 | 5.587 |
| 726 | 167 | 28 | 1j3g−1lba | 1j3g_4 | 0.785 | 6.486 |
| 726 | 167 | 28 | 1j3g−1lba | 1j3g_5 | 0.762 | 7.147 |
| 726 | 167 | 28 | 1j3g−1lba | 1j3g_6 | 0.692 | 7.109 |
| 726 | 167 | 28 | 1j3g−1lba | 1j3g_7 | 0.700 | 7.042 |
| 726 | 167 | 28 | 1j3g−1lba | 1j3g_8 | 0.738 | 6.989 |
| 726 | 167 | 28 | 1j3g−1lba | 1j3g_9 | 0.746 | 6.577 |
| 726 | 167 | 28 | 1j3g−1lba | 1j3g_10 | 0.723 | 6.793 |
| 727 | 342 | 28 | 1fp2−1fp1 | 1fp2_1 | 0.966 | 3.698 |
| 727 | 342 | 28 | 1fp2−1fp1 | 1fp2_2 | 0.923 | 5.129 |
| 727 | 342 | 28 | 1fp2−1fp1 | 1fp2_3 | 0.886 | 6.021 |
| 727 | 342 | 28 | 1fp2−1fp1 | 1fp2_4 | 0.917 | 6.252 |
| 728 | 81 | 28 | 2af8−1acp | 2af8_1 | 0.216 | 4.708 |
| 728 | 81 | 28 | 2af8−1acp | 2af8_2 | 0.743 | 5.520 |
| 728 | 81 | 28 | 2af8−1acp | 2af8_3 | 0.338 | 6.138 |
| 728 | 81 | 28 | 2af8−1acp | 2af8_4 | 0.000 | 7.300 |
| 728 | 81 | 28 | 2af8−1acp | 2af8_5 | 0.081 | 7.888 |
| 728 | 81 | 28 | 2af8−1acp | 2af8_6 | 0.203 | 8.188 |
| 734 | 87 | 27 | 2abd−1hbk | 2abd_1 | 0.942 | 2.788 |
| 734 | 87 | 27 | 2abd−1hbk | 2abd_2 | 0.942 | 2.143 |
| 734 | 87 | 27 | 2abd−1hbk | 2abd_3 | 1.000 | 3.260 |
| 734 | 87 | 27 | 2abd−1hbk | 2abd_4 | 0.837 | 2.956 |
| 734 | 87 | 27 | 2abd−1hbk | 2abd_5 | 0.942 | 3.276 |
| 734 | 87 | 27 | 2abd−1hbk | 2abd_6 | 0.698 | 3.381 |
| 734 | 87 | 27 | 2abd−1hbk | 2abd_7 | 0.698 | 3.693 |
| 734 | 87 | 27 | 2abd−1hbk | 2abd_8 | 0.756 | 3.410 |
| 734 | 87 | 27 | 2abd−1hbk | 2abd_9 | 0.686 | 3.774 |
| 734 | 87 | 27 | 2abd−1hbk | 2abd_10 | 0.802 | 3.368 |
| 734 | 87 | 27 | 1hbk−2abd | 1hbk_1 | 1.000 | 2.494 |
| 734 | 87 | 27 | 1hbk−2abd | 1hbk_2 | 0.942 | 2.506 |
| 734 | 87 | 27 | 1hbk−2abd | 1hbk_3 | 1.000 | 2.464 |
| 734 | 87 | 27 | 1hbk−2abd | 1hbk_4 | 0.860 | 2.959 |
| 734 | 87 | 27 | 1hbk−2abd | 1hbk_5 | 0.942 | 3.138 |
| 734 | 87 | 27 | 1hbk−2abd | 1hbk_6 | 1.000 | 3.754 |
| 734 | 87 | 27 | 1hbk−2abd | 1hbk_7 | 0.756 | 5.536 |
| 734 | 87 | 27 | 1hbk−2abd | 1hbk_8 | 0.837 | 4.944 |
| 734 | 87 | 27 | 1hbk−2abd | 1hbk_9 | 0.942 | 4.836 |
| 734 | 87 | 27 | 1hbk−2abd | 1hbk_10 | 0.767 | 5.284 |
| 735 | 286 | 27 | 5daa−1a3g | 5daa_1 | 0.959 | 1.610 |
| 735 | 286 | 27 | 5daa−1a3g | 5daa_2 | 0.963 | 1.975 |
| 735 | 286 | 27 | 5daa−1a3g | 5daa_3 | 0.959 | 1.801 |
| 735 | 286 | 27 | 5daa−1a3g | 5daa_4 | 0.963 | 1.982 |
| 735 | 286 | 27 | 5daa−1a3g | 5daa_5 | 0.978 | 2.089 |
| 735 | 286 | 27 | 5daa−1a3g | 5daa_6 | 0.978 | 2.390 |
| 735 | 286 | 27 | 5daa−1a3g | 5daa_7 | 0.978 | 2.332 |
| 735 | 286 | 27 | 5daa−1a3g | 5daa_8 | 0.974 | 2.307 |
| 735 | 286 | 27 | 5daa−1a3g | 5daa_9 | 0.955 | 2.427 |
| 735 | 286 | 27 | 5daa−1a3g | 5daa_10 | 0.918 | 2.496 |
| 736 | 76 | 27 | 1b4a−1aoy | 1b4a_1 | 0.986 | 1.803 |
| 736 | 76 | 27 | 1b4a−1aoy | 1b4a_2 | 1.000 | 2.716 |
| 736 | 76 | 27 | 1b4a−1aoy | 1b4a_3 | 1.000 | 2.686 |
| 736 | 76 | 27 | 1b4a−1aoy | 1b4a_4 | 0.986 | 3.599 |
| 736 | 76 | 27 | 1b4a−1aoy | 1b4a_5 | 0.986 | 3.308 |
| 736 | 76 | 27 | 1b4a−1aoy | 1b4a_6 | 0.986 | 3.825 |
| 740 | 141 | 27 | 1coz−1b6t | 1coz_1 | 0.847 | 1.720 |
| 740 | 141 | 27 | 1coz−1b6t | 1coz_2 | 0.907 | 2.579 |
| 740 | 141 | 27 | 1coz−1b6t | 1coz_3 | 0.780 | 2.967 |
| 740 | 141 | 27 | 1coz−1b6t | 1coz_4 | 0.831 | 3.043 |
| 740 | 141 | 27 | 1coz−1b6t | 1coz_5 | 0.839 | 3.367 |
| 740 | 141 | 27 | 1coz−1b6t | 1coz_6 | 0.780 | 3.644 |
| 740 | 141 | 27 | 1coz−1b6t | 1coz_7 | 0.831 | 3.497 |
| 740 | 141 | 27 | 1coz−1b6t | 1coz_8 | 0.653 | 3.871 |
| 740 | 141 | 27 | 1coz−1b6t | 1coz_9 | 0.746 | 3.962 |
| 740 | 141 | 27 | 1coz−1b6t | 1coz_10 | 0.610 | 4.083 |
| 740 | 141 | 27 | 1b6t−1coz | 1b6t_1 | 0.907 | 2.283 |
| 740 | 141 | 27 | 1b6t−1coz | 1b6t_2 | 0.907 | 2.071 |
| 740 | 141 | 27 | 1b6t−1coz | 1b6t_3 | 0.907 | 2.487 |
| 740 | 141 | 27 | 1b6t−1coz | 1b6t_4 | 0.847 | 2.540 |
| 740 | 141 | 27 | 1b6t−1coz | 1b6t_5 | 0.805 | 2.768 |
| 740 | 141 | 27 | 1b6t−1coz | 1b6t_6 | 0.814 | 2.737 |
| 740 | 141 | 27 | 1b6t−1coz | 1b6t_7 | 0.814 | 2.945 |
| 740 | 141 | 27 | 1b6t−1coz | 1b6t_8 | 0.771 | 3.136 |
| 740 | 141 | 27 | 1b6t−1coz | 1b6t_9 | 0.814 | 3.274 |
| 740 | 141 | 27 | 1b6t−1coz | 1b6t_10 | 0.814 | 3.284 |
| 743 | 334 | 27 | 1doz−1hrk | 1doz_1 | 0.948 | 2.409 |
| 743 | 334 | 27 | 1doz−1hrk | 1doz_2 | 0.944 | 2.977 |
| 743 | 334 | 27 | 1doz−1hrk | 1doz_3 | 0.931 | 3.257 |
| 743 | 334 | 27 | 1doz−1hrk | 1doz_4 | 0.863 | 3.946 |
| 743 | 334 | 27 | 1doz−1hrk | 1doz_5 | 0.895 | 4.326 |
| 743 | 334 | 27 | 1doz−1hrk | 1doz_6 | 0.895 | 4.156 |
| 743 | 334 | 27 | 1doz−1hrk | 1doz_7 | 0.895 | 4.333 |
| 743 | 334 | 27 | 1doz−1hrk | 1doz_8 | 0.873 | 4.187 |
| 743 | 334 | 27 | 1doz−1hrk | 1doz_9 | 0.846 | 4.423 |
| 743 | 334 | 27 | 1doz−1hrk | 1doz_10 | 0.853 | 4.095 |
| 744 | 347 | 27 | 2nac−1gdh | 2nac_1 | 0.942 | 1.664 |
| 744 | 347 | 27 | 2nac−1gdh | 2nac_2 | 0.961 | 2.140 |
| 744 | 347 | 27 | 2nac−1gdh | 2nac_3 | 0.997 | 2.205 |
| 744 | 347 | 27 | 2nac−1gdh | 2nac_4 | 0.977 | 2.479 |
| 744 | 347 | 27 | 2nac−1gdh | 2nac_5 | 0.965 | 2.429 |
| 744 | 347 | 27 | 2nac−1gdh | 2nac_6 | 0.948 | 2.202 |
| 746 | 132 | 27 | 1shs−1gme | 1shs_1 | 0.946 | 1.861 |
| 746 | 132 | 27 | 1gme−1shs | 1gme_1 | 0.847 | 5.929 |
| 746 | 132 | 27 | 1gme−1shs | 1gme_2 | 0.874 | 7.580 |
| 746 | 132 | 27 | 1gme−1shs | 1gme_3 | 0.865 | 7.509 |
| 748 | 109 | 27 | 1kvv−1jid | 1kvv_1 | 0.447 | 6.144 |
| 748 | 109 | 27 | 1kvv−1jid | 1kvv_2 | 0.713 | 9.453 |
| 748 | 109 | 27 | 1kvv−1jid | 1kvv_3 | 0.521 | 9.481 |
| 748 | 109 | 27 | 1kvv−1jid | 1kvv_4 | 0.543 | 9.459 |
| 748 | 109 | 27 | 1kvv−1jid | 1kvv_6 | 0.064 | 9.686 |

n: internal numbering; length of alignment; % sequence%ID (cf. Homstrad); refA−refB: reference alignment; altA: alternative snapshot for A; SP: sum−of−pairs score of alt. alignment w/r reference

| n | length | %ID | refA−refB | altA | SP | RMSD |
|---|---|---|---|---|---|---|
| 748 | 109 | 27 | 1kvv−1jid | 1kvv_7 | 0.000 | 9.545 |
| 748 | 109 | 27 | 1jid−1kvv | 1jid_1 | 0.660 | 4.409 |
| 748 | 109 | 27 | 1jid−1kvv | 1jid_2 | 0.660 | 4.092 |
| 748 | 109 | 27 | 1jid−1kvv | 1jid_3 | 0.660 | 4.629 |
| 748 | 109 | 27 | 1jid−1kvv | 1jid_4 | 0.660 | 4.983 |
| 748 | 109 | 27 | 1jid−1kvv | 1jid_5 | 0.649 | 5.537 |
| 748 | 109 | 27 | 1jid−1kvv | 1jid_6 | 0.649 | 5.443 |
| 748 | 109 | 27 | 1jid−1kvv | 1jid_7 | 0.660 | 6.426 |
| 748 | 109 | 27 | 1jid−1kvv | 1jid_8 | 0.660 | 5.693 |
| 748 | 109 | 27 | 1jid−1kvv | 1jid_9 | 0.660 | 5.923 |
| 748 | 109 | 27 | 1jid−1kvv | 1jid_10 | 0.660 | 5.958 |
| 750 | 223 | 27 | 1pysb−1pysa | 1pysb_1 | 0.955 | 1.637 |
| 750 | 223 | 27 | 1pysb−1pysa | 1pysa_1 | 0.943 | 1.851 |
| 752 | 182 | 27 | 1etp−1fcd | 1etp_1 | 0.808 | 2.400 |
| 752 | 182 | 27 | 1etp−1fcd | 1etp_2 | 0.808 | 2.948 |
| 752 | 182 | 27 | 1etp−1fcd | 1etp_3 | 0.838 | 2.766 |
| 752 | 182 | 27 | 1etp−1fcd | 1etp_4 | 0.874 | 3.100 |
| 752 | 182 | 27 | 1etp−1fcd | 1etp_5 | 0.844 | 3.290 |
| 752 | 182 | 27 | 1etp−1fcd | 1etp_6 | 0.772 | 3.147 |
| 752 | 182 | 27 | 1etp−1fcd | 1etp_7 | 0.790 | 3.278 |
| 752 | 182 | 27 | 1etp−1fcd | 1etp_8 | 0.749 | 3.472 |
| 752 | 182 | 27 | 1etp−1fcd | 1etp_9 | 0.796 | 3.495 |
| 752 | 182 | 27 | 1etp−1fcd | 1etp_10 | 0.814 | 3.687 |
| 752 | 182 | 27 | 1fcd−1etp | 1fcd_1 | 0.940 | 2.410 |
| 752 | 182 | 27 | 1fcd−1etp | 1fcd_2 | 0.946 | 2.442 |
| 754 | 117 | 26 | 1c20−1ig6 | 1c20_1 | 0.962 | 3.468 |
| 754 | 117 | 26 | 1c20−1ig6 | 1c20_2 | 0.896 | 3.388 |
| 754 | 117 | 26 | 1c20−1ig6 | 1c20_3 | 0.830 | 3.629 |
| 754 | 117 | 26 | 1c20−1ig6 | 1c20_4 | 0.896 | 3.908 |
| 754 | 117 | 26 | 1c20−1ig6 | 1c20_5 | 0.896 | 3.701 |
| 754 | 117 | 26 | 1c20−1ig6 | 1c20_6 | 0.877 | 3.949 |
| 754 | 117 | 26 | 1c20−1ig6 | 1c20_7 | 0.764 | 4.085 |
| 754 | 117 | 26 | 1c20−1ig6 | 1c20_8 | 0.745 | 3.951 |
| 754 | 117 | 26 | 1c20−1ig6 | 1c20_9 | 0.745 | 3.925 |
| 754 | 117 | 26 | 1c20−1ig6 | 1c20_10 | 0.915 | 3.769 |
| 754 | 117 | 26 | 1ig6−1c20 | 1ig6_1 | 0.858 | 4.122 |
| 754 | 117 | 26 | 1ig6−1c20 | 1ig6_2 | 0.783 | 3.414 |
| 754 | 117 | 26 | 1ig6−1c20 | 1ig6_3 | 0.774 | 5.122 |
| 754 | 117 | 26 | 1ig6−1c20 | 1ig6_4 | 0.745 | 5.022 |
| 754 | 117 | 26 | 1ig6−1c20 | 1ig6_5 | 0.594 | 5.070 |
| 754 | 117 | 26 | 1ig6−1c20 | 1ig6_6 | 0.425 | 5.616 |
| 754 | 117 | 26 | 1ig6−1c20 | 1ig6_7 | 0.717 | 5.854 |
| 754 | 117 | 26 | 1ig6−1c20 | 1ig6_8 | 0.632 | 5.864 |
| 754 | 117 | 26 | 1ig6−1c20 | 1ig6_9 | 0.575 | 5.629 |
| 754 | 117 | 26 | 1ig6−1c20 | 1ig6_10 | 0.415 | 5.390 |
| 757 | 98 | 26 | 1exh−2xbd | 1exh_1 | 0.847 | 2.953 |
| 757 | 98 | 26 | 1exh−2xbd | 1exh_2 | 0.882 | 3.242 |
| 757 | 98 | 26 | 1exh−2xbd | 1exh_3 | 0.847 | 3.398 |
| 757 | 98 | 26 | 1exh−2xbd | 1exh_4 | 0.906 | 3.461 |
| 757 | 98 | 26 | 1exh−2xbd | 1exh_5 | 0.847 | 3.388 |
| 757 | 98 | 26 | 1exh−2xbd | 1exh_6 | 0.929 | 3.586 |
| 757 | 98 | 26 | 1exh−2xbd | 1exh_7 | 0.953 | 3.214 |
| 757 | 98 | 26 | 1exh−2xbd | 1exh_8 | 0.929 | 3.518 |
| 757 | 98 | 26 | 1exh−2xbd | 1exh_9 | 0.929 | 3.308 |
| 757 | 98 | 26 | 1exh−2xbd | 1exh_10 | 0.765 | 3.323 |
| 757 | 98 | 26 | 2xbd−1exh | 2xbd_1 | 0.965 | 1.470 |
| 757 | 98 | 26 | 2xbd−1exh | 2xbd_2 | 0.882 | 1.251 |
| 757 | 98 | 26 | 2xbd−1exh | 2xbd_3 | 0.965 | 1.661 |
| 757 | 98 | 26 | 2xbd−1exh | 2xbd_4 | 0.965 | 1.843 |
| 757 | 98 | 26 | 2xbd−1exh | 2xbd_5 | 0.906 | 1.706 |
| 757 | 98 | 26 | 2xbd−1exh | 2xbd_6 | 0.965 | 1.537 |
| 757 | 98 | 26 | 2xbd−1exh | 2xbd_7 | 0.894 | 1.772 |
| 757 | 98 | 26 | 2xbd−1exh | 2xbd_8 | 0.906 | 1.928 |
| 757 | 98 | 26 | 2xbd−1exh | 2xbd_9 | 0.965 | 1.933 |
| 757 | 98 | 26 | 2xbd−1exh | 2xbd_10 | 0.965 | 1.740 |
| 761 | 154 | 26 | 1dps−1qgh | 1dps_1 | 1.000 | 1.754 |
| 761 | 154 | 26 | 1dps−1qgh | 1dps_2 | 0.993 | 1.989 |
| 761 | 154 | 26 | 1dps−1qgh | 1dps_3 | 0.993 | 2.233 |
| 763 | 231 | 26 | 1wod−1atg | 1wod_1 | 0.955 | 1.803 |
| 763 | 231 | 26 | 1wod−1atg | 1wod_2 | 0.960 | 2.127 |
| 763 | 231 | 26 | 1wod−1atg | 1wod_3 | 0.929 | 2.338 |
| 763 | 231 | 26 | 1wod−1atg | 1wod_4 | 0.929 | 2.442 |
| 763 | 231 | 26 | 1wod−1atg | 1wod_5 | 0.942 | 2.146 |
| 763 | 231 | 26 | 1atg−1wod | 1atg_1 | 0.955 | 2.634 |
| 763 | 231 | 26 | 1atg−1wod | 1atg_2 | 0.991 | 2.928 |
| 763 | 231 | 26 | 1atg−1wod | 1atg_3 | 0.996 | 2.857 |
| 763 | 231 | 26 | 1atg−1wod | 1atg_4 | 0.933 | 3.139 |
| 763 | 231 | 26 | 1atg−1wod | 1atg_5 | 0.973 | 2.965 |
| 763 | 231 | 26 | 1atg−1wod | 1atg_6 | 0.969 | 3.081 |
| 763 | 231 | 26 | 1atg−1wod | 1atg_7 | 0.991 | 3.061 |
| 763 | 231 | 26 | 1atg−1wod | 1atg_8 | 0.951 | 3.032 |
| 763 | 231 | 26 | 1atg−1wod | 1atg_9 | 0.964 | 2.960 |
| 763 | 231 | 26 | 1atg−1wod | 1atg_10 | 0.951 | 3.336 |
| 764 | 241 | 26 | 1mml−1vrt | 1mml_1 | 1.000 | 2.083 |
| 764 | 241 | 26 | 1mml−1vrt | 1mml_2 | 0.965 | 2.493 |
| 764 | 241 | 26 | 1mml−1vrt | 1mml_3 | 0.947 | 2.275 |
| 764 | 241 | 26 | 1mml−1vrt | 1mml_4 | 0.947 | 2.527 |
| 764 | 241 | 26 | 1mml−1vrt | 1mml_5 | 0.965 | 3.515 |
| 764 | 241 | 26 | 1mml−1vrt | 1mml_6 | 0.934 | 3.665 |
| 764 | 241 | 26 | 1mml−1vrt | 1mml_7 | 0.960 | 3.420 |
| 767 | 82 | 26 | 1fip−1ntc | 1fip_1 | 1.000 | 2.018 |
| 767 | 82 | 26 | 1fip−1ntc | 1fip_2 | 1.000 | 2.637 |
| 767 | 82 | 26 | 1fip−1ntc | 1fip_3 | 1.000 | 3.077 |
| 767 | 82 | 26 | 1fip−1ntc | 1fip_4 | 0.861 | 3.183 |
| 767 | 82 | 26 | 1fip−1ntc | 1fip_5 | 1.000 | 2.560 |
| 767 | 82 | 26 | 1fip−1ntc | 1fip_6 | 0.931 | 3.327 |
| 767 | 82 | 26 | 1fip−1ntc | 1fip_7 | 0.986 | 3.435 |
| 767 | 82 | 26 | 1fip−1ntc | 1fip_8 | 0.972 | 2.910 |
| 767 | 82 | 26 | 1fip−1ntc | 1fip_9 | 0.972 | 2.666 |
| 767 | 82 | 26 | 1fip−1ntc | 1fip_10 | 0.972 | 4.655 |
| 767 | 82 | 26 | 1ntc−1fip | 1ntc_1 | 0.694 | 4.877 |
| 767 | 82 | 26 | 1ntc−1fip | 1ntc_2 | 0.556 | 7.819 |
| 767 | 82 | 26 | 1ntc−1fip | 1ntc_3 | 0.722 | 8.875 |
| 773 | 85 | 26 | 1h8c−1i42 | 1h8c_1 | 0.753 | 3.200 |
| 773 | 85 | 26 | 1h8c−1i42 | 1h8c_2 | 0.571 | 3.488 |
| 773 | 85 | 26 | 1h8c−1i42 | 1h8c_3 | 0.675 | 3.664 |
| 773 | 85 | 26 | 1h8c−1i42 | 1h8c_4 | 0.104 | 4.030 |
| 773 | 85 | 26 | 1h8c−1i42 | 1h8c_5 | 0.636 | 4.218 |
| 773 | 85 | 26 | 1h8c−1i42 | 1h8c_6 | 0.558 | 4.038 |
| 773 | 85 | 26 | 1h8c−1i42 | 1h8c_7 | 0.662 | 4.135 |
| 773 | 85 | 26 | 1h8c−1i42 | 1h8c_8 | 0.649 | 4.126 |
| 773 | 85 | 26 | 1h8c−1i42 | 1h8c_9 | 0.429 | 4.470 |
| 773 | 85 | 26 | 1h8c−1i42 | 1h8c_10 | 0.571 | 4.323 |
| 773 | 85 | 26 | 1i42−1h8c | 1i42_1 | 0.584 | 4.205 |
| 775 | 246 | 26 | 1ca1−1ah7 | 1ca1_1 | 0.946 | 1.533 |
| 775 | 246 | 26 | 1ca1−1ah7 | 1ca1_2 | 0.896 | 1.885 |
| 775 | 246 | 26 | 1ca1−1ah7 | 1ca1_3 | 0.932 | 2.205 |
| 775 | 246 | 26 | 1ca1−1ah7 | 1ca1_4 | 0.887 | 2.187 |
| 775 | 246 | 26 | 1ca1−1ah7 | 1ca1_5 | 0.910 | 2.267 |
| 775 | 246 | 26 | 1ca1−1ah7 | 1ca1_6 | 0.828 | 2.463 |
| 775 | 246 | 26 | 1ca1−1ah7 | 1ca1_7 | 0.842 | 2.536 |
| 775 | 246 | 26 | 1ah7−1ca1 | 1ah7_1 | 0.964 | 1.634 |
| 775 | 246 | 26 | 1ah7−1ca1 | 1ah7_2 | 0.959 | 1.688 |
| 775 | 246 | 26 | 1ah7−1ca1 | 1ah7_3 | 0.928 | 1.642 |
| 775 | 246 | 26 | 1ah7−1ca1 | 1ah7_4 | 0.973 | 1.533 |
| 775 | 246 | 26 | 1ah7−1ca1 | 1ah7_5 | 0.941 | 1.876 |
| 775 | 246 | 26 | 1ah7−1ca1 | 1ah7_6 | 0.896 | 2.248 |
| 775 | 246 | 26 | 1ah7−1ca1 | 1ah7_7 | 0.801 | 2.315 |
| 775 | 246 | 26 | 1ah7−1ca1 | 1ah7_8 | 0.810 | 2.134 |
| 775 | 246 | 26 | 1ah7−1ca1 | 1ah7_9 | 0.846 | 2.265 |
| 775 | 246 | 26 | 1ah7−1ca1 | 1ah7_10 | 0.878 | 2.156 |
| 778 | 512 | 25 | 1dpe−1jet | 1dpe_1 | 0.449 | 4.451 |
| 778 | 512 | 25 | 1dpe−1jet | 1dpe_2 | 0.637 | 4.352 |
| 778 | 512 | 25 | 1dpe−1jet | 1dpe_3 | 0.451 | 3.677 |
| 778 | 512 | 25 | 1dpe−1jet | 1dpe_4 | 0.451 | 4.084 |
| 778 | 512 | 25 | 1dpe−1jet | 1dpe_5 | 0.451 | 3.404 |
| 780 | 214 | 25 | 1fua−1jdi | 1fua_1 | 0.980 | 2.552 |
| 780 | 214 | 25 | 1fua−1jdi | 1fua_2 | 0.956 | 1.980 |
| 780 | 214 | 25 | 1fua−1jdi | 1fua_3 | 0.936 | 2.955 |
| 780 | 214 | 25 | 1fua−1jdi | 1fua_4 | 0.892 | 2.837 |
| 780 | 214 | 25 | 1fua−1jdi | 1fua_5 | 0.902 | 2.815 |
| 780 | 214 | 25 | 1fua−1jdi | 1fua_6 | 0.897 | 2.915 |
| 780 | 214 | 25 | 1fua−1jdi | 1fua_7 | 0.902 | 2.875 |
| 780 | 214 | 25 | 1fua−1jdi | 1fua_8 | 0.902 | 2.976 |
| 780 | 214 | 25 | 1fua−1jdi | 1fua_9 | 0.912 | 2.841 |
| 780 | 214 | 25 | 1fua−1jdi | 1fua_10 | 0.941 | 3.223 |
| 782 | 325 | 25 | 1b63−1h7s | 1b63_1 | 0.951 | 2.683 |
| 782 | 325 | 25 | 1b63−1h7s | 1b63_2 | 0.951 | 2.886 |
| 782 | 325 | 25 | 1b63−1h7s | 1b63_3 | 0.915 | 4.371 |
| 782 | 325 | 25 | 1b63−1h7s | 1b63_4 | 0.898 | 3.716 |
| 782 | 325 | 25 | 1b63−1h7s | 1b63_5 | 0.908 | 4.617 |
| 782 | 325 | 25 | 1b63−1h7s | 1b63_6 | 0.908 | 3.511 |
| 782 | 325 | 25 | 1b63−1h7s | 1b63_7 | 0.919 | 4.096 |
| 782 | 325 | 25 | 1b63−1h7s | 1b63_8 | 0.933 | 4.278 |
| 782 | 325 | 25 | 1b63−1h7s | 1b63_9 | 0.919 | 4.085 |
| 782 | 325 | 25 | 1b63−1h7s | 1b63_10 | 0.933 | 4.530 |
| 783 | 258 | 25 | 1i7d−1cy9 | 1i7d_1 | 0.829 | 2.286 |
| 783 | 258 | 25 | 1i7d−1cy9 | 1i7d_2 | 0.829 | 3.725 |
| 785 | 369 | 25 | 1oyc−2tmd | 1oyc_1 | 0.902 | 2.150 |
| 785 | 369 | 25 | 1oyc−2tmd | 1oyc_2 | 0.893 | 2.211 |
| 785 | 369 | 25 | 1oyc−2tmd | 1oyc_3 | 0.851 | 2.222 |
| 785 | 369 | 25 | 1oyc−2tmd | 1oyc_4 | 0.854 | 2.500 |
| 785 | 369 | 25 | 1oyc−2tmd | 1oyc_5 | 0.893 | 2.744 |
| 785 | 369 | 25 | 1oyc−2tmd | 1oyc_6 | 0.866 | 2.635 |
| 785 | 369 | 25 | 1oyc−2tmd | 1oyc_7 | 0.875 | 2.752 |
| 785 | 369 | 25 | 1oyc−2tmd | 1oyc_8 | 0.854 | 2.642 |
| 785 | 369 | 25 | 1oyc−2tmd | 1oyc_9 | 0.848 | 2.684 |
| 785 | 369 | 25 | 1oyc−2tmd | 1oyc_10 | 0.896 | 2.750 |
| 785 | 369 | 25 | 2tmd−1oyc | 2tmd_1 | 0.915 | 1.328 |
| 785 | 369 | 25 | 2tmd−1oyc | 2tmd_2 | 0.936 | 1.437 |
| 785 | 369 | 25 | 2tmd−1oyc | 2tmd_3 | 0.927 | 1.720 |
| 785 | 369 | 25 | 2tmd−1oyc | 2tmd_4 | 0.893 | 1.670 |
| 786 | 112 | 25 | 1ekg−1ew4 | 1ekg_1 | 0.953 | 1.423 |
| 786 | 112 | 25 | 1ekg−1ew4 | 1ekg_2 | 0.811 | 2.260 |
| 786 | 112 | 25 | 1ekg−1ew4 | 1ekg_3 | 0.774 | 2.655 |
| 786 | 112 | 25 | 1ekg−1ew4 | 1ekg_4 | 0.783 | 2.853 |
| 786 | 112 | 25 | 1ekg−1ew4 | 1ekg_5 | 0.906 | 2.819 |
| 786 | 112 | 25 | 1ekg−1ew4 | 1ekg_6 | 0.783 | 2.864 |
| 786 | 112 | 25 | 1ekg−1ew4 | 1ekg_7 | 0.792 | 3.135 |
| 786 | 112 | 25 | 1ekg−1ew4 | 1ekg_8 | 0.906 | 3.090 |
| 786 | 112 | 25 | 1ekg−1ew4 | 1ekg_9 | 0.849 | 2.985 |
| 786 | 112 | 25 | 1ekg−1ew4 | 1ekg_10 | 0.792 | 3.097 |
| 786 | 112 | 25 | 1ew4−1ekg | 1ew4_1 | 0.840 | 2.064 |
| 786 | 112 | 25 | 1ew4−1ekg | 1ew4_2 | 0.953 | 2.885 |
| 786 | 112 | 25 | 1ew4−1ekg | 1ew4_3 | 0.840 | 3.813 |
| 786 | 112 | 25 | 1ew4−1ekg | 1ew4_4 | 0.915 | 4.324 |
| 786 | 112 | 25 | 1ew4−1ekg | 1ew4_5 | 0.821 | 4.277 |
| 786 | 112 | 25 | 1ew4−1ekg | 1ew4_6 | 0.491 | 7.568 |
| 786 | 112 | 25 | 1ew4−1ekg | 1ew4_7 | 0.519 | 7.885 |
| 786 | 112 | 25 | 1ew4−1ekg | 1ew4_8 | 0.481 | 7.795 |
| 786 | 112 | 25 | 1ew4−1ekg | 1ew4_9 | 0.481 | 8.006 |
| 786 | 112 | 25 | 1ew4−1ekg | 1ew4_10 | 0.358 | 8.193 |
| 787 | 298 | 25 | 1bhg−1bgl | 1bhg_1 | 0.931 | 1.927 |
| 790 | 168 | 25 | 1bxd−1b3q | 1bxd_1 | 0.817 | 5.852 |
| 790 | 168 | 25 | 1bxd−1b3q | 1bxd_2 | 0.855 | 6.337 |
| 790 | 168 | 25 | 1bxd−1b3q | 1bxd_3 | 0.824 | 7.827 |
| 790 | 168 | 25 | 1bxd−1b3q | 1bxd_4 | 0.824 | 7.769 |
| 790 | 168 | 25 | 1bxd−1b3q | 1bxd_5 | 0.824 | 8.470 |
| 790 | 168 | 25 | 1bxd−1b3q | 1bxd_6 | 0.863 | 8.918 |
| 790 | 168 | 25 | 1bxd−1b3q | 1bxd_7 | 0.809 | 8.930 |
| 790 | 168 | 25 | 1bxd−1b3q | 1bxd_8 | 0.855 | 8.909 |
| 792 | 136 | 25 | 1gd5−1h6h | 1gd5_1 | 0.863 | 3.663 |
| 792 | 136 | 25 | 1gd5−1h6h | 1gd5_2 | 0.790 | 4.244 |
| 792 | 136 | 25 | 1gd5−1h6h | 1gd5_3 | 0.726 | 4.309 |
| 792 | 136 | 25 | 1gd5−1h6h | 1gd5_4 | 0.677 | 4.295 |
| 792 | 136 | 25 | 1gd5−1h6h | 1gd5_5 | 0.718 | 4.479 |
| 792 | 136 | 25 | 1gd5−1h6h | 1gd5_6 | 0.726 | 4.323 |
| 792 | 136 | 25 | 1gd5−1h6h | 1gd5_7 | 0.790 | 4.174 |
| 792 | 136 | 25 | 1gd5−1h6h | 1gd5_8 | 0.710 | 4.549 |
| 792 | 136 | 25 | 1gd5−1h6h | 1gd5_9 | 0.726 | 4.765 |
| 792 | 136 | 25 | 1gd5−1h6h | 1gd5_10 | 0.726 | 4.360 |
| 792 | 136 | 25 | 1h6h−1gd5 | 1h6h_1 | 0.944 | 4.367 |
| 792 | 136 | 25 | 1h6h−1gd5 | 1h6h_2 | 0.718 | 5.112 |

n: internal numbering; length of alignment; % sequence%ID (cf. Homstrad); refA−refB: reference alignment; altA: alternative snapshot for A; SP: sum−of−pairs score of alt. alignment w/r reference

| n | length | %ID | refA-refB | altA | SP | RMSD |
|---|---|---|---|---|---|---|
| 792 | 136 | 25 | 1h6h-1gd5 | 1h6h_3 | 0.685 | 6.566 |
| 792 | 136 | 25 | 1h6h-1gd5 | 1h6h_4 | 0.774 | 6.017 |
| 792 | 136 | 25 | 1h6h-1gd5 | 1h6h_5 | 0.565 | 6.782 |
| 792 | 136 | 25 | 1h6h-1gd5 | 1h6h_6 | 0.831 | 6.268 |
| 792 | 136 | 25 | 1h6h-1gd5 | 1h6h_7 | 0.839 | 6.261 |
| 792 | 136 | 25 | 1h6h-1gd5 | 1h6h_8 | 0.823 | 6.961 |
| 792 | 136 | 25 | 1h6h-1gd5 | 1h6h_9 | 0.742 | 7.512 |
| 792 | 136 | 25 | 1h6h-1gd5 | 1h6h_10 | 0.548 | 8.297 |
| 794 | 91 | 25 | 1flt-1pdg | 1flt_1 | 0.939 | 1.513 |
| 794 | 91 | 25 | 1flt-1pdg | 1flt_2 | 0.939 | 2.070 |
| 794 | 91 | 25 | 1flt-1pdg | 1flt_3 | 0.939 | 2.311 |
| 797 | 353 | 25 | 1k4g-1iq8 | 1k4g_1 | 0.958 | 2.336 |
| 797 | 353 | 25 | 1k4g-1iq8 | 1k4g_2 | 0.955 | 3.154 |
| 797 | 353 | 25 | 1k4g-1iq8 | 1k4g_3 | 0.934 | 3.356 |
| 797 | 353 | 25 | 1k4g-1iq8 | 1k4g_4 | 0.921 | 2.753 |
| 797 | 353 | 25 | 1k4g-1iq8 | 1k4g_5 | 0.897 | 3.540 |
| 797 | 353 | 25 | 1k4g-1iq8 | 1k4g_6 | 0.900 | 3.775 |
| 797 | 353 | 25 | 1k4g-1iq8 | 1k4g_7 | 0.894 | 3.781 |
| 797 | 353 | 25 | 1iq8-1k4g | 1iq8_1 | 0.991 | 1.843 |
| 799 | 254 | 25 | 1a48-1kut | 1a48_1 | 0.915 | 2.203 |
| 799 | 254 | 25 | 1a48-1kut | 1a48_2 | 0.915 | 2.298 |
| 799 | 254 | 25 | 1a48-1kut | 1a48_3 | 0.900 | 2.249 |
| 799 | 254 | 25 | 1a48-1kut | 1a48_4 | 0.896 | 2.587 |
| 799 | 254 | 25 | 1a48-1kut | 1a48_5 | 0.910 | 2.610 |
| 799 | 254 | 25 | 1a48-1kut | 1a48_6 | 0.900 | 2.601 |
| 799 | 254 | 25 | 1a48-1kut | 1a48_7 | 0.905 | 2.491 |
| 799 | 254 | 25 | 1a48-1kut | 1a48_8 | 0.886 | 2.830 |
| 799 | 254 | 25 | 1a48-1kut | 1a48_9 | 0.891 | 2.863 |
| 799 | 254 | 25 | 1a48-1kut | 1a48_10 | 0.881 | 2.863 |
| 802 | 196 | 25 | 1jcu-1hru | 1jcu_1 | 0.685 | 7.386 |
| 802 | 196 | 25 | 1jcu-1hru | 1jcu_2 | 0.730 | 9.167 |
| 802 | 196 | 25 | 1jcu-1hru | 1jcu_3 | 0.697 | 8.664 |
| 802 | 196 | 25 | 1jcu-1hru | 1jcu_4 | 0.635 | 7.862 |
| 802 | 196 | 25 | 1jcu-1hru | 1jcu_5 | 0.618 | 9.481 |
| 802 | 196 | 25 | 1jcu-1hru | 1jcu_6 | 0.663 | 8.272 |
| 808 | 291 | 24 | 1dhp-1nal | 1dhp_1 | 0.924 | 2.450 |
| 808 | 291 | 24 | 1dhp-1nal | 1dhp_2 | 0.893 | 2.924 |
| 808 | 291 | 24 | 1dhp-1nal | 1dhp_3 | 0.903 | 2.828 |
| 812 | 80 | 24 | 1vih-1khm | 1vih_1 | 0.857 | 3.564 |
| 812 | 80 | 24 | 1vih-1khm | 1vih_2 | 0.686 | 3.609 |
| 812 | 80 | 24 | 1vih-1khm | 1vih_3 | 0.714 | 3.305 |
| 812 | 80 | 24 | 1vih-1khm | 1vih_4 | 0.629 | 3.593 |
| 812 | 80 | 24 | 1vih-1khm | 1vih_5 | 0.629 | 3.453 |
| 812 | 80 | 24 | 1vih-1khm | 1vih_6 | 0.471 | 3.851 |
| 812 | 80 | 24 | 1vih-1khm | 1vih_7 | 0.657 | 3.540 |
| 812 | 80 | 24 | 1vih-1khm | 1vih_8 | 0.471 | 3.534 |
| 812 | 80 | 24 | 1vih-1khm | 1vih_9 | 0.529 | 3.324 |
| 812 | 80 | 24 | 1vih-1khm | 1vih_10 | 0.629 | 3.573 |
| 812 | 80 | 24 | 1khm-1vih | 1khm_1 | 0.886 | 4.696 |
| 812 | 80 | 24 | 1khm-1vih | 1khm_2 | 0.914 | 4.391 |
| 812 | 80 | 24 | 1khm-1vih | 1khm_3 | 0.943 | 4.496 |
| 812 | 80 | 24 | 1khm-1vih | 1khm_4 | 0.929 | 4.198 |
| 812 | 80 | 24 | 1khm-1vih | 1khm_5 | 0.914 | 4.463 |
| 812 | 80 | 24 | 1khm-1vih | 1khm_6 | 0.900 | 3.083 |
| 812 | 80 | 24 | 1khm-1vih | 1khm_7 | 0.886 | 4.839 |
| 812 | 80 | 24 | 1khm-1vih | 1khm_8 | 0.857 | 5.180 |
| 812 | 80 | 24 | 1khm-1vih | 1khm_9 | 0.914 | 4.851 |
| 812 | 80 | 24 | 1khm-1vih | 1khm_10 | 0.886 | 4.798 |
| 813 | 144 | 24 | 1gcu-1ofg | 1gcu_1 | 0.928 | 2.579 |
| 813 | 144 | 24 | 1gcu-1ofg | 1gcu_2 | 0.936 | 2.777 |
| 813 | 144 | 24 | 1gcu-1ofg | 1gcu_3 | 0.944 | 3.071 |
| 813 | 144 | 24 | 1gcu-1ofg | 1gcu_4 | 0.928 | 3.214 |
| 813 | 144 | 24 | 1gcu-1ofg | 1gcu_5 | 0.928 | 3.849 |
| 813 | 144 | 24 | 1gcu-1ofg | 1gcu_6 | 0.912 | 3.273 |
| 813 | 144 | 24 | 1gcu-1ofg | 1gcu_7 | 0.896 | 3.446 |
| 813 | 144 | 24 | 1gcu-1ofg | 1gcu_8 | 0.888 | 3.668 |
| 817 | 146 | 24 | 1vsd-1biz | 1vsd_1 | 0.935 | 3.134 |
| 817 | 146 | 24 | 1vsd-1biz | 1vsd_2 | 0.895 | 2.663 |
| 817 | 146 | 24 | 1vsd-1biz | 1vsd_3 | 0.952 | 3.055 |
| 817 | 146 | 24 | 1vsd-1biz | 1vsd_4 | 0.960 | 2.931 |
| 817 | 146 | 24 | 1vsd-1biz | 1vsd_5 | 0.944 | 3.387 |

| n | length | %ID | refA-refB | altA | SP | RMSD |
|---|---|---|---|---|---|---|
| 817 | 146 | 24 | 1vsd-1biz | 1vsd_6 | 0.935 | 3.159 |
| 817 | 146 | 24 | 1vsd-1biz | 1vsd_7 | 0.935 | 3.611 |
| 817 | 146 | 24 | 1vsd-1biz | 1vsd_8 | 0.935 | 3.360 |
| 817 | 146 | 24 | 1vsd-1biz | 1vsd_9 | 0.927 | 3.580 |
| 817 | 146 | 24 | 1vsd-1biz | 1vsd_10 | 0.887 | 3.700 |
| 819 | 168 | 24 | 1rl6-1jj2 | 1rl6_1 | 1.000 | 2.293 |
| 819 | 168 | 24 | 1rl6-1jj2 | 1rl6_2 | 1.000 | 2.483 |
| 819 | 168 | 24 | 1rl6-1jj2 | 1rl6_3 | 0.950 | 2.287 |
| 819 | 168 | 24 | 1rl6-1jj2 | 1rl6_4 | 0.913 | 3.462 |
| 819 | 168 | 24 | 1rl6-1jj2 | 1rl6_5 | 1.000 | 3.668 |
| 819 | 168 | 24 | 1rl6-1jj2 | 1rl6_6 | 0.938 | 4.039 |
| 819 | 168 | 24 | 1rl6-1jj2 | 1rl6_7 | 0.950 | 4.131 |
| 819 | 168 | 24 | 1rl6-1jj2 | 1rl6_8 | 0.913 | 3.857 |
| 821 | 326 | 24 | 1gln-1qtq | 1gln_1 | 0.923 | 3.077 |
| 821 | 326 | 24 | 1gln-1qtq | 1gln_2 | 0.956 | 4.047 |
| 821 | 326 | 24 | 1gln-1qtq | 1gln_3 | 0.978 | 3.190 |
| 824 | 89 | 24 | 1dcj-1jdq | 1dcj_1 | 0.905 | 2.428 |
| 824 | 89 | 24 | 1dcj-1jdq | 1dcj_2 | 0.919 | 2.502 |
| 824 | 89 | 24 | 1dcj-1jdq | 1dcj_3 | 0.946 | 3.187 |
| 824 | 89 | 24 | 1dcj-1jdq | 1dcj_4 | 0.946 | 2.734 |
| 824 | 89 | 24 | 1dcj-1jdq | 1dcj_5 | 0.932 | 2.884 |
| 824 | 89 | 24 | 1dcj-1jdq | 1dcj_6 | 0.743 | 2.875 |
| 824 | 89 | 24 | 1dcj-1jdq | 1dcj_7 | 0.932 | 3.051 |
| 824 | 89 | 24 | 1dcj-1jdq | 1dcj_8 | 0.932 | 2.945 |
| 824 | 89 | 24 | 1dcj-1jdq | 1dcj_9 | 0.932 | 3.027 |
| 824 | 89 | 24 | 1dcj-1jdq | 1dcj_10 | 0.730 | 3.083 |
| 824 | 89 | 24 | 1jdq-1dcj | 1jdq_1 | 0.784 | 7.658 |
| 824 | 89 | 24 | 1jdq-1dcj | 1jdq_2 | 0.770 | 7.147 |
| 824 | 89 | 24 | 1jdq-1dcj | 1jdq_3 | 0.527 | 8.278 |
| 824 | 89 | 24 | 1jdq-1dcj | 1jdq_4 | 0.568 | 9.461 |
| 824 | 89 | 24 | 1jdq-1dcj | 1jdq_5 | 0.554 | 9.100 |
| 824 | 89 | 24 | 1jdq-1dcj | 1jdq_6 | 0.676 | 8.161 |
| 824 | 89 | 24 | 1jdq-1dcj | 1jdq_7 | 0.689 | 8.247 |
| 824 | 89 | 24 | 1jdq-1dcj | 1jdq_8 | 0.581 | 9.268 |
| 824 | 89 | 24 | 1jdq-1dcj | 1jdq_9 | 0.676 | 8.542 |
| 824 | 89 | 24 | 1jdq-1dcj | 1jdq_10 | 0.514 | 9.184 |
| 830 | 137 | 23 | 1k6k-1khy | 1k6k_1 | 0.992 | 2.209 |
| 830 | 137 | 23 | 1k6k-1khy | 1k6k_2 | 0.906 | 2.629 |
| 830 | 137 | 23 | 1k6k-1khy | 1k6k_3 | 0.992 | 2.668 |
| 830 | 137 | 23 | 1k6k-1khy | 1k6k_4 | 0.992 | 2.453 |
| 830 | 137 | 23 | 1k6k-1khy | 1k6k_5 | 0.992 | 2.545 |
| 830 | 137 | 23 | 1k6k-1khy | 1k6k_6 | 0.992 | 2.883 |
| 830 | 137 | 23 | 1k6k-1khy | 1k6k_7 | 1.000 | 2.611 |
| 830 | 137 | 23 | 1k6k-1khy | 1k6k_8 | 1.000 | 2.515 |
| 830 | 137 | 23 | 1k6k-1khy | 1k6k_9 | 0.914 | 2.893 |
| 830 | 137 | 23 | 1k6k-1khy | 1k6k_10 | 1.000 | 2.844 |
| 836 | 248 | 23 | 1chk-1qgi | 1chk_1 | 0.906 | 1.965 |
| 836 | 248 | 23 | 1chk-1qgi | 1chk_2 | 0.897 | 2.590 |
| 836 | 248 | 23 | 1chk-1qgi | 1chk_3 | 0.901 | 2.771 |
| 836 | 248 | 23 | 1qgi-1chk | 1qgi_1 | 0.910 | 1.699 |
| 836 | 248 | 23 | 1qgi-1chk | 1qgi_2 | 0.888 | 2.130 |
| 836 | 248 | 23 | 1qgi-1chk | 1qgi_3 | 0.915 | 3.236 |
| 836 | 248 | 23 | 1qgi-1chk | 1qgi_4 | 0.848 | 3.850 |
| 836 | 248 | 23 | 1qgi-1chk | 1qgi_5 | 0.812 | 4.289 |
| 836 | 248 | 23 | 1qgi-1chk | 1qgi_6 | 0.762 | 4.437 |
| 836 | 248 | 23 | 1qgi-1chk | 1qgi_7 | 0.803 | 4.205 |
| 836 | 248 | 23 | 1qgi-1chk | 1qgi_8 | 0.767 | 4.364 |
| 836 | 248 | 23 | 1qgi-1chk | 1qgi_9 | 0.803 | 4.826 |
| 836 | 248 | 23 | 1qgi-1chk | 1qgi_10 | 0.691 | 5.312 |
| 839 | 167 | 23 | 1lki-1evs | 1lki_1 | 0.893 | 1.826 |
| 839 | 167 | 23 | 1lki-1evs | 1lki_2 | 0.886 | 1.691 |
| 839 | 167 | 23 | 1lki-1evs | 1lki_3 | 0.852 | 1.782 |
| 839 | 167 | 23 | 1lki-1evs | 1lki_4 | 0.893 | 1.717 |
| 839 | 167 | 23 | 1lki-1evs | 1lki_5 | 0.852 | 1.623 |
| 839 | 167 | 23 | 1lki-1evs | 1lki_6 | 0.933 | 1.922 |
| 840 | 147 | 23 | 1a6j-1a3a | 1a6j_1 | 0.915 | 1.366 |
| 840 | 147 | 23 | 1a6j-1a3a | 1a6j_2 | 0.908 | 1.532 |
| 840 | 147 | 23 | 1a6j-1a3a | 1a6j_3 | 0.972 | 2.040 |
| 840 | 147 | 23 | 1a6j-1a3a | 1a6j_4 | 0.915 | 2.323 |
| 840 | 147 | 23 | 1a6j-1a3a | 1a6j_5 | 0.965 | 2.221 |
| 840 | 147 | 23 | 1a6j-1a3a | 1a6j_6 | 0.936 | 2.495 |

| n | length | %ID | refA-refB | altA | SP | RMSD |
|---|---|---|---|---|---|---|
| 840 | 147 | 23 | 1a6j-1a3a | 1a6j_7 | 0.943 | 2.276 |
| 840 | 147 | 23 | 1a6j-1a3a | 1a6j_8 | 0.979 | 2.097 |
| 840 | 147 | 23 | 1a6j-1a3a | 1a6j_9 | 0.950 | 2.191 |
| 840 | 147 | 23 | 1a6j-1a3a | 1a6j_10 | 0.943 | 2.322 |
| 844 | 324 | 23 | 1rkd-1bx4 | 1rkd_1 | 0.929 | 1.916 |
| 844 | 324 | 23 | 1rkd-1bx4 | 1rkd_2 | 0.925 | 2.191 |
| 844 | 324 | 23 | 1rkd-1bx4 | 1rkd_3 | 0.915 | 2.823 |
| 844 | 324 | 23 | 1rkd-1bx4 | 1rkd_4 | 0.932 | 3.208 |
| 844 | 324 | 23 | 1rkd-1bx4 | 1rkd_5 | 0.892 | 3.101 |
| 844 | 324 | 23 | 1rkd-1bx4 | 1rkd_6 | 0.939 | 3.302 |
| 844 | 324 | 23 | 1rkd-1bx4 | 1rkd_7 | 0.953 | 3.176 |
| 844 | 324 | 23 | 1rkd-1bx4 | 1rkd_8 | 0.915 | 2.581 |
| 844 | 324 | 23 | 1rkd-1bx4 | 1rkd_9 | 0.895 | 2.865 |
| 844 | 324 | 23 | 1rkd-1bx4 | 1rkd_10 | 0.912 | 2.732 |
| 844 | 324 | 23 | 1bx4-1rkd | 1bx4_1 | 0.898 | 1.716 |
| 844 | 324 | 23 | 1bx4-1rkd | 1bx4_2 | 0.902 | 1.770 |
| 844 | 324 | 23 | 1bx4-1rkd | 1bx4_3 | 0.892 | 1.987 |
| 844 | 324 | 23 | 1bx4-1rkd | 1bx4_4 | 0.878 | 2.068 |
| 844 | 324 | 23 | 1bx4-1rkd | 1bx4_5 | 0.861 | 2.057 |
| 844 | 324 | 23 | 1bx4-1rkd | 1bx4_6 | 0.817 | 2.274 |
| 844 | 324 | 23 | 1bx4-1rkd | 1bx4_7 | 0.844 | 2.331 |
| 844 | 324 | 23 | 1bx4-1rkd | 1bx4_8 | 0.861 | 2.417 |
| 844 | 324 | 23 | 1bx4-1rkd | 1bx4_9 | 0.841 | 2.488 |
| 844 | 324 | 23 | 1bx4-1rkd | 1bx4_10 | 0.837 | 2.476 |
| 851 | 445 | 22 | 1l5j-1c96 | 1l5j_1 | 0.968 | 1.580 |
| 857 | 286 | 22 | 1fo6-1ems | 1fo6_1 | 0.970 | 1.615 |
| 857 | 286 | 22 | 1fo6-1ems | 1fo6_2 | 0.973 | 1.720 |
| 857 | 286 | 22 | 1fo6-1ems | 1fo6_3 | 0.924 | 1.745 |
| 857 | 286 | 22 | 1fo6-1ems | 1fo6_4 | 0.989 | 1.939 |
| 858 | 180 | 22 | 1bpy-1jqr | 1bpy_1 | 0.880 | 2.497 |
| 858 | 180 | 22 | 1bpy-1jqr | 1bpy_2 | 0.795 | 3.071 |
| 858 | 180 | 22 | 1bpy-1jqr | 1bpy_3 | 0.867 | 2.844 |
| 858 | 180 | 22 | 1bpy-1jqr | 1bpy_4 | 0.849 | 3.221 |
| 858 | 180 | 22 | 1bpy-1jqr | 1bpy_5 | 0.873 | 3.176 |
| 858 | 180 | 22 | 1bpy-1jqr | 1bpy_6 | 0.928 | 3.297 |
| 858 | 180 | 22 | 1bpy-1jqr | 1bpy_7 | 0.898 | 3.796 |
| 858 | 180 | 22 | 1bpy-1jqr | 1bpy_8 | 0.849 | 3.540 |
| 858 | 180 | 22 | 1bpy-1jqr | 1bpy_9 | 0.873 | 3.504 |
| 858 | 180 | 22 | 1bpy-1jqr | 1bpy_10 | 0.928 | 3.565 |
| 858 | 180 | 22 | 1jqr-1bpy | 1jqr_1 | 0.892 | 2.870 |
| 858 | 180 | 22 | 1jqr-1bpy | 1jqr_2 | 0.934 | 3.433 |
| 858 | 180 | 22 | 1jqr-1bpy | 1jqr_3 | 0.861 | 3.400 |
| 858 | 180 | 22 | 1jqr-1bpy | 1jqr_4 | 0.488 | 4.338 |
| 858 | 180 | 22 | 1jqr-1bpy | 1jqr_5 | 0.633 | 3.905 |
| 858 | 180 | 22 | 1jqr-1bpy | 1jqr_6 | 0.608 | 4.490 |
| 858 | 180 | 22 | 1jqr-1bpy | 1jqr_7 | 0.428 | 5.848 |
| 858 | 180 | 22 | 1jqr-1bpy | 1jqr_8 | 0.416 | 5.929 |
| 858 | 180 | 22 | 1jqr-1bpy | 1jqr_9 | 0.488 | 5.885 |
| 858 | 180 | 22 | 1jqr-1bpy | 1jqr_10 | 0.645 | 5.197 |
| 859 | 94 | 22 | 1fsh-1o7f | 1fsh_1 | 1.000 | 2.720 |
| 859 | 94 | 22 | 1fsh-1o7f | 1fsh_2 | 0.967 | 2.621 |
| 859 | 94 | 22 | 1fsh-1o7f | 1fsh_3 | 0.844 | 3.362 |
| 859 | 94 | 22 | 1fsh-1o7f | 1fsh_4 | 0.944 | 3.355 |
| 859 | 94 | 22 | 1fsh-1o7f | 1fsh_5 | 0.967 | 3.873 |
| 859 | 94 | 22 | 1fsh-1o7f | 1fsh_6 | 0.789 | 3.867 |
| 859 | 94 | 22 | 1fsh-1o7f | 1fsh_7 | 0.733 | 4.238 |
| 859 | 94 | 22 | 1fsh-1o7f | 1fsh_8 | 0.733 | 4.058 |
| 859 | 94 | 22 | 1fsh-1o7f | 1fsh_9 | 0.911 | 4.160 |
| 859 | 94 | 22 | 1fsh-1o7f | 1fsh_10 | 0.933 | 4.371 |
| 861 | 396 | 22 | 1qay-1dqa | 1qay_1 | 0.781 | 1.781 |
| 861 | 396 | 22 | 1qay-1dqa | 1qay_2 | 0.975 | 1.803 |
| 861 | 396 | 22 | 1qay-1dqa | 1qay_3 | 0.961 | 2.043 |
| 861 | 396 | 22 | 1qay-1dqa | 1qay_4 | 0.857 | 2.213 |
| 861 | 396 | 22 | 1qay-1dqa | 1qay_5 | 0.831 | 2.575 |
| 861 | 396 | 22 | 1qay-1dqa | 1qay_6 | 0.784 | 2.676 |
| 861 | 396 | 22 | 1dqa-1qay | 1dqa_1 | 0.871 | 1.755 |
| 863 | 54 | 22 | 1ihv-1c1a | 1ihv_1 | 0.863 | 1.701 |
| 863 | 54 | 22 | 1ihv-1c1a | 1ihv_2 | 0.980 | 1.933 |
| 863 | 54 | 22 | 1ihv-1c1a | 1ihv_3 | 0.902 | 2.518 |
| 863 | 54 | 22 | 1ihv-1c1a | 1ihv_4 | 0.863 | 2.303 |
| 863 | 54 | 22 | 1ihv-1c1a | 1ihv_5 | 0.882 | 2.080 |

| n | length | %ID | refA−refB | altA | SP | RMSD |
|---|---|---|---|---|---|---|
| 863 | 54 | 22 | 1ihv−1c1a | 1ihv_6 | 0.863 | 2.241 |
| 863 | 54 | 22 | 1ihv−1c1a | 1ihv_7 | 0.882 | 1.935 |
| 863 | 54 | 22 | 1ihv−1c1a | 1ihv_8 | 0.843 | 2.383 |
| 863 | 54 | 22 | 1ihv−1c1a | 1ihv_9 | 0.863 | 2.107 |
| 863 | 54 | 22 | 1ihv−1c1a | 1ihv_10 | 0.902 | 2.083 |
| 867 | 147 | 22 | 1tum−1f3y | 1tum_1 | 0.808 | 3.050 |
| 867 | 147 | 22 | 1tum−1f3y | 1tum_2 | 0.600 | 3.156 |
| 867 | 147 | 22 | 1tum−1f3y | 1tum_3 | 0.712 | 3.605 |
| 867 | 147 | 22 | 1tum−1f3y | 1tum_4 | 0.528 | 4.443 |
| 867 | 147 | 22 | 1tum−1f3y | 1tum_5 | 0.344 | 4.631 |
| 867 | 147 | 22 | 1tum−1f3y | 1tum_6 | 0.576 | 5.070 |
| 867 | 147 | 22 | 1tum−1f3y | 1tum_7 | 0.456 | 5.298 |
| 867 | 147 | 22 | 1tum−1f3y | 1tum_8 | 0.344 | 5.020 |
| 867 | 147 | 22 | 1tum−1f3y | 1tum_9 | 0.344 | 5.792 |
| 867 | 147 | 22 | 1tum−1f3y | 1tum_10 | 0.264 | 5.453 |
| 867 | 147 | 22 | 1f3y−1tum | 1f3y_1 | 0.928 | 3.878 |
| 867 | 147 | 22 | 1f3y−1tum | 1f3y_2 | 0.888 | 3.809 |
| 867 | 147 | 22 | 1f3y−1tum | 1f3y_3 | 0.896 | 4.446 |
| 867 | 147 | 22 | 1f3y−1tum | 1f3y_4 | 0.848 | 5.441 |
| 867 | 147 | 22 | 1f3y−1tum | 1f3y_5 | 0.712 | 5.433 |
| 867 | 147 | 22 | 1f3y−1tum | 1f3y_6 | 0.816 | 5.297 |
| 867 | 147 | 22 | 1f3y−1tum | 1f3y_7 | 0.736 | 5.251 |
| 867 | 147 | 22 | 1f3y−1tum | 1f3y_8 | 0.880 | 5.386 |
| 867 | 147 | 22 | 1f3y−1tum | 1f3y_9 | 0.856 | 5.359 |
| 867 | 147 | 22 | 1f3y−1tum | 1f3y_10 | 0.824 | 5.343 |
| 869 | 252 | 22 | 1dbt−1dqw | 1dbt_1 | 0.819 | 1.979 |
| 869 | 252 | 22 | 1dbt−1dqw | 1dbt_2 | 0.819 | 2.169 |
| 869 | 252 | 22 | 1dqw−1dbt | 1dqw_1 | 0.847 | 1.747 |
| 869 | 252 | 22 | 1dqw−1dbt | 1dqw_2 | 0.829 | 2.181 |
| 870 | 498 | 22 | 2pgi−1dqr | 2pgi_1 | 0.814 | 5.421 |
| 870 | 498 | 22 | 2pgi−1dqr | 2pgi_2 | 0.801 | 5.399 |
| 870 | 498 | 22 | 2pgi−1dqr | 2pgi_3 | 0.801 | 5.536 |
| 878 | 176 | 22 | 154l−1qsa | 154l_1 | 0.865 | 2.036 |
| 878 | 176 | 22 | 154l−1qsa | 154l_2 | 0.921 | 2.107 |
| 878 | 176 | 22 | 154l−1qsa | 154l_3 | 0.889 | 2.066 |
| 878 | 176 | 22 | 154l−1qsa | 154l_4 | 0.857 | 2.291 |
| 878 | 176 | 22 | 154l−1qsa | 154l_5 | 0.921 | 2.382 |
| 878 | 176 | 22 | 154l−1qsa | 154l_6 | 0.881 | 2.291 |
| 878 | 176 | 22 | 154l−1qsa | 154l_7 | 0.897 | 2.283 |
| 878 | 176 | 22 | 154l−1qsa | 154l_8 | 0.889 | 2.662 |
| 878 | 176 | 22 | 154l−1qsa | 154l_9 | 0.897 | 2.360 |
| 878 | 176 | 22 | 154l−1qsa | 154l_10 | 0.841 | 2.399 |
| 884 | 100 | 21 | 1ixd−1lpl | 1ixd_1 | 0.882 | 5.408 |
| 884 | 100 | 21 | 1ixd−1lpl | 1ixd_2 | 0.796 | 6.096 |
| 884 | 100 | 21 | 1ixd−1lpl | 1ixd_3 | 0.731 | 8.277 |
| 884 | 100 | 21 | 1ixd−1lpl | 1ixd_4 | 0.720 | 7.463 |
| 884 | 100 | 21 | 1ixd−1lpl | 1ixd_5 | 0.817 | 7.924 |
| 884 | 100 | 21 | 1ixd−1lpl | 1ixd_6 | 0.828 | 7.678 |
| 884 | 100 | 21 | 1ixd−1lpl | 1ixd_7 | 0.817 | 7.830 |
| 884 | 100 | 21 | 1ixd−1lpl | 1ixd_8 | 0.828 | 8.308 |
| 884 | 100 | 21 | 1ixd−1lpl | 1ixd_9 | 0.817 | 8.528 |
| 884 | 100 | 21 | 1ixd−1lpl | 1ixd_10 | 0.817 | 8.430 |
| 884 | 100 | 21 | 1lpl−1ixd | 1lpl_1 | 0.935 | 3.525 |
| 884 | 100 | 21 | 1lpl−1ixd | 1lpl_2 | 0.839 | 4.415 |
| 884 | 100 | 21 | 1lpl−1ixd | 1lpl_3 | 0.839 | 4.463 |
| 884 | 100 | 21 | 1lpl−1ixd | 1lpl_4 | 0.849 | 4.561 |
| 884 | 100 | 21 | 1lpl−1ixd | 1lpl_5 | 0.839 | 4.450 |
| 884 | 100 | 21 | 1lpl−1ixd | 1lpl_6 | 0.849 | 4.704 |
| 884 | 100 | 21 | 1lpl−1ixd | 1lpl_7 | 0.828 | 4.782 |
| 884 | 100 | 21 | 1lpl−1ixd | 1lpl_8 | 0.828 | 4.603 |
| 884 | 100 | 21 | 1lpl−1ixd | 1lpl_9 | 0.839 | 4.613 |
| 884 | 100 | 21 | 1lpl−1ixd | 1lpl_10 | 0.839 | 4.759 |
| 885 | 202 | 21 | 1cex−1bs9 | 1cex_1 | 0.923 | 1.850 |
| 885 | 202 | 21 | 1cex−1bs9 | 1cex_2 | 0.863 | 1.964 |
| 885 | 202 | 21 | 1cex−1bs9 | 1cex_3 | 0.857 | 2.287 |
| 885 | 202 | 21 | 1cex−1bs9 | 1cex_4 | 0.875 | 2.634 |
| 885 | 202 | 21 | 1cex−1bs9 | 1cex_5 | 0.827 | 2.863 |
| 885 | 202 | 21 | 1cex−1bs9 | 1cex_6 | 0.827 | 2.804 |
| 885 | 202 | 21 | 1cex−1bs9 | 1cex_7 | 0.750 | 2.892 |
| 885 | 202 | 21 | 1cex−1bs9 | 1cex_8 | 0.839 | 3.111 |
| 885 | 202 | 21 | 1cex−1bs9 | 1cex_9 | 0.845 | 3.046 |
| 885 | 202 | 21 | 1cex−1bs9 | 1cex_10 | 0.845 | 3.152 |
| 885 | 202 | 21 | 1bs9−1cex | 1bs9_1 | 0.958 | 2.688 |
| 885 | 202 | 21 | 1bs9−1cex | 1bs9_2 | 0.798 | 2.716 |
| 885 | 202 | 21 | 1bs9−1cex | 1bs9_3 | 0.875 | 2.940 |
| 885 | 202 | 21 | 1bs9−1cex | 1bs9_4 | 0.893 | 2.702 |
| 885 | 202 | 21 | 1bs9−1cex | 1bs9_5 | 0.821 | 2.896 |
| 885 | 202 | 21 | 1bs9−1cex | 1bs9_6 | 0.815 | 3.250 |
| 885 | 202 | 21 | 1bs9−1cex | 1bs9_7 | 0.804 | 3.298 |
| 885 | 202 | 21 | 1bs9−1cex | 1bs9_8 | 0.887 | 3.363 |
| 885 | 202 | 21 | 1bs9−1cex | 1bs9_9 | 0.881 | 3.601 |
| 885 | 202 | 21 | 1bs9−1cex | 1bs9_10 | 0.881 | 3.623 |
| 887 | 363 | 21 | 1b9i−1jg8 | 1b9i_1 | 0.923 | 1.853 |
| 887 | 363 | 21 | 1b9i−1jg8 | 1b9i_2 | 0.907 | 2.076 |
| 887 | 363 | 21 | 1b9i−1jg8 | 1b9i_3 | 0.913 | 2.285 |
| 887 | 363 | 21 | 1b9i−1jg8 | 1b9i_4 | 0.869 | 2.653 |
| 887 | 363 | 21 | 1b9i−1jg8 | 1b9i_5 | 0.878 | 2.821 |
| 887 | 363 | 21 | 1b9i−1jg8 | 1b9i_6 | 0.837 | 3.450 |
| 887 | 363 | 21 | 1b9i−1jg8 | 1b9i_7 | 0.865 | 3.972 |
| 887 | 363 | 21 | 1b9i−1jg8 | 1b9i_8 | 0.862 | 4.145 |
| 887 | 363 | 21 | 1b9i−1jg8 | 1b9i_9 | 0.830 | 4.436 |
| 887 | 363 | 21 | 1b9i−1jg8 | 1b9i_10 | 0.776 | 4.065 |
| 891 | 245 | 21 | 1thf−1qo2 | 1thf_1 | 0.914 | 1.827 |
| 891 | 245 | 21 | 1thf−1qo2 | 1thf_2 | 0.909 | 2.038 |
| 891 | 245 | 21 | 1thf−1qo2 | 1thf_3 | 0.936 | 2.495 |
| 891 | 245 | 21 | 1thf−1qo2 | 1thf_4 | 0.909 | 2.474 |
| 891 | 245 | 21 | 1thf−1qo2 | 1thf_5 | 0.932 | 2.573 |
| 891 | 245 | 21 | 1thf−1qo2 | 1thf_6 | 0.918 | 2.599 |
| 891 | 245 | 21 | 1thf−1qo2 | 1thf_7 | 0.905 | 2.586 |
| 891 | 245 | 21 | 1thf−1qo2 | 1thf_8 | 0.891 | 2.634 |
| 891 | 245 | 21 | 1thf−1qo2 | 1thf_9 | 0.886 | 2.819 |
| 891 | 245 | 21 | 1thf−1qo2 | 1thf_10 | 0.873 | 2.830 |
| 894 | 70 | 21 | 1ej5−1f3m | 1ej5_1 | 0.224 | 3.465 |
| 894 | 70 | 21 | 1ej5−1f3m | 1ej5_2 | 0.209 | 3.695 |
| 894 | 70 | 21 | 1ej5−1f3m | 1ej5_3 | 0.209 | 3.846 |
| 894 | 70 | 21 | 1ej5−1f3m | 1ej5_4 | 0.209 | 4.267 |
| 894 | 70 | 21 | 1ej5−1f3m | 1ej5_5 | 0.567 | 3.962 |
| 894 | 70 | 21 | 1ej5−1f3m | 1ej5_6 | 0.254 | 4.606 |
| 894 | 70 | 21 | 1ej5−1f3m | 1ej5_7 | 0.194 | 4.472 |
| 894 | 70 | 21 | 1ej5−1f3m | 1ej5_8 | 0.627 | 5.025 |
| 894 | 70 | 21 | 1ej5−1f3m | 1ej5_9 | 0.687 | 4.928 |
| 894 | 70 | 21 | 1ej5−1f3m | 1ej5_10 | 0.672 | 5.083 |
| 898 | 132 | 21 | 1jw3−1j5u | 1jw3_1 | 0.984 | 3.070 |
| 898 | 132 | 21 | 1jw3−1j5u | 1jw3_2 | 0.968 | 3.336 |
| 898 | 132 | 21 | 1jw3−1j5u | 1jw3_3 | 0.904 | 3.297 |
| 898 | 132 | 21 | 1jw3−1j5u | 1jw3_4 | 0.704 | 3.325 |
| 898 | 132 | 21 | 1jw3−1j5u | 1jw3_5 | 0.896 | 3.361 |
| 898 | 132 | 21 | 1jw3−1j5u | 1jw3_6 | 0.816 | 3.579 |
| 898 | 132 | 21 | 1jw3−1j5u | 1jw3_7 | 0.824 | 3.777 |
| 898 | 132 | 21 | 1jw3−1j5u | 1jw3_8 | 0.824 | 3.755 |
| 898 | 132 | 21 | 1jw3−1j5u | 1jw3_9 | 0.864 | 4.026 |
| 898 | 132 | 21 | 1jw3−1j5u | 1jw3_10 | 0.728 | 4.045 |
| 900 | 73 | 21 | 1sro−1ah9 | 1sro_1 | 0.765 | 3.682 |
| 900 | 73 | 21 | 1sro−1ah9 | 1sro_2 | 0.721 | 3.329 |
| 900 | 73 | 21 | 1sro−1ah9 | 1sro_3 | 0.706 | 3.670 |
| 900 | 73 | 21 | 1sro−1ah9 | 1sro_4 | 0.691 | 3.821 |
| 900 | 73 | 21 | 1sro−1ah9 | 1sro_5 | 0.897 | 3.234 |
| 900 | 73 | 21 | 1sro−1ah9 | 1sro_6 | 0.691 | 3.233 |
| 900 | 73 | 21 | 1sro−1ah9 | 1sro_7 | 0.574 | 3.356 |
| 900 | 73 | 21 | 1sro−1ah9 | 1sro_8 | 0.559 | 3.590 |
| 900 | 73 | 21 | 1sro−1ah9 | 1sro_9 | 0.706 | 3.327 |
| 900 | 73 | 21 | 1sro−1ah9 | 1sro_10 | 0.647 | 3.390 |
| 900 | 73 | 21 | 1ah9−1sro | 1ah9_1 | 0.794 | 3.031 |
| 900 | 73 | 21 | 1ah9−1sro | 1ah9_2 | 0.794 | 2.647 |
| 900 | 73 | 21 | 1ah9−1sro | 1ah9_3 | 0.809 | 2.834 |
| 900 | 73 | 21 | 1ah9−1sro | 1ah9_4 | 0.941 | 2.552 |
| 900 | 73 | 21 | 1ah9−1sro | 1ah9_5 | 0.779 | 2.578 |
| 900 | 73 | 21 | 1ah9−1sro | 1ah9_6 | 0.809 | 2.910 |
| 900 | 73 | 21 | 1ah9−1sro | 1ah9_7 | 0.809 | 2.771 |
| 900 | 73 | 21 | 1ah9−1sro | 1ah9_8 | 0.809 | 3.031 |
| 900 | 73 | 21 | 1ah9−1sro | 1ah9_9 | 0.809 | 2.981 |
| 900 | 73 | 21 | 1ah9−1sro | 1ah9_10 | 0.735 | 3.265 |
| 901 | 102 | 21 | 2spc−1aj3 | 2spc_1 | 0.226 | 3.087 |
| 901 | 102 | 21 | 2spc−1aj3 | 2spc_2 | 0.935 | 5.964 |
| 901 | 102 | 21 | 2spc−1aj3 | 2spc_3 | 0.269 | 5.683 |
| 901 | 102 | 21 | 2spc−1aj3 | 2spc_4 | 0.688 | 6.410 |
| 901 | 102 | 21 | 1aj3−2spc | 1aj3_1 | 0.720 | 2.734 |
| 901 | 102 | 21 | 1aj3−2spc | 1aj3_2 | 0.871 | 3.276 |
| 901 | 102 | 21 | 1aj3−2spc | 1aj3_3 | 0.516 | 3.494 |
| 901 | 102 | 21 | 1aj3−2spc | 1aj3_4 | 0.914 | 3.983 |
| 901 | 102 | 21 | 1aj3−2spc | 1aj3_5 | 0.828 | 4.263 |
| 901 | 102 | 21 | 1aj3−2spc | 1aj3_6 | 0.742 | 4.177 |
| 901 | 102 | 21 | 1aj3−2spc | 1aj3_7 | 0.495 | 4.296 |
| 901 | 102 | 21 | 1aj3−2spc | 1aj3_8 | 0.484 | 5.465 |
| 901 | 102 | 21 | 1aj3−2spc | 1aj3_9 | 0.312 | 6.276 |
| 901 | 102 | 21 | 1aj3−2spc | 1aj3_10 | 0.269 | 6.725 |
| 903 | 138 | 21 | 1eb0−1gmw | 1eb0_1 | 0.977 | 3.065 |
| 903 | 138 | 21 | 1eb0−1gmw | 1eb0_2 | 0.955 | 2.793 |
| 903 | 138 | 21 | 1eb0−1gmw | 1eb0_3 | 0.955 | 3.374 |
| 903 | 138 | 21 | 1eb0−1gmw | 1eb0_4 | 0.962 | 3.648 |
| 903 | 138 | 21 | 1eb0−1gmw | 1eb0_5 | 0.955 | 3.106 |
| 903 | 138 | 21 | 1eb0−1gmw | 1eb0_6 | 0.955 | 3.325 |
| 903 | 138 | 21 | 1eb0−1gmw | 1eb0_7 | 0.985 | 3.767 |
| 903 | 138 | 21 | 1eb0−1gmw | 1eb0_8 | 0.835 | 4.407 |
| 903 | 138 | 21 | 1eb0−1gmw | 1eb0_9 | 0.857 | 4.407 |
| 903 | 138 | 21 | 1eb0−1gmw | 1eb0_10 | 0.835 | 4.128 |
| 905 | 224 | 20 | 1i6a−1al3 | 1i6a_1 | 0.955 | 2.127 |
| 905 | 224 | 20 | 1i6a−1al3 | 1i6a_2 | 0.970 | 3.469 |
| 905 | 224 | 20 | 1i6a−1al3 | 1i6a_3 | 0.970 | 2.951 |
| 905 | 224 | 20 | 1i6a−1al3 | 1i6a_4 | 0.916 | 3.135 |
| 905 | 224 | 20 | 1i6a−1al3 | 1i6a_5 | 0.832 | 4.156 |
| 905 | 224 | 20 | 1i6a−1al3 | 1i6a_6 | 0.851 | 3.838 |
| 905 | 224 | 20 | 1i6a−1al3 | 1i6a_7 | 0.827 | 4.045 |
| 905 | 224 | 20 | 1i6a−1al3 | 1i6a_8 | 0.842 | 4.559 |
| 905 | 224 | 20 | 1i6a−1al3 | 1i6a_9 | 0.916 | 5.084 |
| 905 | 224 | 20 | 1i6a−1al3 | 1i6a_10 | 0.847 | 4.651 |
| 905 | 224 | 20 | 1al3−1i6a | 1al3_1 | 0.891 | 2.098 |
| 905 | 224 | 20 | 1al3−1i6a | 1al3_2 | 0.921 | 2.538 |
| 905 | 224 | 20 | 1al3−1i6a | 1al3_3 | 0.896 | 2.385 |
| 905 | 224 | 20 | 1al3−1i6a | 1al3_4 | 0.955 | 2.558 |
| 905 | 224 | 20 | 1al3−1i6a | 1al3_5 | 0.876 | 3.042 |
| 905 | 224 | 20 | 1al3−1i6a | 1al3_6 | 0.916 | 2.833 |
| 905 | 224 | 20 | 1al3−1i6a | 1al3_7 | 0.891 | 2.524 |
| 905 | 224 | 20 | 1al3−1i6a | 1al3_8 | 0.931 | 2.837 |
| 905 | 224 | 20 | 1al3−1i6a | 1al3_9 | 0.842 | 2.990 |
| 905 | 224 | 20 | 1al3−1i6a | 1al3_10 | 0.921 | 2.875 |
| 907 | 206 | 20 | 1by1−1dbh | 1by1_1 | 0.675 | 5.162 |
| 907 | 206 | 20 | 1by1−1dbh | 1by1_2 | 0.639 | 6.164 |
| 907 | 206 | 20 | 1by1−1dbh | 1by1_3 | 0.550 | 6.696 |
| 907 | 206 | 20 | 1by1−1dbh | 1by1_4 | 0.597 | 6.495 |
| 907 | 206 | 20 | 1by1−1dbh | 1by1_5 | 0.696 | 7.424 |
| 907 | 206 | 20 | 1by1−1dbh | 1by1_6 | 0.503 | 8.376 |
| 907 | 206 | 20 | 1by1−1dbh | 1by1_7 | 0.618 | 8.339 |
| 907 | 206 | 20 | 1by1−1dbh | 1by1_8 | 0.618 | 8.503 |
| 907 | 206 | 20 | 1by1−1dbh | 1by1_9 | 0.597 | 8.998 |
| 907 | 206 | 20 | 1by1−1dbh | 1by1_10 | 0.545 | 8.858 |
| 909 | 120 | 20 | 1b9l−1dhn | 1b9l_1 | 0.921 | 1.755 |
| 909 | 120 | 20 | 1b9l−1dhn | 1b9l_2 | 0.956 | 2.228 |
| 909 | 120 | 20 | 1b9l−1dhn | 1b9l_3 | 0.895 | 2.697 |
| 909 | 120 | 20 | 1b9l−1dhn | 1b9l_4 | 0.825 | 2.220 |
| 909 | 120 | 20 | 1b9l−1dhn | 1b9l_5 | 0.851 | 2.587 |
| 909 | 120 | 20 | 1b9l−1dhn | 1b9l_6 | 0.886 | 2.409 |
| 909 | 120 | 20 | 1b9l−1dhn | 1b9l_7 | 0.877 | 2.755 |
| 909 | 120 | 20 | 1b9l−1dhn | 1b9l_8 | 0.842 | 2.497 |
| 909 | 120 | 20 | 1dhn−1b9l | 1dhn_1 | 0.851 | 3.023 |
| 909 | 120 | 20 | 1dhn−1b9l | 1dhn_2 | 0.886 | 3.262 |
| 909 | 120 | 20 | 1dhn−1b9l | 1dhn_3 | 0.833 | 3.591 |
| 909 | 120 | 20 | 1dhn−1b9l | 1dhn_4 | 0.886 | 3.805 |
| 909 | 120 | 20 | 1dhn−1b9l | 1dhn_5 | 0.833 | 3.816 |
| 909 | 120 | 20 | 1dhn−1b9l | 1dhn_6 | 0.833 | 4.600 |
| 909 | 120 | 20 | 1dhn−1b9l | 1dhn_7 | 0.825 | 4.921 |
| 909 | 120 | 20 | 1dhn−1b9l | 1dhn_8 | 0.877 | 4.919 |
| 909 | 120 | 20 | 1dhn−1b9l | 1dhn_9 | 0.667 | 4.358 |

n: internal numbering; length of alignment; % sequence%ID (cf. Homstrad); refA−refB: reference alignment; altA: alternative snapshot for A; SP: sum−of−pairs score of alt. alignment w/r reference 

| n | length | %ID | refA−refB | altA | SP | RMSD |
|---|---|---|---|---|---|---|
| 909 | 120 | 20 | 1dhn−1b9l | 1dhn_10 | 0.825 | 5.423 |
| 911 | 218 | 20 | 1mun−2abk | 1mun_1 | 0.956 | 2.433 |
| 911 | 218 | 20 | 1mun−2abk | 1mun_2 | 0.956 | 2.381 |
| 911 | 218 | 20 | 1mun−2abk | 1mun_3 | 0.908 | 3.078 |
| 911 | 218 | 20 | 1mun−2abk | 1mun_4 | 0.951 | 2.887 |
| 911 | 218 | 20 | 1mun−2abk | 1mun_5 | 0.927 | 3.057 |
| 911 | 218 | 20 | 1mun−2abk | 1mun_6 | 0.553 | 3.065 |
| 911 | 218 | 20 | 1mun−2abk | 1mun_7 | 0.951 | 3.244 |
| 911 | 218 | 20 | 1mun−2abk | 1mun_8 | 0.879 | 3.431 |
| 911 | 218 | 20 | 1mun−2abk | 1mun_9 | 0.553 | 3.722 |
| 911 | 218 | 20 | 1mun−2abk | 1mun_10 | 0.951 | 3.333 |
| 911 | 218 | 20 | 2abk−1mun | 2abk_1 | 0.956 | 2.750 |
| 911 | 218 | 20 | 2abk−1mun | 2abk_2 | 0.917 | 2.688 |
| 911 | 218 | 20 | 2abk−1mun | 2abk_3 | 0.917 | 2.942 |
| 911 | 218 | 20 | 2abk−1mun | 2abk_4 | 0.893 | 3.181 |
| 911 | 218 | 20 | 2abk−1mun | 2abk_5 | 0.801 | 4.420 |
| 915 | 120 | 20 | 4rhn−4fit | 4rhn_1 | 0.821 | 2.605 |
| 915 | 120 | 20 | 4rhn−4fit | 4rhn_2 | 0.717 | 3.586 |
| 915 | 120 | 20 | 4rhn−4fit | 4rhn_3 | 0.726 | 3.451 |
| 915 | 120 | 20 | 4rhn−4fit | 4rhn_4 | 0.708 | 3.729 |
| 915 | 120 | 20 | 4rhn−4fit | 4rhn_5 | 0.726 | 3.734 |
| 915 | 120 | 20 | 4rhn−4fit | 4rhn_6 | 0.717 | 4.513 |
| 915 | 120 | 20 | 4rhn−4fit | 4rhn_7 | 0.755 | 4.256 |
| 915 | 120 | 20 | 4rhn−4fit | 4rhn_8 | 0.726 | 4.284 |
| 915 | 120 | 20 | 4rhn−4fit | 4rhn_9 | 0.764 | 4.480 |
| 915 | 120 | 20 | 4rhn−4fit | 4rhn_10 | 0.755 | 4.586 |
| 921 | 69 | 20 | 1tafb−1tafa | 1tafb_1 | 1.000 | 1.920 |
| 921 | 69 | 20 | 1tafb−1tafa | 1tafb_2 | 1.000 | 2.865 |
| 921 | 69 | 20 | 1tafb−1tafa | 1tafb_3 | 1.000 | 4.332 |
| 921 | 69 | 20 | 1tafb−1tafa | 1tafb_4 | 0.754 | 5.056 |
| 921 | 69 | 20 | 1tafb−1tafa | 1tafb_5 | 0.754 | 4.786 |
| 921 | 69 | 20 | 1tafb−1tafa | 1tafb_6 | 0.323 | 4.917 |
| 921 | 69 | 20 | 1tafb−1tafa | 1tafb_7 | 0.508 | 5.458 |
| 921 | 69 | 20 | 1tafb−1tafa | 1tafb_8 | 0.323 | 5.804 |
| 921 | 69 | 20 | 1tafb−1tafa | 1tafb_9 | 0.462 | 6.163 |
| 921 | 69 | 20 | 1tafb−1tafa | 1tafb_10 | 0.323 | 5.623 |
| 921 | 69 | 20 | 1tafb−1tafa | 1tafa_1 | 1.000 | 2.493 |
| 921 | 69 | 20 | 1tafb−1tafa | 1tafa_2 | 0.908 | 2.728 |
| 921 | 69 | 20 | 1tafb−1tafa | 1tafa_3 | 0.908 | 3.459 |
| 921 | 69 | 20 | 1tafb−1tafa | 1tafa_4 | 1.000 | 3.256 |
| 921 | 69 | 20 | 1tafb−1tafa | 1tafa_5 | 1.000 | 3.309 |
| 921 | 69 | 20 | 1tafb−1tafa | 1tafa_6 | 0.723 | 3.983 |
| 921 | 69 | 20 | 1tafb−1tafa | 1tafa_7 | 0.723 | 5.123 |
| 921 | 69 | 20 | 1tafb−1tafa | 1tafa_8 | 0.677 | 5.652 |
| 921 | 69 | 20 | 1tafb−1tafa | 1tafa_9 | 0.677 | 5.563 |
| 921 | 69 | 20 | 1tafb−1tafa | 1tafa_10 | 0.600 | 6.273 |
| 926 | 66 | 19 | 1jt6−2tct | 1jt6_1 | 0.938 | 1.346 |
| 927 | 123 | 19 | 1cgp−1rgs | 1cgp_1 | 0.905 | 1.827 |
| 927 | 123 | 19 | 1cgp−1rgs | 1cgp_2 | 0.957 | 1.676 |
| 927 | 123 | 19 | 1cgp−1rgs | 1cgp_3 | 0.914 | 1.714 |
| 927 | 123 | 19 | 1cgp−1rgs | 1cgp_4 | 0.905 | 1.725 |
| 927 | 123 | 19 | 1cgp−1rgs | 1cgp_5 | 1.000 | 2.002 |
| 927 | 123 | 19 | 1cgp−1rgs | 1cgp_6 | 0.966 | 1.868 |
| 927 | 123 | 19 | 1cgp−1rgs | 1cgp_7 | 0.957 | 1.892 |
| 927 | 123 | 19 | 1cgp−1rgs | 1cgp_8 | 0.957 | 2.033 |
| 927 | 123 | 19 | 1cgp−1rgs | 1cgp_9 | 0.966 | 1.818 |
| 927 | 123 | 19 | 1cgp−1rgs | 1cgp_10 | 0.905 | 1.994 |
| 932 | 250 | 19 | 1hyo−1gtt | 1hyo_1 | 0.995 | 1.553 |
| 932 | 250 | 19 | 1hyo−1gtt | 1hyo_2 | 0.974 | 1.732 |
| 933 | 399 | 19 | 1bhe−1rmg | 1bhe_1 | 0.954 | 1.781 |
| 933 | 399 | 19 | 1bhe−1rmg | 1bhe_2 | 0.951 | 1.943 |
| 933 | 399 | 19 | 1bhe−1rmg | 1bhe_3 | 0.957 | 1.865 |
| 933 | 399 | 19 | 1bhe−1rmg | 1bhe_4 | 0.960 | 1.900 |
| 933 | 399 | 19 | 1bhe−1rmg | 1bhe_5 | 0.954 | 2.177 |
| 933 | 399 | 19 | 1bhe−1rmg | 1bhe_6 | 0.960 | 1.963 |
| 933 | 399 | 19 | 1bhe−1rmg | 1bhe_7 | 0.957 | 2.215 |
| 933 | 399 | 19 | 1bhe−1rmg | 1bhe_8 | 0.942 | 2.274 |
| 933 | 399 | 19 | 1bhe−1rmg | 1bhe_9 | 0.939 | 2.180 |
| 933 | 399 | 19 | 1bhe−1rmg | 1bhe_10 | 0.957 | 2.051 |
| 933 | 399 | 19 | 1rmg−1bhe | 1rmg_1 | 0.966 | 1.872 |
| 933 | 399 | 19 | 1rmg−1bhe | 1rmg_2 | 0.942 | 2.148 |
| 933 | 399 | 19 | 1rmg−1bhe | 1rmg_3 | 0.954 | 2.381 |
| 933 | 399 | 19 | 1rmg−1bhe | 1rmg_4 | 0.972 | 2.167 |
| 933 | 399 | 19 | 1rmg−1bhe | 1rmg_5 | 0.957 | 1.843 |
| 933 | 399 | 19 | 1rmg−1bhe | 1rmg_6 | 0.957 | 2.232 |
| 935 | 184 | 19 | 1d0b−1a9n | 1d0b_1 | 0.871 | 2.229 |
| 935 | 184 | 19 | 1d0b−1a9n | 1d0b_2 | 0.023 | 2.232 |
| 935 | 184 | 19 | 1d0b−1a9n | 1d0b_3 | 0.045 | 2.241 |
| 935 | 184 | 19 | 1d0b−1a9n | 1d0b_4 | 0.045 | 2.172 |
| 935 | 184 | 19 | 1d0b−1a9n | 1d0b_5 | 0.023 | 2.403 |
| 935 | 184 | 19 | 1d0b−1a9n | 1d0b_6 | 0.045 | 2.745 |
| 935 | 184 | 19 | 1d0b−1a9n | 1d0b_7 | 0.045 | 2.387 |
| 935 | 184 | 19 | 1d0b−1a9n | 1d0b_8 | 0.030 | 2.535 |
| 935 | 184 | 19 | 1d0b−1a9n | 1d0b_9 | 0.023 | 2.366 |
| 935 | 184 | 19 | 1d0b−1a9n | 1d0b_10 | 0.856 | 2.513 |
| 936 | 426 | 19 | 1jki−1gr0 | 1jki_1 | 0.949 | 1.911 |
| 938 | 107 | 19 | 1bxy−1jj2 | 1bxy_1 | 1.000 | 1.186 |
| 938 | 107 | 19 | 1bxy−1jj2 | 1bxy_2 | 1.000 | 1.895 |
| 938 | 107 | 19 | 1bxy−1jj2 | 1bxy_3 | 1.000 | 1.966 |
| 938 | 107 | 19 | 1bxy−1jj2 | 1bxy_4 | 1.000 | 2.225 |
| 938 | 107 | 19 | 1bxy−1jj2 | 1bxy_5 | 1.000 | 1.416 |
| 938 | 107 | 19 | 1bxy−1jj2 | 1bxy_6 | 1.000 | 1.889 |
| 938 | 107 | 19 | 1bxy−1jj2 | 1bxy_7 | 1.000 | 2.120 |
| 938 | 107 | 19 | 1bxy−1jj2 | 1bxy_8 | 1.000 | 1.876 |
| 938 | 107 | 19 | 1bxy−1jj2 | 1bxy_9 | 1.000 | 1.897 |
| 938 | 107 | 19 | 1bxy−1jj2 | 1bxy_10 | 1.000 | 1.866 |
| 945 | 113 | 18 | 1qts−1e42 | 1qts_1 | 1.000 | 1.642 |
| 945 | 113 | 18 | 1qts−1e42 | 1qts_2 | 0.897 | 1.953 |
| 945 | 113 | 18 | 1qts−1e42 | 1qts_3 | 0.963 | 2.151 |
| 945 | 113 | 18 | 1qts−1e42 | 1qts_4 | 0.907 | 1.901 |
| 945 | 113 | 18 | 1qts−1e42 | 1qts_5 | 0.907 | 2.208 |
| 945 | 113 | 18 | 1qts−1e42 | 1qts_6 | 0.925 | 2.234 |
| 945 | 113 | 18 | 1qts−1e42 | 1qts_7 | 0.925 | 2.378 |
| 946 | 240 | 18 | 1jfl−1b74 | 1jfl_1 | 0.937 | 1.688 |
| 946 | 240 | 18 | 1jfl−1b74 | 1jfl_2 | 0.893 | 2.201 |
| 946 | 240 | 18 | 1jfl−1b74 | 1jfl_3 | 0.908 | 2.802 |
| 946 | 240 | 18 | 1jfl−1b74 | 1jfl_4 | 0.864 | 3.133 |
| 946 | 240 | 18 | 1jfl−1b74 | 1jfl_5 | 0.927 | 2.875 |
| 946 | 240 | 18 | 1b74−1jfl | 1b74_1 | 0.922 | 2.199 |
| 946 | 240 | 18 | 1b74−1jfl | 1b74_2 | 0.883 | 2.458 |
| 946 | 240 | 18 | 1b74−1jfl | 1b74_3 | 0.850 | 2.915 |
| 946 | 240 | 18 | 1b74−1jfl | 1b74_4 | 0.874 | 2.987 |
| 946 | 240 | 18 | 1b74−1jfl | 1b74_5 | 0.879 | 3.124 |
| 946 | 240 | 18 | 1b74−1jfl | 1b74_6 | 0.850 | 3.499 |
| 946 | 240 | 18 | 1b74−1jfl | 1b74_7 | 0.854 | 3.424 |
| 946 | 240 | 18 | 1b74−1jfl | 1b74_8 | 0.801 | 3.916 |
| 946 | 240 | 18 | 1b74−1jfl | 1b74_9 | 0.791 | 3.942 |
| 946 | 240 | 18 | 1b74−1jfl | 1b74_10 | 0.752 | 4.108 |
| 951 | 174 | 18 | 1h72−1fi4 | 1h72_1 | 0.830 | 1.477 |
| 951 | 174 | 18 | 1h72−1fi4 | 1h72_2 | 0.850 | 2.069 |
| 951 | 174 | 18 | 1h72−1fi4 | 1h72_3 | 0.912 | 2.175 |
| 951 | 174 | 18 | 1h72−1fi4 | 1h72_4 | 0.857 | 2.310 |
| 951 | 174 | 18 | 1h72−1fi4 | 1h72_5 | 0.878 | 1.948 |
| 951 | 174 | 18 | 1h72−1fi4 | 1h72_6 | 0.837 | 2.075 |
| 951 | 174 | 18 | 1h72−1fi4 | 1h72_7 | 0.891 | 1.782 |
| 951 | 174 | 18 | 1h72−1fi4 | 1h72_8 | 0.946 | 1.672 |
| 951 | 174 | 18 | 1h72−1fi4 | 1h72_9 | 0.837 | 1.869 |
| 951 | 174 | 18 | 1h72−1fi4 | 1h72_10 | 0.884 | 1.894 |
| 962 | 367 | 18 | 1ju3−1lns | 1ju3_1 | 0.904 | 2.508 |
| 962 | 367 | 18 | 1ju3−1lns | 1ju3_2 | 0.874 | 2.492 |
| 962 | 367 | 18 | 1ju3−1lns | 1ju3_3 | 0.870 | 2.532 |
| 962 | 367 | 18 | 1ju3−1lns | 1ju3_4 | 0.894 | 2.741 |
| 962 | 367 | 18 | 1lns−1ju3 | 1lns_1 | 0.874 | 1.401 |
| 962 | 367 | 18 | 1lns−1ju3 | 1lns_2 | 0.915 | 1.434 |
| 971 | 419 | 17 | 1pbe−1foh | 1pbe_1 | 0.853 | 2.351 |
| 971 | 419 | 17 | 1pbe−1foh | 1pbe_2 | 0.826 | 2.494 |
| 971 | 419 | 17 | 1pbe−1foh | 1pbe_3 | 0.832 | 2.242 |
| 971 | 419 | 17 | 1pbe−1foh | 1pbe_4 | 0.886 | 2.349 |
| 971 | 419 | 17 | 1pbe−1foh | 1pbe_5 | 0.832 | 2.331 |
| 971 | 419 | 17 | 1pbe−1foh | 1pbe_6 | 0.886 | 2.747 |
| 971 | 419 | 17 | 1pbe−1foh | 1pbe_7 | 0.804 | 3.105 |
| 971 | 419 | 17 | 1pbe−1foh | 1pbe_8 | 0.736 | 2.951 |
| 971 | 419 | 17 | 1pbe−1foh | 1pbe_9 | 0.851 | 2.805 |
| 971 | 419 | 17 | 1pbe−1foh | 1pbe_10 | 0.812 | 2.492 |
| 973 | 269 | 17 | 1h2w−1n1m | 1h2w_1 | 0.964 | 1.538 |
| 973 | 269 | 17 | 1h2w−1n1m | 1h2w_2 | 0.960 | 1.506 |
| 973 | 269 | 17 | 1h2w−1n1m | 1h2w_3 | 0.929 | 1.866 |
| 973 | 269 | 17 | 1h2w−1n1m | 1h2w_4 | 0.945 | 1.869 |
| 976 | 400 | 17 | 1kas−1afw | 1kas_1 | 0.821 | 1.935 |
| 976 | 400 | 17 | 1kas−1afw | 1kas_2 | 0.794 | 1.937 |
| 976 | 400 | 17 | 1kas−1afw | 1kas_3 | 0.791 | 2.354 |
| 976 | 400 | 17 | 1kas−1afw | 1kas_4 | 0.767 | 2.457 |
| 976 | 400 | 17 | 1kas−1afw | 1kas_5 | 0.858 | 2.635 |
| 978 | 360 | 17 | 2vsg−1vsg | 2vsg_1 | 0.831 | 1.965 |
| 978 | 360 | 17 | 2vsg−1vsg | 2vsg_2 | 0.819 | 2.617 |
| 978 | 360 | 17 | 2vsg−1vsg | 2vsg_3 | 0.792 | 2.936 |
| 978 | 360 | 17 | 2vsg−1vsg | 2vsg_4 | 0.764 | 2.732 |
| 984 | 367 | 16 | 1f0k−1iir | 1f0k_1 | 0.875 | 2.443 |
| 984 | 367 | 16 | 1f0k−1iir | 1f0k_2 | 0.850 | 2.390 |
| 988 | 122 | 16 | 1dqe−1c3z | 1dqe_1 | 0.565 | 1.955 |
| 988 | 122 | 16 | 1dqe−1c3z | 1dqe_2 | 0.565 | 2.616 |
| 988 | 122 | 16 | 1dqe−1c3z | 1dqe_3 | 0.593 | 2.900 |
| 988 | 122 | 16 | 1dqe−1c3z | 1dqe_4 | 0.713 | 3.046 |
| 988 | 122 | 16 | 1dqe−1c3z | 1dqe_5 | 0.407 | 3.248 |
| 988 | 122 | 16 | 1dqe−1c3z | 1dqe_6 | 0.630 | 3.187 |
| 988 | 122 | 16 | 1c3z−1dqe | 1c3z_1 | 0.722 | 3.785 |
| 988 | 122 | 16 | 1c3z−1dqe | 1c3z_2 | 0.556 | 5.303 |
| 988 | 122 | 16 | 1c3z−1dqe | 1c3z_3 | 0.713 | 5.429 |
| 988 | 122 | 16 | 1c3z−1dqe | 1c3z_4 | 0.528 | 5.731 |
| 988 | 122 | 16 | 1c3z−1dqe | 1c3z_5 | 0.731 | 6.124 |
| 988 | 122 | 16 | 1c3z−1dqe | 1c3z_6 | 0.731 | 6.576 |
| 988 | 122 | 16 | 1c3z−1dqe | 1c3z_7 | 0.750 | 6.439 |
| 988 | 122 | 16 | 1c3z−1dqe | 1c3z_8 | 0.741 | 6.188 |
| 988 | 122 | 16 | 1c3z−1dqe | 1c3z_9 | 0.731 | 6.180 |
| 988 | 122 | 16 | 1c3z−1dqe | 1c3z_10 | 0.750 | 6.175 |
| 991 | 94 | 16 | 1ctn−1b4r | 1ctn_1 | 0.987 | 1.862 |
| 991 | 94 | 16 | 1ctn−1b4r | 1ctn_2 | 1.000 | 2.067 |
| 991 | 94 | 16 | 1b4r−1ctn | 1b4r_1 | 0.886 | 2.101 |
| 991 | 94 | 16 | 1b4r−1ctn | 1b4r_2 | 0.886 | 2.952 |
| 991 | 94 | 16 | 1b4r−1ctn | 1b4r_3 | 0.861 | 3.151 |
| 991 | 94 | 16 | 1b4r−1ctn | 1b4r_4 | 0.886 | 3.300 |
| 991 | 94 | 16 | 1b4r−1ctn | 1b4r_5 | 0.873 | 2.933 |
| 991 | 94 | 16 | 1b4r−1ctn | 1b4r_6 | 0.823 | 3.001 |
| 991 | 94 | 16 | 1b4r−1ctn | 1b4r_7 | 0.937 | 3.627 |
| 991 | 94 | 16 | 1b4r−1ctn | 1b4r_8 | 0.329 | 3.088 |
| 991 | 94 | 16 | 1b4r−1ctn | 1b4r_9 | 0.886 | 3.190 |
| 991 | 94 | 16 | 1b4r−1ctn | 1b4r_10 | 0.785 | 3.188 |
| 992 | 157 | 16 | 1ez3−1fio | 1ez3_1 | 0.991 | 3.737 |
| 995 | 228 | 15 | 1h7e−1ezi | 1h7e_1 | 0.942 | 2.068 |
| 995 | 228 | 15 | 1h7e−1ezi | 1h7e_2 | 0.879 | 2.521 |
| 995 | 228 | 15 | 1h7e−1ezi | 1h7e_3 | 0.916 | 2.732 |
| 998 | 176 | 15 | 1eje−1i0r | 1eje_1 | 0.841 | 7.187 |
| 998 | 176 | 15 | 1eje−1i0r | 1eje_2 | 0.898 | 7.809 |
| 998 | 176 | 15 | 1eje−1i0r | 1eje_3 | 0.879 | 9.011 |
| 998 | 176 | 15 | 1eje−1i0r | 1eje_4 | 0.809 | 9.417 |
| 998 | 176 | 15 | 1eje−1i0r | 1eje_5 | 0.783 | 9.295 |
| 998 | 176 | 15 | 1eje−1i0r | 1eje_6 | 0.847 | 9.194 |
| 998 | 176 | 15 | 1eje−1i0r | 1eje_7 | 0.777 | 9.312 |
| 998 | 176 | 15 | 1eje−1i0r | 1eje_8 | 0.904 | 9.438 |
| 998 | 176 | 15 | 1eje−1i0r | 1eje_9 | 0.790 | 8.185 |
| 998 | 176 | 15 | 1eje−1i0r | 1eje_10 | 0.828 | 7.653 |
| 998 | 176 | 15 | 1i0r−1eje | 1i0r_1 | 0.892 | 1.364 |
| 998 | 176 | 15 | 1i0r−1eje | 1i0r_2 | 0.924 | 1.708 |
| 998 | 176 | 15 | 1i0r−1eje | 1i0r_3 | 0.885 | 1.622 |
| 998 | 176 | 15 | 1i0r−1eje | 1i0r_4 | 0.924 | 1.784 |
| 998 | 176 | 15 | 1i0r−1eje | 1i0r_5 | 0.892 | 1.493 |
| 998 | 176 | 15 | 1i0r−1eje | 1i0r_6 | 0.904 | 1.853 |
| 998 | 176 | 15 | 1i0r−1eje | 1i0r_7 | 0.898 | 1.676 |
| 998 | 176 | 15 | 1i0r−1eje | 1i0r_8 | 0.917 | 1.908 |
| 998 | 176 | 15 | 1i0r−1eje | 1i0r_9 | 0.879 | 1.739 |
| 998 | 176 | 15 | 1i0r−1eje | 1i0r_10 | 0.885 | 1.915 |
| 1002 | 220 | 15 | 1nba−1yac | 1nba_1 | 0.955 | 1.502 |
| 1002 | 220 | 15 | 1nba−1yac | 1nba_2 | 0.921 | 1.623 |

n: internal numbering; length of alignment; % sequence%ID (cf. Homstrad); refA−refB: reference alignment; altA: alternative snapshot for A; SP: sum−of−pairs score of alt. alignment w/r reference

| n | length | %ID | refA−refB | altA | SP | RMSD |
|---|---|---|---|---|---|---|
| 1002 | 220 | 15 | 1nba−1yac | 1nba_3 | 0.966 | 2.111 |
| 1002 | 220 | 15 | 1nba−1yac | 1nba_4 | 0.944 | 2.557 |
| 1002 | 220 | 15 | 1yac−1nba | 1yac_1 | 1.000 | 1.413 |
| 1002 | 220 | 15 | 1yac−1nba | 1yac_2 | 0.949 | 1.945 |
| 1002 | 220 | 15 | 1yac−1nba | 1yac_3 | 0.989 | 1.793 |
| 1005 | 48 | 15 | 1vpc−1ceu | 1vpc_1 | 0.789 | 3.942 |
| 1005 | 48 | 15 | 1vpc−1ceu | 1vpc_2 | 0.289 | 6.392 |
| 1005 | 48 | 15 | 1vpc−1ceu | 1vpc_3 | 0.000 | 8.481 |
| 1005 | 48 | 15 | 1vpc−1ceu | 1vpc_4 | 0.000 | 8.730 |
| 1005 | 48 | 15 | 1vpc−1ceu | 1vpc_5 | 0.000 | 8.903 |
| 1005 | 48 | 15 | 1vpc−1ceu | 1vpc_6 | 0.000 | 8.596 |
| 1005 | 48 | 15 | 1vpc−1ceu | 1vpc_7 | 0.000 | 8.982 |
| 1005 | 48 | 15 | 1vpc−1ceu | 1vpc_8 | 0.000 | 7.582 |
| 1005 | 48 | 15 | 1vpc−1ceu | 1vpc_9 | 0.000 | 6.384 |
| 1005 | 48 | 15 | 1vpc−1ceu | 1vpc_10 | 0.000 | 8.397 |
| 1005 | 48 | 15 | 1ceu−1vpc | 1ceu_1 | 0.000 | 5.056 |
| 1005 | 48 | 15 | 1ceu−1vpc | 1ceu_2 | 0.000 | 8.077 |
| 1005 | 48 | 15 | 1ceu−1vpc | 1ceu_3 | 0.026 | 8.495 |
| 1005 | 48 | 15 | 1ceu−1vpc | 1ceu_4 | 0.105 | 5.733 |
| 1005 | 48 | 15 | 1ceu−1vpc | 1ceu_5 | 0.000 | 7.593 |
| 1005 | 48 | 15 | 1ceu−1vpc | 1ceu_6 | 0.000 | 7.465 |
| 1005 | 48 | 15 | 1ceu−1vpc | 1ceu_7 | 0.368 | 7.378 |
| 1005 | 48 | 15 | 1ceu−1vpc | 1ceu_8 | 0.000 | 7.869 |
| 1005 | 48 | 15 | 1ceu−1vpc | 1ceu_9 | 0.000 | 7.697 |
| 1005 | 48 | 15 | 1ceu−1vpc | 1ceu_10 | 0.368 | 9.041 |
| 1006 | 90 | 14 | 1psd−1phz | 1psd_1 | 0.940 | 1.363 |
| 1008 | 96 | 14 | 3crd−1cy5 | 3crd_1 | 0.528 | 4.147 |
| 1008 | 96 | 14 | 3crd−1cy5 | 3crd_2 | 0.539 | 4.761 |
| 1008 | 96 | 14 | 3crd−1cy5 | 3crd_3 | 0.494 | 4.678 |
| 1008 | 96 | 14 | 3crd−1cy5 | 3crd_4 | 0.640 | 4.768 |
| 1008 | 96 | 14 | 3crd−1cy5 | 3crd_5 | 0.787 | 5.211 |
| 1008 | 96 | 14 | 3crd−1cy5 | 3crd_6 | 0.775 | 5.028 |
| 1008 | 96 | 14 | 1cy5−3crd | 1cy5_1 | 0.944 | 1.895 |
| 1008 | 96 | 14 | 1cy5−3crd | 1cy5_2 | 0.910 | 1.731 |
| 1008 | 96 | 14 | 1cy5−3crd | 1cy5_3 | 0.809 | 2.256 |
| 1008 | 96 | 14 | 1cy5−3crd | 1cy5_4 | 0.685 | 2.810 |
| 1008 | 96 | 14 | 1cy5−3crd | 1cy5_5 | 0.697 | 3.005 |
| 1008 | 96 | 14 | 1cy5−3crd | 1cy5_6 | 0.697 | 3.064 |
| 1008 | 96 | 14 | 1cy5−3crd | 1cy5_7 | 0.685 | 3.158 |
| 1008 | 96 | 14 | 1cy5−3crd | 1cy5_8 | 0.685 | 3.644 |
| 1008 | 96 | 14 | 1cy5−3crd | 1cy5_9 | 0.629 | 3.061 |
| 1008 | 96 | 14 | 1cy5−3crd | 1cy5_10 | 0.685 | 3.312 |
| 1009 | 136 | 14 | 1bwza2−1bwza1 | 1bwza2_1 | 0.927 | 1.783 |
| 1009 | 136 | 14 | 1bwza2−1bwza1 | 1bwza2_2 | 0.902 | 1.553 |
| 1009 | 136 | 14 | 1bwza2−1bwza1 | 1bwza2_3 | 0.878 | 1.643 |
| 1009 | 136 | 14 | 1bwza2−1bwza1 | 1bwza2_4 | 0.902 | 1.766 |
| 1009 | 136 | 14 | 1bwza2−1bwza1 | 1bwza2_5 | 0.813 | 1.924 |
| 1009 | 136 | 14 | 1bwza2−1bwza1 | 1bwza2_6 | 0.837 | 1.864 |
| 1009 | 136 | 14 | 1bwza2−1bwza1 | 1bwza2_7 | 0.878 | 1.952 |
| 1009 | 136 | 14 | 1bwza2−1bwza1 | 1bwza2_8 | 0.862 | 1.962 |
| 1009 | 136 | 14 | 1bwza2−1bwza1 | 1bwza2_9 | 0.935 | 1.858 |
| 1009 | 136 | 14 | 1bwza2−1bwza1 | 1bwza1_1 | 0.854 | 1.705 |
| 1009 | 136 | 14 | 1bwza2−1bwza1 | 1bwza1_3 | 0.911 | 2.165 |
| 1009 | 136 | 14 | 1bwza2−1bwza1 | 1bwza1_4 | 0.846 | 2.229 |
| 1009 | 136 | 14 | 1bwza2−1bwza1 | 1bwza1_5 | 0.902 | 2.119 |
| 1009 | 136 | 14 | 1bwza2−1bwza1 | 1bwza1_6 | 0.878 | 2.238 |
| 1009 | 136 | 14 | 1bwza2−1bwza1 | 1bwza1_7 | 0.878 | 2.375 |
| 1009 | 136 | 14 | 1bwza2−1bwza1 | 1bwza1_8 | 0.797 | 2.625 |
| 1009 | 136 | 14 | 1bwza2−1bwza1 | 1bwza1_9 | 0.846 | 2.648 |
| 1013 | 229 | 13 | 1bd0−1ct5 | 1bd0_1 | 0.902 | 1.567 |
| 1013 | 229 | 13 | 1bd0−1ct5 | 1bd0_2 | 0.881 | 1.995 |
| 1013 | 229 | 13 | 1bd0−1ct5 | 1bd0_3 | 0.912 | 2.133 |
| 1013 | 229 | 13 | 1bd0−1ct5 | 1bd0_4 | 0.866 | 2.351 |
| 1013 | 229 | 13 | 1bd0−1ct5 | 1bd0_5 | 0.840 | 2.604 |
| 1013 | 229 | 13 | 1bd0−1ct5 | 1bd0_6 | 0.856 | 2.751 |
| 1015 | 51 | 13 | 1svfb−1svfa | 1svfb_1 | 0.868 | 3.719 |
| 1015 | 51 | 13 | 1svfb−1svfa | 1svfa_1 | 0.316 | 3.870 |
| 1016 | 89 | 13 | 1bht−1hky | 1bht_1 | 0.870 | 1.592 |
| 1016 | 89 | 13 | 1bht−1hky | 1bht_2 | 0.961 | 1.996 |
| 1016 | 89 | 13 | 1bht−1hky | 1bht_3 | 0.987 | 1.921 |
| 1016 | 89 | 13 | 1bht−1hky | 1bht_4 | 0.922 | 2.239 |

| n | length | %ID | refA−refB | altA | SP | RMSD |
|---|---|---|---|---|---|---|
| 1016 | 89 | 13 | 1bht−1hky | 1bht_5 | 0.922 | 2.593 |
| 1016 | 89 | 13 | 1bht−1hky | 1bht_6 | 0.818 | 2.792 |
| 1016 | 89 | 13 | 1bht−1hky | 1bht_7 | 0.831 | 2.628 |
| 1016 | 89 | 13 | 1bht−1hky | 1bht_8 | 0.922 | 2.630 |
| 1016 | 89 | 13 | 1bht−1hky | 1bht_9 | 0.831 | 2.711 |
| 1016 | 89 | 13 | 1bht−1hky | 1bht_10 | 0.883 | 3.043 |
| 1016 | 89 | 13 | 1hky−1bht | 1hky_1 | 0.883 | 4.390 |
| 1016 | 89 | 13 | 1hky−1bht | 1hky_2 | 0.701 | 4.549 |
| 1016 | 89 | 13 | 1hky−1bht | 1hky_3 | 0.753 | 3.934 |
| 1016 | 89 | 13 | 1hky−1bht | 1hky_4 | 0.623 | 4.050 |
| 1016 | 89 | 13 | 1hky−1bht | 1hky_5 | 0.818 | 4.961 |
| 1016 | 89 | 13 | 1hky−1bht | 1hky_6 | 0.818 | 7.343 |
| 1016 | 89 | 13 | 1hky−1bht | 1hky_7 | 0.831 | 7.006 |
| 1016 | 89 | 13 | 1hky−1bht | 1hky_8 | 0.870 | 7.277 |
| 1016 | 89 | 13 | 1hky−1bht | 1hky_9 | 0.818 | 7.168 |
| 1016 | 89 | 13 | 1hky−1bht | 1hky_10 | 0.831 | 7.115 |
| 1018 | 183 | 13 | 1moq2−1moq1 | 1moq2_1 | 0.951 | 2.056 |
| 1018 | 183 | 13 | 1moq2−1moq1 | 1moq2_2 | 0.871 | 1.982 |
| 1018 | 183 | 13 | 1moq2−1moq1 | 1moq2_3 | 0.865 | 2.134 |
| 1018 | 183 | 13 | 1moq2−1moq1 | 1moq2_4 | 0.816 | 2.308 |
| 1018 | 183 | 13 | 1moq2−1moq1 | 1moq2_5 | 0.822 | 2.352 |
| 1018 | 183 | 13 | 1moq2−1moq1 | 1moq2_6 | 0.798 | 2.597 |
| 1018 | 183 | 13 | 1moq2−1moq1 | 1moq1_1 | 0.920 | 1.664 |
| 1018 | 183 | 13 | 1moq2−1moq1 | 1moq1_2 | 0.840 | 2.082 |
| 1018 | 183 | 13 | 1moq2−1moq1 | 1moq1_3 | 0.816 | 2.343 |
| 1018 | 183 | 13 | 1moq2−1moq1 | 1moq1_4 | 0.798 | 2.427 |
| 1018 | 183 | 13 | 1moq2−1moq1 | 1moq1_5 | 0.853 | 2.360 |
| 1018 | 183 | 13 | 1moq2−1moq1 | 1moq1_6 | 0.791 | 2.470 |
| 1019 | 129 | 13 | 2sak−1qqr | 2sak_1 | 0.928 | 2.002 |
| 1019 | 129 | 13 | 2sak−1qqr | 2sak_2 | 0.973 | 2.019 |
| 1019 | 129 | 13 | 2sak−1qqr | 2sak_3 | 0.946 | 2.758 |
| 1019 | 129 | 13 | 2sak−1qqr | 2sak_4 | 0.865 | 3.128 |
| 1019 | 129 | 13 | 2sak−1qqr | 2sak_5 | 0.928 | 4.055 |
| 1019 | 129 | 13 | 2sak−1qqr | 2sak_6 | 0.919 | 4.305 |
| 1019 | 129 | 13 | 2sak−1qqr | 2sak_7 | 0.865 | 3.115 |
| 1025 | 646 | 11 | 3pfl−1b8b | 3pfl_1 | 0.907 | 1.931 |
| 1025 | 646 | 11 | 3pfl−1b8b | 3pfl_2 | 0.770 | 2.085 |
| 1029 | 147 | 9 | 1uox2−1uox1 | 1uox2_1 | 0.934 | 3.797 |
| 1029 | 147 | 9 | 1uox2−1uox1 | 1uox2_2 | 0.926 | 4.590 |
| 1029 | 147 | 9 | 1uox2−1uox1 | 1uox2_3 | 0.831 | 5.716 |
| 1029 | 147 | 9 | 1uox2−1uox1 | 1uox1_1 | 0.904 | 3.683 |
| 1029 | 147 | 9 | 1uox2−1uox1 | 1uox1_2 | 0.912 | 4.476 |
| 1029 | 147 | 9 | 1uox2−1uox1 | 1uox1_3 | 0.926 | 4.837 |

n: internal numbering; length of alignment; % sequence%ID (cf. Homstrad); refA−refB: reference alignment; altA: alternative snapshot for A; SP: sum−of−pairs score of alt. alignment w/r reference
